# Integrative analyses reveal the evolution of the Old World Swallowtail in the Palearctic

**DOI:** 10.1371/journal.pone.0343793

**Published:** 2026-07-08

**Authors:** Valentina Todisco, Jennifer F. Hoyal Cuthill, Anatoly Krupitsky, Galina Shapoval, Boris Nalitkin, Anna Romanovich, Raluca Vodă, Marko Mutanen, Roger Vila, Nazar Shapoval, Vazrick Nazari

**Affiliations:** 1 Department of Environment and Biodiversity, Evolutionary Zoology, University of Salzburg, Salzburg, Austria; 2 School of Life Sciences, University of Essex, Colchester, United Kingdom; 3 Department of Entomology, Faculty of Biology, Lomonosov Moscow State University, Moscow, Russia; 4 Severtsov Institute of Ecology and Evolution, Russian Academy of Sciences, Moscow, Russia; 5 Department of Karyosystematics, Zoological Institute, Russian Academy of Sciences, St. Petersburg, Russia; 6 Lazorevy proezd, Moscow, Russia; 7 Resource Center for Development of Molecular and Cellular Technologies, St. Petersburg State University, St. Petersburg, Russia; 8 Naturéum, State Museum of Natural Sciences, Palais de Rumine, Lausanne, Switzerland; 9 Ecology and Genetics Research Unit, University of Oulu, Oulu, Finland; 10 Institut de Biologia Evolutiva (CSIC-UPF), Barcelona, Spain; 11 Department of Karyosystematics, Zoological Institute, Russian Academy of Sciences, St. Petersburg, Russia; 12 Department of Biology, University of Padova, Padova, Italy; Sunrise University, INDIA

## Abstract

Based on a dataset of 585 samples originating from across the Palearctic region and four genes (COI, CAD, Ca-ATPase, and 28S) for a subset of these samples, we studied the phylogenetic relationships within and between the Old World Swallowtail (*Papilio machaon* Linnaeus, 1758) and its closest relatives in the Palearctic region. We used Machine Learning (ML) to investigate the concordance of morphological characteristics with the molecular data. Our phylogenetic analyses showed that the Palearctic taxa *everesti*, *archias* and *hippocrates* formed strongly supported sister-group relationships with *P. machaon*. Within the Palearctic *machaon* a few distinct mitochondrial linages were observed, including one from the Himalayas and Central Asia, and another one that included all of the north African samples (taxa *mauretanica*, *saharae* and *neosaharae*) without any distinction. ML clustering was supportive of the haplotype and geographic analyses and a positive correlation was measured between average genetic phylogenetic and machine learnt specimen-image distances. Screening for *Wolbachia* revealed infection only in females of *P. archias.* We present a biogeographic scenario for the evolution of the *P. machaon* species group in the Palearctic region.

## Introduction

In recent decades, taxonomic and phylogeographic studies on Holarctic insects have contributed immensely to our knowledge of speciation, zoogeography and history of the continental connections and have provided insights on the types of habitats that may have existed during these connections [1–9]. The effects of Pleistocene glaciations on the distribution and evolution of species inhabiting the Holarctic region are well studied [10,11]. In many organisms, morphological traits provide a phenotypic link between genotypes and the environment [12–15]. Only very recently, new methods for machine learning (ML) in evolution and ecology (reviewed in [16]) have enabled tests for inter-species phenotypic diversity and it can now be used to quantify variation across different butterfly groups [17,18]. Even though butterflies are among the best-studied invertebrates, comprehensive taxonomic and phylogeographic studies on widespread Holarctic species are still rare [19–23]. This is especially true for the Old World Swallowtail, *Papilio machaon* Linnaeus, 1758, an iconic butterfly that belongs to the large family of swallowtail butterflies (Papilionidae), famous for its beauty and unique in the genus *Papilio* for having a Holarctic distribution. *Papilio machaon* also represents the nominate species of the entire superfamily Papilionoidea (true butterflies) and hence is the “true” butterfly species by definition. Found throughout the entire Palearctic region and across Alaska and Canada, this species displays different ecological races occupying habitats as varied as the Arctic tundra, high altitude steppe, Saharan desert oases, temperate coastal forests, vegetable gardens, and citrus orchards.

Several closely related species are recognized under a *machaon* “species group” [(i.e., subgenus *Papilio* sensu stricto; see 24–33)], and the taxonomy of the group continues to be revised. In the Nearctic region, at least three subspecies of *P. machaon* (*aliaska* Scudder, 1869*, pikei* Sperling 1987, and *hudsonianus* Clark, 1932) are recognized, together with closely-related sister species *P. kahli* F. & R. Chermock, 1937 and *P. bairdii* Edwards, 1866, as well as some hybrid lineages with *P. polyxenes* Fabricius, 1775 (e.g., *P. brevicauda* Saunders, 1868, and *P. joanae* Heitzman, 1973) [34,35]. The latest treatment of this species complex in the Palearctic region has recognized *P. saharae* Oberthür, 1879*, P. everesti* Riley, 1927*, P. hospiton* Géné, 1839*, P. verityi* Fruhstorfer, 1907*, P. archias* Fruhstorfer, 1907, and *P. hippocrates* C. & R. Felder, 1864 as separate species, alongside 15 subspecies of *P. machaon* [36]. Taxa *P. hospiton, P. zelicaon* Lucas, 1852*, P. polyxenes*, and *P. indra* Reakirt, 1866 are generally recognized as sisters to the *machaon* species group, with the Asiatic *P. xuthus* Linnaeus, 1767 as a more distant relative [34,35]. Hybridization (natural and artificial) between many of these lineages has been extensively documented (e.g., [37–45]).

The uncertainty over the taxonomic boundaries in the *P. machaon* species group is to some extent due to discordant character variation, since wing and body color pattern, as well as wing shape, have been the main source of morphological characters used to taxonomically separate the taxa in this group. In fact, the presence of numerous forms, aberrations and color polymorphisms within populations has been a consistent source of taxonomic confusion [46]. Such a pattern reflects complex evolutionary history including range fragmentation, hybridization, and ecological race formation. Although extensive sympatry occurs between species in the Western United States and Canada, most other regions support either a single species, or a contact zone between two or more species maintained in part by habitat segregation [34,35,47].

Even though *P. machaon* is generally common and widespread throughout Eurasia and is not a threatened species, it has been listed as ‘vulnerable’ in the Red Data books of South Korea, Austria, and the former Soviet Union [48] and is protected by law in Czech Republic, Slovakia, Hungary, Romania, and Moldova. *P. m. britannicus* is the largest protected resident butterfly in the United Kingdom, where its range is limited only to a few areas in the Norfolk Broads of East Anglia [49]. The taxon *verityi* (as *P. m. verityi*) is protected in India [48]. The Taiwanese endemic subspecies *P. m. sylvina*, listed as ‘Critically Endangered’, has not been seen since the catastrophic earthquake of 1999 that destroyed its habitat and is presumed extinct [50,51].

In this study we analyzed the barcoding region of the mitochondrial cytochrome c oxidase subunit 1 (COI) gene for all species and subspecies of *P. machaon* species group from the entire Palearctic region recognized by modern treatments, including, for the first time, the endangered *P. m. britannicus*. In order to better understand the evolution of this species group, we complemented our COI dataset with additional nuclear gene sequences (Ca-ATPase, 28S rDNA, and CAD) for selected specimens attributed to currently recognized taxa. In addition, we used machine learning (ML) to quantify and visualize inter-species diversity in visible phenotype (disparity) within the *P. machaon* species group. We aimed to: (1) Infer the phylogenetic relationships between *P. machaon* and its closest relatives with a focus on the Palearctic region, (2) Investigate the concordance of morphological and morphometric data with results of the genetic analyses in the *machaon* group, and (3) Investigate if the current taxonomic entities correspond with these patterns.

## Materials and methods

### Molecular taxon sampling

**Fig 1:**
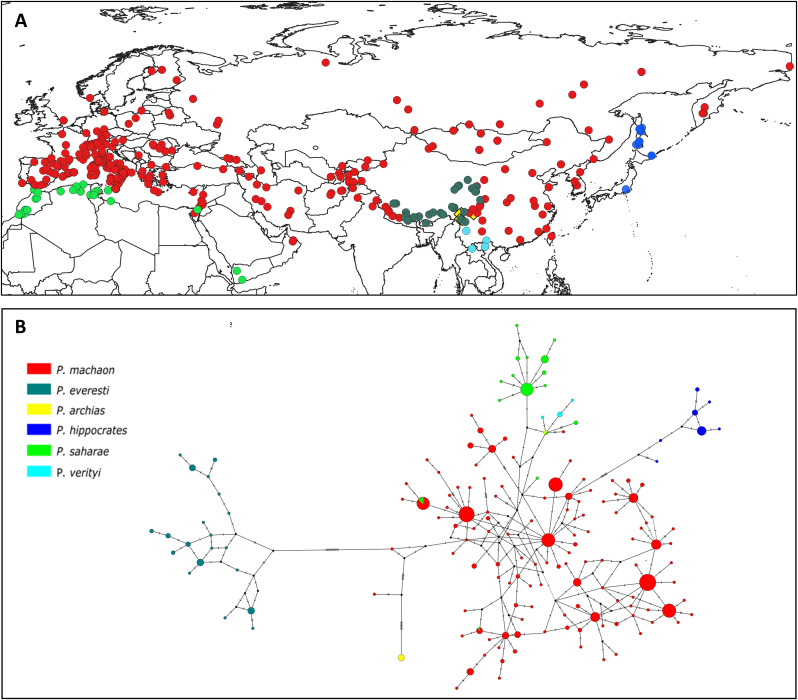
(A) Sampling localities for the populations included in this study are shown with different coloured circles. The map was prepared using Quantum GIS 2.8.2 (https://qgis.org/downloads/) based on a map from Natural Earth (www.naturalearthdata.com). (B) Median-Joining Network of *P. machaon* COI sequences. The size of circles is proportional to haplotype frequency and numbers of mutations between haplotypes are shown at the connections.

We analyzed 585 individuals representing 13 currently considered species in the *P. machaon* species group and 22 recognized subspecies of *P. machaon* [34,36] from sites across the Holarctic region (Fig 1A). Museum specimens were sampled from several private and public collections. Of the total entries in our dataset, 309 samples were barcoded for the first time through this study, and the remaining 275 sequences were retrieved from GenBank and BOLD (S1 Table). We selected *P. zelicaon, P. polyxenes, P. indra* and *P. xuthus* as outgroups and rooted the tree with the more distant *P. maackii* or *P. ulysses*. The relevant sequences for the latter were also retrieved from GenBank.

### DNA extraction, PCR Amplification and Sequencing

Legs removed from samples were used for extraction of total genomic DNA in three different laboratories, the Canadian Centre for DNA Barcoding (CCDB) (University of Guelph, Guelph, Canada), the Ecology and Genetics Research Unit (EGRU, University of Oulu, Oulu, Finland), and the Zoological Institute of the Russian Academy of Science (ZISP) (St. Petersburg, Russia). In the CCDB lab, the extraction was carried out on Biomek FX liquid handling robot using a semi-automated DNA extraction protocol [52] on glass fiber plates (PALL Acroprep 96 with 3 μm GF membrane over 0.2 μm Bioinert membrane). DNA was eluted in 35–40 μl of ddH20 pre-warmed to 56 °C and stored at − 80 °C. In the EGRU lab, total genomic DNA was extracted using the Qiagen columns of QIAamp DNA mini kit and DNeasy Blood & Tissue Kits (Qiagen, Hilden, Germany) and it was eluted in 20–50µl of Elution buffer. The eluted DNA obtained in the EGRU lab was sent to Macrogen (https://dna.macrogen.com/#) for sequencing. In the ZISP lab, for the samples more than 10 years old, the total genomic DNA was extracted using QIAamp DNA Investigator Kit (Qiagen, Venlo, The Netherlands), following the manufacturer’s protocol. For the specimens up to 10 years old, DNA extraction was performed using the CTAB-based method [53] with minor modifications (the time of digestion with proteinase K was increased to 24h [54,55]).

In CCDB, the mtDNA sequencing was carried out using standard protocols and LepF/LepR primers, supplemented by failure-tracking with mini-primers (mLepF/mLepR) [56].

In ZISP, a 658 bp fragment of the COI gene (mitochondrial DNA barcode) was amplified using LCO1490/HCO2198 [57] and LepF/LepR primer pairs [56]. In addition, three nuclear gene regions (CAD, Ca-ATPase, and 28S) were sequenced in ZISP for selected samples (S1 Table). Sequencing was carried out at the Research Resource Center for Molecular and Cell Technologies (Scientific Park, St. Petersburg State University, St. Petersburg, Russia) within the framework of state assignment No. 125022803066−3. In case standard lepidopteran primers failed to yield a sufficient product, we amplified full-length barcode and nuclear fragments using self-designed primer pairs (see S2 Table). The PCR amplifications and sequencing were performed according to the protocol described in [58,59].

All sequences were submitted to GenBank (see S1 Table) and BOLD system repository (dataset “DS-MACHAON”, accessible at dx.doi.org/10.5883/DS-MACHAON).

### *Wolbachia* screening

A total of 101 specimens of the *P. machaon* species group were screened for *Wolbachia* infection (see S1 Table) by amplifying three genes, commonly used as markers to detect the presence of the bacteria, namely *Wolbachia* surface protein (wsp), 16S ribosomal RNA, and Filamentation temperature-sensitive protein Z (ftsZ) (S1 Table). *Wolbachia*-specific primer pairs, wsp81F/wsp691R [60], W-Specf/W-Specr [61], and ftsZ-F/ftsZ-R [62] were used, amplifying ~550 bp fragment of the wsp gene, ~ 440 bp fragment of the 16S RNA gene and ~510 bp (actual fragment sizes depended on the individual *Wolbachia* strain), respectively. The PCR amplifications were performed according to the protocol described in [58,63].

### Dataset compilation

A combined dataset of four genes (COI, CAD, Ca-ATPase, and 28S) was assembled using MEGA 11.0.8 [64]. Alignment of sequences was carried out using MUSCLE modules implemented in ALIVIEW 1.28 [65] and double-checked visually. After final alignment, the dataset contained a total of 1941 base pairs (bps). The partitioned Nexus file was used to generate a Maximum Likelihood tree using the IQTREE web server (http://iqtree.cibiv.univie.ac.at) [66] setting an automatic selection of models. Bayesian analysis was allowed to run in MrBayes 3.2.7a [67] for 10 million generations, with the first 25% of the trees discarded as burnin. The resulting consensus tree was viewed in FigTree 1.4.4 [68]. Excluding short and incomplete sequences, a Median-Joining Network of full-length (658 bps) COI barcode sequences (473) of *Papilio* was constructed in NETWORK 10.2 [69]. Haplotype diversity was calculated using DnaSP 6.0 [70].

### Species delimitation

We followed the phylogenetic species concept to delimit molecular operative taxonomic units (MOTUs) as putative species using the COI alignment for the phylogenetic analysis, excluding outgroups and sequences with significant amounts of missing data (> 200 bp). To test the taxonomic status of recovered clades, we used recently developed method ASAP, Assemble Species by Automatic Partitioning [71], proposing partitions of species hypotheses using genetic distances calculated between DNA sequences. ASAP analysis was run through a web-based interface (https://bioinfo.mnhn.fr/abi/public/asap) using default parameters.

### Estimation of diversification time and biogeography

Estimation of diversification time and biogeography was based on a dataset including 49 selected specimens covering the range of the *P. machaon* group and most of the described taxa. For most of them, all four gene sequences (COI, CAD, Ca-ATPase and 28S) were available (S1 Table). Due to their hybrid origin and shared mitochondrial haplotypes with *P. machaon*, we excluded the taxa *P. brevicauda, P. joanae* and *P. kahli* in order to avoid any negative influence on the biogeographic reconstruction [34]. To infer a dated phylogeny, we used BEAST v.2.6.2 software [71] with an uncorrelated relaxed clock model and the tree prior set to birth–death. All other priors were set as the default. We used a fossil-based secondary calibration point of the most recent common ancestor (MRCA) of *Sinoprinceps* + *Papilio* sensu stricto (17.39 Mya) from the dated phylogeny of the genus *Papilio* by Condamine et al. [35]. This calibration point has been substantiated by subsequent studies [72]. The analyses were run for 50 million generations, sampled every 5000 generations and repeated three times. The parameters of all three runs were compared in Tracer v.1.5 [73], in which we also checked the model convergence (effective sample size *>* 200). Trees from all three runs were combined by LogCombiner v.1.8.4 [74], and 20% of trees were discarded as burn-in. The maximum credibility tree was selected using TreeAnnotator v.1.8.4 [74]. The final phylogenetic tree was rendered in FigTree v.1.4.0 [68].

We used a set of trees and the maximum credibility tree from the BEAST analysis for the statistical dispersal-vicariance analysis (S-DIVA) implemented in RASP v.4.0 [75]. Four distribution areas were selected: (A) W Palearctic, (B) E Palearctic, (C) Indomalayan realm and (D) Nearctic.

### Morphology

The systematic treatment of the Palearctic *Papilio machaon* group used in this study is largely adopted from Nazari et al. [36] where diagnostic morphological characters from wing pattern and genitalia are discussed in detail.

### Machine learning analyses of specimen photographs

**Fig 2:**
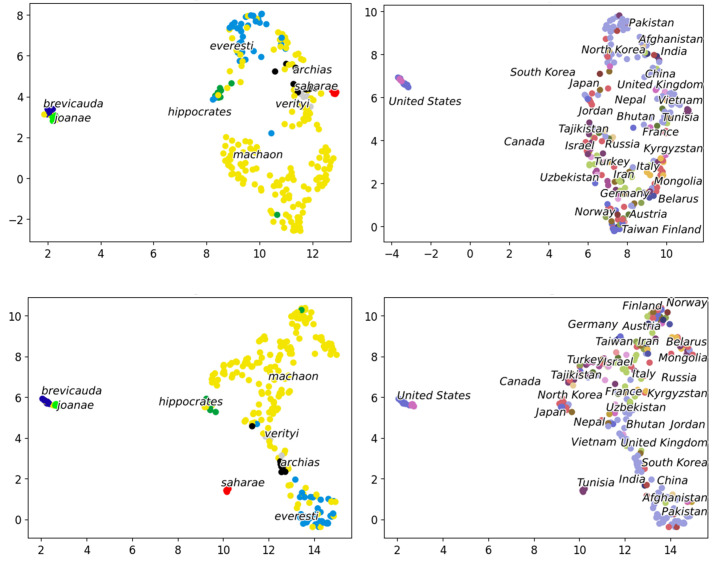
Machine-learnt embeddings of 245 photographs of butterflies from the *Papilio machaon* species group. Two-dimensional visualizations of a 64-dimensional embedding from a triplet-trained CNN in which Euclidean distance represents image similarity. Embeddings fine-tuned by training for 1000 (upper) or 2000 (lower) training epochs, using a pre-trained Papilionidae CNN [18]. Colors represent the corresponding labels (species or country).

In general, image-based machine learning methods have potential advantages for biological analysis, over previously available techniques. These include direct image analysis, without the need for manual landmark or homology identification, comparative robustness to feature translation [18], ability to incorporate information from across a specimen image, e.g., including within an outline [76], many to many feature comparisons [18], and potential to quantitatively analyse both qualitatively noticeable and subtle variation [18,76]. In the machine-learning (ML) analyses conducted here, a triplet-trained convolutional neural network (CNN) was trained on 245 dorsal photographs of the swallowtail butterfly species under study (using a standard, 80/20 train/test split, and training labels corresponding to the hypothetical species in S1 Table). The photographs used in the ML analyses are available in S3 Fig. This method used a previously published convolutional neural network (CNN) architecture (ButterflyNet version 1.2, code available as Supplementary Software 1 of reference [18]). ButterflyNet version 1.2 performs image embedding by optimising triplet-loss (results, S4 Fig), such that for sampled triplets of images (two of the same label taxon, one of a different label taxon) the Euclidean distance is minimised between images of the same label (iterating on ButterflyNet version 1, which used optimization based jointly on embedding and classification). This study used the following default parameters of ButterflyNet version 1.2: Adam optimizer, learning rate of 0.0001, embedding dimension 64, random affine image augmentation [18]. Batch size was reduced to 10 (from 100 [18]). Hypothetical species under study for which photographs were available for the machine learning analyses were those with machine learning labels: *archias*, *brevicauda*, *everesti, hippocrates*, *joanae*, *machaon*, *saharae*, and *verityi*. Machine learning analyses were conducted comparing two sample balancing protocols [18] to account for variable image sample sizes among the label classes. Balancing sampling ensures label taxa are sampled uniformly for the triplet images from the same taxon (Fig 2) and additionally from the different taxon (S4-S5 Fig). Prior to this fine-tuning on the swallowtail image dataset, the network was pre-trained [18] on ~17,000 photographs of birdwing butterflies (also included in family Papilionidae). Fine-tuning is a machine learning training method that uses pre-training on one dataset (here a larger one that is also more taxonomically disparate, in covering three genera) aiming to improve performance on a second dataset (here a smaller, more taxonomically focused dataset, in a comparatively closely related, but phenotypically distinct butterfly genus). Additional ML parameters were as documented in reference [18]. The output of the embedding analysis is a matrix of embedded Euclidean distances between photographs in which proximity represents image similarity. Two-dimensional visualisations of the resultant embedding distances were produced using the UMAP algorithm (‘Uniform Manifold Approximation and Projection for Dimension Reduction’) [77]. The Pearson correlation was quantified between average distances in the machine learnt embedding and in the genetic phylogeny across six taxa that were comparable due to representation in both photographic and genetic datasets (with embedding labels *archias*, *everesti*, *hippocrates*, *machaon*, *saharae*, and *verityi*).

## Results

### Sequencing results

Of the 309 samples barcoded in this study, 253 were full-length (658 bp) and only five were shorter than 500 bp. Nuclear genes (CAD: 496 bp, 28S: 341 bp, Ca-ATPase: 446 bp) were successfully obtained for nearly all 49 selected samples. See S1 Table for material examined and GenBank accessions.

### *Wolbachia* screening

Screening of 101 samples of *Papilio machaon* species group for three *Wolbachia* genes (16S, wsp and ftsZ) did not reveal any cases of dissimilar results (i.e., when a specimen was positive for one *Wolbachia* gene, but negative for another gene/genes). In total, all tested specimens but two were scored negative for *Wolbachia* infection. We found *Wolbachia* infection only in two females (out of 2 females tested) of *P. archias* (PAP38, PAP41), while three analyzed males of this taxon were not infected. Thus, our analysis suggested that *Wolbachia* infection of *P. archias* is sex-dependent; however, further research based on additional material is needed to confirm this assumption.

Sequencing of *Wolbachia* 16S, wsp and ftsZ genes of two *Wolbachia*-positive females of *P. archias* showed that these specimens were infected by a *Wolbachia* strain belonging to the supergroup B (grouping according to [78]). Infected *P. archias* specimens share the 16S *Wolbachia* sequence with various insect taxa, belonging to orders Diptera (families Tephritidae, Culicidae, Syrphidae), Hemiptera (Cixiidae, Lophopidae, Derbidae, Kerriidae), Hymenoptera (Trichogrammatidae), Orthoptera (Gryllidae) Lepidoptera (Noctuidae, Geometridae, Tischeriidae) and spider mites (Acari: Tetranychidae). This is the first time that this particular *Wolbachia* allele has been found in any member of the superfamily Papilionoidea (see S6 Fig).

### Geographic distribution of mtDNA haplotypes

The mtDNA haplotype network of 162 haplotypes (Haplotype diversity 0,9764) highlighted four main haplogroups attributable to *P. machaon, P. everesti, P. hippocrates* and *P. archias*. In the latter case, one population (PAP074 from Sichuan) shared its haplotype with *P. saharae rathjensi* from Yemen, while the other sequences (5 out of 6 from Yunnan, Sichuan and Tibet) shared a peculiar haplotype (Fig 1B). The North African taxa *saharae* and *neosaharae* Tarrier, 2016 shared haplotypes with each other and with *mauretanica* Verity, 1905 which have all been shown to be conspecific [79]. In addition, *P. verityi* haplotypes appeared within the *P. machaon* haplogroup (Fig 1B).

### Species delimitation analysis

Application of the Assemble Species by Automatic Partitioning (ASAP) delimitation method, based on the genetic pairwise distances of COI, revealed that in our case, this method tends towards lumping (S7 Fig). The number of MOTUs recovered in the best partitioning schemes is lower than the number of species based on the integrative analysis. The partitioning scheme with the best ASAP score (3.0, species threshold 2.2%) counts only three MOTUs corresponding to *P. machaon*, *P*. *hospiton* and *P*. *everesti*. The second-best scheme (ASAP score 4.0, species threshold 2.27%) counts only two MOTUs corresponding to *P. machaon* and *P*. *everesti*, and the third (ASAP score 4.5, species threshold 1.46%) accounts for five MOTUs corresponding to *P. machaon*, *P*. *hospiton*, *P*. *everesti*, *P*. *archias* and *P. machaon aliaska*. It is noteworthy that none of these schemes include *P*. *hippocrates* as a distinct species.

### Estimation of phylogenetic relationships

In our phylogenetic analyses, the tree topologies inferred using ML (IQTree) and Bayesian (Mr. Bayes, BEAST) methods were nearly identical (S8–S10 Fig). In all these inferences, a strongly supported monophyletic *everesti* appeared as sister to the *archias+*(*hippocrates+*(*brevicauda*+*machaon*)) group. Within the Palearctic *machaon*, only a few populations stood out: A clade containing samples from the Himalayas (Northern Pakistan, India, Tibet, Nepal) and two specimens from Uzbekistan and Tajikistan; and another clade that included all of our north African samples, inclusive of taxa *mauretanica*, *saharae* and *neosaharae* without distinction. Instead, the taxon *rathjensi* from Yemen seemed completely unrelated to the North African *saharae* and appeared in an odd clade together with the taxa *verity, archias* (one sample from Sichuan), *machaon* from Sicily, and a few others Canadian samples. In a similar way, a few other smaller clades showed moderate to good support, however no geographic structure could be discerned.

### Estimation of diversification time and biogeography

**Fig 3:**
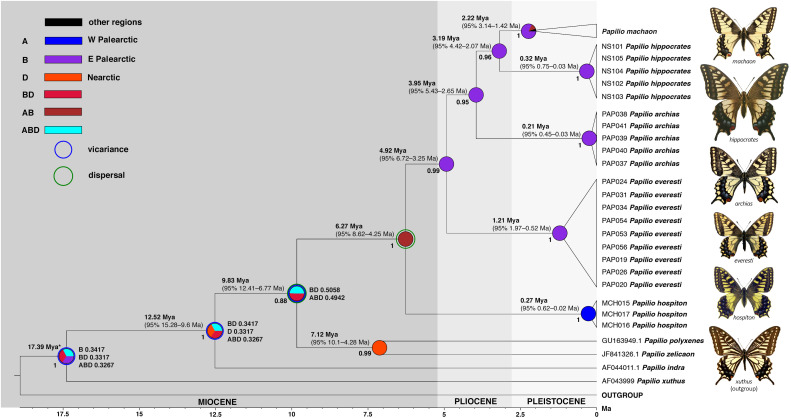
Historical biogeography of the *Papilio machaon* species group. Summary of BEAST and RASP analyses, ancestral area reconstruction model S-DIVA. Region C (Indomalayan realm) is not shown as specimens originated there are included in the collapsed *P. machaon* clade. Pie charts on each node depict the relative probabilities of ancestral ranges. Numbers below nodes indicate Bayesian posterior probability (see S11 Fig for the expanded tree).

Our dated phylogenetic analysis in BEAST (Fig 3) suggested that the *P. machaon* species-group originated in the Middle Miocene, ~ 12.52 Mya. The most recent common ancestor (MRCA) of the Old World *P. machaon* species group and Nearctic *P. polyxenes* + *P. zelicaon* diverged in the mid-late Miocene, ~ 9.83 Mya. Biogeographic estimation in RASP suggested nearly equal probability of the place of its origin, either in the Eastern Palearctic + Nearctic (region BD) or in the Holarctic (region ABD). The first split in the MRCA of the Old World *P. machaon* species group occurred in late Miocene, ~ 6.27 Mya, which gave rise to the ancestor of *P. hospiton* and the MRCA of the remaining Palearctic species. Based on the placement of two Tibetan species (*P. everesti* and *P. archias*) on the tree, we suggest that further diversification occurred in the region of the Tibetan Plateau, where two successive speciation events in the Pliocene, ~ 4.92 Mya and ~3.95 Mya, gave rise to the ancestors of *P. everesti* and *P. archias*, respectively. Finally, the MRCA of *P. machaon* and *P. hippocrates* branched off in the late Pliocene, ~ 3.19 Mya in East Asia. Further diversification of all species of the group occurred during the Pleistocene. *Papilio machaon* began to diversify about 2.22 Mya and dispersed westward to the Western Palearctic and eastward to the Nearctic via Beringia.

### Morphology

The result of the machine learning (ML) analyses is an “embedding” that captures the visual similarity between butterfly photographs in a multidimensional space in which images found to be more visually similar are relatively close together (using ButterflyNet 1.2 [18] to embed, based on triplet-loss, 245 dorsal photographs of swallowtails species under study by fine-tuning a network pre-trained [18] on ~17,000 photographs of birdwing butterflies). The multidimensional space of the resultant embedding is visualized by projection to two dimensions (Fig 2), using the python UMAP package [77] (a widely-used tool for the visualization of high-dimensional data). Plots resulting from the machine-learnt embedding of our swallowtail butterfly images illustrate how the trained network clusters the photographs by training label (corresponding to the operational taxonomic units representing hypothetical species; see S1 Table). The correlation between average distances from genetic phylogeny (Fig 3) and photographs in the machine learnt embedding was 0.76 (S4 Fig: supplementary ML analyses of sample-size balancing, genetic correlation and triplet loss plots). Embedding plots also revealed suggestions of visible sub-structure, e.g., within *P. machaon* where the sample size was the largest. In particular, some clustering by country, and inter-country geographic distance, was apparent within *P. machaon* (Fig 2). Overall, there was a weak but statistically significant positive correlation between geographic distance and image embedding distance p < 0.001, r = 0.344 (3 d. p.), in line with a tendency toward greater learnt image similarity between specimens sampled from geographically closer locations (S11 Fig).

## Discussion

### Evolution of *Papilio machaon* in the Palearctic/Oriental regions

The subgenus *Papilio* sensu stricto originated in the Middle Miocene in a region common for the Eastern Palearctic and the Nearctic, likely in Beringia, as was suggested by Condamine et al. [35]. This event was then followed by further westward dispersal of the MRCA of the Old World *P. machaon* clade to Asia in the late Miocene. During the Middle and the Late Miocene, Beringia was characterized by rather high annual temperatures allowing migrations of warm-adapted butterflies from the Old World to the New World [18]. It should be noted that our inferred dates were slightly older than the previous estimations by Condamine et al. [35]; in addition, our BEAST phylogeny did not support the sister-group relationship between *P. polyxenes* + *P. zelicaon* and the Old World species.

In Eurasia, the main diversification event occurred in the Late Miocene. It gave rise to western and eastern lineages, the MRCA of *P. hospiton* and the MRCA of the rest of the species of the *P. machaon* clade, respectively. This period is characterized by drastic changes in terrestrial environments across the modern Holarctic region caused by global cooling [80,81], which finally gave rise to modern ecosystems; moreover, this time corresponds to the rapid diversification of the main host plants of the *P. machaon* clade, members of the subfamily Apioideae (Apiaceae) [82].

Our results suggest that the initially warm-adapted ancestor of *P. hospiton* previously had wider distribution in the West Palearctic, but its range dramatically decreased in the Pleistocene likely due to glaciations. Nowadays the range of this species is restricted to Corsica and Sardinia, which are known as glacial refugia for various plants and animals [83,84]. Based on our reconstruction, it is likely that the ancestor of *P. hospiton* colonized Corsica and Sardinia in the late Miocene during the Messinian Salinity Crisis, between 5.97 and 5.33 Ma ago.

Members of the eastern lineage initially diversified from the early to the middle Pliocene in mountains of the Tibetan Plateau, which took their definite shape by this time [85], and gave rise to *P. machaon* and *P. hippocrates* in the Late Pliocene in East Asia. *Papilio machaon* colonized the entire Palearctic, large part of the Nearctic, and, partially, the Oriental region during the Pleistocene, while *P. hippocrates* colonized Sakhalin Island and the Japanese Archipelago, likely via land bridges between Sakhalin, Kuril Islands and Hokkaido formed during glacial periods [86].

The deep divergence and the absence of shared haplotypes between *P. hospiton* and *P. machaon* likely suggest a relatively recent arrival of the latter in the West Palearctic and, specifically, in the West Mediterranean, even if the North African populations seem to indicate an earlier colonization. Our COI barcode phylogenies are in line with the conclusions by Cassar et al. [79] based on genomic data that all populations from North Africa (Morocco, Tunisia, Algeria) and Italy (Lampedusa) are best to be regarded as a monophyletic unit separate from the continental *P. machaon*; however, barcode data alone does not support a separate species status for *P. saharae*, as the North African clade appears nested within the Eurasian *P. machaon* (S8–S10 Fig). We did not find any *P. saharae* haplotypes among our examined material identified as such from Israel. In addition we suggest that, pending additional morphological and genomic data, the Arabian endemic taxon *rathjensi* should be raised as a separate species. Beside a radically different morphology, the mitochondrial haplotypes of the samples of *rathjensi* examined in our study from Yemen (PQ885012 and PQ885039) appear completely unrelated to *P. saharae* and closer to the Asian taxa *P. verityi* and *P. archias* (S8–S10 Fig).

### Utility of Machine-Learning in butterfly taxonomy

In general, ML methods – including convolutional neural networks (CNNs) – benefit from large sample sizes. Sample sizes in computer-science contexts (e.g., up to millions of images, or more) have often been far larger than traditional biological datasets. However, some recent biological applications of ML have also found that CNNs can be effective when applied with modest biological image dataset sizes (e.g., [76], image pairs drawn from images of 46 wolf skull specimens). The utility of ML methods has also been demonstrated in studies on Papilionidae [18]. In this study, ML clustering based on photographic image similarity (Fig 2) is supportive of the haplotype and geographic analyses, with a large cluster of images of *P. machaon* (the most-sampled species in the image dataset), around the periphery of which lie small image clusters sampled from the other species in the group. A measured, relatively strong, correlation (S4 Fig) provides quantitative evidence that average patterns of similarity in morphology evident in specimen photographs (Fig 2, S5 Fig.) are positively correlated with the observed phylogenetic distances among taxa (Fig 3). Indications of sub-structure associated with geographic location are also visible within the phenotypic image embedding, supported by a positive correlation between learnt image similarity and geographic distance. This analysis suggests that collecting large samples of images for ML analysis in future work on *Papilio* populations of conservation concern (e.g., from the United Kingdom) has the potential to provide further information on their phenotypic distinctiveness.

## Supporting information

S1 Table Material examined and GenBank accessions. (PDF)

S2 Table Primers combinations used in this study for COI gene fragment amplification. (PDF)

S3 Fig Images used in the machine learning analysis. (ZIP)

S4 Fig Supplementary machine learning analysis testing correlation between machine learnt embedding and genetic distances (corresponding to the phylogeny in Fig 3). Machine learning trained for 2000 epochs, incorporating an additional triplet balancing step to account for imbalanced sample sizes among label taxa. Final correlation coefficient at 2000 epochs: r = 0.76 (2 d. p.).
(PDF)

S5 Fig Visualisation of the 245 swallowtail butterfly photographs used as input for the machine learning analysis (upper) at their locations in a 2D UMAP projection of the embedding (lower) corresponding to S4 Fig. (PDF)

S6 Fig Phylogenetic trees showing placement of the *Wolbachia* strain retrieved from two female samples of *Papilio archias* among closely related 16S, wsp, ftsZ Wolbachia sequences mined from GenBank. (PDF)

S7 Fig Results of the ASAP analysis. (PDF)

S8 Fig IQTREE. (PDF)

S9 Fig Mr. Bayes tree. (PDF)

S10 Fig Tree from BEAST analysis. (PDF)

S11 Fig Comparisons of latitude, longitude and machine learnt image distances. Latitude and longitude of swallowtail butterfly specimens included in the machine learning image analyses, with points coloured by country. Pairwise comparison of Euclidean distances machine learnt from 245 specimen photographs and the geographic latitude or longitude associated with that specimen. Geographic distance is the geodesic distance calculated using the scipy and geopy packages. Machine learning results correspond to those of SI S7. Pearson correlation p < 0.001, r = 0.344 (3 d. p.).
(PDF)
