# Supplementary material for: Integrative analyses reveal the evolution of the Old World Swallowtail in the Palearctic
Source: PLoS One. 2026 Jul 8;21(7):e0343793. doi: 10.1371/journal.pone.0343793 (PMC13345299; doi:10.1371/journal.pone.0343793)
Supplement: S1 Table — (PDF) [file pone.0343793.s001.pdf]

S1 Table. Material examined and GenBank accessions.

0	Spcies	Sample ID	Photos	COI Accession	lengh	Ca-ATPase	CAD	28S	Phylogeny	*16S*	*wsp*	*ftsZ*	Collectors	Collection Date		Lat	Lon	Elev
1	archias	PAP037	1	PQ884961	658 bp	PQ885107	PQ885130	PQ885073	x	negative	negative	negative		06-May-2019	China: Sichuan, Batang	30	99.1	1500
2	archias	PAP038	1	PQ884967	658 bp	PQ885108	PQ885131	PQ885074	x	positive	positive	positive		06-May-2019	China: Sichuan, Batang	30	99.1	
3	archias	PAP039	1	PQ884727	658 bp	PQ885083	PQ885118	PQ885050	x	negative	negative	negative		06-May-2019	China: Tibet, Mangkang county, Chamdo	29.7	98.6	3200
4	archias	PAP040	1	PQ884983	658 bp	PQ885111	PQ885134	PQ885077	x	positive	positive	positive		06-May-2019	China: Yunnan, Deqin county, Meilixueshan mountain	28.5	98.7	2000
5	archias	PAP041	1	PQ884840	658 bp	-	PQ885121	PQ885056	x	negative	negative	negative		07-Jun-2019	China: Yunnan, Xiangerila area, W of Benzilan town	28.2	99.3	2700
6	archias	PAP074	1	PP865970	658 bp	-	-	-		negative	negative	negative		15-Jun-2020	China: Sichuan, Ganzi, Mt. Gonggashan	29.6	102.1	
7	brevicauda	09BBELE-2648	1	HM416180	658 bp	-	-	-		-	-	-	BIObus 2009	19-Jul-2009	Canada: Newfoundland and Labrador, Gros Morne NP, Stuckl	49.427	-57.739	56
8	brevicauda	CB0070	2	PQ884993	658 bp	-	-	-		-	-	-	Carle Belanger	19-Jun-2005	Canada: Quebec, Longue-Pte-de-Mingan	50.258	-64.158	
9	brevicauda	CB0071	2	PQ884897	636 bp	-	-	-		-	-	-	Carle Belanger	19-Jun-2005	Canada: Quebec, Longue-Pte-de-Mingan	50.258	-64.158	
10	brevicauda	CCDB-24271-E01	1	PQ885005	658 bp	-	-	-		-	-	-		17-Jun-2001	Canada: Newfoundland and Labrador, Deer Lake	49.1697	-57.4323	
11	brevicauda	CCDB-24274-C07	2	PQ884956	658 bp	-	-	-		-	-	-	J.Troubridge, I	03-Jul-1983	Canada: New Brunswick, Beach Southeast of Tabusintac	47.32	-65	
12	brevicauda	CCDB-24274-C08	1	PQ884830	616 bp	-	-	-		-	-	-	J.Troubridge, I	03-Jul-1983	Canada: New Brunswick, Beach Southeast of Tabusintac	47.32	-65	
13	brevicauda	CNCLEP00083867	0	PQ884755	658 bp	-	-	-		-	-	-	P.D.Tonnanco	08-May-2012	Canada: Quebec, Pointe-des-Monts, Municipalite Regionale de	49.32	-67.3865	
14	brevicauda	CNCLEP00083868	0	PQ884836	658 bp	-	-	-		-	-	-	P.D.Tonnanco	08-May-2012	Canada: Quebec, Pointe-des-Monts, Municipalite Regionale de	49.32	-67.3865	
15	brevicauda	DH008596	1	PQ884728	658 bp	-	-	-		-	-	-	D. Handfield	07-Jul-2004	Canada: Quebec, Sept iles	50.213296	-66.375793	
16	brevicauda	DH010967	1	PQ884942	658 bp	-	-	-		-	-	-	D.Handfield	21-May-2005	Canada: Quebec, Havre-aux-maisons, Iles-de-la-Madeleine	47.46	-61.76	
17	brevicauda	DH010968	1	PQ884723	658 bp	-	-	-		-	-	-	D.Handfield	23-May-2005	Canada: Quebec, Havre-aux-maisons, Iles-de-la-Madeleine	47.46	-61.76	
18	brevicauda	EZ0012CNC	1	PQ884733	658 bp	-	-	-		-	-	-	J. & L. Troubri	04-Jul-1988	Canada: Newfoundland and Labrador,, Doyles	47.8303	-59.1913	
19	brevicauda	FS319	0	KJ363193	2,288 bp	-	-	-		-	-	-			Canada: Newfoundland and Labrador,, Fishell	48.316792	-58.710212	
20	everesti	AC-SP1638	2	PQ884975	633 bp	-	-	-		-	-	-		06-Jun-2009	China: Tibet, NE Tibet, Kaqila	31.3	97.35	4300
21	everesti	AC-SP1798	2	PQ885017	658 bp	-	-	-		-	-	-		25-Jun-2014	China: Tibet, SE Tibet, Demu-La	29.6	94.6	4875
22	everesti	DNAcdb0066	1	PQ884938	627 bp	-	-	-		-	-	-	Local collector	23-May-2014	China: Tibet, Nylam county, S of Shigatse (Xigaze)' LalungLa	28.501	86.101	5000
23	everesti	DNAcdb0068	1	PP865966	658 bp	-	-	-		-	-	-	L. Bieber	03-Jun-2007	China: Tibet, Nyalam	28.155	85.982	4200
24	everesti	DNAcdb0084	1	PQ885027	627 bp	-	-	-		-	-	-	Della Bruna	17-Jul-2014	China: Qinghai, Laji Shan, Huangzhong vic.	36.384	102.561	3100
25	everesti	DNAcdb0085	1	PQ884912	627 bp	-	-	-		-	-	-		07-Aug-2017	China: Qinghai, Dulan county, the montain near Xiariha town	36.466	98.198	4000
26	everesti	DNAcdb0086	1	PQ884722	645 bp	-	-	-		-	-	-	J. Klir	05-Jun-2007	China: Yunnan, De-Quin 10km S	28.433	98.917	2900
27	everesti	DNAcdb0089	1	PQ884872	627 bp	-	-	-		-	-	-	P. Castellani	10-Jul-2012	China: Sichuan, Min Shan, Provincial road 301 (km 104), 9km	33.168	103.705	3000
28	everesti	DNAcdb0093	1	PQ884996	627 bp	-	-	-		-	-	-	PC & CDB	04-Jul-2019	China: Gansu, Gannan prefecture, Xiahe vicinity	35.226	102.659	3000
29	everesti	GCB03	2	PQ884802	658 bp	-	-	-		-	-	-	Karma Wahgdi	01-Jan-2005	Bhutan	27.514	90.434	
30	everesti	not given	0	MK602655	15,339 bp	-	-	-		-	-	-		15-Jul-16	China: Qinhai, Qilianshan Mountains	38.083333	100.65	
31	everesti	PAP016	1	PQ884742	658 bp	-	-	-		-	-	-	L. Bieber	13-Jun-1996	China: Tibet, West Gangdize Mts., 40 km East of Saga	29.3	85.6	5150
32	everesti	PAP017	1	PQ885037	658 bp	-	-	-		-	-	-	L. Bieber	18-Jun-1996	China: Tibet, West Nganglong Mts., 100 km West of Gerze	32.3	83.1	4300
33	everesti	PAP018	1	PQ884972	658 bp	-	-	-		negative	negative	negative	L. Bieber	23-Jun-2005	China: Tibet, Tsochen pass, Tsochen pass	30.1	85.5	5200
34	everesti	PAP019	1	PQ885019	658 bp	PQ885115	-	-	x	negative	negative	negative	J. Pavol	18-Jun-2011	China: Tibet, 60 km S of Saga	28.8	85.3	5000
35	everesti	PAP020	1	PQ884777	658 bp	PQ885092	-	-	x	negative	negative	negative	L. Bieber	15-Jun-2005	China: Tibet, Nyalam	28.1	86	4200
36	everesti	PAP021	1	PQ884737	658 bp	-	-	-		-	-	-	B. Malacek	12-Jun-1999	China: Tibet, N. Nyingri prefecture, Basum Tso Lake	30	94	4050
37	everesti	PAP022	1	PQ884808	658 bp	-	-	-		-	-	-	B. Malacek	06-Jun-1999	China: Tibet, Lhasa	29.6	91.2	3700
38	everesti	PAP023	1	PQ884756	658 bp	-	-	-		negative	negative	negative		08-Jul-2018	China: Tibet, Nanda County, Chamdo, Nandaxiang	31.5	96.8	3800
39	everesti	PAP024	1	PQ884828	658 bp	-	-	PQ885055	x	negative	negative	negative		07-Jun-2018	China: Tibet, Linzhi area, Demula Pass	29.6	94.6	5000
40	everesti	PAP025	1	PQ884784	658 bp	-	-	-		negative	negative	negative		15-Jul-2018	China: Tibet, Yadong county, Xigaze area	29.1	88.7	5000
41	everesti	PAP026	1	PQ884774	658 bp	PQ885091	-	-	x	negative	negative	negative		15-Jul-2018	China: Tibet, Yadong county, Xigaze area	29.13	88.7	5000
42	everesti	PAP027	1	PQ884916	658 bp	-	-	-		negative	negative	negative		15-Jul-2018	China: Tibet, Yadong county, Xigaze area	29.1	88.7	5000
43	everesti	PAP028	1	PQ884726	658 bp	-	-	-		negative	negative	negative		15-Jul-2018	China: Tibet, Yadong county, Xigaze area	29.1	88.7	5000
44	everesti	PAP029	1	PQ884790	658 bp	-	-	-		negative	negative	negative	Beranek	13-Jul-2005	China: Qinghai, Wulan	36.7	98.5	4000
45	everesti	PAP031	1	PQ884937	658 bp	PQ885103	-	PQ885068	x	negative	negative	negative		15-Jun-2014	China: Qinghai, Guide county, Qunjia Pass	36	101.7	3000
46	everesti	PAP032	1	PQ884987	658 bp	-	-	-		-	-	-	L. Bieber	08-Jun-1998	China: Tibet, Xiahe, Labrang	35.2	102.5	3500
47	everesti	PAP033	1	PQ884799	658 bp	-	-	-		-	-	-	L. Bieber	08-Jun-1998	China: Tibet, Xiahe, Labrang	35.2	102.5	3500
48	everesti	PAP034	1	PQ884751	658 bp	PQ885087	-	-	x	negative	negative	negative		15-Jul-2018	China: Qinghai, Dulan county, Xiariha	36.5	98.2	4000
49	everesti	PAP035	1	PQ884741	658 bp	-	-	-		negative	negative	negative		07-Jun-2018	China: Tibet, N of Chamdo city	31.2	97.2	4000
50	everesti	PAP036	1	PQ885004	658 bp	-	-	-		negative	negative	negative		07-Jun-2018	China: Tibet, N of Chamdo city	31.2	97.2	4000
51	everesti	PAP053	1	PQ885013	658 bp	PQ885114	PQ885137	PQ885080	x	negative	negative	negative	A. Krupitsky	10-Jul-2019	China: Sichuan, 50 km NNW Litang, Heni vill.	30.24	99.82	4250
52	everesti	PAP054b	1	PQ884925	658 bp	PQ885101	PQ885125	PQ885066	x	negative	negative	negative	A. Krupitsky	03-Jul-2017	China: Yunnan, Shangri La	27.796	100.065	
53	everesti	PAP056	1	PQ885043	658 bp	PQ885117	PQ885139	PQ885082	x	negative	negative	negative		06-Jul-2020	China: Qinghai, Dulan county, Xiariha	36.5	98.2	3250
54	everesti	PAP057	1	PQ884740	658 bp	-	-	-		negative	negative	negative		06-Jul-2020	China: Qinghai, Dulan county, Xiariha	36.5	98.2	3250
55	everesti	PAP063	1	PQ885042	658 bp	-	-	-		negative	negative	negative		15-Jun-2021	China: Tibet, Maizhokunggar County, Drigung Til	30.1	92.2	4200
56	everesti	PAP077	1	PQ884918	658 bp	-	-	-		-	-	-	L. Bieber	27-May-1996	China: Tibet, Gyatso La Pass	28.95	87.44	5300
57	everesti	PAP082	1	PQ884964	658 bp	-	-	-		-	-	-	L. Bieber	21-Jun-1996	China: Tibet, West Nganglong Mts, 20 km S of Yanhu	32.3	82.5	5150
58	everesti	PAP083	1	PQ884800	658 bp	-	-	-		-	-	-	L. Bieber	27-May-1996	China: Tibet, Gyatso La Pass	28.95	87.44	5300
59	everesti	PAP084	1	PQ884884	658 bp	-	-	-		-	-	-	L. Bieber	06-Jun-1997	China: Tibet, Nyalam	28.2	86	4200
60	everesti	PAP085	1	PQ884902	658 bp	-	-	-		-	-	-	L. Bieber	21-Jun-1996	China: Tibet, West Nganglong Mts, 20 km S of Yanhu	32.3	82.5	5150
61	everesti	PAP088	1	PQ884827	658 bp	-	-	-		negative	negative	negative		06-Jun-2021	China: Qinghai, Dulan county, Buerhan Buda	35.7	97.4	4250

0	Spcies	Sample ID	Photos	COI Accession	lengh	Ca-ATPase	CAD	28S	Phylogeny	*16S*	*wsp*	*ftsZ*	Collectors	Collection Date		Lat	Lon	Elev
62	hippocrates	AC-SP1632	2	PQ884893	614 bp	-	-	-		-	-	-		12-Jun-2009	Russia: Sakhalinskaya Oblast, C. Sakhalin, Tumovsk Dist., Longari	50.529	142.738	
63	hippocrates	DNAcdb0048	1	PQ884731	589 bp	-	-	-		-	-	-		07-Jul-2001	Russia: Sakhalinskaya Oblast, East Sakhalin range, Khrebtoviy rive	50.86	143.332	300
64	hippocrates	DNAwth031	2	PP865976	658 bp	-	-	-		-	-	-	Goshko	30-Jun-2012	Russia: Sakhalinskaya Oblast, S. Sakhalin Isl., The Cape Kril`on' 10	46.649	142.454	50
65	hippocrates	not given	0	GU372546	1,099 bp	-	-	-		-	-	-			South Korea: Jeju, Andeok-valley	33.258217	126.353328	
66	hippocrates	NS_101	1	PQ884953	658 bp	-	-	PQ885071	x	negative	negative	negative		05-Jun-2012	Russia: Sakhalinskaya Oblast, Sakhalin Island, Dolinsky District' Sc	47.5	142.7	
67	hippocrates	NS_102	1	PQ884803	658 bp	-	-	PQ885054	x	negative	negative	negative		05-Jun-2012	Russia: Sakhalinskaya Oblast, Sakhalin Island, Dolinsky District' Sc	47.5	142.7	
68	hippocrates	NS_103	1	PQ884865	658 bp	-	-	PQ885060	x	negative	negative	negative		05-Jun-2012	Russia: Sakhalinskaya Oblast, Sakhalin Island, Dolinsky District' Sc	47.5	142.7	
69	hippocrates	NS_104	1	PQ884724	658 bp	-	-	PQ885049	x	negative	negative	negative		05-Jun-2012	Russia: Sakhalinskaya Oblast, Sakhalin Island, Dolinsky District' Sc	47.5	142.7	
70	hippocrates	NS_105	1	PQ884949	658 bp	PQ885105	PQ885128	PQ885070	x	negative	negative	negative		07-Jul-2017	Russia: Sakhalinskaya Oblast, Sakhalin Island, Smirnykhovsky Dist	50	143.1	
71	hippocrates	NS_106	1	PQ884849	658 bp	-	-	-		negative	negative	negative		07-Jul-2017	Russia: Sakhalinskaya Oblast, Sakhalin Island, Smirnykhovsky Dist	50	143.1	
72	hippocrates	NS_107	1	PQ885038	658 bp	-	-	-		negative	negative	negative		07-Jul-2017	Russia: Sakhalinskaya Oblast, Sakhalin Island, Smirnykhovsky Dist	50	143.1	
73	hippocrates	NS_108	1	PQ884789	658 bp	-	-	-		negative	negative	negative		07-Jul-2017	Russia: Sakhalinskaya Oblast, Sakhalin Island, Smirnykhovsky Dist	50	143.1	
74	hippocrates	PAP092	1	PQ884858	658 bp	-	-	-		negative	negative	negative			Russia: Sakhalinskaya Oblast, Sakhalin Island, Nevelsky district' Yas	46.75	141.91	
75	hippocrates	PAP093	1	PQ884985	658 bp	-	-	-		negative	negative	negative	M. Jinkubo	11-Jul-2009	Japan: Saitama, Horinouchi	35.8	139.4	
76	hippocrates	PAP104 =AC-SP1737	2	PQ884758	658 bp	-	-	-		negative	negative	negative		31-Aug-2006	Japan: Nagasaki, Hashima, Hachijiri' Takehana	35.308	136.712	
77	hippocrates	PAP106 =AC-SP1633	2	PQ885006	658 bp	-	-	-		negative	negative	negative		12-Jun-2009	Russia: Sakhalinskaya Oblast, Sakhalin Island, Tumovsk District' Lc	50.529	142.738	
78	hippocrates	PAP108	3	PQ884761	658 bp	-	-	-		negative	negative	negative	B. Gavrilov	15-Jul-2023	Russia: Sakhalinskaya Oblast, Kunashir Island	44.0323	145.702	
79	hippocrates	PAP109	1	PQ884966	658 bp	-	-	-		negative	negative	negative	B. Gavrilov	15-Jul-2023	Russia: Sakhalinskaya Oblast, Kunashir Island	44.032337	145.70245	
80	hippocrates	PAP110	2	PQ885020	658 bp	-	-	-		negative	negative	negative	B. Gavrilov	15-Jul-2023	Russia: Sakhalinskaya Oblast, Kunashir Island	44.032337	145.70245	
81	hippocrates	Papilio_machaon_22	0	LC189130	1,029 bp	-	-	-		-	-	-			Japan: Aichi, Kasugai	36.767387	137.46935	
82	hippocrates	UASM9900078	0	AY457593	2,291 bp	-	-	-		-	-	-	J. Okura	1989	Japan: Gifu, vicinity of Gifu	35.423423	136.760622	
83	hospiton	EC-021	0	EF514438	596 bp	-	-	-		-	-	-			Italy: Sardinia, Nuoro, Gennargentu Mountains' Aritzo	39.955714	9.196834	1400
84	hospiton	EC-022	0	EF514443	596 bp	-	-	-		-	-	-			Italy: Sardinia, Nuoro, Gennargentu Mountains' Aritzo	39.955714	9.196834	1400
85	hospiton	MCH015	0	PQ884890	658 bp	PQ885098	-	PQ885063	x	negative	negative	negative		14-Jun-2012	Italy: Sardinia, Monti Gennargentu, Paulinu	40.006800	9.318900	
86	hospiton	MCH016	0	PQ884762	658 bp	PQ885088	-	PQ885052	x	negative	negative	negative		12-Jun-2012	Italy: Sardinia, Monti Gennargentu, Paulinu	40.006800	9.318900	
87	hospiton	MCH017	0	PQ884892	658 bp	PQ885099	-	PQ885064	x	negative	negative	negative		07-Jun-2012	Italy: Sardinia, Monti Gennargentu, Bruncu Spina	40.016667	9.300000	
88	hospiton	not given	0	AF044009	2,292 bp	-	-	-		-	-	-	R. Crnjar		Italy: Sardinia			
89	hospiton	RVcoll.13-T209	0	MN140747	658 bp	-	-	-		-	-	-			France: Corsica	46.64237	2.1940236	
90	hospiton	RVcoll.13-T218	0	MN141324	658 bp	-	-	-		-	-	-			France: Corsica	46.64237	2.1940236	
91	hospiton	RVcoll.13-T635	1	MN141128	658 bp	-	-	-		-	-	-			Italy: Sardinia, Monte Albo	42.98201	12.763658	
92	hospiton	RVcoll.13-T642	1	MN138550	658 bp	-	-	-		-	-	-			Italy: Sardinia, Lula	42.98201	12.763658	
93	hospiton	RVcoll.13-U519	1	MN142036	658 bp	-	-	-		-	-	-			Italy: Sardinia, Ogliastra, road SS389' between Villagrande and La	42.98201	12.763658	
94	hospiton	RVcoll.14-E400	0	MN143114	658 bp	-	-	-		-	-	-			France: Haute-Corse, Ponte Leccia	46.64237	2.1940236	
95	indra	not given		AF044011	2,291 bp	-	-	-	x	-	-	-			United States: Washington, Wawawai			
96	joanae	BIOUG36855-A10	1	PQ884957	658 bp	-	-	-		-	-	-	J.R.Heitzman	23-May-1975	United States: Missouri, Benton County, 3 miles Northwest of	38.2803	-93.4364	
97	joanae	BIOUG36855-B01	1	PQ885018	658 bp	-	-	-		-	-	-	J.R.Heitzman	27-May-1975	United States: Missouri, Benton County, 4 miles Northwest of	38.2803	-93.4364	
98	joanae	CCDB-30818-B10	1	PQ884785	635 bp	-	-	-		-	-	-	R.Grothe	07-Jun-1979	United States: Missouri, Benton County, Highway UU, Warsaw	38.2447	-93.3845	
99	joanae	CCDB-30818-B11	1	PQ885014	658 bp	-	-	-		-	-	-	R.Grothe	13-Jun-1979	United States: Missouri, Benton County, Highway UU, Warsaw	38.2447	-93.3845	
100	joanae	FS312	0	KJ363192	2,288 bp	-	-	-		-	-	-			United States: Missouri	38.24686	-93.40773	
101	machaon	0216JSB-071	0	PQ884730	510 bp	-	-	-		-	-	-	K.W. Philip	11-Jul-1994	United States: Alaska, interior, mi 85 Steese Hwy' Eagle Summit	65.48	-145.4	
102	machaon	11-H368	1	MH419249	658 bp	-	-	-		-	-	-	L. Dapporto	14-Aug-2011	Italy: Tuscany, Reggello	43.68	11.55	
103	machaon	11-Y095	1	MH420229	658 bp	-	-	-		-	-	-	Dapporto, Lec	29-Jun-2011	Italy: Lago Arancio	37.62	13.05	324
104	machaon	12-O366	1	MH418918	658 bp	-	-	-		-	-	-	Insitut de Biol	20-Jun-2012	Italy: Sardinia, Sassari, Perfugas	40.817	8.934	
105	machaon	12-R119	1	MH419948	658 bp	-	-	-		-	-	-	Dapporto, Lec	03-Aug-2012	Italy: Madonie, Pomieri	37.84	14.06	1290
106	machaon	12-R161	1	MN143768	658 bp	-	-	-		-	-	-	Leonardo Dap	04-Aug-2012	Italy: Sicily, Monte Busambra	37.84	13.42	
107	machaon	13-T686	1	MH420066	658 bp	-	-	-		-	-	-	Insitut de Biol	28-Apr-2013	Italy: Sardinia, Near Mount Sette Fratelli	39.256	9.367	
108	machaon	14-N963	1	MN139878	658 bp	-	-	-		-	-	-	Leonardo Dap	22-Jul-2014	Italy: Loppio	45.86	10.934	
109	machaon	14-U798	1	MN144041	658 bp	-	-	-		-	-	-	Dapporto, Leonardo		Italy: Bracciano	41.102	12.174	
110	machaon	14-U815	1	MH418666	658 bp	-	-	-		-	-	-	Dapporto, Lec	05-May-2010	Italy: Reggio Calabria	38.18	15.71	
111	machaon	15-A507	1	MH419041	658 bp	-	-	-		-	-	-	L. Forbicioni	08-May-2015	Italy: Gorgona, Gorgona	43.43	9.9	20
112	machaon	15-A537	1	MH420052	658 bp	-	-	-		-	-	-	L. Dapporto	01-May-2013	Italy: Elba, San Piero	42.75	10.2	
113	machaon	15-A539	1	MH418785	658 bp	-	-	-		-	-	-	L. Dapporto	08-Oct-2010	Italy: Tuscany, San Donato	43.511	11.231	
114	machaon	15-A555	1	MN145113	658 bp	-	-	-		-	-	-	L. Dapporto	12-May-2015	Italy: Marches, Monte Nerone	43.545	12.505	1200
115	machaon	15-A902	1	MN140552	658 bp	-	-	-		-	-	-	L. Dapporto	12-May-2015	Italy: Marches, Fano	43.768	12.981	
116	machaon	15-A926	1	MH418640	658 bp	-	-	-		-	-	-	L. Dapporto	14-Jun-2015	Italy: Tuscany, Colognole, Livorno	43.501	10.451	
117	machaon	15-C218	1	MN144867	623 bp	-	-	-		-	-	-	Insitut de Biol	03-Jun-2015	Italy: Matera, Matera	40.667	16.614	347
118	machaon	15-L906	1	MN142930	658 bp	-	-	-		-	-	-	Insitut de Biol	03-Jul-2015	Italy: Monte Matajur	46.208	13.526	1475
119	machaon	15-M986	1	MN140952	658 bp	-	-	-		-	-	-	Insitut de Biol	23-Jun-2015	Italy: Vasto	42.133	14.657	
120	machaon	15-M997	1	MN140078	658 bp	-	-	-		-	-	-	Insitut de Biol	23-Jun-2015	Italy: Termoli	41.955	14.966	50
121	machaon	15-N087	1	MH420039	658 bp	-	-	-		-	-	-	Insitut de Biol	26-May-2015	Italy: Toscana, Monte Calvi	43.079	10.613	
122	machaon	16-A553	1	MN139298	658 bp	-	-	-		-	-	-	DAPPORTO-SC	28-Mar-2016	Italy: Sicily, Fiumara, Modica	36.8259	14.7242	

0	Spcies	Sample ID	Photos	COI Accession	lengh	Ca-ATPase	CAD	28S	Phylogeny	*16S*	*wsp*	*ftsZ*	Collectors	Collection Date		Lat	Lon	Elev
123	machaon	2005MAC01	0	OR048300	658 bp	-	-	-		-	-	-		19-Jun-2005	China	25.6392	104.20042	
124	machaon	2017MAC01	0	OR048301	658 bp	-	-	-		-	-	-		10-Jul-2017	China	28.124298	104.248181	750
125	machaon	606NK-DT18	1	PQ884932	658 bp	-	-	-		-	-	-	David Threatfu	12-May-1999	Canada: British Columbia, nr Vernon, Goose Lake	50.313	-119.286	
126	machaon	606NK-DT19	1	PQ885003	658 bp	-	-	-		-	-	-	David Threatfu	12-May-1999	Canada: British Columbia, nr Vernon, Goose Lake	50.313	-119.286	
127	machaon	606NK-DT20	1	PQ884816	658 bp	-	-	-		-	-	-	David Threatfu	12-May-1999	Canada: British Columbia, nr Vernon, Goose Lake	50.313	-119.286	
128	machaon	606NK-DT21	1	PQ884881	658 bp	-	-	-		-	-	-	David Threatfu	12-May-1999	Canada: British Columbia, nr Vernon, Goose Lake	50.313	-119.286	
129	machaon	606NK-DT22	1	PQ884826	658 bp	-	-	-		-	-	-	David Threatfu	12-May-1999	Canada: British Columbia, nr Vernon, Goose Lake	50.313	-119.286	
130	machaon	766NK-2811	1	PQ884989	658 bp	-	-	-		-	-	-	NG Kondla	27-Jun-2005	Canada: Yukon Territory, km 13 Fish Lake road nr Whitehorse	60.65	-135.229	
131	machaon	766NK-2813	1	PQ884796	658 bp	-	-	-		-	-	-	NG Kondla	27-Jun-2005	Canada: Yukon Territory, km 18 Annie Lake road	60.34	-134.98	
132	machaon	766NK-BB814	1	PQ884837	656 bp	-	-	-		-	-	-	B Beck	18-Jun-2005	Canada: Yukon Territory, S of Whitehorse	60.558	-134.876	
133	machaon	766NK-JB216	1	PQ884839	658 bp	-	-	-		-	-	-	J Beck	18-Jun-2005	Canada: Yukon Territory, Montana Mountain	60.159	-134.703	
134	machaon	AC-PQ001	1	PQ884921	582 bp	-	-	-		-	-	-	ex Osipov	17-Jun-2008	Russia: Sakha, Yakutia, NE Siberia, Oimjakon district, Indigirka rive	64.56	143.224	
135	machaon	AC-PQ002	1	PQ884812	603 bp	-	-	-		-	-	-	ex Osipov	17-Jun-2008	Russia: Sakha, Yakutia, NE Siberia, Oimjakon district, Indigirka rive	64.56	143.224	
136	machaon	AC-PQ008	1	PQ884793	614 bp	-	-	-		-	-	-	Bogdan Klejzer	01-Jul-2012	Cyprus	35.126	33.43	
137	machaon	AC-PQ011	1	PQ884901	658 bp	-	-	-		-	-	-	Yuri Shevnin	21-Jun-2006	Armenia: Kotayk', 25 km NE of Yerevan, Hatis Mt.	40.335	44.733	2000
138	machaon	AC-PQ020	1	PQ884979	621 bp	-	-	-		-	-	-		15-Jun-2002	Russia: Lipetskaya Oblast, Lipetsk, Zaritsky	52.612	39.598	
139	machaon	AC-PQ026	1	PQ884820	627 bp	-	-	-		-	-	-		15-Jan-2012	China: Gansu, NW Lanzhou, Ta-tung-kiao	36.525	102.878	
140	machaon	AC-SP1622	2	PQ884991	658 bp	-	-	-		-	-	-		15-Jul-2008	China: Sichuan, Ya`an	29.981	103.013	2300
141	machaon	AC-SP1625	2	PQ884857	658 bp	-	-	-		-	-	-		28-Jun-2009	Afghanistan: Bamyan, Bandi-Amir	34.846	67.225	3000
142	machaon	AC-SP1628	2	PP865974	627 bp	-	-	-		-	-	-		01-Aug-2009	Russia: Primorskiy Kray, S. Ussuri, Khasan District' Barisoviy Res	43.193	131.491	
143	machaon	AC-SP1639	2	PQ884815	614 bp	-	-	-		-	-	-		20-Jun-2008	Kyrgyzstan: Chuy, N. Tianshan Mts., Trans-Ili Range, Kichi-Kemer	42.879	76.133	2200
144	machaon	AC-SP1643	2	PQ884847	658 bp	-	-	-		-	-	-		07-Jul-2007	Mongolia: SW Mongolia, Uvhod Ula Mt., 45 km. SW Bulgan, Hovd	45.733	91.083	1300
145	machaon	AC-SP1645	2	PQ884734	658 bp	-	-	-		-	-	-		01-Jul-2007	Russia: Kemerovskaya Oblast, Kemerovo, nr. Kemerovo city	55.344	86.062	400
146	machaon	AC-SP1651	2	PQ884994	658 bp	-	-	-		-	-	-		23-Jun-2006	Russia: Zabaykalsky Kray, N. Chita region, Kodar Mts.' Novaya Ch	56.794	118.273	1800
147	machaon	AC-SP1653	2	PQ884978	658 bp	-	-	-		-	-	-		11-Jul-2009	Kyrgyzstan: Karatau Mts., Inner Tienshan, Uljun river	41.308	75.553	3150
148	machaon	AC-SP1654	2	PQ884798	658 bp	-	-	-		-	-	-		24-Jun-2009	Kyrgyzstan: Kichik-Alai, Ak-Bura River, Maidontal	40.03	72.91	2000
149	machaon	AC-SP1658	2	PQ885016	658 bp	-	-	-		-	-	-		16-Jul-2009	Tajikistan: Peter the Great Mts., Muksu River	39.256	71.433	2550
150	machaon	AC-SP1745	2	PQ884750	627 bp	-	-	-		-	-	-		11-May-2009	Russia: Voronezhskaya Oblast, Voronezh	51.668	39.192	
151	machaon	AC-VT002	1	PQ884817	658 bp	-	-	-		-	-	-	Fred Goussey	29-Jun-2007	Russia: Karachayevo-Cherkesiya Republic, Caucasus, Neberda	43.427	41.738	
152	machaon	AC-VT003	1	PQ884877	658 bp	-	-	-		-	-	-	D.G. Zamolodc	14-Jul-2010	Russia: Khabarovskiy Kray, Khabarovsk, Lazo district' Pavlenkov	47.963	135.251	
153	machaon	AC-VT005	1	PQ884871	652 bp	-	-	-		-	-	-		08-Jul-2010	Russia: Amurskaya Oblast, Amur, Bureja	50.263	130.276	
154	machaon	AC-VT007	1	PP865971	610 bp	-	-	-		-	-	-	A. Filippov	02-Jul-2008	Russia: Buryatiya Republic, Buryatia, Ulan Ude	51.833589	107.584043	
155	machaon	AC-VT011	1	PQ884735	579 bp	-	-	-		-	-	-	A. Filippov	01-Jun-2010	Russia: Buryatiya Republic, Buryatia, Mondy	51.676	100.996	1500
156	machaon	AC-VT012	2	PQ884806	521 bp	-	-	-		-	-	-	Yuriy Shevnin	01-Jul-2006	Russia: Yamalo-Nenets Autonomous Okrug, Polar Ural, Slantsev	66.846	65.843	
157	machaon	AC-VT016	2	PQ885007	658 bp	-	-	-		-	-	-	Roman Yakovle	03-Jul-2005	Mongolia: Hovd, Tsagduuldai Valley, 50 km N of Uench Aimak	46.35	92.083	2800
158	machaon	AC-VT017	2	PQ884911	598 bp	-	-	-		-	-	-	D. Belousov leg	28-Jun-2003	Russia: Novosibirskaya Oblast, Novosibirsk, Iskitim	57.643	83.308	150
159	machaon	AC-VT018	2	PQ884778	514 bp	-	-	-		-	-	-	S. Shishigin leg.	15-Jun-2007	Russia: Sakha-Yakutiya Republic, S of Yakutsk, Sakha, Lena River' F	61.477	129.125	
160	machaon	AC-VT020	2	PQ884976	307 bp	-	-	-		-	-	-	ex David Cassat	01-May-2007	China: Guangxi, N Guangxi, Yuecheng Ling, Ziyuan (Dahe)	26.22	110.876	1000
161	machaon	BC ZSM Lep 21843	1	JF415720	658 bp	-	-	-		-	-	-	P.Stamer	12-Sep-2006	Germany: Bavaria, Oberbayern, Diessen	47.93333	11.08333	600
162	machaon	BC ZSM Lep 27060	1	GU707119	658 bp	-	-	-		-	-	-	Dr. Andreas H.	13-Jul-2009	Germany: Bavaria, Oberpfalz, Regensburg' Irlbach bei Wenzenba	49.0663	12.16365	385
163	machaon	BC ZSM Lep 30335	1	HM910498	658 bp	-	-	-		-	-	-	A.Hausmann	08-Aug-2009	Italy: Basilicata, Potenza, Valle del Noce, Trecchina	40.16361	15.90028	300
164	machaon	BC ZSM Lep 56960	1	PQ884860	658 bp	-	-	-		-	-	-			Israel: Jerusalem	31.768319	35.21371	
165	machaon	BC ZSM Lep 72611	1	MH419058	658 bp	-	-	-	x	-	-	-	D. Gerber	27-Jul-2012	Germany: Saarland, St. Wendel, Wolfersweiler, Flachshuebel	49.56254	7.179925	378
166	machaon	BC ZSM Lep 87046	1	MH418967	658 bp	-	-	-		-	-	-	Dieter Doczkal	07-Jul-2014	Germany: Bavaria, Unterfranken, Eussenheim-Aschfeld, Ammer	50.007	9.806	280
167	machaon	BC ZSM Lep 87277	1	MH419683	658 bp	-	-	-		-	-	-	D.Doczkal, S.Sch	20-Jul-2014	Germany: Bavaria, Allgaeu, Oybach' Oytalhaus	47.388649	10.335809	
168	machaon	BIOUG00716-A08	1	JN298783	658 bp	-	-	-		-	-	-	D.Marsden	22-Aug-2010	United States: Arizona, Yavapai Co., 25 mi W Cottonwood Ming	34.696	-112.168	
169	machaon	BIOUG26668-A01	0	MG363262	612 bp	-	-	-		-	-	-	C.S. Guppy	05-Jul-2014	Canada: Yukon Territory, Whitehorse, Mount McIntyre	60.6344	-135.162	1360
170	machaon	BIOUG26668-A02	0	MG357342	558 bp	-	-	-		-	-	-	C.S. Guppy	05-Jul-2014	Canada: Yukon Territory, Whitehorse, Mount McIntyre	60.6344	-135.162	1360
171	machaon	BIOUG26668-A03	0	MG361075	591 bp	-	-	-		-	-	-	C.S. Guppy	05-Jul-2014	Canada: Yukon Territory, Whitehorse, Mount McIntyre	60.6344	-135.162	1360
172	machaon	BIOUG26668-A04	0	MG361983	567 bp	-	-	-		-	-	-	C.S. Guppy	05-Jul-2014	Canada: Yukon Territory, Whitehorse, Mount McIntyre	60.6344	-135.162	1360
173	machaon	BIOUG26668-A05	0	MG360514	576 bp	-	-	-		-	-	-	C.S. Guppy	05-Jul-2014	Canada: Yukon Territory, Whitehorse, Mount McIntyre	60.6344	-135.162	1360
174	machaon	BIOUG26668-A06	0	MG364300	555 bp	-	-	-		-	-	-	C.S. Guppy	05-Jul-2014	Canada: Yukon Territory, Whitehorse, Mount McIntyre	60.6344	-135.162	1360
175	machaon	BIOUG26668-A07	0	MG363256	591 bp	-	-	-		-	-	-	C.S. Guppy	05-Jul-2014	Canada: Yukon Territory, Whitehorse, Mount McIntyre	60.6344	-135.162	1360
176	machaon	BIOUG26668-A08	0	MG361721	579 bp	-	-	-		-	-	-	C.S. Guppy	05-Jul-2014	Canada: Yukon Territory, Whitehorse, Mount McIntyre	60.6344	-135.162	1360
177	machaon	BJUP589	1	PQ884908	645 bp	-	-	-		-	-	-	John James Wilson, Gary Sing Kon		China: Beijing Shi, Nanhaizi Park	39.77303	116.475296	
178	machaon	BJUP590	1	PQ884867	645 bp	-	-	-		-	-	-	John James Wilson, Gary Sing Kon		China: Beijing Shi, Nanhaizi Park	39.77303	116.475296	
179	machaon	BOT1	0	PP087364	584 bp	-	-	-		-	-	-			Mongolia: Khuvsgul	50.220448	100.321376	
180	machaon	BOT11	0	PP087370	584 bp	-	-	-		-	-	-			Mongolia: Dundgobi	45.582278	106.764421	
181	machaon	BOT12	0	PP087371	584 bp	-	-	-		-	-	-			Mongolia: Khentii	47.608122	109.937284	
182	machaon	BOT16	0	PP087372	584 bp	-	-	-		-	-	-			Mongolia: Selenge	49.018355	106.333429	
183	machaon	BOT17	0	PP087373	584 bp	-	-	-		-	-	-			Mongolia: Selenge	49.018355	106.333429	

0	Spcies	Sample ID	Photos	COI Accession	lengh	Ca-ATPase	CAD	28S	Phylogeny	*16S*	*wsp*	*ftsZ*	Collectors	Collection Date		Lat	Lon	Elev
184	machaon	BOT18	0	PP087374	584 bp	-	-	-		-	-	-			Mongolia: Dundgobi	45.582278	106.764421	
185	machaon	BOT19	0	PP087375	584 bp	-	-	-		-	-	-			Mongolia: Dundgobi	45.582278	106.764421	
186	machaon	BOT2	0	PP087365	584 bp	-	-	-		-	-	-			Mongolia: Khuvsgul	50.220448	100.321376	
187	machaon	BOT20	0	PP087376	584 bp	-	-	-		-	-	-			Mongolia: Khentii	47.608122	109.937284	
188	machaon	BOT21	0	PP087377	584 bp	-	-	-		-	-	-			Mongolia: Dundgobi	45.582278	106.764421	
189	machaon	BOT22	0	PP087378	584 bp	-	-	-		-	-	-			Mongolia: Selenge	49.018355	106.333429	
190	machaon	BOT23	0	PP087379	584 bp	-	-	-		-	-	-			Mongolia: Khentii	47.608122	109.937284	
191	machaon	BOT24	0	PP087380	584 bp	-	-	-		-	-	-			Mongolia: Khentii	47.608122	109.937284	
192	machaon	BOT25	0	PP087381	584 bp	-	-	-		-	-	-			Mongolia: Dundgobi	45.582278	106.764421	
193	machaon	BOT26	0	PP087382	584 bp	-	-	-		-	-	-			Mongolia: Selenge	49.018355	106.333429	
194	machaon	BOT3	0	PP087366	584 bp	-	-	-		-	-	-			Mongolia: Dundgobi	45.582278	106.764421	
195	machaon	BOT5	0	PP087367	584 bp	-	-	-		-	-	-			Mongolia: Khuvsgul	50.220448	100.321376	
196	machaon	BOT8	0	PP087368	584 bp	-	-	-		-	-	-			Mongolia: Dundgobi	45.582278	106.764421	
197	machaon	BOT9	0	PP087369	584 bp	-	-	-		-	-	-			Mongolia: Khuvsgul	50.220448	100.321376	
198	machaon	CBjS07-1017r	1	KT147981	658 bp	-	-	-		-	-	-	Schmidt, B. C.	29-May-2003	Canada: Saskatchewan, Cypress Hills, Jones Peak, 10km W Easter	49.502	-108.916	
199	machaon	CCDB-24271-A05	1	PQ885001	658 bp	-	-	-		-	-	-	J.Troubridge	03-May-1990	Canada: British Columbia, Summerland	49.6078	-119.677	
200	machaon	CCDB-24271-E02	1	PQ885015	658 bp	-	-	-		-	-	-	J.Troubridge	24-Mar-1983	Canada: Manitoba, Duck Mountains	51.5	-101	
201	machaon	CCDB-24271-G01	1	PQ884913	658 bp	-	-	-		-	-	-	J.Troubridge	24-Mar-1983	Canada: Manitoba, Duck Mountains	51.5	-101	
202	machaon	CCDB-24271-G02	1	PQ884888	658 bp	-	-	-		-	-	-	F.A.H.Sperling	02-Jul-1983	Canada: Manitoba, Thompson	55.74	-97.85	
203	machaon	CSG21550	1	PQ884760	658 bp	-	-	-		-	-	-	Crispin S Gupp	28-Jun-2003	Canada: British Columbia, Peace River, Peace River; Clayhurst Ec	56.135	-120.035	
204	machaon	CSG21551	1	PQ884980	658 bp	-	-	-		-	-	-	Crispin S Gupp	28-Jun-2003	Canada: British Columbia, Peace River, Peace River; Clayhurst Ec	56.135	-120.035	
205	machaon	CSG21552	1	PQ884882	658 bp	-	-	-		-	-	-	Crispin S Gupp	28-Jun-2003	Canada: British Columbia, Peace River, Peace River; Clayhurst Ec	56.135	-120.035	
206	machaon	CSG23132 NEBC	0	PQ884927	658 bp	-	-	-		-	-	-	Crispin S Gupp	25-Jun-2004	Canada: British Columbia, Northeast BC, Pink Mountain, sout	57.049	-122.867	
207	machaon	CSG23133 NEBC	0	PQ884792	658 bp	-	-	-		-	-	-	Crispin S Gupp	25-Jun-2004	Canada: British Columbia, Northeast BC, Pink Mountain, sout	57.049	-122.867	
208	machaon	CSG23134 NEBC	0	PQ884855	658 bp	-	-	-		-	-	-	Crispin S Gupp	25-Jun-2004	Canada: British Columbia, Northeast BC, Pink Mountain, sout	57.049	-122.867	
209	machaon	DH000073	1	PQ884971	574 bp	-	-	-		-	-	-	D. Handfield	01-Jul-2002	Canada: Quebec, Baie James	51.700765	-76.046835	
210	machaon	DMEX1	0	MW305954	696 bp	-	-	-		-	-	-			Malta	35.937496	14.375416	
211	machaon	DMEX13	0	MW305955	696 bp	-	-	-		-	-	-			Malta	35.937496	14.375416	
212	machaon	DNA 122	0	FJ808906	1,433 bp	-	-	-		-	-	-			United States: Washington, Wishram	45.657	-120.965	
213	machaon	DNA 152	0	FJ808905	1,433 bp	-	-	-		-	-	-			United States: Montana, McCone County	47.352	-105.592	
214	machaon	DNA 1611	0	FJ808897	1,433 bp	-	-	-		-	-	-			Canada: Alberta			
215	machaon	DNA 223	0	FJ808909	1,433 bp	-	-	-		-	-	-			United States: Alaska, Eagle Summit	65.293	-145.149	
216	machaon	DNA 2300	0	FJ808896	1,433 bp	-	-	-		-	-	-			United States: Alaska, Eagle Summit	65.293	-145.149	
217	machaon	DNA 234	0	FJ808907	1,433 bp	-	-	-		-	-	-			United States: Washington, Thompson Culture	47.39	-120.363	
218	machaon	DNA 260	0	FJ808901	1,433 bp	-	-	-		-	-	-			Canada: British Columbia, Pink Mountain	57.04231	-122.5231	
219	machaon	DNA 261	0	FJ808898	1,433 bp	-	-	-		-	-	-			Canada: British Columbia, Taylor	56.177	-120.665	
220	machaon	DNA 2801	0	FJ808895	1,433 bp	-	-	-		-	-	-			[Alberta BC or Alaska]			
221	machaon	DNA 2804	0	FJ808899	1,433 bp	-	-	-		-	-	-			Canada: Alberta, Road to Judah, south of Peace River	56.2022	-117.2856	
222	machaon	DNA 2819	0	FJ808900	1,433 bp	-	-	-		-	-	-			Canada: British Columbia, Pink Mountain	57.04231	-122.5231	
223	machaon	DNA 2870	0	FJ808902	1,433 bp	-	-	-		-	-	-			Canada: Alberta			
224	machaon	DNA 2872	0	FJ808904	1,433 bp	-	-	-		-	-	-			Canada: Alberta, Southeast Lethbridge	49.6795	-112.8442	
225	machaon	DNA 3056	0	FJ808903	1,433 bp	-	-	-		-	-	-			[Alberta or Montana]			
226	machaon	DNA 77	0	FJ808908	1,433 bp	-	-	-		-	-	-			United States: Washington, Palouse Falls	46.663	-118.223	
227	machaon	DNAcdb0023	1	PQ884835	614 bp	-	-	-		-	-	-	K. Hojgaard	16-Aug-2006	United Kingdom: England, Norfolk, Norfolk Broads	52.689	1.609	
228	machaon	DNAcdb0024	1	PQ884850	658 bp	-	-	-		-	-	-	J. Klir	21-Feb-2007	Oman: Hail	23.283	57.917	400
229	machaon	DNAcdb0025	1	PQ884748	658 bp	-	-	-		-	-	-	J. Klir	15-Feb-2007	Oman: Qaroot	23	57.783	600
230	machaon	DNAcdb0031	1	PQ884718	658 bp	-	-	-		-	-	-	Kairouz	15-Sep-2006	Lebanon: Tripoli	34.435	35.836	10
231	machaon	DNAcdb0035	1	PQ884747	658 bp	-	-	-		-	-	-	Local Collector	15-Jul-2005	Afghanistan: Parwan, Panjshir Valley	35.503	69.955	
232	machaon	DNAcdb0043	1	PQ884926	633 bp	-	-	-		-	-	-		17-Jun-2004	Russia: Kamchatskiy Kray, Milkovo disrtict, Kluckvennaya river' 3	54.422	158.489	
233	machaon	DNAcdb0056	1	PQ884825	658 bp	-	-	-		-	-	-		12-Jun-2003	Russia: Sakha, Yakutia, Aldan district' Tommot village	58.964	126.273	600
234	machaon	DNAcdb0076	1	PQ884797	636 bp	-	-	-		-	-	-	L.Bieber	02-Jul-2006	China: Tibet, Ngari prefecture, Nalakankar Himalaya' Mandhata M	30.519	81.278	5000
235	machaon	DNAcdb0098	1	PQ884920	658 bp	-	-	-		-	-	-	Della Bruna	04-Aug-2006	China: Sichuan, Maoxian-Songpan	31.949	103.698	1750
236	machaon	DNAwth002	2	PQ884845	658 bp	-	-	-		-	-	-	Martina, Anja,	17-Jul-2013	Russia: Kabardino-Balkariya Republic, Caucasus, Elbrus' Kabard	43.257	42.514	2300
237	machaon	DNAwth003	2	PQ884729	658 bp	-	-	-		-	-	-	Wolfgang, Anja	18-Jun-2012	Russia: Novgorodskaya Oblast, 300 km NW Moscow, 5 km SE Va	57.935	33.345	200
238	machaon	DNAwth004	2	PQ884769	307 bp	-	-	-		-	-	-	W. ten Hagen	18-Apr-1993	Jordan: Irbid, Oum Quais	32.654	35.688	500
239	machaon	DNAwth005	2	PQ884917	658 bp	-	-	-		-	-	-	W. ten Hagen	21-Jun-1997	Turkey: Rize, N. Ovit Gecidi, 2 km N Sivrikaya	40.7	40.721	1550
240	machaon	DNAwth008	2	PQ884895	307 bp	-	-	-		-	-	-		10-Jun-1989	Turkey: Taskopru, 30 km E Taskopru	41.513	34.399	300
241	machaon	DNAwth009	2	PQ884909	396 bp	-	-	-		-	-	-	W. ten Hagen	01-Aug-1992	Turkey: Thrakia, N. Uzunkopru	41.267	26.688	50
242	machaon	DNAwth012	2	PQ884894	627 bp	-	-	-		-	-	-	Wolfgang, Anja	05-Jun-2012	Uzbekistan: Hissar Mountains, Qashqadarya, SE Sharisabz, vic. Ta	38.844	67.09	1300
243	machaon	DNAwth014	2	PQ884739	627 bp	-	-	-		-	-	-	Wolfgang, Mar	09-Jun-2012	Uzbekistan: Pskemsky Mountains, NE Tashkent, S. Chimgan, Beld	41.491	69.882	2200
244	machaon	DNAwth015	2	PQ884809	658 bp	-	-	-		-	-	-		22-May-2013	Tajikistan: S. Ghissar Mts., Kalininabad city environs, Tabakchi rar	39.738	69.151	600

0	Spcies	Sample ID	Photos	COI Accession	lengh	Ca-ATPase	CAD	28S	Phylogeny	16S	wsp	ftsZ	Collectors	Collection Date		Lat	Lon	Elev
245	machaon	DNAwth016	2	PQ884876	627 bp	-	-	-		-	-	-	V. Zurilina	12-Jun-2015	Russia: Altay Republic, SW Siberia, Altai Mts., Ulagansky District' /	50.311	87.599	1500
246	machaon	DNAwth017	2	PQ885030	627 bp	-	-	-		-	-	-	Goshko	10-Jul-2017	Tajikistan: SE Pamir Mts, Murgab district, Ak-Bura massif, Kurus	37.823	74.647	4000
247	machaon	DNAwth018	2	PQ884970	627 bp	-	-	-		-	-	-	Goshko	22-Jun-2018	Tajikistan: W Pamir, Shungan range, Chorog city environs, Sango	37.488	71.59	4100
248	machaon	DNAwth019	2	PQ884818	658 bp	-	-	-		-	-	-	Goshko	07-Jun-2018	Tajikistan: Ghissar mts., Romit reserve, Sorbo village vicinity	38.817	69.486	2900
249	machaon	DNAwth020	2	PQ884771	473 bp	-	-	-		-	-	-	W. ten Hagen	08-May-2007	Iran: Zanjan, E. Talish, NE Abbar	36.945	48.985	1400
250	machaon	DNAwth022	2	PQ884940	620 bp	-	-	-		-	-	-	W. ten Hagen	01-Aug-2004	Iran: East Azerbaijan, N Ahar, vic. Kaleybar	38.862	47.046	1700
251	machaon	DNAwth024	2	PQ884910	643 bp	-	-	-		-	-	-	W. ten Hagen	09-Aug-2003	Iran: Mazandaran, central Elburs, N. Kendevan, vic. Valiabad	36.252	51.302	1700
252	machaon	DNAwth025	2	PQ884878	627 bp	-	-	-		-	-	-	W. ten Hagen	02-Jul-2010	Iran: Esfahan, N. Semirom, (N. Stadtrand)	31.477	51.565	2700
253	machaon	DNAwth026	2	PQ884853	627 bp	-	-	-		-	-	-	W. ten Hagen	16-May-2007	Iran: Kerman, N Kerman, Kuh-e Kuhpayeh, vic. Kuhpayeh	30.497	57.204	2700
254	machaon	DNAwth027	2	PQ885035	627 bp	-	-	-		-	-	-	W. ten Hagen	14-May-2010	Iran: Kerman, N. Baft, Gardaneh Qaleh Askar	29.486	56.67	2850
255	machaon	DNAwth028	2	PQ884864	658 bp	-	-	-		-	-	-	W. ten Hagen	05-Jun-2004	Iran: Khorasan, Kuh-e Sorkh, S. Robat-e Sang, vic. Kameholya	35.486	59.17	1800
256	machaon	DNAwth030	2	PQ884804	658 bp	-	-	-		-	-	-	W. ten Hagen	07-Jul-2002	Iran: Lorestan, Pass SW Dorud-Khorramabad	33.394	48.711	2100
257	machaon	EZ0001CNC	1	PQ885002	658 bp	-	-	-		-	-	-	J. & L. Troubri	22-Jun-1990	Canada: British Columbia, 2 km W of Taylor	56.1499	-120.715	
258	machaon	EZ0002CNC	1	PQ884869	658 bp	-	-	-		-	-	-	J. & L. Troubri	22-Jun-1990	Canada: British Columbia, 2 km W of Taylor	56.1499	-120.715	
259	machaon	EZ0003CNC	1	PQ884924	658 bp	-	-	-		-	-	-	J. & L. Troubri	22-Jun-1990	Canada: British Columbia, 2 km W of Taylor	56.1499	-120.715	
260	machaon	EZ0004CNC	1	PQ884783	658 bp	-	-	-		-	-	-	J. & L. Troubri	22-Jun-1990	Canada: British Columbia, 2 km W of Taylor	56.1499	-120.715	
261	machaon	EZ0005CNC	1	PQ884773	658 bp	-	-	-		-	-	-	J. & L. Troubri	22-Jun-1990	Canada: British Columbia, 2 km W of Taylor	56.1499	-120.715	
262	machaon	EZ0006CNC	1	PQ884791	658 bp	-	-	-		-	-	-	J. & L. Troubri	22-Jun-1990	Canada: British Columbia, 2 km W of Taylor	56.1499	-120.715	
263	machaon	EZ0007CNC	1	PQ884969	658 bp	-	-	-		-	-	-		13-May-1992	Canada: British Columbia, Spences Bridge	50.4137	-121.359	
264	machaon	EZ0008CNC	1	PQ884743	658 bp	-	-	-		-	-	-		17-Jun-1987	Canada: Yukon Territory, Dempster Hwy KM.411	66.55	-136.34	
265	machaon	EZ0009CNC	1	PQ884958	658 bp	-	-	-		-	-	-		19-Jun-1987	Canada: Yukon Territory, Dempster Hwy KM.411	66.55	-136.34	
266	machaon	EZ0010CNC	1	PQ884819	658 bp	-	-	-		-	-	-		16-Jun-1987	Canada: Yukon Territory, Dempster Hwy KM.411	66.55	-136.34	
267	machaon	EZ0011CNC	1	PQ884757	658 bp	-	-	-		-	-	-	J. & L. Troubri	27-Jun-1985	Canada: Yukon Territory, Dempster Hwy KM.270	65.84	-137.7	
268	machaon	EZ1197CNC	1	PQ884780	658 bp	-	-	-		-	-	-	Richard Popko	15-Jun-2006	Canada: Northwest Territories, Dodo Canyon, Mackenzie Mtns	64.932	-127.341	
269	machaon	EZ1198CNC	1	PQ885022	658 bp	-	-	-		-	-	-	Richard Popko	15-Jun-2006	Canada: Northwest Territories, Dodo Canyon, Mackenzie Mtns	64.932	-127.341	
270	machaon	EZ1199CNC	1	PQ884950	658 bp	-	-	-		-	-	-	Richard Popko	20-Jun-2006	Canada: Northwest Territories, Jawbone Lake, Mackenzie Mtns.	64.992	-127.615	
271	machaon	EZ1393CNC	1	PQ885028	658 bp	-	-	-		-	-	-	R. A. Layberry	19-Jun-2005	Canada: Northwest Territories, 2 km S Fort Good Hope	66.236	-128.641	
272	machaon	EZ1394CNC	1	PQ884941	658 bp	-	-	-		-	-	-	R. A. Layberry	26-Jun-2005	Canada: Northwest Territories, Canol Heritage Trail, mi 224.1	63.282	-129.869	
273	machaon	GBIFCH-BOL_LEPAA_0022	0	MK186609	654 bp	-	-	-		-	-	-	Sanchez Andre	30-Jul-2013	Switzerland: Fribourg, Plasselb	46.726	7.24242	
274	machaon	GBIFCH-BOL_LEPAA_0039	0	MK186610	639 bp	-	-	-		-	-	-	Palmi Paolo	15-Jul-2013	Switzerland: Ticino, Morcote	45.9068	8.92058	
275	machaon	GBIFCH-BOL_LEPAA_0276	0	MK186611	621 bp	-	-	-		-	-	-	Chittaro Yann	16-Apr-2013	Switzerland: Valais, Pfynwald	46.2763	7.63323	
276	machaon	GBIFCH-BOL_LEPAA_0466	0	MK186608	654 bp	-	-	-		-	-	-	Mayr Anton	08-Jun-2013	Switzerland: Graubunden, Piz Beverin	46.6205	9.3319	
277	machaon	GCB02	2	PQ884776	658 bp	-	-	-		-	-	-		12-Jun-2003	Russia: Buryatiya Republic, Ilka village	51.72	108.518	
278	machaon	GCB05	2	PQ884844	580 bp	-	-	-		-	-	-	G.C. Bozano	07-Jul-2003	China: Hubei, W Hubei, Wudang Shan, N. of Zhushan	32.575	110.233	800
279	machaon	GCB06	2	PQ884880	658 bp	-	-	-		-	-	-	G.C. Bozano	02-Jul-2003	China: Henan, W Henan, 30 km N. of Xiping	33.642	111.179	1100
280	machaon	GCB08	2	PQ884903	619 bp	-	-	-		-	-	-	Zamolodchiko	01-Jul-2006	Russia: Chukotka Autonomous Okrug, E Chukotka, Chukotsky	65.94	179.564	
281	machaon	GCB09	2	PQ884779	627 bp	-	-	-		-	-	-	A. Floriani, A. S	08-Jul-2008	China: Inner Mongolia, Hinggang Mts., 50 km NE Arxan Mt' road	47.356	120.019	950
282	machaon	KWP252-015	0	PQ884899	658 bp	-	-	-		-	-	-	Ken Philip	24-May-2005	United States: Alaska, MUD			
283	machaon	KWP252-016	0	PQ884787	616 bp	-	-	-		-	-	-	Ken Philip	19-May-2005	United States: Alaska, BCF			
284	machaon	KWP252-017	0	PQ885000	658 bp	-	-	-		-	-	-	Ken Philip	13-May-2004	United States: Alaska, BCF			
285	machaon	LEP-SS-00141	1	MH419239	658 bp	-	-	-		-	-	-	Stefano Scaler	18-Sep-2014	Italy: Calabria, Pantano Martucci	39.6158	16.7656	
286	machaon	LEP-SS-00142	1	MH420151	658 bp	-	-	-		-	-	-	Stefano Scaler	03-Aug-2014	Italy: Calabria, Mendicino	39.2819	16.2067	
287	machaon	LEP-SS-00929	1	PQ884982	658 bp	-	-	-		-	-	-	Scalercio Stefa	29-Aug-2018	Italy: Calabria, Cozzo Lungo, Morano Cal. (CS)	39.8889	16.0473	995
288	machaon	LEP-SS-00932	1	PQ885025	657 bp	-	-	-		-	-	-	Scalercio Stefa	04-Aug-2018	Italy: Calabria, Casino Blotta	39.8973	16.0422	1150
289	machaon	LEP-SS-00938	1	PQ884900	658 bp	-	-	-		-	-	-	Scalercio Stefa	04-Aug-2018	Italy: Calabria, Cozzo Lungo, Morano Cal. (CS)	39.8889	16.0473	995
290	machaon	LEP-SS-00981	1	PQ884807	624 bp	-	-	-		-	-	-	Scalercio Stefa	28-Aug-2018	Italy: Calabria, Ianni-Pirillo	39.2166	16.1414	1100
291	machaon	LEP-SS-00983	1	PQ884823	658 bp	-	-	-		-	-	-	Scalercio Stefa	14-Aug-2018	Italy: Calabria, Fosso Cucolo	39.2369	16.2972	550
292	machaon	LEP-SS-00985	1	PQ884998	658 bp	-	-	-		-	-	-	Scalercio Stefa	28-Aug-2018	Italy: Calabria, Monte Cocuzzo	39.219	16.133	1500
293	machaon	LEP-SS-01024	1	PQ884745	658 bp	-	-	-		-	-	-	Scalercio Stefa	29-Aug-2018	Italy: Calabria, Colle Dragone	39.8933	16.0787	1440
294	machaon	LEP-SS-01029	1	PQ884765	658 bp	-	-	-		-	-	-	Scalercio Stefa	29-Aug-2018	Italy: Calabria, Commenda di Malta, Frascineto (CS)	39.8403	16.2572	535
295	machaon	LN-BD1337	1	KX041639	658 bp	-	-	-		-	-	-	Bernard Dard	28-Apr-1990	France: Normandie, Haute Normandie, Eure' Amfreville sous les	49.305	1.26	
296	machaon	LOWA-NL-5	1	PP865975	658 bp	-	-	-		-	-	-	Andrei Sourak	18-May-1997	Nepal: Jumla, S. Slope	29.25	82.25	2600
297	machaon	LOWA-NL-6	1	PQ884859	658 bp	-	-	-		-	-	-	Andrei Sourak	18-May-1997	Nepal: Jumla, S. Slope	29.25	82.25	2600
298	machaon	MCH018	0	PQ884945	658 bp	-	-	-		negative	negative	negative		06-Oct-2018	Oman: Quraisa, Jabal Nakhl	29.94	33.7	600
299	machaon	MCH020	0	PV173971	658 bp	-	-	-		-	-	-		24-May-1999	Malta: Zebbug	35.9	14.4	
300	machaon	MCH023	0	PQ884833	658 bp	-	-	-		-	-	-			Libya: Tripoli	32.9	13.2	
301	machaon	MCH025	0	PQ884977	658 bp	-	-	-		-	-	-		24-May-1999	Malta: Zebbug	35.9	14.4	
302	machaon	MM00727	1	HM871158	658 bp	-	-	-		-	-	-	Marko Mutan	03-Jul-2001	Finland: Ostrobottnia ouluensis, Liminka	64.759	23.217	
303	machaon	MM05450	1	HM872835	658 bp	-	-	-		-	-	-	Meelika Mutan	06-Jun-2007	Finland: Northern Ostrobothnia, Ostrobottnia ouluensis, Kiin	65.071	25.725	
304	machaon	MM05702	0	HM872901	658 bp	-	-	-		-	-	-			Finland: North Karelia, Karelia borealis, Liperi	62.545	29.332	
305	machaon	NHMO-DAR-11929	1	PQ884788	658 bp	-	-	-		-	-	-	Svein Grundet	08-Jun-2002	Norway: Vest-Agder, Kristiansand, Nedre Timenes	58.1611	8.099289	

0	Spcies	Sample ID	Photos	COI Accession	lengh	Ca-ATPase	CAD	28S	Phylogeny	16S	wsp	ftsZ	Collectors	Collection Date		Lat	Lon	Elev
306	machaon	NIBGEBUT-00155	1	KC158431	658 bp	-	-	-		-	-	-	S. Akhtar	03-Oct-2010	Pakistan: Azad Kashmir, Sudhnati, Pallandri	33.7	73.6833	1355
307	machaon	not given	0	AF044007	2,291 bp	-	-	-		-	-	-	S. Anderson		United States: Washington, Palouse Falls	46.663479	-118.223577	
308	machaon	not given	0	HM243594	15,185 bp	-	-	-		-	-	-			China: Tianshan Mountains	38.073254	104.69114	
309	machaon	not given	0	HM246461	661 bp	-	-	-		-	-	-			China			
310	machaon	not given	0	LS975121	15,179 bp	-	-	-		-	-	-		24-Apr-1986	United States: Missouri	38.278201	-93.451435	
311	machaon	not given	0	NC_018047	15,185 bp	-	-	-		-	-	-			China: Tianshan Mountains			
312	machaon	OCIC-PM2	2	PQ884951	658 bp	-	-	-		-	-	-	Berquier Cyril	02-Jul-2021	France: Corsica, Olmeto, Tavaria	41.708	8.83	
313	machaon	OCIC-PM8	2	PQ884822	658 bp	-	-	-		-	-	-	Berquier Cyril	08-Sep-2021	France: Corsica, Ventiseri, Base militaire	41.925	9.398	
314	machaon	P6_18	0	MW305956	680 bp	-	-	-		-	-	-			Malta	35.937496	14.375416	
315	machaon	PAP002	1	PQ884883	658 bp	-	-	-		negative	negative	negative	Safronov D.A.	18-Jul-2014	Tajikistan: Pamir, Dzhilondy env.	37.6	72.6	4300
316	machaon	PAP003	1	PQ885040	658 bp	-	-	-		-	-	-		15-Jul-1999	Afghanistan: Panjaw, Koh-i-Baba Mts.	34.4	67	
317	machaon	PAP004	1	PQ885029	658 bp	-	-	-		-	-	-	L. Bieber	08-Jul-1995	Pakistan: Gilgit, Gilgit-Chitral Rd, Shandur Pass	36.1	72.5	3850
318	machaon	PAP005	1	PQ884935	658 bp	-	-	-		negative	negative	negative	D.A. Safronov	17-Jul-2014	Tajikistan: Pamir, Dzhilondy	37.6	72.6	4300
319	machaon	PAP006	1	PQ884721	658 bp	-	-	-		-	-	-	L. Bieber	09-Jul-1996	China: Tibet, West Himalaya, 80 km NW Kailash Mt.	31.5	80.5	4400
320	machaon	PAP007	1	PQ884843	658 bp	PQ885093	-	PQ885057	x	negative	negative	negative	L. Bieber	18-Jun-2006	China: Tibet, 20 km SE of Meshe	31.1	80.9	4600
321	machaon	PAP008	1	PQ885023	658 bp	-	-	-		-	-	-	L. Bieber	08-Jul-1995	Pakistan: Gilgit, Gilgit-Chitral Rd, Shandur Pass	36.1	72.5	3850
322	machaon	PAP009	1	PQ884810	658 bp	-	-	-		-	-	-	L. Bieber	08-Jul-1995	Pakistan: Gilgit, Gilgit-Chitral Rd, Shandur Pass	36.1	72.5	3850
323	machaon	PAP010	1	PQ884862	658 bp	PQ885095	-	PQ885059	x	negative	negative	negative	L. Bieber	02-Jul-2006	China: Tibet, Mandhata Mt.	30.4	81.3	5000
324	machaon	PAP011	1	PP865965	658 bp	-	-	-		negative	negative	negative		15-Aug-2012	India: Ladakh, Leh district, Namshang	33.1	78.3	3700
325	machaon	PAP012	1	PQ884744	658 bp	PQ885085	-	-	x	negative	negative	negative	L. Bieber	12-Jun-2006	China: Tibet, Tsochen pass	30.1	85.5	5200
326	machaon	PAP013	1	PQ884944	658 bp	-	-	-		negative	negative	negative	L. Bieber	02-Jul-2006	China: Tibet, Mandhata Mt.	30.4	81.3	5000
327	machaon	PAP014	1	PQ885008	658 bp	-	-	-		negative	negative	negative	L. Bieber	02-Jul-2006	China: Tibet, Mandhata Mt.	30.4	81.3	5000
328	machaon	PAP015	1	PQ884946	658 bp	-	-	-		negative	negative	negative	L. Bieber	19-Jun-2003	Nepal: Anapurna, Thorong pass	28.8	83.9	5000
329	machaon	PAP042	1	PQ884907	658 bp	-	-	-		negative	negative	negative		05-Jul-2015	China: Sichuan, Yajiang county	30	101	1500
330	machaon	PAP043	1	PQ885009	658 bp	-	-	-		negative	negative	negative		05-Jun-2015	China: Sichuan	30.9	101.9	1500
331	machaon	PAP044	1	PQ884768	658 bp	-	-	-		negative	negative	negative	F. Renner	15-May-2008	China: Jiangsu, Chun An Xin	29.6	119	
332	machaon	PAP045	1	PQ884930	658 bp	-	-	-		negative	negative	negative		10-Dec-2005	China: Guangdong, Guanghzou	23.4	113.5	
333	machaon	PAP046	1	PQ884749	658 bp	PQ885086	-	-	x	negative	negative	negative		03-Aug-2019	China: Anhui, Huabei, Xiangshan park	33	116.79	
334	machaon	PAP048	1	PQ884766	658 bp	PQ885089	-	-	x	negative	negative	negative		10-Sep-2015	India: Himachal Pradesh, Kullu, Jibhi	31.59	77.35	2000
335	machaon	PAP050	1	PQ884805	658 bp	-	-	-		-	-	-	L. Bieber	08-Jul-1995	Pakistan: Gilgit, Gilgit-Chitral Rd, Shandur Pass	36.1	72.5	3850
336	machaon	PAP052	1	PQ884885	658 bp	PQ885097	-	PQ885062	x	negative	negative	negative		05-Jul-2003	Afghanistan: Panjshir valley, Schaba vill.	35.4	69.6	
337	machaon	PAP054a	1	PQ884811	658 bp	-	-	-		negative	negative	negative	B. Khramov	20-Mar-2013	Jordan: As-Salt	32	35.7	
338	machaon	PAP055	1	PQ884988	658 bp	PQ885112	PQ885135	PQ885078	x	negative	negative	negative	A. Krupitsky	19-Jun-2011	Tajikistan: Pamir, Rushan Range, Khuf vill.	37.8	71.6	
339	machaon	PAP055XR	1	PQ885011	658 bp	-	-	-		negative	negative	negative	G. Kuleshov, O	13-Jun-2005	Russia: Kamchatskiy Kray, Milkovsky District, Sharomy vill.	54.404	158.21	200
340	machaon	PAP056XR	1	PP865973	658 bp	-	-	-		negative	negative	negative	V. Zurilina	15-Jul-2015	Russia: Kamchatskiy Kray, Esso vill.	55.9	158.7	
341	machaon	PAP057XR	1	PQ884725	658 bp	-	-	-		negative	negative	negative	V. Zurilina	15-Jul-2015	Russia: Kamchatskiy Kray, Esso vill.	55.9	158.7	
342	machaon	PAP058XR	1	PQ885026	658 bp	-	-	-		negative	negative	negative	G. Kuleshov, O	13-Jun-2005	Russia: Kamchatskiy Kray, Milkovsky District, Sharomy vill.	54.404	158.21	200
343	machaon	PAP059XR	1	PQ884821	658 bp	-	-	-		negative	negative	negative	D. Zamolodchi	02-Jun-2006	Russia: Chukotka Autonomous Okrug, Chukotka, Lavrentia Ba	65.58	-171.01	
344	machaon	PAP061	1	PQ884963	658 bp	-	-	-		negative	negative	negative		15-Jun-2021	China: Liaoning, Shenyang	41.9	123.5	1000
345	machaon	PAP062	1	PQ884794	658 bp	-	-	-		-	-	-			China: Tibet, Ali Pulan	30.3	81.2	
346	machaon	PAP064	1	PQ884720	658 bp	-	-	-		negative	negative	negative		15-Jun-2020	China: Inner Mongolia, Baotou	40.7	109.8	1200
347	machaon	PAP065	1	PP865972	658 bp	-	-	-		negative	negative	negative		15-Apr-2019	China: Fujian, Yongchun County, Yudou	25.4	118	
348	machaon	PAP066	1	PQ884928	658 bp	-	-	-		negative	negative	negative		15-Apr-2019	China: Anhui, Feixi County, Xiaogaozhuang	31.7	117.1	
349	machaon	PAP067	1	PQ884959	658 bp	-	-	-		negative	negative	negative		25-May-2017	China: Jiangsu, Wuxi, Nanquan	31.6	120.3	
350	machaon	PAP069	1	PQ884904	658 bp	-	-	-		negative	negative	negative		15-Jun-2021	China: Shandong, Qingdao	36.3	120.5	
351	machaon	PAP070	1	PQ884984	658 bp	-	-	-		negative	negative	negative		15-Jun-2021	China: Inner Mongolia, Baotou	40.7	109.8	1200
352	machaon	PAP071	1	PQ885021	658 bp	-	-	-		negative	negative	negative		15-Jun-2021	China: Shandong, Qingdao	36.3	120.5	
353	machaon	PAP072	1	PQ884719	658 bp	-	-	-		negative	negative	negative			China: Liaoning, Shenyang	41.9	123.5	1000
354	machaon	PAP073	1	PP865977	658 bp	-	-	-		negative	negative	negative		15-Jun-2021	China: Shandong, Qingdao	36.3	120.5	
355	machaon	PAP078	0	PQ884842	658 bp	-	-	-		negative	negative	negative	N. Shapoval, A.	15-Jul-2021	Russia: Kaliningradskaya Oblast, Zelenogradsk District, Courish S	55.089	20.735	
356	machaon	PAP081	1	PQ884868	658 bp	-	-	-		-	-	-	A. Gorodinski	30-Jul-2007	China: Sichuan, Venchuan	31.5	103.6	1400
357	machaon	PAP089	1	PQ884834	658 bp	-	-	-		negative	negative	negative	K. Tian	20-Jul-2019	South Korea: Gyeonggi, Goyang-Si, Gyenggi	37.738	126.861	250
358	machaon	PAP090	1	PQ884898	658 bp	-	-	-		negative	negative	negative	K. Tian	26-Apr-2019	South Korea: Gyeonggi, Goyang-Si, Gyenggi	37.738	126.861	250
359	machaon	PAP091	1	PQ884746	658 bp	-	-	-		-	-	-	S. Murzin	20-Jun-1990	North Korea: South Hamgyong, Sinpho	40	128.2	
360	machaon	PAP094	1	PQ884889	658 bp	-	-	-		-	-	-		07-Jun-2021	China: Gansu, Wenxian County, Longnan	33.4	104.9	1500
361	machaon	PAP095	1	PQ884831	658 bp	-	-	-	x	-	-	-	F. Renner	15-May-1989	United Kingdom: England, Norfolk, Filby Broad	52.66	1.64	
362	machaon	PAP096	1	PQ884861	658 bp	-	-	-		-	-	-		20-May-2010	United Kingdom: England, Norfolk, Filby Broad	52.66	1.64	
363	machaon	PAP097	1	PQ884848	658 bp	-	-	-		-	-	-		21-May-2010	United Kingdom: England, Norfolk, Filby Broad	52.66	1.64	
364	machaon	PAP100 = HSU03	1	PQ884955	658 bp	-	-	-		negative	negative	negative	Hsu, Frank, Hu	19-Jul-2020	China: Taiwan, Lienchiang County, Matsu Islands' Tiebao, Nanga	26.1534	119.931	
365	machaon	PAP102 = DNAcdb0022	1	PQ884786	658 bp	-	-	-		-	-	-	K. Hojgaard	15-Jul-1996	United Kingdom: England, Norfolk, Norfolk Broads	52.689	1.609	
366	machaon	PAP107	1	PQ884738	658 bp	-	-	-		-	-	-	A. Krupitsky	18-Aug-2023	Iran: Esfahan	32.8653	50.1016	

0	Spcies	Sample ID	Photos	COI Accession	lengh	Ca-ATPase	CAD	28S	Phylogeny	*16S*	*wsp*	*ftsZ*	Collectors	Collection Date		Lat	Lon	Elev
367	machaon	PAP111	2	PQ884736	658 bp	-	-	-		negative	negative	negative	N. Shapoval	23-May-2023	Azerbaijan: Baku	40.42899	49.9953	
368	machaon	PAP113	2	PP865969	610 bp	-	-	-	x	-	-	-			Taiwan			
369	machaon	PAP115	0	PQ884931	658 bp	-	-	-		-	-	-			Nepal			
370	machaon	PAP116	0	PQ884891	658 bp	-	-	-		-	-	-			Nepal			
371	machaon	PAP120	0	PQ884954	658 bp	-	-	-		-	-	-			Israel			
372	machaon	PAP123	0	PQ884948	658 bp	-	-	-		-	-	-			Israel			
373	machaon	PAP124	0	PQ884943	658 bp	-	-	-		-	-	-			Israel: Jerusalem	31.768319	35.21371	
374	machaon	PAP125	0	PQ885032	658 bp	-	-	-		-	-	-			Israel: Jerusalem	31.768319	35.21371	
375	machaon	PAP126	0	PQ884781	658 bp	-	-	-		-	-	-			Israel			
376	machaon	PAP127	0	PQ884829	658 bp	-	-	-		-	-	-			Israel: Jerusalem	31.768319	35.21371	
377	machaon	PAP128	0	PQ884990	658 bp	-	-	-		-	-	-			Israel: Jerusalem	31.768319	35.21371	
378	machaon	pm1	0	OR491823	675 bp	-	-	-		-	-	-			China			
379	machaon	pm110711	0	OM346749	658 bp	-	-	-		-	-	-		11-Jul-2011	Denmark	54.862362	11.911336	
380	machaon	pm210714	0	OM348530	658 bp	-	-	-		-	-	-		21-Jul-2014	Greece	35.524933	23.807733	
381	machaon	RR-PABA-ND001	1	PQ884851	658 bp	-	-	-		-	-	-	Ronald Alan R	28-May-2008	United States: North Dakota, Great Plains, SE4 27-144-102, Bi	45.2586	-103.588	
382	machaon	RV-06-K527	1	HQ004888	658 bp	-	-	-		-	-	-		30-Apr-2006	Romania: Cluj, Transylvania, Badeni	46.484	23.721	
383	machaon	RV-06-K638	1	HQ004890	658 bp	-	-	-		-	-	-		23-May-2006	Romania: Cluj, Transylvania, Baciu forest (Cluj-Napoca)	46.814	23.512	
384	machaon	RV-06-M955	1	HQ004889	652 bp	-	-	-		-	-	-		24-Jun-2006	Romania: Buzau, Muntenia, Dealul Istrita, Breaza	45.094	26.533	
385	machaon	RV-07-D302	1	HQ004887	658 bp	-	-	-		-	-	-		28-May-2007	Romania: Brasov, Transylvania, Dumbrava Vadului, Vad	45.777	25.115	
386	machaon	RV-08-A016	1	HQ004886	646 bp	-	-	-		-	-	-		27-Apr-2003	Romania: Buzau, Muntenia, Dealul Istrita, Coama Istritei	45.094	26.533	
387	machaon	Rvcoll_15-M121	1	MN144211	658 bp	-	-	-		-	-	-	Insitut de Biol	10-Jul-2015	France: Bonneville-Saint Etienne	46.092	6.403	640
388	machaon	Rvcoll_16-A049	1	MH419458	658 bp	-	-	-		-	-	-	Insitut de Biol	18-Aug-2015	Italy: Massarosa	43.866	10.333	3
389	machaon	RVcoll.11-D511	1	MH418722	658 bp	-	-	-		-	-	-	ROGER VILA	18-Apr-2011	Italy: Calabria, Zaccanopoli	38.674	15.926	
390	machaon	RVcoll.14-I304	1	MN144264	658 bp	-	-	-		-	-	-		21-Apr-2014	Italy: Apulia, Presicce	39.886	18.266	
391	machaon	RVcoll.14-I381	1	MH418548	658 bp	-	-	-		-	-	-		24-May-2014	Italy: Tuscany, Talamone	42.575	11.14	
392	machaon	RVcoll.14-I514	1	MN141062	658 bp	-	-	-		-	-	-		06-Aug-2014	Italy: Liguria, Monte San Giovanni	44.326	9.388	
393	machaon	RVcoll.14-L182	1	MN143540	658 bp	-	-	-		-	-	-		01-Aug-2014	Italy: Emilia-Romagna, Bottegone	44.809	11.1	
394	machaon	RVcoll.08-H454	1	GU676652	658 bp	-	-	-		-	-	-	Munguira, Mig	05-Jun-2008	Spain: Comunidad de Madrid, Campo Real	40.358	-3.365	
395	machaon	RVcoll.08-H555	1	MN139567	658 bp	-	-	-		-	-	-	Garcia-Barros,	26-Apr-2008	Spain: Guadalajara, Castilla-La Mancha, Canredondo, La Alcarria	40.799	-2.507	
396	machaon	RVcoll.08-H627	1	GU676601	658 bp	-	-	-		-	-	-	Hernandez-Ro	02-May-2008	Spain: Andalusia, Cadiz, El Gastor	36.856	-5.339	
397	machaon	RVcoll.08-J326	1	GU676539	658 bp	-	-	-		-	-	-		30-Mar-2008	Spain: Aragon, Huesca, Barranco de Valcuerna, Candasnos	41.465	0.02	
398	machaon	RVcoll.08-J346	1	GU669682	658 bp	-	-	-		-	-	-		13-Apr-2008	Spain: Catalonia, Girona, Cantallops, Alt Emporda	42.423	-2.926	
399	machaon	RVcoll.08-J361	1	GU669689	658 bp	-	-	-		-	-	-		19-Apr-2008	Spain: Catalonia, Barcelona, Vallgrassa, Parc Natural del Garraf	41.284	1.876	
400	machaon	RVcoll.08-J745	1	HM901252	658 bp	-	-	-		-	-	-	S. Montagud, /	14-Jun-2008	Spain: Comunidad Valenciana, Valencia, Casa de Camineros, Tue	39.822	-1.13	
401	machaon	RVcoll.08-J830	1	GU675872	658 bp	-	-	-		-	-	-	F. Gonzalez	08-Aug-2008	Spain: Castilla y Leon, Leon, Mansilla de las Mulas	42.479	-5.547	
402	machaon	RVcoll.08-M338	1	HQ004884	658 bp	-	-	-		-	-	-		01-Jun-2008	Romania: Harghita, Transylvania, 1 Km E of Gheorgheni	46.743	25.664	
403	machaon	RVcoll.08-M392	1	HQ004885	658 bp	-	-	-		-	-	-		04-Jun-2008	Romania: Tulcea, Dobrogea, Macin Mts. (3 Km S of Jijila)	45.269	28.177	
404	machaon	RVcoll.08-M966	1	MN139169	658 bp	-	-	-		-	-	-		09-Jul-2008	France: Languedoc-Roussillon, Col de Puymorens	42.556	1.815	
405	machaon	RVcoll.08-P251	1	GU676002	658 bp	-	-	-		-	-	-		20-Jul-2008	Spain: Balearic Islands, Mallorca, Santa Maria	39.685	2.761	
406	machaon	RVcoll.08-P267	1	GU675999	658 bp	-	-	-		-	-	-		20-Jul-2008	Spain: Balearic Islands, Mallorca, S'Albufera	39.797	3.105	
407	machaon	RVcoll.08-P286	1	GU676000	658 bp	-	-	-		-	-	-		21-Jul-2008	Spain: Balearic Islands, Mallorca, Genova	39.57	2.592	
408	machaon	RVcoll.08-R265	1	GU669683	658 bp	-	-	-		-	-	-		02-Aug-2008	Spain: Catalonia, Lleida, Vilamos, Vall d`Aran	42.707	0.91	
409	machaon	RVcoll.08-R325	1	GU675856	658 bp	-	-	-		-	-	-		14-Oct-2008	Spain: Balearic Islands, Mallorca, Pic Tomir	39.837	2.922	
410	machaon	RVcoll.09-T105	1	HM901370	658 bp	-	-	-		-	-	-	Hernandez-Ro	26-Jul-2009	Spain: Castilla-La Mancha, Cuenca, Una	40.231	-1.96	
411	machaon	RVcoll.09-V419	1	HM901399	658 bp	-	-	-		-	-	-		16-Jul-2009	Spain: Andalusia, Granada, San Juan (Sierra Nevada)	37.094	-3.115	
412	machaon	RVcoll.09-X895	1	MN140953	658 bp	-	-	-		-	-	-		07-Jul-2009	Italy: Campania, Napoli, Capri Island	40.548	14.224	
413	machaon	RVcoll.09-X914	1	MN145140	658 bp	-	-	-		-	-	-			Italy: Salerno, Ottati	40.478	15.312	
414	machaon	RVcoll.09-X932	1	MH418821	658 bp	-	-	-		-	-	-		01-Sep-2009	Italy: Tuscany, Livorno, Monte Perone, Elba Island	42.773	10.188	
415	machaon	RVcoll.09-X936	1	MN140087	658 bp	-	-	-		-	-	-		15-Jun-2009	Italy: Lazio, Latina, Ponza Island	40.906	12.955	
416	machaon	RVcoll.09-X937.1	1	MN144727	658 bp	-	-	-		-	-	-		15-Jun-2009	Italy: Lazio, Latina, Ponza Island	40.906	12.955	
417	machaon	RVcoll.09-X938	1	MN145307	658 bp	-	-	-		-	-	-		15-Jun-2009	Italy: Lazio, Latina, Ponza Island	40.906	12.955	
418	machaon	RVcoll.10-B602	1	MN145188	658 bp	-	-	-		-	-	-		08-Aug-2010	France: Provence-Alpes-Cote d'Azur, Var, Domaine de la chasse	43.554	5.73	
419	machaon	RVcoll.10-C134	1	MN140123	658 bp	-	-	-		-	-	-		18-Aug-2010	France: Hautes Alpes, Rosans	44.39	5.49	
420	machaon	RVcoll.10-C576	1	MH418713	658 bp	-	-	-		-	-	-		10-Jun-2010	Italy: Grosseto, Argentario	42.428	11.159	
421	machaon	RVcoll.10-C608	1	MH420243	658 bp	-	-	-		-	-	-			Italy: Reggio Calabria, Reggio Calabria	38.122	15.675	
422	machaon	RVcoll.11-D330	1	MH418700	658 bp	-	-	-		-	-	-		13-Apr-2011	Italy: Sicily, Siracusa, Melilli	37.197	15.118	
423	machaon	RVcoll.11-D403	1	MH419860	658 bp	-	-	-		-	-	-		13-Apr-2011	Italy: Sicily, Sortino, Siracusa	37.158	15.049	
424	machaon	RVcoll.11-D414	1	MH419534	658 bp	-	-	-		-	-	-		14-Apr-2011	Italy: Sicily, Messina, Larderia (Messina)	38.13	15.496	
425	machaon	RVcoll.11-D492	1	MH418766	658 bp	-	-	-		-	-	-		18-Apr-2011	Italy: Calabria, Vibo Valentia, Briatico	38.722	16.013	
426	machaon	RVcoll.11-D873	1	MN141530	658 bp	-	-	-		-	-	-	Vila, R., Monta	14-May-2011	Spain: Malaga, Andalucia, Frigiliana	36.803	-3.93	
427	machaon	RVcoll.11-E119	1	MH419173	658 bp	-	-	-		-	-	-		20-May-2011	Malta: Xaghra, Gozo Island, Xaghra	36.042	14.274	

0	Spcies	Sample ID	Photos	COI Accession	lengh	Ca-ATPase	CAD	28S	Phylogeny	16S	wsp	ftsZ	Collectors	Collection Date		Lat	Lon	Elev
428	machaon	RVcoll.11-E120	1	MH419011	658 bp	-	-	-		-	-	-		20-May-2011	Malta: Xaghra, Gozo Island, Xaghra, Gozo Island	36.042	14.274	
429	machaon	RVcoll.11-E121	1	MH419932	658 bp	-	-	-		-	-	-		20-May-2011	Malta: Xaghra, Gozo Island, Xaghra, Gozo Island	36.042	14.274	
430	machaon	RVcoll.11-E122	1	MH420063	658 bp	-	-	-		-	-	-		20-May-2011	Malta: Xaghra, Gozo Island, Xaghra, Gozo Island	36.042	14.274	
431	machaon	RVcoll.11-E302	1	MH419665	658 bp	-	-	-		-	-	-		14-May-2011	Italy: Sardinia, Cagliari, Faro Sant Elia	39.188	9.146	
432	machaon	RVcoll.11-E398	1	MH419848	658 bp	-	-	-		-	-	-		16-May-2011	Italy: Sardinia, Ogliastra, Sardinia, Gairo, Sardinia Island	39.856	9.49	
433	machaon	RVcoll.11-E673	1	MH419019	658 bp	-	-	-		-	-	-		23-May-2011	Italy: Sardinia, Olbia-Tempio, Acapulco	41.186	9.377	
434	machaon	RVcoll.11-F403	1	PQ884874	658 bp	-	-	-		-	-	-	Vila, R., Dinca, V	27-Jun-2011	Morocco: Tangier-Tetouan Region, Chaouen, 5 km NE Amesoug	34.968	-4.688	1531
435	machaon	RVcoll.11-G020	0	PQ884717	658 bp	-	-	-		-	-	-	Vila, R., Dinca, V	02-Jul-2011	Morocco: Fes-Meknes, Fes, Annoceur	33.662	-4.854	1410
436	machaon	RVcoll.11-H279	1	MH418971	658 bp	-	-	-		-	-	-		15-Jul-2011	Italy: Lazio, Viterbo, Monte Romano	42.27	11.95	
437	machaon	RVcoll.11-H554	1	MH419910	658 bp	-	-	-		-	-	-		08-Jun-2011	Italy: Sicily, Palermo, San Martino alle Scale	38.312	13.341	
438	machaon	RVcoll.11-H602	1	MH418642	658 bp	-	-	-		-	-	-		08-Jun-2011	Italy: Sicily, Palermo, Cinisi, Sicily	38.226	13.222	
439	machaon	RVcoll.11-H627	1	MH419762	658 bp	-	-	-		-	-	-		09-Jun-2011	Italy: Sicily, Agrigento, Isola di Lampedusa, Sicilian Islands	35.52	12.55	
440	machaon	RVcoll.11-H628	1	MH418969	658 bp	-	-	-		-	-	-		09-Jun-2011	Italy: Sicily, Agrigento, Isola di Lampedusa, Sicilian Islands	35.52	12.55	
441	machaon	RVcoll.11-H629	1	MH418775	658 bp	-	-	-		-	-	-		09-Jun-2011	Italy: Sicily, Agrigento, Isola di Lampedusa, Sicilian Islands	35.52	12.55	
442	machaon	RVcoll.11-H630	1	MH419425	658 bp	-	-	-		-	-	-		09-Jun-2011	Italy: Sicily, Agrigento, Isola di Lampedusa, Sicilian Islands	35.52	12.55	
443	machaon	RVcoll.11-H791	1	MH419718	658 bp	-	-	-		-	-	-		14-Jun-2011	Italy: Sicily, Messina, Isola di Vulcano, Sicilian Islands	38.39	14.97	
444	machaon	RVcoll.11-H792	1	MH419314	658 bp	-	-	-		-	-	-		14-Jun-2011	Italy: Sicily, Messina, Isola di Vulcano, Sicilian Islands	38.39	14.97	
445	machaon	RVcoll.11-H866	1	MH418929	658 bp	-	-	-		-	-	-		15-Jun-2011	Italy: Sicily, Messina, Isola di Salina, Sicilian Islands	38.56	14.83	
446	machaon	RVcoll.11-J350	1	KP870927	658 bp	-	-	-		-	-	-		10-Sep-2011	Romania: Muntenia, Buzau, Breaza, Dealul Istrita	45.103	26.541	
447	machaon	RVcoll.11-J399	1	KP870574	658 bp	-	-	-		-	-	-		11-Sep-2011	Romania: Muntenia, Buzau, Breaza, Dealul Istrita	45.103	26.541	
448	machaon	RVcoll.11-J917	1	MH419672	658 bp	-	-	-		-	-	-	Khaldi, Moura	21-May-2008	Algeria: M`sila, Oum Mrazem	35.6	3.95	664
449	machaon	RVcoll.11-Y005	1	MH420096	658 bp	-	-	-		-	-	-		31-Mar-2011	Italy: Tuscany, Livorno, San Piero, Elba Island	42.752	10.205	
450	machaon	RVcoll.12-L763	1	PQ884795	658 bp	-	-	-		-	-	-	Voda, Raluca	16-May-2012	Morocco: Tangier-Tetouan Region, Assilah, Ezzammige	35.702	-5.629	430
451	machaon	Rvcoll.12-L879	1	KP870235	658 bp	-	-	-		-	-	-	Garcia, Oscar	15-May-2012	Spain: Balearic Islands, Menorca, Rafael Vell, Mao, Menorca	39.91	4.19	
452	machaon	Rvcoll.12-L882	1	KP870273	658 bp	-	-	-		-	-	-	Garcia, Oscar	17-May-2012	Spain: Balearic Islands, Menorca, Turdonell de Dalt, Mao, Menor	39.96	4.22	
453	machaon	Rvcoll.12-L899	1	KP870584	658 bp	-	-	-		-	-	-	Garcia, Oscar	07-Jun-2012	Spain: Balearic Islands, Menorca, Son Gornes, Ferreries, Menorca	39.98	4	
454	machaon	RVcoll.12-M179	0	MW503162	658 bp	-	-	-		-	-	-		06-Apr-2012	Greece: Crete, Phaestos	35.06	24.81	
455	machaon	RVcoll.12-M286	1	MH418625	658 bp	-	-	-		-	-	-		05-May-2012	Italy: Sicily, Messina, Lipari Island	38.485	14.95	
456	machaon	Rvcoll.12-M963	1	KP870541	658 bp	-	-	-		-	-	-		07-Jul-2012	Spain: Balearic Islands, Ibiza, Santa Eularia des Riu, Ibiza	38.998	1.511	
457	machaon	RVcoll.12-N336	1	MH419661	658 bp	-	-	-		-	-	-	Dapporto, L. &	29-May-2012	Tunisia: Kasserine, Kasserine	35.053	9.257	484
458	machaon	RVcoll.12-N337	1	MH420346	658 bp	-	-	-		-	-	-	Dapporto, L. &	29-May-2012	Tunisia: Kasserine, Kasserine	35.053	9.257	484
459	machaon	RVcoll.12-N402	1	MH418983	658 bp	-	-	-		-	-	-	Dapporto, L. &	30-May-2012	Tunisia: Gafsa, Gafsa Mountain	34.337	9.051	331
460	machaon	RVcoll.12-N861	1	MW501266	658 bp	-	-	-		-	-	-	Sylvain Cuvelie	08-Jun-2012	Greece: Sigri-Faneromeni,,	39.225	25.857	
461	machaon	RVcoll.12-O048	1	MH419670	658 bp	-	-	-		-	-	-		10-Jun-2012	Italy: Tuscany, Grosseto, Argentario	42.428	11.159	
462	machaon	RVcoll.12-O049	1	MH419791	658 bp	-	-	-		-	-	-		10-Jun-2012	Italy: Tuscany, Grosseto, Argentario	42.428	11.159	
463	machaon	RVcoll.12-O197	1	MH419266	658 bp	-	-	-		-	-	-		17-Jun-2012	France: Corsica, Corse-du-Sud, Monicia d`Aullene, Corsica Islan	41.484	9	
464	machaon	RVcoll.12-O221	1	MH420311	658 bp	-	-	-		-	-	-		17-Jun-2012	France: Corsica, Corse-du-Sud, Fautea, Corsica Island	41.7	9.383	
465	machaon	RVcoll.12-O397	1	MN140511	658 bp	-	-	-		-	-	-		21-Jun-2012	Italy: Sardinia, Carbonia-Iglesias, Isola di San Pietro	39.139	8.278	
466	machaon	RVcoll.12-O552	1	MN144325	658 bp	-	-	-		-	-	-		24-Jun-2012	Italy: Sardinia, Ogliastra, Tortoli, Sardinia Island	39.922	9.631	
467	machaon	RVcoll.12-O597	1	MH419548	658 bp	-	-	-		-	-	-		24-Jun-2012	Italy: Sardinia, Nuoro, Fonni, Sardinia Island	40.037	9.257	
468	machaon	RVcoll.12-P221	1	MN141755	658 bp	-	-	-		-	-	-		26-Jul-2012	France: Occitanie, Languedoc-Roussillon, Aude' Caves	42.925	2.958	
469	machaon	RVcoll.12-P290	1	MN142349	658 bp	-	-	-		-	-	-			France: Occitanie, Languedoc-Roussillon, Aude' Gruissan	43.133	3.061	
470	machaon	RVcoll.12-P948	1	MN142863	658 bp	-	-	-		-	-	-		04-Aug-2012	France: Provence-Alpes-Cote d'Azur, Hyeres Islands, Levant Isla	43.02	6.434	
471	machaon	RVcoll.12-Q338	1	MN144253	658 bp	-	-	-		-	-	-		08-Aug-2012	France: Provence-Alpes-Cote d'Azur, Alpes-Maritimes, Valdeblo	44.069	7.208	
472	machaon	RVcoll.12-Q790	1	MH418756	658 bp	-	-	-		-	-	-		30-Jul-2012	Italy: Potenza, Pollino, Piano Ruggio, S Appennines	39.93	16.17	
473	machaon	RVcoll.12-R196	1	MN140690	658 bp	-	-	-		-	-	-		05-Aug-2012	Italy: Lazio, Latina, Aurunci, Monte S. Angelo, Italian coast hills	41.3	13.617	
474	machaon	RVcoll.13-S385	1	MN142462	658 bp	-	-	-		-	-	-		04-Jun-2013	Spain: Illes Balears, Sal Rossa, Sant Jordi, Ibiza	38.872	1.398	
475	machaon	RVcoll.13-S575	1	MN143257	658 bp	-	-	-		-	-	-		09-Jun-2013	Italy: Foggia, Molise, San Domino island	42.111	15.486	
476	machaon	RVcoll.13-T735	1	MH418615	658 bp	-	-	-		-	-	-		30-Apr-2013	Italy: Sardinia, Cuglieri	40.146	8.544	
477	machaon	RVcoll.13-T773	1	MH418599	658 bp	-	-	-		-	-	-			Italy: Tuscany, Cavallaia	43.77	10.8	
478	machaon	RVcoll.14-A553	1	MH419943	658 bp	-	-	-		-	-	-			Italy: Lucca, Monte Tamburalow	44.093	10.248	
479	machaon	RVcoll.14-C930	1	MW499776	658 bp	-	-	-		-	-	-		04-Aug-2013	Bulgaria: Kresna Gorges	41.764	23.156	
480	machaon	RVcoll.14-E178	1	MH419461	658 bp	-	-	-		-	-	-			Italy: Livorno, Toscana, Piombino, Fiorentina	42.968	10.531	
481	machaon	RVcoll.14-F753	1	MW502609	658 bp	-	-	-		-	-	-		05-Jul-2014	Greece: Platanos	39.132	22.837	
482	machaon	RVcoll.14-F912	1	MW502993	658 bp	-	-	-		-	-	-		06-Jul-2014	Greece: Chelmos Mt., Gaidurrorachi	38.031	22.218	
483	machaon	RVcoll.14-G615	1	MW502421	658 bp	-	-	-		-	-	-		15-Jul-2014	Greece: Romia	39.221	20.84	
484	machaon	RVcoll.14-G673	1	MW499722	614 bp	-	-	-		-	-	-		17-Jul-2014	Greece: Drosopigi	40.233	20.955	
485	machaon	RVcoll.14-H384	1	MW500205	658 bp	-	-	-		-	-	-		24-May-2014	Greece: Sterea Ellada, Arachova	38.477	22.597	
486	machaon	RVcoll.14-H617	1	MW500042	658 bp	-	-	-		-	-	-		27-May-2014	Greece: Peloponnesos, Parorio gorges	37.06	22.38	
487	machaon	RVcoll.14-H959	1	MN140067	658 bp	-	-	-		-	-	-		16-Jul-2014	France: Figeac	44.626	2.053	
488	machaon	RVcoll.14-I471	1	MN145425	658 bp	-	-	-		-	-	-		23-Jun-2014	Italy: Lazio, Monte Scalambra	41.849	13.087	

0	Spcies	Sample ID	Photos	COI Accession	lengh	Ca-ATPase	CAD	28S	Phylogeny	*16S*	*wsp*	*ftsZ*	Collectors	Collection Date		Lat	Lon	Elev
489	machaon	RVcoll.14-I993	1	MN138654	658 bp	-	-	-		-	-	-		08-May-2014	Italy: Sardinia, Olbia-Tempio, La Maddalena, Maddalena archipela	41.234	9.409	
490	machaon	RVcoll.14-J356	1	MH418659	658 bp	-	-	-		-	-	-		02-Jun-2014	Italy: Livorno, Pianosa	42.59	10.09	
491	machaon	RVcoll.14-J357	1	MH418596	658 bp	-	-	-		-	-	-		02-Jun-2014	Italy: Livorno, Pianosa	42.59	10.09	
492	machaon	RVcoll.14-J421	1	MH418943	658 bp	-	-	-		-	-	-		03-Jun-2014	Italy: Grosseto, Giglio Castello	42.36	10.9	
493	machaon	RVcoll.14-K030	1	MN143937	658 bp	-	-	-		-	-	-		22-May-2014	Switzerland: Leuk, Susten, Leuk distr.	46.306	7.671	
494	machaon	RVcoll.14-L054	1	MH419234	658 bp	-	-	-		-	-	-	Ait Hammou M	10-May-2009	Algeria: Tiaret, Tafrent (Meriama)	35.28	0.92	829
495	machaon	RVcoll.14-L131	1	MH418822	658 bp	-	-	-		-	-	-	Khaldi, Moura	10-Mar-2013	Algeria: Pole universitaire de M`sila	35.75	4.55	511
496	machaon	RVcoll.14-L138	1	MH418993	658 bp	-	-	-		-	-	-	Rebbas, Khella	18-Apr-2014	Algeria: Bejaia, chenaie verte - Trouna	36.5	4.76	1004
497	machaon	RVcoll.14-N657	1	MH418538	658 bp	-	-	-		-	-	-	Voda, R. and D	01-Oct-2014	Italy: Messina, Salina	38.57	14.83	
498	machaon	RVcoll.LD-2381	1	MH420159	658 bp	-	-	-		-	-	-			Italy: Palermo, Isola di Ustica	38.709	13.179	
499	machaon	RVcoll.LD-2673	1	MH419240	658 bp	-	-	-		-	-	-			Italy: Grosseto, Isola del Giglio, Castello	42.364	10.9	
500	machaon	RVcoll.LD-2914	1	MN139923	658 bp	-	-	-		-	-	-			Italy: Sassari, Isola dell`Asinara	41.061	8.271	
501	machaon	RVcoll.LD-3077	1	MN145339	658 bp	-	-	-		-	-	-			Italy: Salerno, Isola di Capri	40.548	14.224	
502	machaon	RVcoll.LD-3078	1	MN141479	658 bp	-	-	-		-	-	-			Italy: Salerno, Isola di Capri	40.548	14.224	
503	machaon	RVcoll07D803	1	PQ884919	658 bp	-	-	-		-	-	-	Vila, Roger	07-Jul-2007	Italy: Latium	41.767	12.315	10
504	machaon	RVcoll13S388	1	PQ884887	658 bp	-	-	-		-	-	-	Vila, Roger	04-Jun-2013	Spain: Balearic Islands	38.872	1.398	7
505	machaon	RVcoll14A078	0	MN140872	658 bp	-	-	-		-	-	-			Italy: Monte Faito	40.67	14.473	
506	machaon	RVcoll14F023	1	MW500786	658 bp	-	-	-		-	-	-		27-Jun-2014	Serbia: Divcibare, Mt. Maljen	44.1219	20.0153	
507	machaon	RVcoll14I971	1	PQ884952	658 bp	-	-	-		-	-	-		08-May-2014	Italy: Sardinia	41.163	9.315	23.666626
508	machaon	RVcoll14N621	1	PQ884981	658 bp	-	-	-		-	-	-	Voda, R. and L	30-Sep-2014	Italy	38.38	14.97	350
509	machaon	RVcoll15C977	0	PQ884801	658 bp	-	-	-		-	-	-			Morocco: Tizi-n-Bachkoum, N Tazenakht	30.69	-7.27	1600
510	machaon	RVcoll15G486	1	MW502226	658 bp	-	-	-		-	-	-			Switzerland: Brig, Schallberg	46.736782	8.286929	
511	machaon	RVcoll15H201	1	MW503151	658 bp	-	-	-		-	-	-			Switzerland: Interlaken, Bort	46.736782	8.286929	
512	machaon	RVcoll15I902	1	MW503139	633 bp	-	-	-		-	-	-			Liechtenstein: north Liechtenstein	47.146275	9.547675	
513	machaon	RVcoll15J461	1	MW501969	658 bp	-	-	-		-	-	-			France: Hautes-Alpes, Prorel	46.64237	2.1940236	
514	machaon	RVcoll15P011	1	MW501635	658 bp	-	-	-		-	-	-		04-Jul-2015	Belarus: Minsk city, Stsiklyova	53.88	27.71	
515	machaon	RVcoll15P661	1	PQ884753	658 bp	-	-	-		-	-	-	Helena Romo	21-Aug-2015	Spain: Aragon, Ejea de los Caballeros, Zaragoza	42.125607	-1.116992	336
516	machaon	RVcoll16I684	1	MW503618	640 bp	-	-	-		-	-	-		01-Aug-2016	Poland: Kuyavia-Pomerania, Niewierz	53.2424	19.2924	
517	machaon	RVcoll16I789	1	MW502920	658 bp	-	-	-		-	-	-		15-Aug-2016	Germany: Thuringia, Thierbach	50.4912	11.5558	
518	machaon	RVcoll16J251	1	MW500338	658 bp	-	-	-		-	-	-		14-Aug-2016	Germany: Bavaria, Liederberg	48.8552	10.9242	
519	machaon	RVcoll16J798	0	MW501166	658 bp	-	-	-		-	-	-		04-Sep-2016	Greece: Nas	37.6198	26.0623	
520	machaon	RVcoll16L020	0	MH419614	658 bp	-	-	-		-	-	-		16-Sep-2016	France: Cannella	41.794	9.395	
521	machaon	RVcoll17A026	0	MW502976	658 bp	-	-	-		-	-	-			Malta: Luqa	35.8536	14.494	
522	machaon	RVcoll17A394	1	PQ885024	658 bp	-	-	-		-	-	-	Aurelien Gaun	08-Apr-2017	France: Pyrenees-Atlantiques, Briscous	43.44528	-1.345833	70
523	machaon	RVcoll17B815	1	PQ884866	658 bp	-	-	-		-	-	-	Joan Carles Hi	17-May-2017	Portugal: Monchique, Faro	37.306713	-8.533874	720
524	machaon	RVcoll17B906	1	PQ884870	658 bp	-	-	-		-	-	-	Joan Carles Hi	19-May-2017	Spain: Extremadura, Monesterio, Badajoz	38.0615	-6.27032	873
525	machaon	RVcoll19C231	1	PQ884775	658 bp	-	-	-		-	-	-	Dapporto, Leo	16-Apr-2016	Italy: Nirano	44.495	10.825	
526	machaon	SMcoll.270708ZB68	0	GU675943	658 bp	-	-	-		-	-	-	Garcia, F.	20-Aug-2009	Spain: Castilla-La Mancha, Albacete, Huelga-Utrera, Pontones	38.167	-2.609	
527	machaon	SMcoll.311007WR55	0	GU675945	658 bp	-	-	-		-	-	-	S. Montagud	15-Mar-2008	Spain: Comunidad Valenciana, Valencia, Montecanada, Paterna	39.531	-0.46	
528	machaon	SWC-09-5005	0	GU696031	658 bp	-	-	-		-	-	-		03-May-2009	South Korea	36.739	127.455	
529	machaon	TLMF Lep 09113	1	KM572218	658 bp	-	-	-		-	-	-	Aistleitner	01-Jun-2002	Austria: Vorarlberg, Kalkalpen, Innerbraz NE: Gafreu	47.151	9.916	
530	machaon	TLMF Lep 13866	1	MN140360	658 bp	-	-	-		-	-	-	Erlebach S.	10-Apr-2005	Austria: Tyrol, Nordtirol, Mittelberg	46.956	10.875	
531	machaon	TLMF Lep 14022	1	MN142237	658 bp	-	-	-		-	-	-	Otter A.	14-Jul-2005	Austria: Tyrol, Nordtirol, Umg. Innsbruck' Vill S	47.232	11.4	850
532	machaon	TLMF Lep 14174	1	MN140291	658 bp	-	-	-		-	-	-	Niederkofler K	22-May-2006	Italy: South Tyrol, Suedtirol, Aufhofen	46.814	11.946	849
533	machaon	TLMF Lep 14175	1	MN139575	658 bp	-	-	-		-	-	-	Niederkofler K	21-Jul-2006	Italy: South Tyrol, Suedtirol, Baerental, Gais	46.839	11.961	900
534	machaon	TLMF Lep 19628	1	MN141185	658 bp	-	-	-		-	-	-		02-Jul-2013	Austria: Steiermark, Hochrettelstein	47.425	14.233	2220
535	machaon	TLMF Lep 21125	1	MN144388	658 bp	-	-	-		-	-	-	Buchner	26-Jun-2016	Austria: Bad Fischau Trockenhang	47.833	16.167	300
536	machaon	UAM:Ento:19012	1	KU875741	658 bp	-	-	-		-	-	-	H. Williams	12-Jul-2003	United States: Alaska, Bettles	66.917	-151.513	180
537	machaon	VR0238	1	PQ884863	643 bp	-	-	-		-	-	-	Voda Raluca	03-Jul-2017	Italy: Piedmont, Devero	46.311311	8.259717	
538	machaon	ZMBSU-02123	2	MW502950	658 bp	-	-	-		-	-	-	E. M. Setrakova	16-Jul-2010	Belarus: Minsk Region, neighborhood vill. Schemyislitsa	53.467915	27.964252	
539	neosaharae	MCH001	0	PQ884772	658 bp	PQ885090	PQ885120	PQ885053	x	negative	negative	negative		24-May-2019	Morocco: Anti-Atlas, Djebel, Siroua, N. Taliouine	30.71	-7.62	1900
540	neosaharae	MCH002	0	PQ884960	658 bp	PQ885106	PQ885129	PQ885072	x	negative	negative	negative			Morocco: Anti-Atlas, Djebel, Siroua, Asagaour	30.72	-7.76	1900
541	neosaharae	MCH003	0	PQ884973	658 bp	PQ885110	PQ885133	PQ885076	x	negative	negative	negative		30-Sep-2018	Morocco: Anti-Atlas, Djebel, Askaoun	30.74	-7.8	1900
542	neosaharae	MCH004	0	PQ884732	658 bp	PQ885084	PQ885119	PQ885051	x	negative	negative	negative		15-Jul-2018	Morocco: Anti-Atlas, Djebel, Siroua, N. Taliouine	30.54	-7.92	2100
543	neosaharae	MCH005	0	PQ884852	658 bp	PQ885094	PQ885122	PQ885058	x	negative	negative	negative		24-May-2019	Morocco: Anti-Atlas, Djebel, Siroua	30.71	-7.62	1900
544	neosaharae	MCH006	0	PQ884813	658 bp	-	-	-		negative	negative	negative		25-Mar-2019	Morocco: Moyen Atlas, NE Azrou	33.46	-5.21	
545	polyxenes	NVG-2481	0	MW136699	658 bp	-	-	-		-	-	-	Nick V. Grishin	01-Sep-13	United States: California, Colorado Desert	34	-115.34	
546	polyxenes	NVG-2483	0	MW136700	658 bp	-	-	-		-	-	-	Nick V. Grishin	15-Aug-13	United States: California, Colorado Desert	34	-115.34	
547	polyxenes	04-SRNP-36224		GU163949	658 bp	-	-	-	x	-	-	-			Costa Rica			
548	saharae	BC ZSM Lep 89953	0	PQ884934	658 bp	-	-	-		-	-	-			Morocco: Guelmim	28.988366	-10.05275	
549	saharae	DNAcdb0113	1	PQ885039	658 bp	-	-	-		-	-	-		31-Jul-1999	Yemen: Ibb, Jabal Bada`an	15.783	44.083	1700

0	Spcies	Sample ID	Photos	COI Accession	lengh	Ca-ATPase	CAD	28S	Phylogeny	*16S*	*wsp*	*ftsZ*	Collectors	Collection Date		Lat	Lon	Elev
550	saharae	MCH007	0	PQ884873	658 bp	PQ885096	PQ885123	PQ885061	x	negative	negative	negative		03-Apr-2019	Algeria: Biskra, El Kantara	35.25	5.8	650
551	saharae	MCH008	0	PQ884947	658 bp	PQ885104	PQ885127	PQ885069	x	negative	negative	negative		03-Apr-2019	Algeria: Biskra, El Kantara	35.25	5.8	650
552	saharae	MCH009	0	PQ884999	658 bp	PQ885113	PQ885136	PQ885079	x	negative	negative	negative		01-Apr-2019	Algeria: Biskra, El Kantara	35.25	5.8	750
553	saharae	MCH010	0	PQ885010	658 bp	-	-	-		negative	negative	negative		01-Apr-2019	Algeria: Biskra, El Kantara	35.25	5.8	750
554	saharae	MCH011	0	PQ884974	658 bp	-	-	-		negative	negative	negative		01-Apr-2019	Algeria: Biskra, El Kantara	35.25	5.8	750
555	saharae	MCH012	0	PQ884995	658 bp	-	-	-		negative	negative	negative		04-Apr-2019	Algeria: Biskra, Goufi	36.97	6.43	350
556	saharae	MCH013	0	PQ885034	658 bp	PQ885116	PQ885138	PQ885081	x	negative	negative	negative		08-Apr-2019	Tunisia: Gafsa, Sened	34.55	9.25	450
557	saharae	MCH014	0	PQ884968	658 bp	PQ885109	PQ885132	PQ885075	x	negative	negative	negative		08-Apr-2019	Tunisia: Gafsa, El Guitare	34.4	9.1	525
558	saharae	MCH019	0	PQ885012	658 bp	-	-	-		-	-	-		18-May-1980	Yemen: Jabal Bada	13.6	45.4	2900
559	saharae	PAP117	0	PQ884767	658 bp	-	-	-		-	-	-			Israel			
560	saharae	PAP118	0	PQ884782	658 bp	-	-	-		-	-	-			Israel			
561	saharae	PAP119	0	PQ884997	658 bp	-	-	-		-	-	-			Israel			
562	saharae	PAP121	0	PQ884824	658 bp	-	-	-		-	-	-			Israel: Jerusalem	31.768319	35.21371	
563	saharae	PAP122	0	PQ884814	658 bp	-	-	-		-	-	-			Israel: Jerusalem	31.768319	35.21371	
564	saharae	RVcoll.14-O164	2	PQ884896	538 bp	-	-	-		-	-	-		03-May-2002	Tunisia: Gafsa, 5 km. NE of Redeyef	34.42	8.19	250
565	saharae	RVcoll.14-O165	2	PQ884906	658 bp	-	-	-		-	-	-		12-May-2004	Tunisia: Gafsa, 5 km. NE of Redeyef	34.42	8.19	250
566	saharae	RVcoll.14-O166	2	PQ884754	658 bp	-	-	-		-	-	-		07-Oct-1997	Tunisia: Kairouan, Menzel Mehiri	35.42	9.85	200
567	saharae	RVcoll.14-O167	2	PQ884854	658 bp	-	-	-		-	-	-		13-Nov-1997	Tunisia: Kairouan, Menzel Mehiri	35.42	9.85	200
568	saharae	RVcoll14V799	0	PQ884939	658 bp	-	-	-		-	-	-			Morocco: Tafraoute, Col du Kerdous, on the ridge S of the ho	29.546	-9.339	1250
569	saharae	RVcoll15C978	0	PQ884879	658 bp	-	-	-		-	-	-			Morocco: Adrar Akdal, N Ida-Ouaassam	29.54	-9.1	1350
570	saharae	RVcoll15C979	0	PQ884936	658 bp	-	-	-		-	-	-			Morocco: Adrar Akdal, N Ida-Ouaassam	29.54	-9.1	1350
571	saharae	RVcoll15C987	0	PQ884764	658 bp	-	-	-		-	-	-			Morocco: Tizi-n-Bachkoum, N Tazenakht	30.69	-7.27	1600
572	saharae	RVcoll15C988	0	PQ885036	658 bp	-	-	-		-	-	-			Morocco: Env. Ait-Abdallah, djebel Akoumbi, E Tafraoute	29.82	-8.78	1800
573	saharae	RVcoll15C989	0	PQ884986	658 bp	-	-	-		-	-	-			Morocco: Env. Ait-Abdallah, djebel Akoumbi, E Tafraoute	29.82	-8.78	1800
574	ulysses	USNM:ENT:00645977		HQ570589	658 bp	-	-	-	x	-	-	-			Papua New Guinea			
575	verityi	AC-SP1738	2	PQ884992	658 bp	-	-	-		-	-	-		01-Jan-2009	China: Yunnan, S Yunnan, Mohan	21.236	101.719	
576	verityi	GCB01	2	PP865967	658 bp	-	-	-		-	-	-		15-Aug-2014	Vietnam: N. Vietnam, Dongvan	23.225	105.243	1600
577	verityi	PAP049	1	PQ884933	658 bp	PQ885102	PQ885126	PQ885067	x	negative	negative	negative		15-Jun-2019	Vietnam: Yen Bai, Yen Bai	21.7	104.9	
578	verityi	PAP060	1	PQ884914	658 bp	PQ885100	PQ885124	PQ885065	x	negative	negative	negative		06-Jul-2020	China: Yunnan, Dali area, Fengyi town	25.6	100.3	2000
579	verityi	PAP080	1	PQ884929	658 bp	-	-	-		negative	negative	negative		15-Jun-2019	Vietnam: Yen Bai	21.7	104.9	
580	xuthus	not given	0	AF043999	2,291 bp	-	-	-	x	-	-	-	M. Taguchi		Japan: Tokyo	35.676422	139.650027	
581	xuthus	not given	0	MN727324	658 bp	-	-	-		-	-	-			China	28.827146	112.694868	
582	zelicaon	CAS:NVG-3302	0	MW136703	658 bp	-	-	-		-	-	-	Nick V. Grishin		United States: California, Colorado Desert	34	-115.34	
583	zelicaon	NVG-2482	0	MW136708	658 bp	-	-	-		-	-	-			United States	32.56	-116.98	
584	zelicaon	10BBCLP-0287		JF841326	658 bp	-	-	-	x	-	-	-			Canada			
	machaon	Pap103		PQ884841	287 bp	-	-	-		-	-	-	K. Hojgaard	15-Jul-1996	United Kingdom: England, Norfolk, Norfolk Broads	52.689	1.609	
