# Supplementary material for: Integrative analyses reveal the evolution of the Old World Swallowtail in the Palearctic
Source: PLoS One. 2026 Jul 8;21(7):e0343793. doi: 10.1371/journal.pone.0343793 (PMC13345299; doi:10.1371/journal.pone.0343793)
Supplement: S2 Table — (PDF) [file pone.0343793.s002.pdf]

S2 Table. Primers combinations used in this study for COI gene fragment amplification.

Primer Pair	Fragment Length (bp)	Positions of the DNA Barcode	Reference
LepF (ATTCAACCAATCATAAAGATATTGG) / LepR (TAAACTTCTGGATGTCCAAAAAATCA)	658	1-658	Hajibabaei et al. 2006
LCO1490 (GGTCAACAAATCATAAAGATATTGG) / HCO2198 (TAAACTTCAGGGTGACCAAAAAATCA)	658	1-658	Folmer et al. 1994
LepF (ATTCAACCAATCATAAAGATATTGG) / MH-MR1 (CCTGTTCCAGCTCCATTTTC)	307	1-307	Hajibabaei et al. 2006/ Lukhtanov et al. 2009
LCO1490 (GGTCAACAAATCATAAAGATATTGG) / MH-MR1 (CCTGTTCCAGCTCCATTTTC)	307	1-307	Folmer et al. 1994 / Lukhtanov et al. 2009
MH-MF1 (GCTTTCCCACGA ATAAATAATA) / LepR (TAAACTTCTGGATGTCCAAAAAATCA)	407	251-658	Lukhtanov et al. 2009/ Hajibabaei et al. 2006
MH-MF1 (GCTTTCCCACGA ATAAATAATA) / HCO2198 (TAAACTTCAGGGTGACCAAAAAATCA)	407	251-658	Lukhtanov et al. 2009/ Folmer et al. 1994
LepF (ATTCAACCAATCATAAAGATATTGG) / mch01R (AGGTATAACYATAAAAAAAATTAT)	145	1-145	Hajibabaei et al. 2006/ Present study
LCO1490 (GGTCAACAAATCATAAAGATATTGG) / mch01R (AGGTATAACYATAAAAAAAATTAT)	145	1-145	Folmer et al. 1994 / Present study
mch02F (ATTGGRGAYGATCAAATTTATAAT) / mch02R (TCAAAATCTYATATTATTTATTCG)	120	119-238	Present study
mch03F (TTATAATTGGRGGATTTGGRAATTG) / mch03R (AGRGGRGGRTAAAYAGTTCAACC)	130	196-325	Present study
mch04F (AGAATAATTGTRGAAAATGG) / mch04R (GTTGTRATRAARTTAATTGCYCC)	114	316-430	Present study
mch05F (ATTTTTTCYTTRCATTTAGCAGG) / mcho5R (GTTAATARYATAGTAATAGCTCC)	148	415-562	Present study
mch06F (TATTTGTTTGAGCTGTTGGAATTAC) / LepR (TAAACTTCTGGATGTCCAAAAAATCA)	133	526-658	Present study/ Hajibabaei et al. 2006
mch06F (TATTTGTTTGAGCTGTTGGAATTAC) / HCO2198 (TAAACTTCAGGGTGACCAAAAAATCA)	133	526-658	Present study/ Folmer et al. 1994
