# Supplementary figures and images for: Integrative analyses reveal the evolution of the Old World Swallowtail in the Palearctic (part 2 of 3)
Source: PLoS One. 2026 Jul 8;21(7):e0343793. doi: 10.1371/journal.pone.0343793 (PMC13345299; doi:10.1371/journal.pone.0343793)

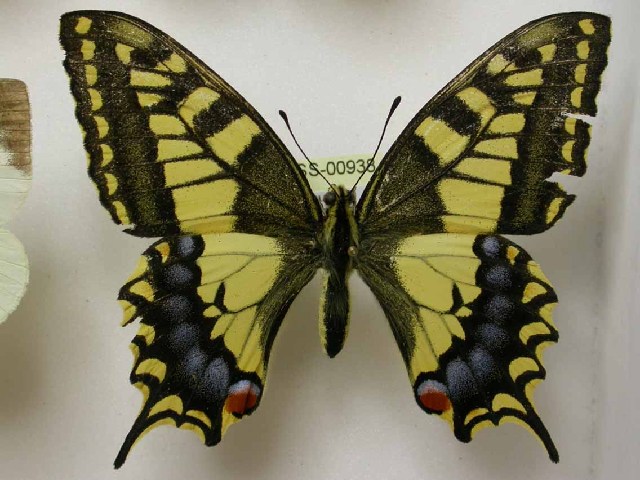

Supplement: S3 Fig — (ZIP) [file pone.0343793.s003.zip › S3/LEP-SS-00938.jpg]

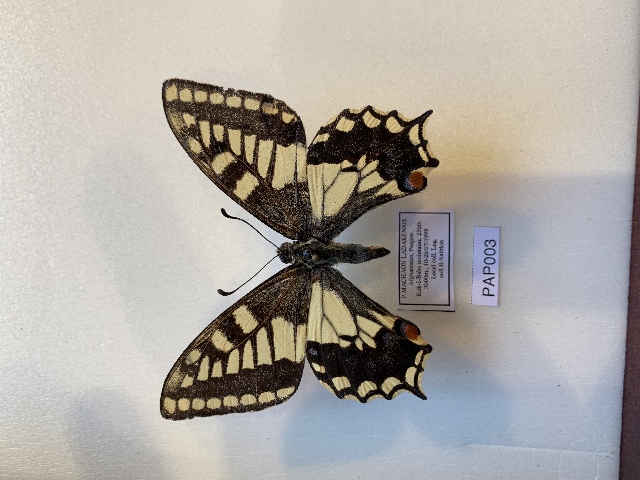

Supplement: S3 Fig — (ZIP) [file pone.0343793.s003.zip › S3/PAP003.jpeg]

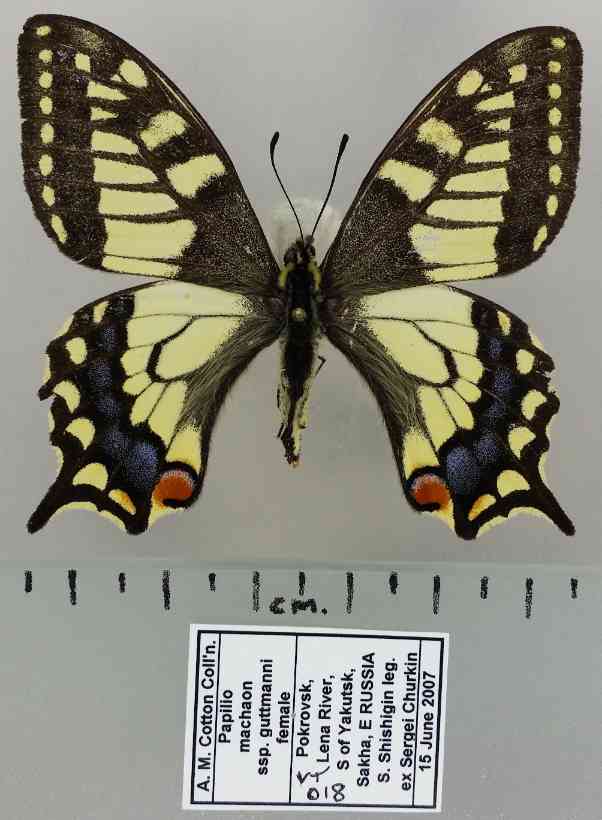

Supplement: S3 Fig — (ZIP) [file pone.0343793.s003.zip › S3/AC-VT018-D copy.jpg]

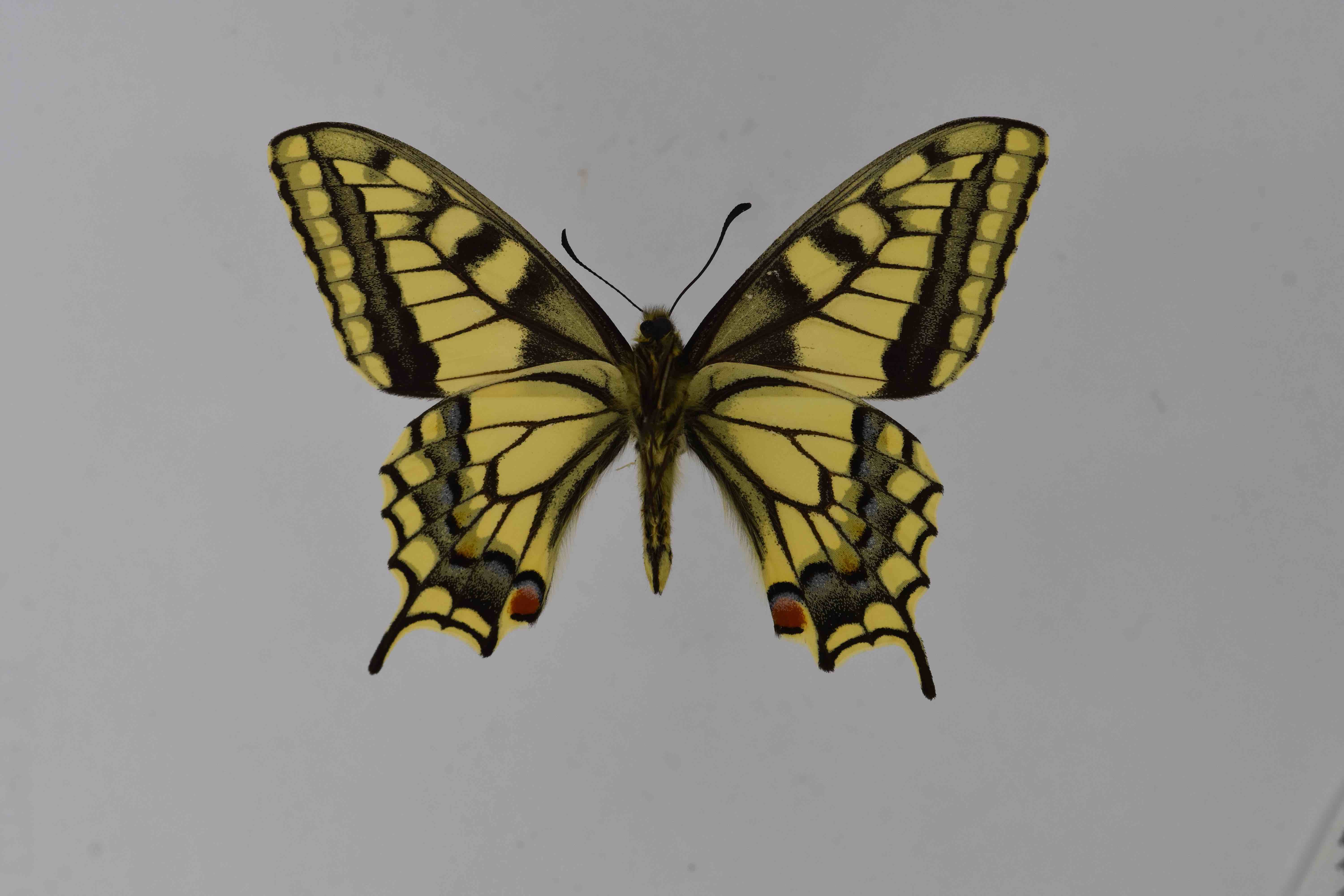

Supplement: S3 Fig — (ZIP) [file pone.0343793.s003.zip › S3/DNAwth016-V copy.jpeg]

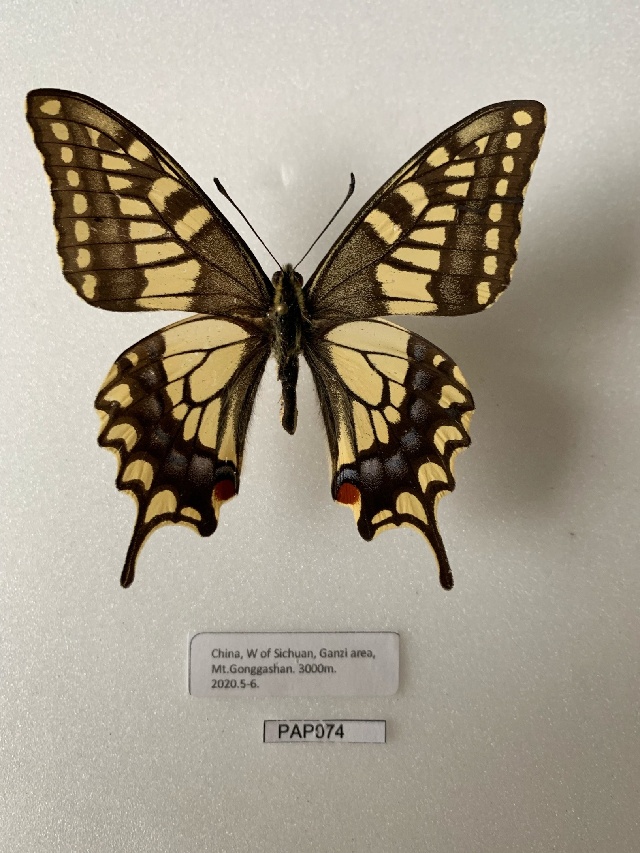

Supplement: S3 Fig — (ZIP) [file pone.0343793.s003.zip › S3/PAP074.jpeg]

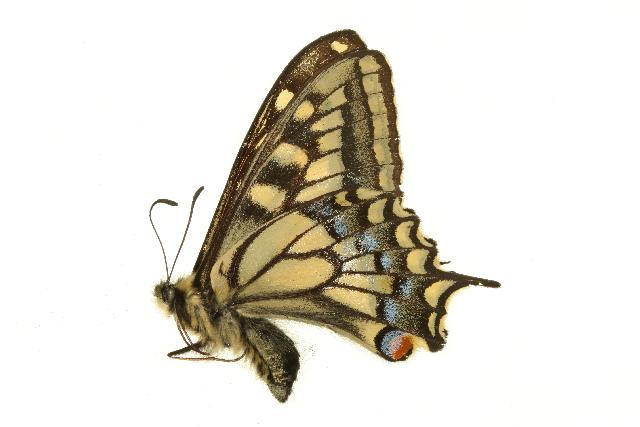

Supplement: S3 Fig — (ZIP) [file pone.0343793.s003.zip › S3/EZ0010CNC .jpeg]

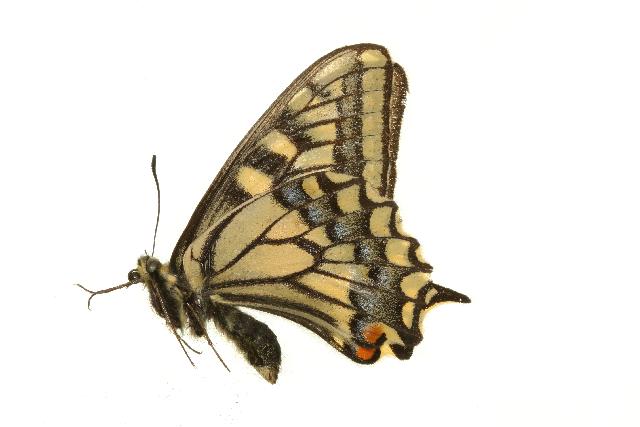

Supplement: S3 Fig — (ZIP) [file pone.0343793.s003.zip › S3/EZ0011CNC .jpeg]

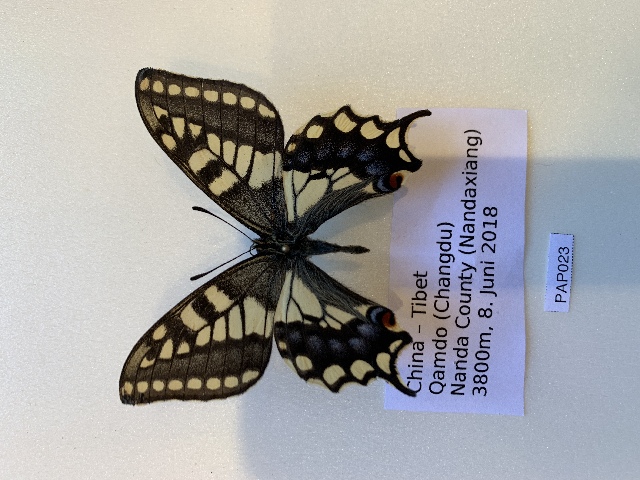

Supplement: S3 Fig — (ZIP) [file pone.0343793.s003.zip › S3/PAP023.jpeg]

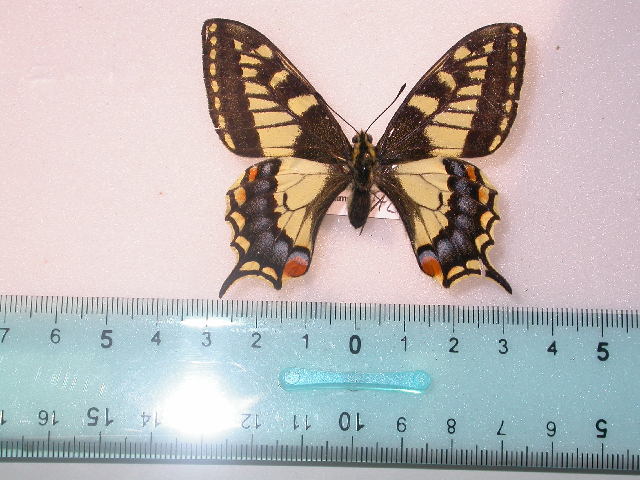

Supplement: S3 Fig — (ZIP) [file pone.0343793.s003.zip › S3/11-H368.jpeg]

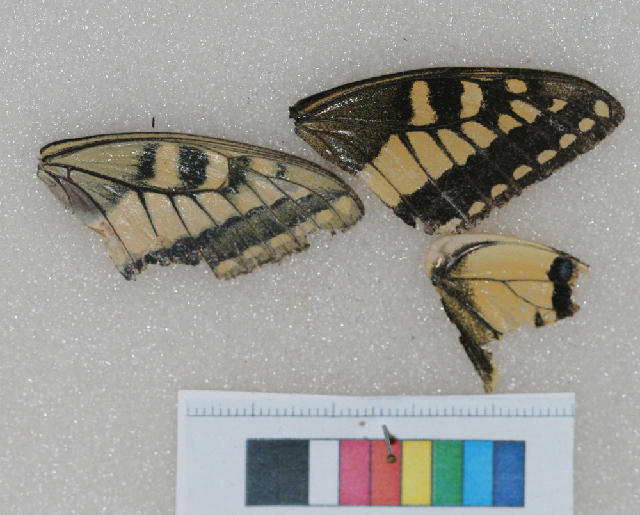

Supplement: S3 Fig — (ZIP) [file pone.0343793.s003.zip › S3/RVcoll.09-X914.jpeg]

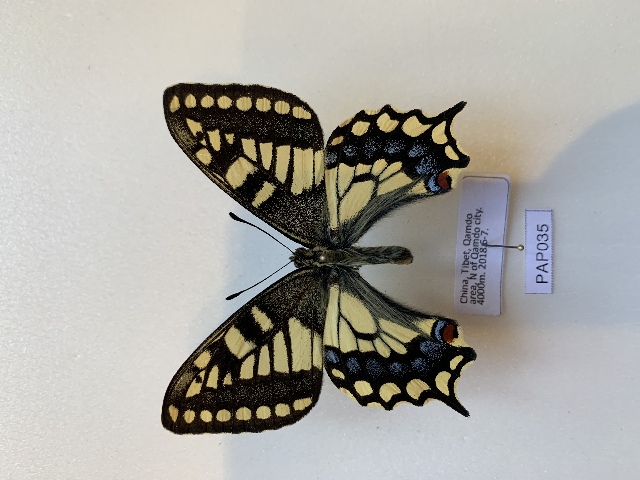

Supplement: S3 Fig — (ZIP) [file pone.0343793.s003.zip › S3/PAP035.jpeg]

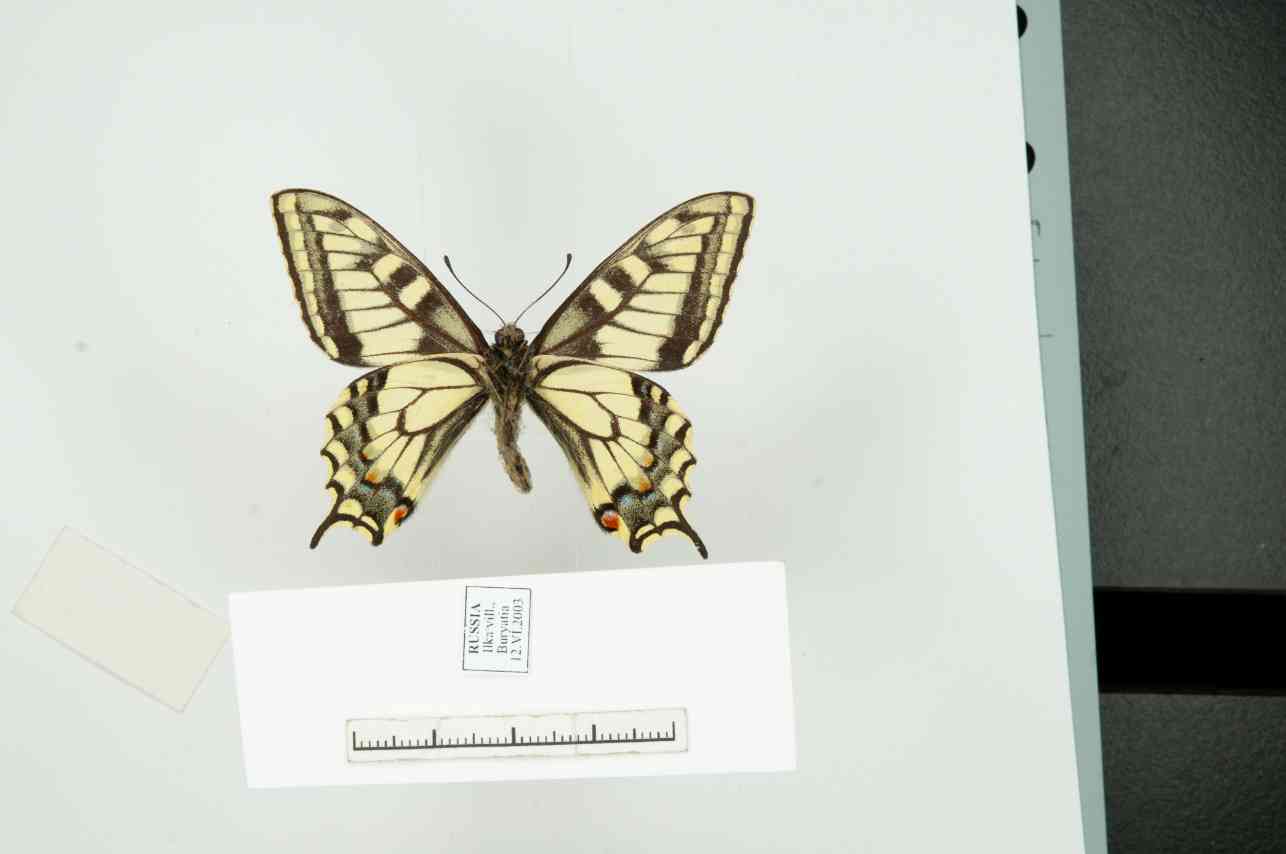

Supplement: S3 Fig — (ZIP) [file pone.0343793.s003.zip › S3/GCB02-V copy.jpg]

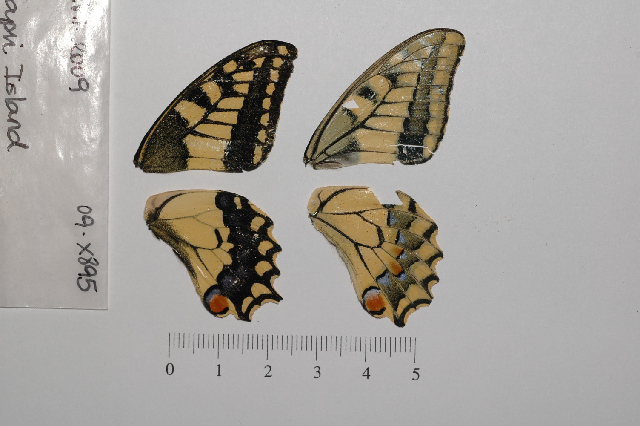

Supplement: S3 Fig — (ZIP) [file pone.0343793.s003.zip › S3/RVcoll.09-X895 .jpg]

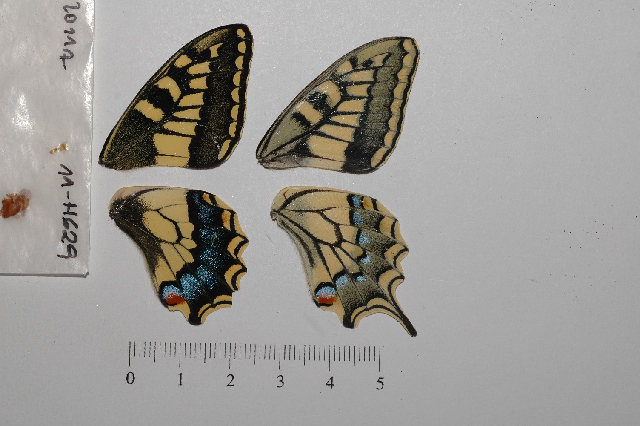

Supplement: S3 Fig — (ZIP) [file pone.0343793.s003.zip › S3/RVcoll.11-H629 .jpg]

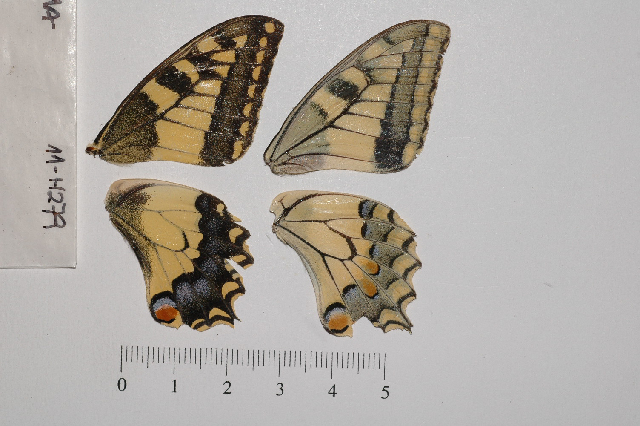

Supplement: S3 Fig — (ZIP) [file pone.0343793.s003.zip › S3/RVcoll.11-H279 .jpg]

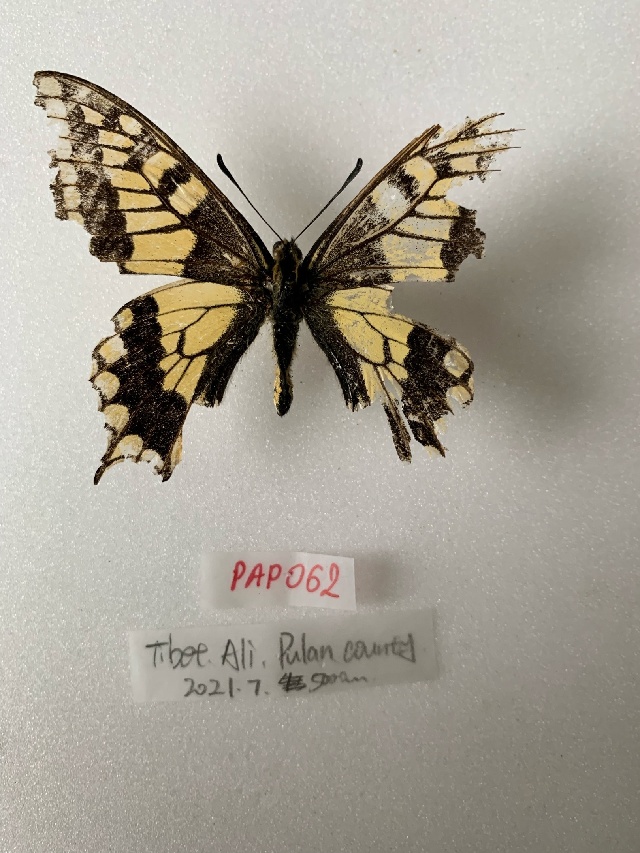

Supplement: S3 Fig — (ZIP) [file pone.0343793.s003.zip › S3/PAP062.jpeg]

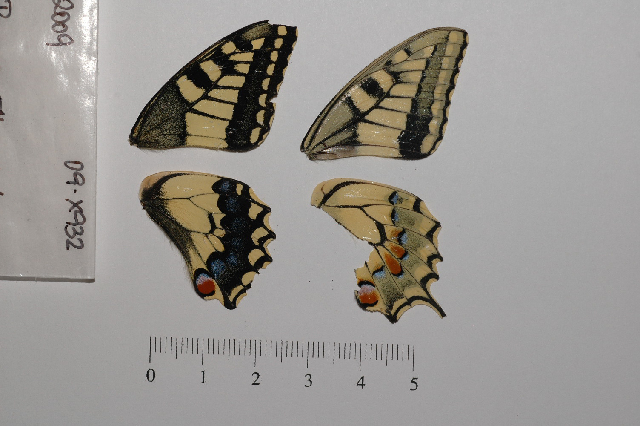

Supplement: S3 Fig — (ZIP) [file pone.0343793.s003.zip › S3/RVcoll.09-X932.jpg]

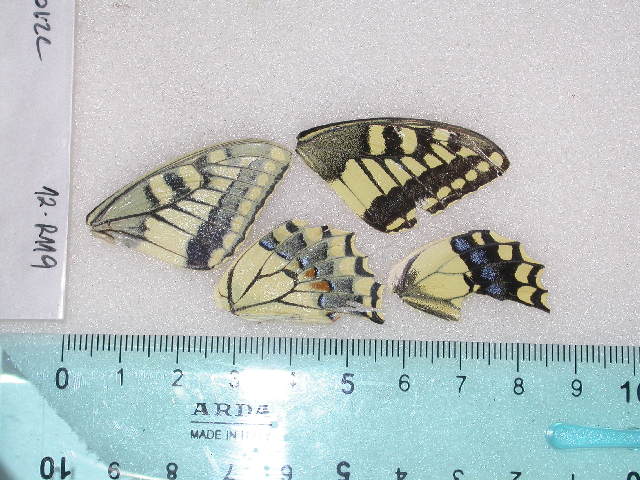

Supplement: S3 Fig — (ZIP) [file pone.0343793.s003.zip › S3/12-R119.jpeg]

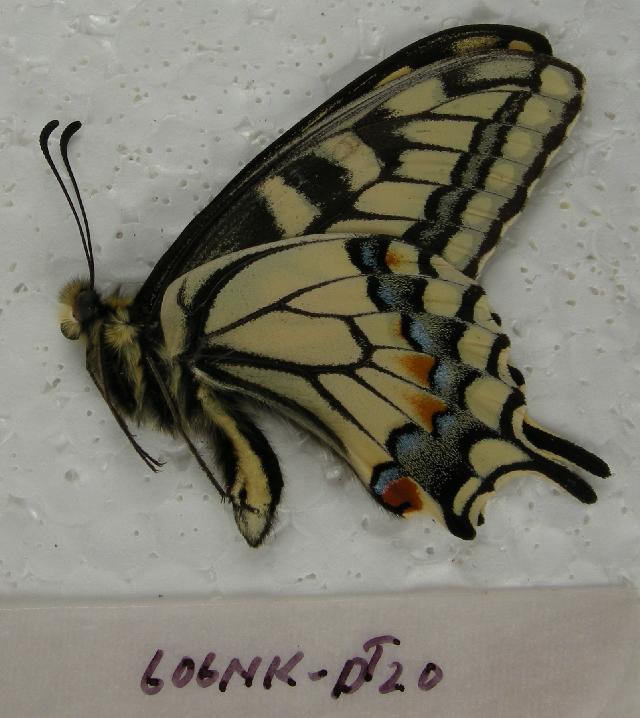

Supplement: S3 Fig — (ZIP) [file pone.0343793.s003.zip › S3/606NK-DT20.jpeg]

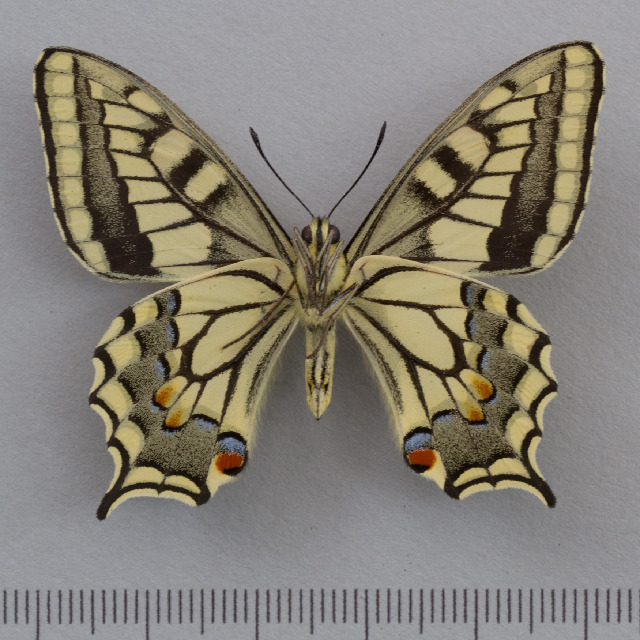

Supplement: S3 Fig — (ZIP) [file pone.0343793.s003.zip › S3/RVcoll.14-O167-V.jpg]

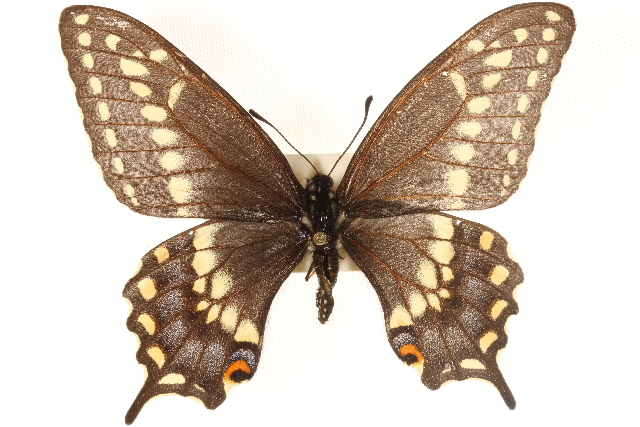

Supplement: S3 Fig — (ZIP) [file pone.0343793.s003.zip › S3/CCDB-24271-E02.jpeg]

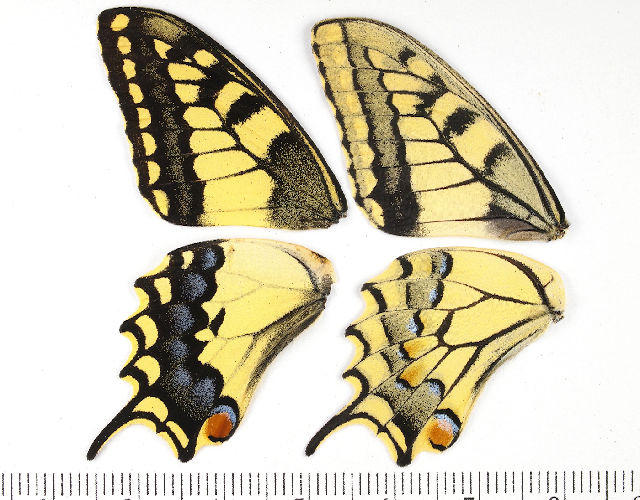

Supplement: S3 Fig — (ZIP) [file pone.0343793.s003.zip › S3/RVcoll15I902 .jpeg]

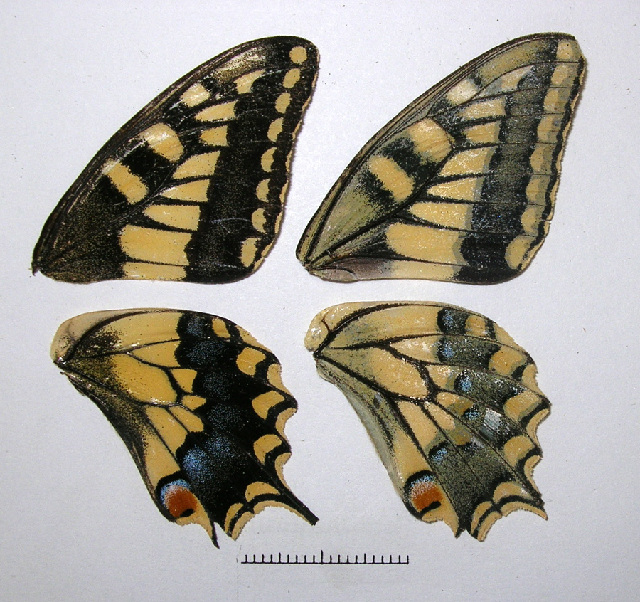

Supplement: S3 Fig — (ZIP) [file pone.0343793.s003.zip › S3/RVcoll.11-H630 .jpeg]

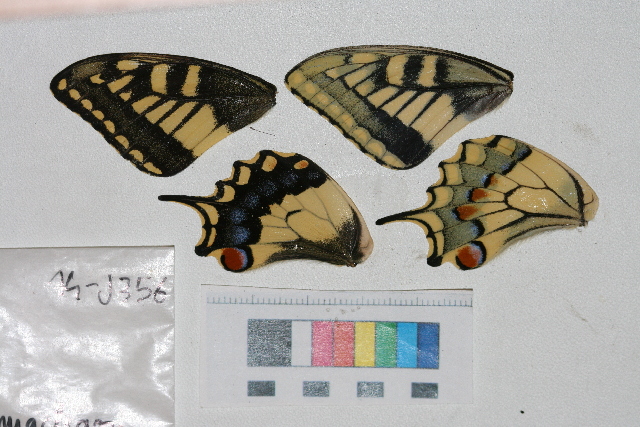

Supplement: S3 Fig — (ZIP) [file pone.0343793.s003.zip › S3/RVcoll.14-J356 .jpeg]

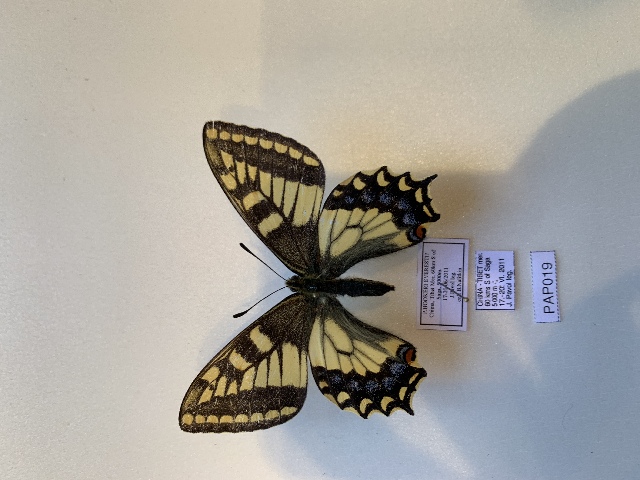

Supplement: S3 Fig — (ZIP) [file pone.0343793.s003.zip › S3/PAP019.jpeg]

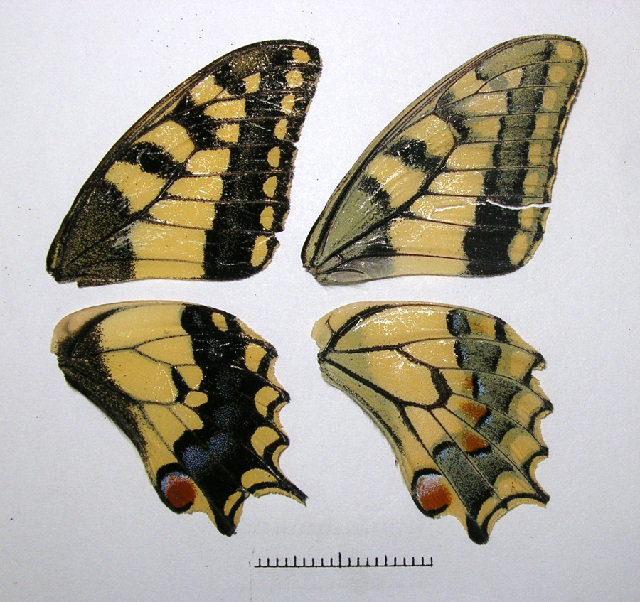

Supplement: S3 Fig — (ZIP) [file pone.0343793.s003.zip › S3/RVcoll.10-B602 .jpeg]

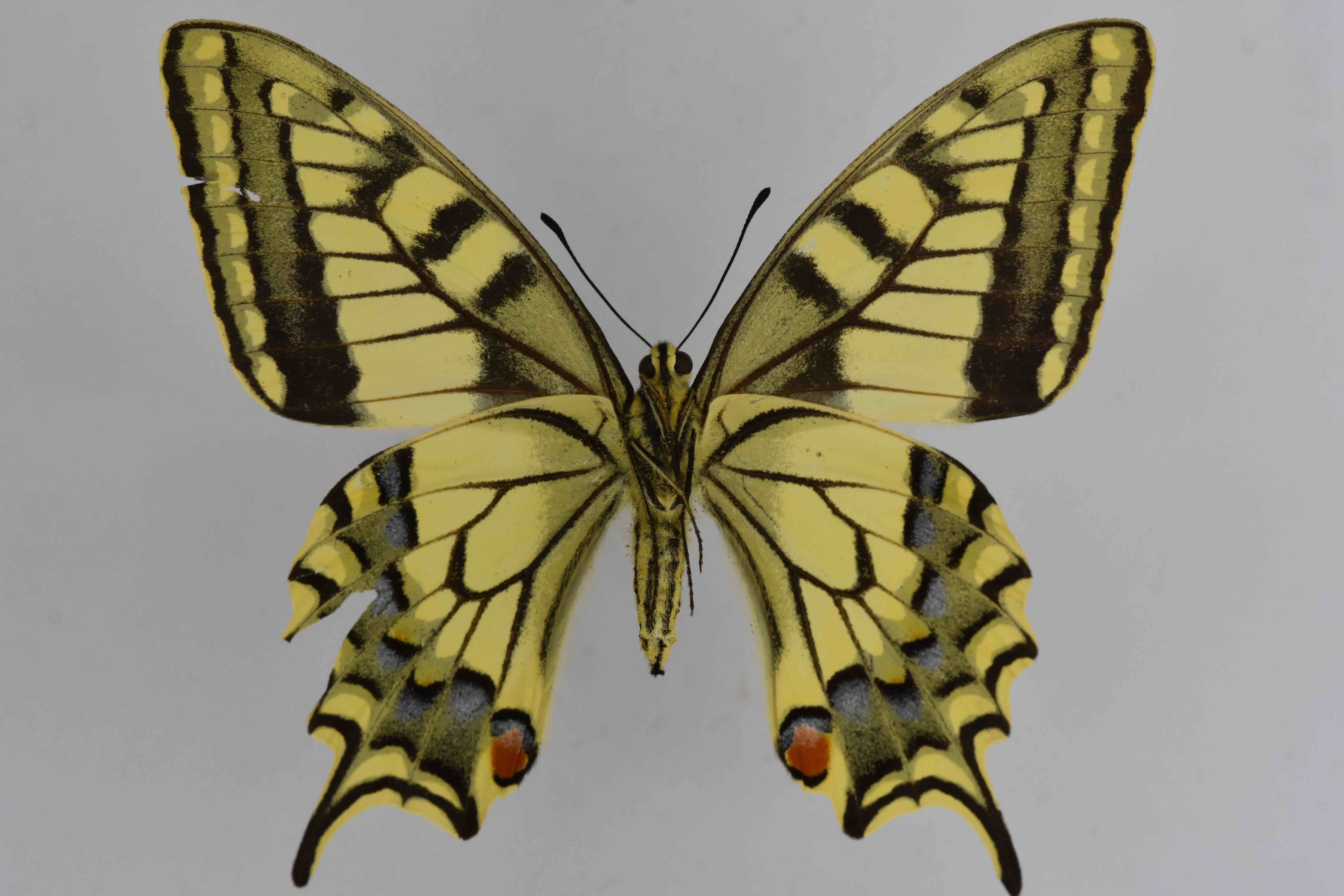

Supplement: S3 Fig — (ZIP) [file pone.0343793.s003.zip › S3/DNAwth024-V copy.jpeg]

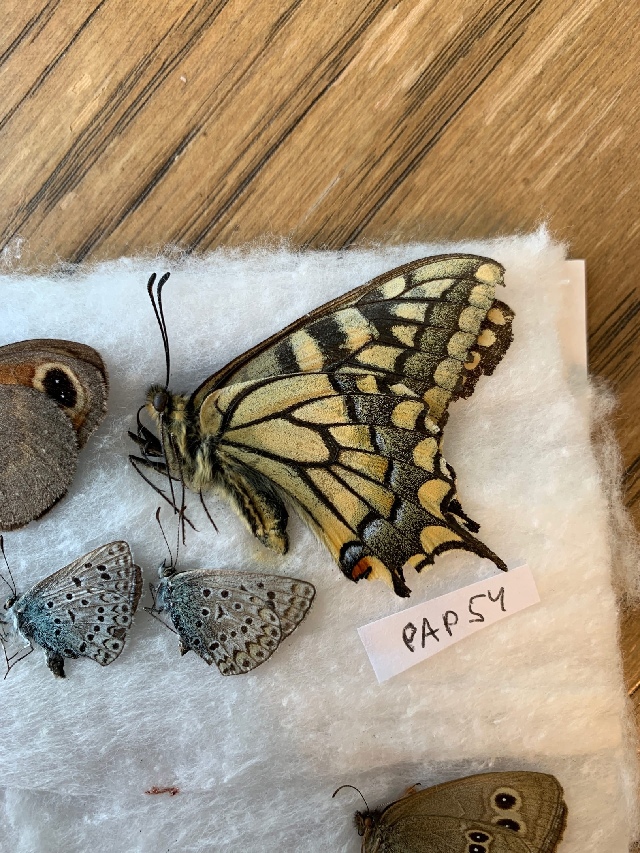

Supplement: S3 Fig — (ZIP) [file pone.0343793.s003.zip › S3/PAP054b.jpeg]

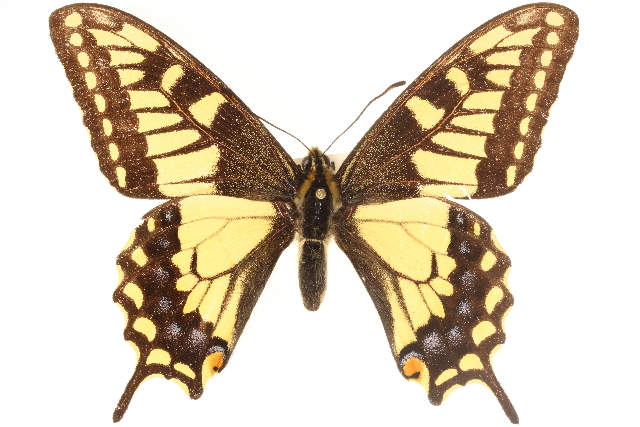

Supplement: S3 Fig — (ZIP) [file pone.0343793.s003.zip › S3/CCDB-24271-A05.jpeg]

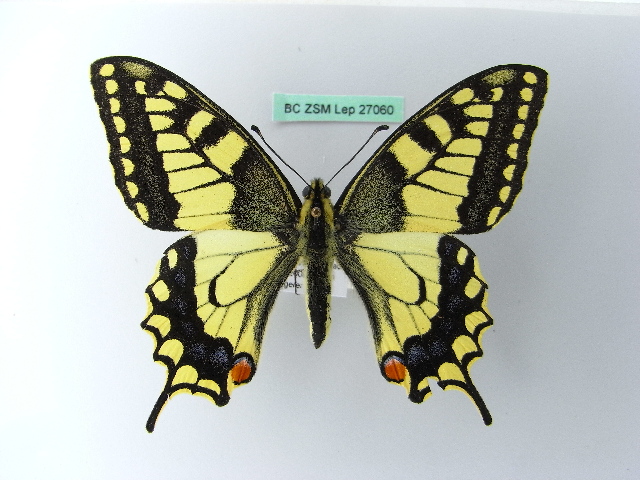

Supplement: S3 Fig — (ZIP) [file pone.0343793.s003.zip › S3/BC ZSM Lep 27060.jpg]

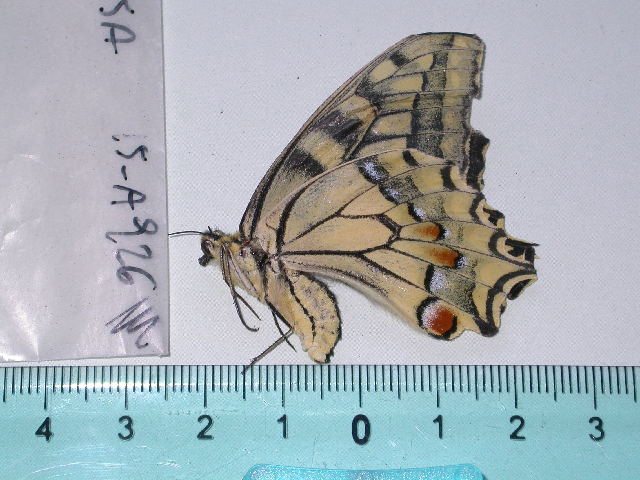

Supplement: S3 Fig — (ZIP) [file pone.0343793.s003.zip › S3/15-A926.jpeg]

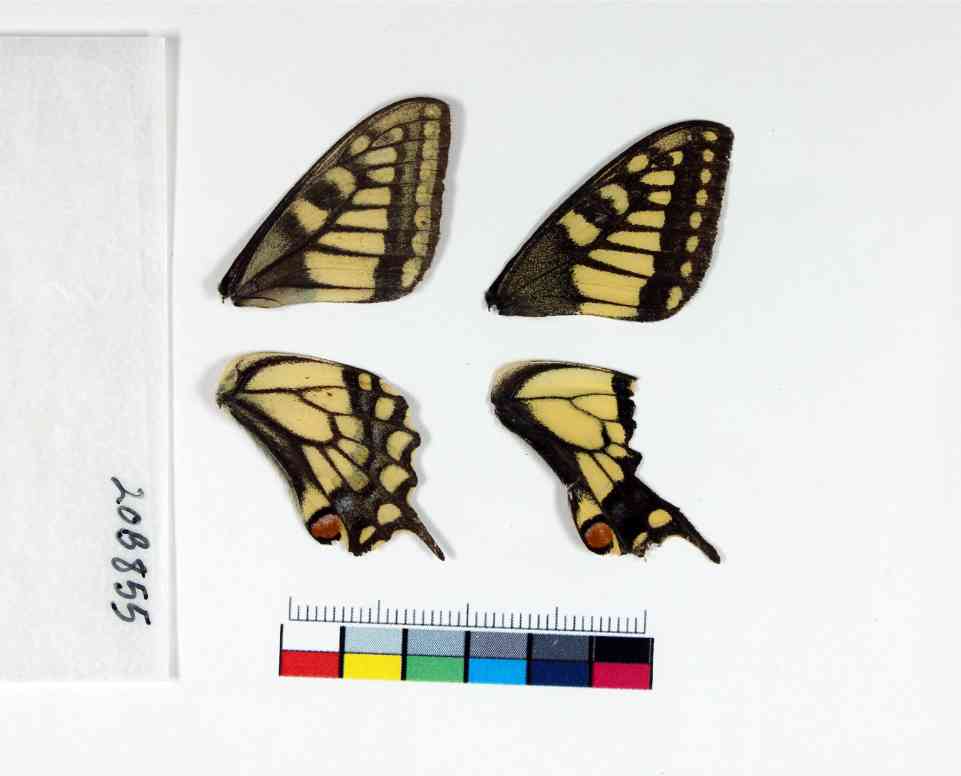

Supplement: S3 Fig — (ZIP) [file pone.0343793.s003.zip › S3/AC-PQ002 copy.jpg]

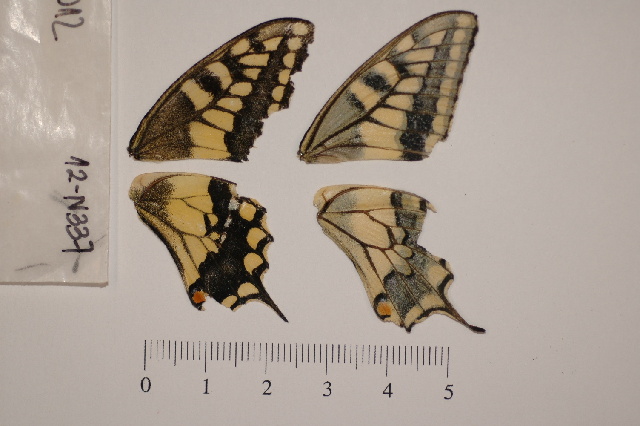

Supplement: S3 Fig — (ZIP) [file pone.0343793.s003.zip › S3/RVcoll.12-N337 .jpeg]

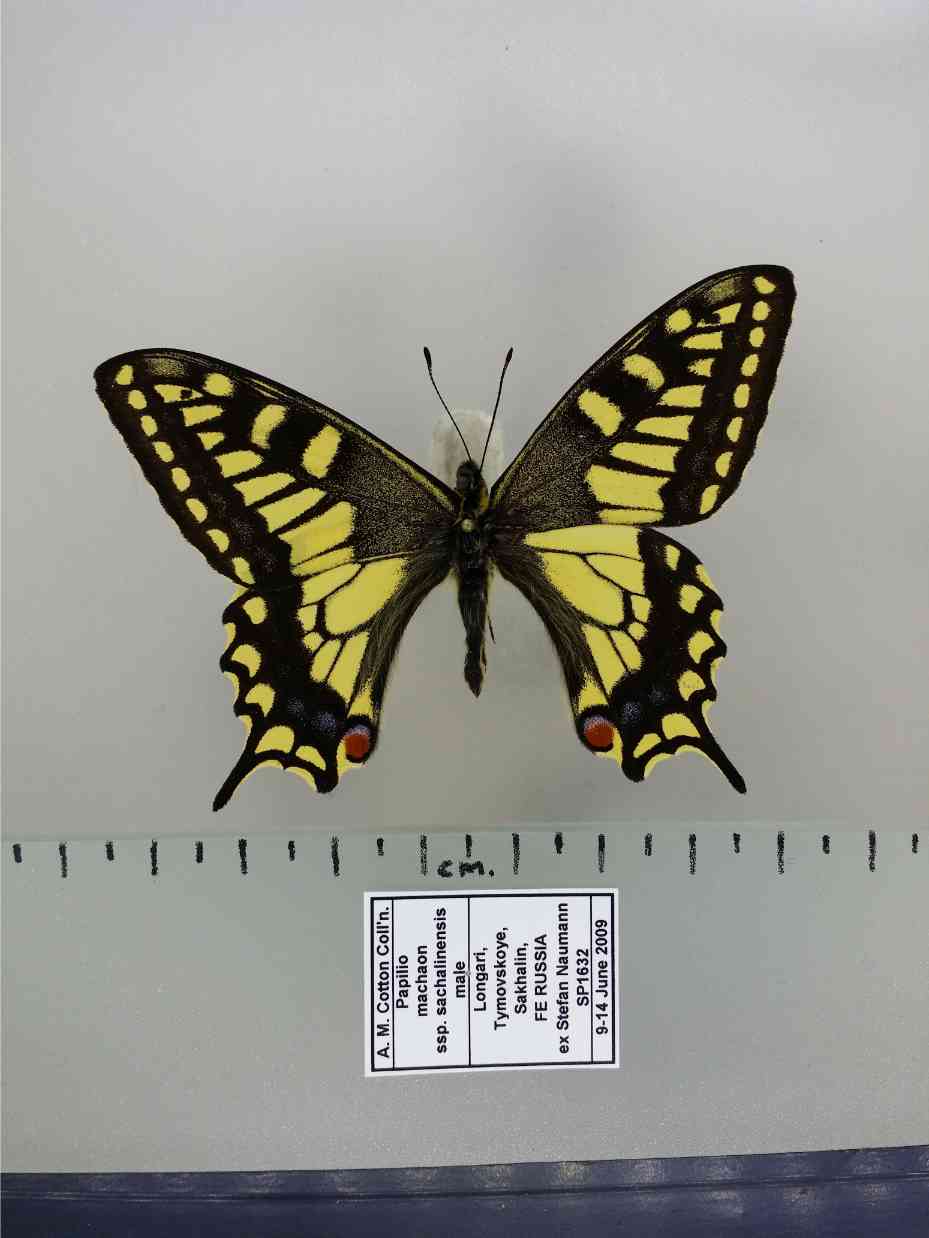

Supplement: S3 Fig — (ZIP) [file pone.0343793.s003.zip › S3/AC-SP1632D copy.jpg]

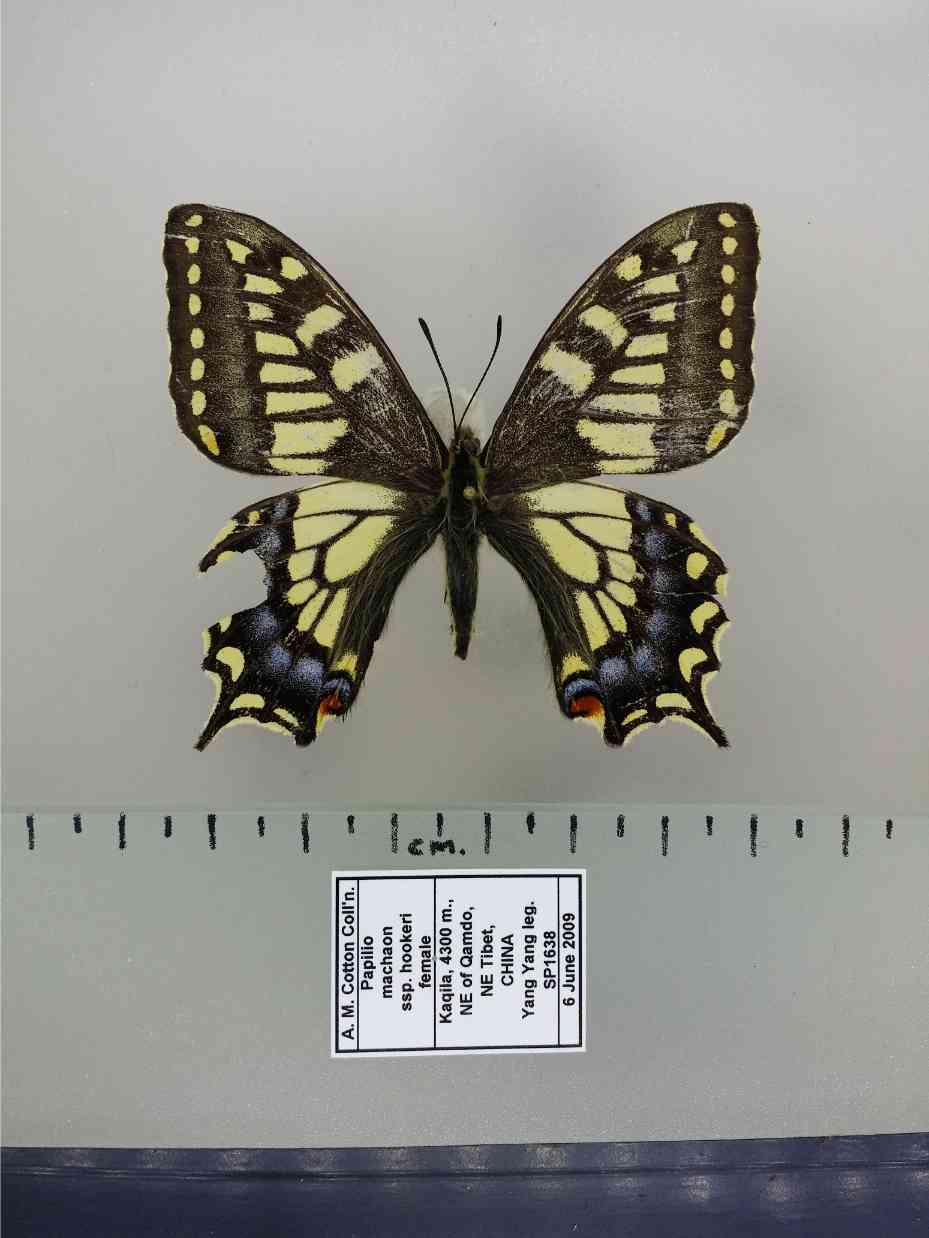

Supplement: S3 Fig — (ZIP) [file pone.0343793.s003.zip › S3/AC-SP1638D copy.jpg]

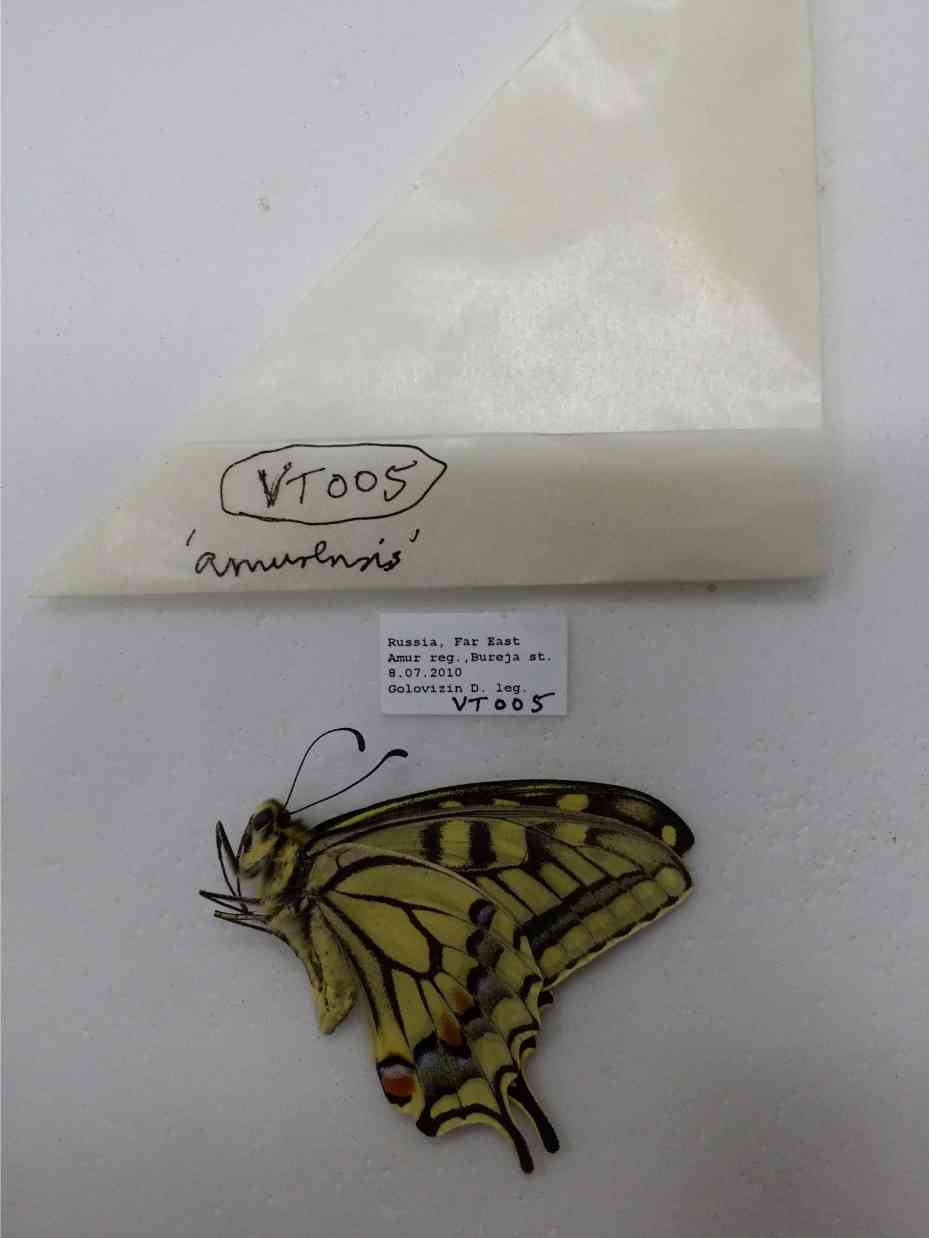

Supplement: S3 Fig — (ZIP) [file pone.0343793.s003.zip › S3/AC-VT005 copy.jpg]

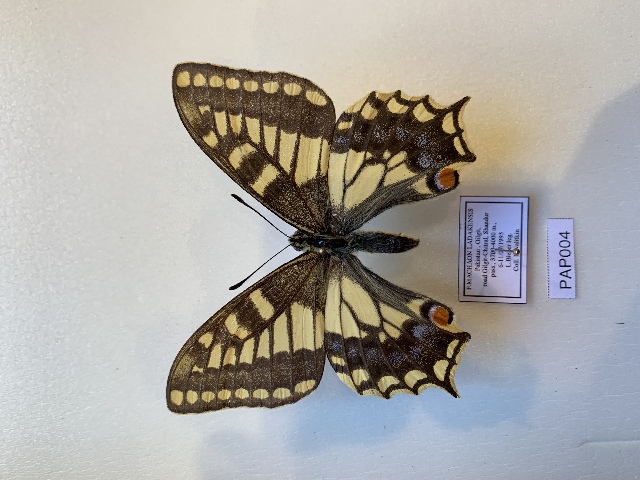

Supplement: S3 Fig — (ZIP) [file pone.0343793.s003.zip › S3/PAP004.jpeg]

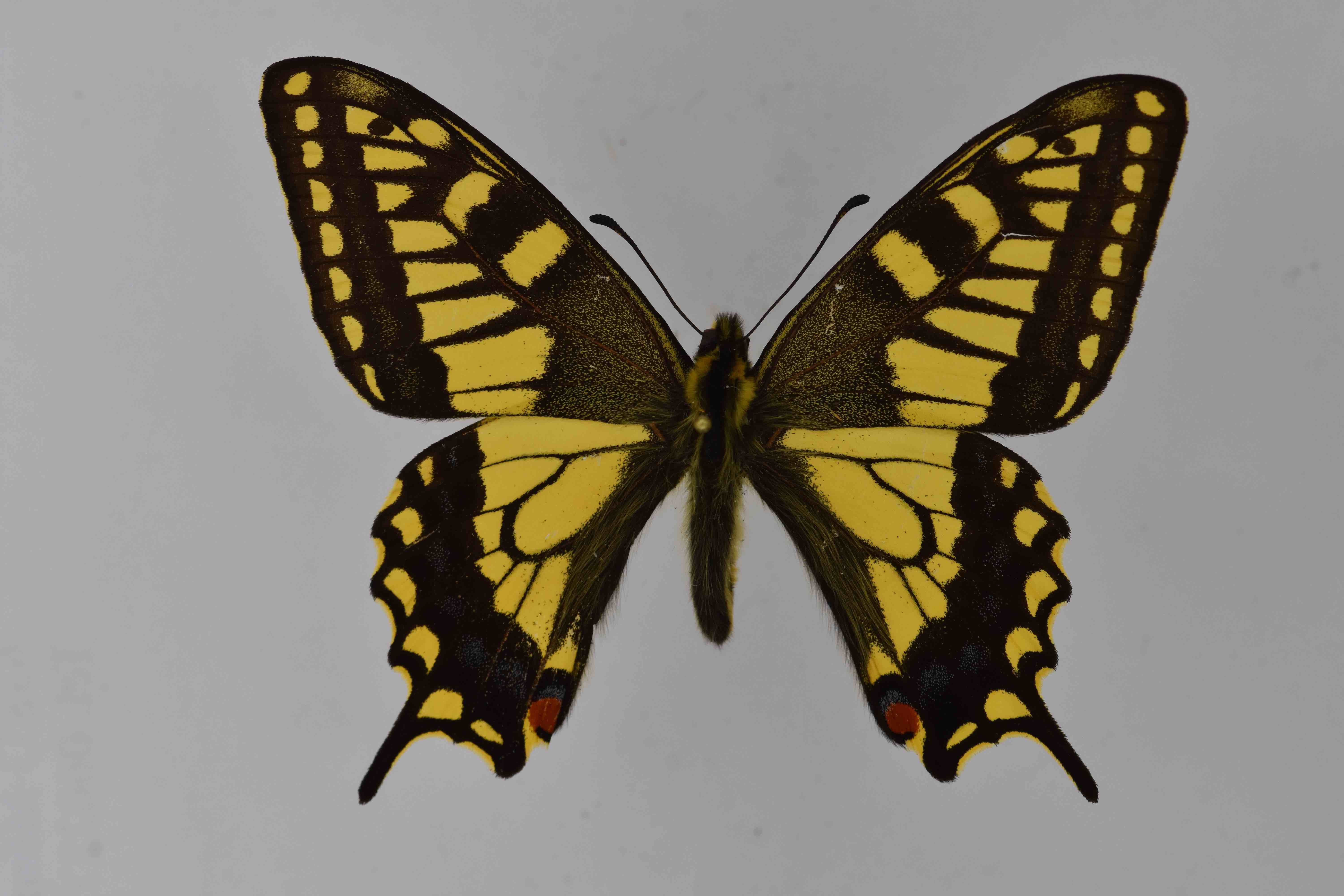

Supplement: S3 Fig — (ZIP) [file pone.0343793.s003.zip › S3/DNAwth031-D copy.jpeg]

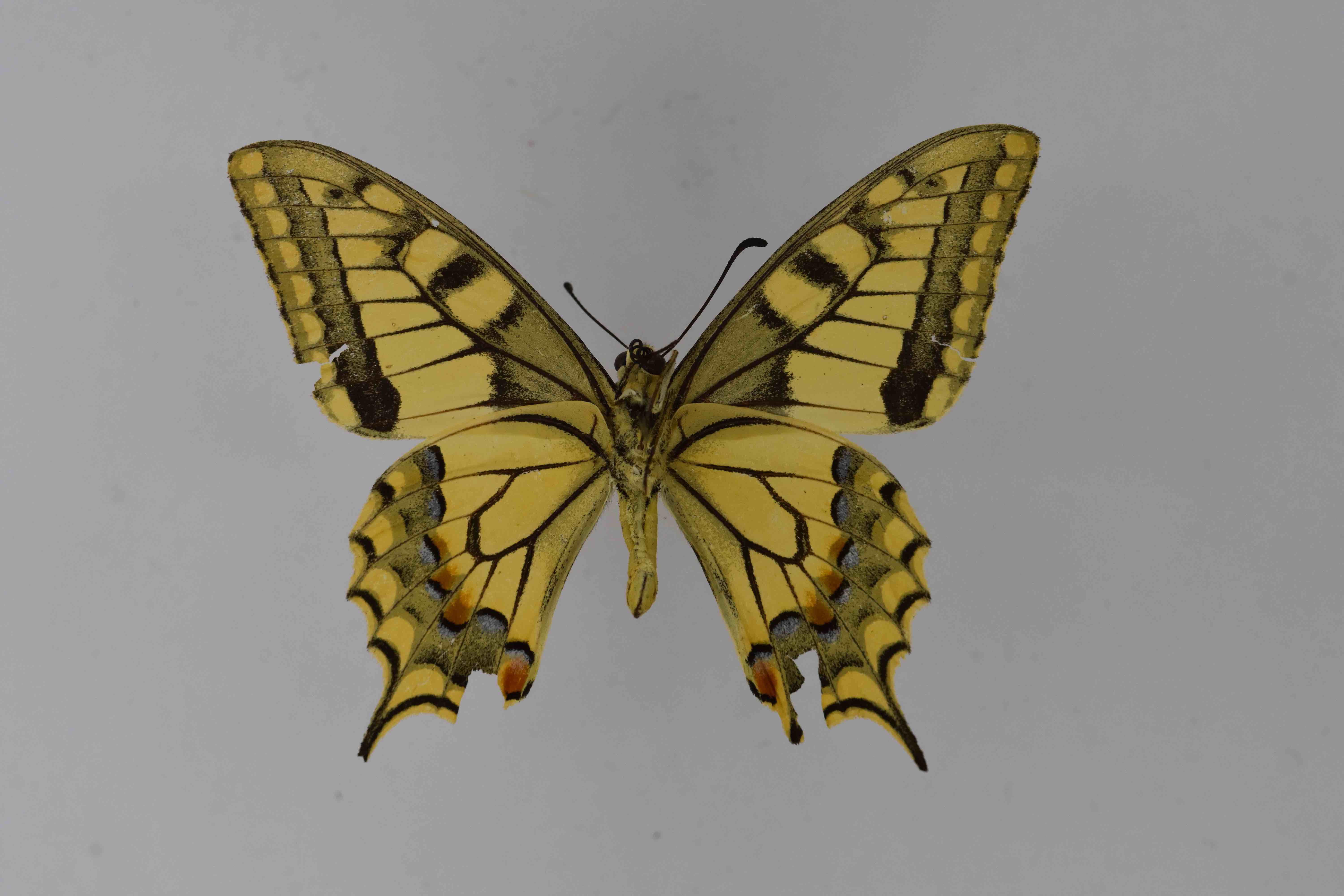

Supplement: S3 Fig — (ZIP) [file pone.0343793.s003.zip › S3/DNAwth009-V copy.jpeg]

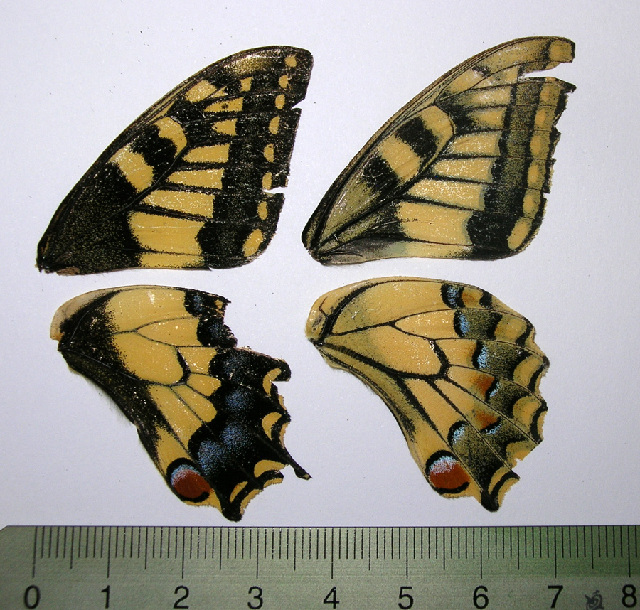

Supplement: S3 Fig — (ZIP) [file pone.0343793.s003.zip › S3/Rvcoll.12-L899 .jpeg]

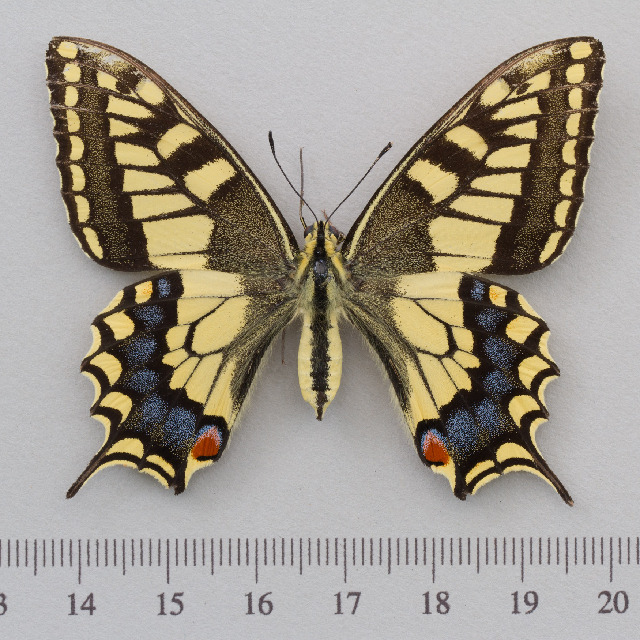

Supplement: S3 Fig — (ZIP) [file pone.0343793.s003.zip › S3/RVcoll.14-O164-D.jpg]

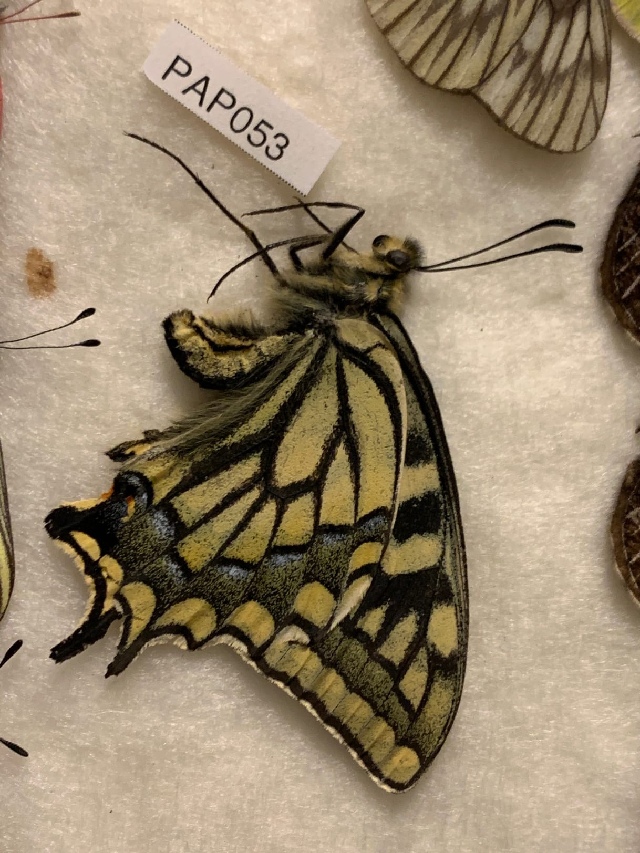

Supplement: S3 Fig — (ZIP) [file pone.0343793.s003.zip › S3/PAP053.jpeg]

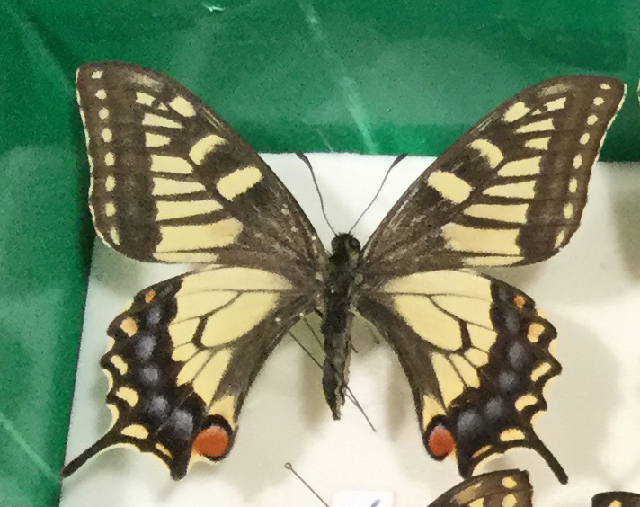

Supplement: S3 Fig — (ZIP) [file pone.0343793.s003.zip › S3/NS_101.jpeg]

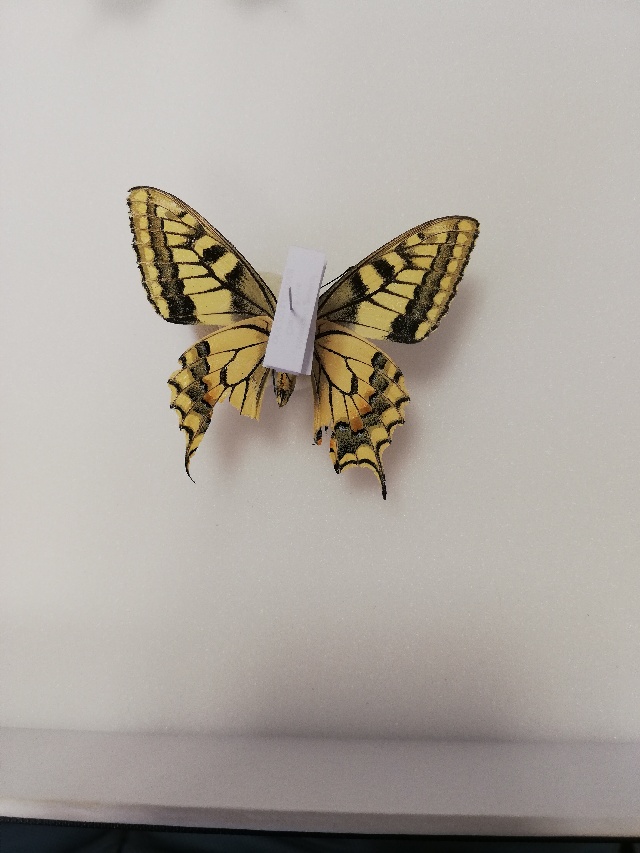

Supplement: S3 Fig — (ZIP) [file pone.0343793.s003.zip › S3/OCIC-PM8-V.jpeg]

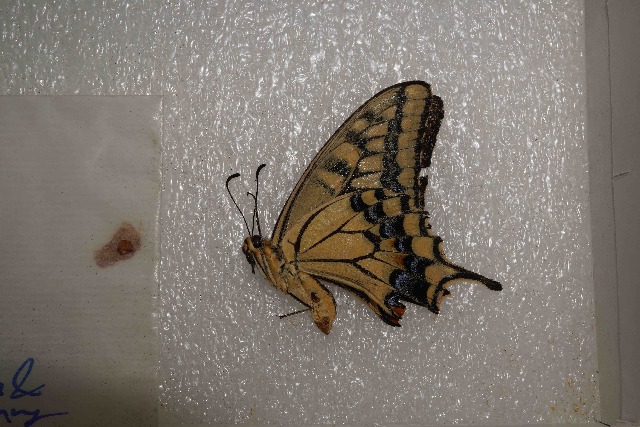

Supplement: S3 Fig — (ZIP) [file pone.0343793.s003.zip › S3/PAP100.jpeg]

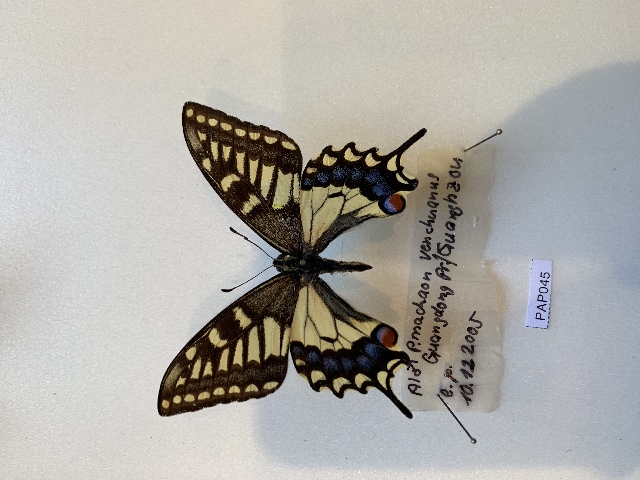

Supplement: S3 Fig — (ZIP) [file pone.0343793.s003.zip › S3/PAP045.jpeg]

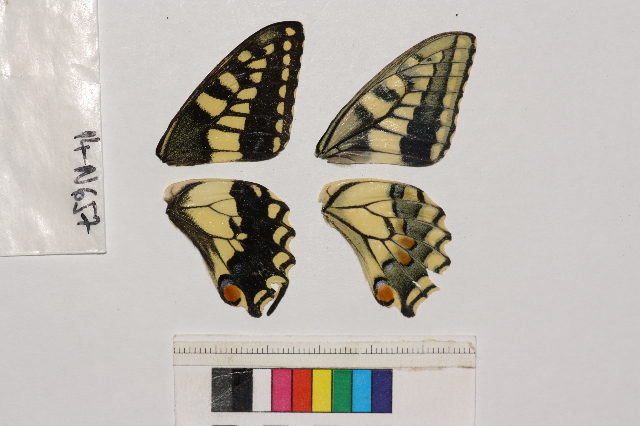

Supplement: S3 Fig — (ZIP) [file pone.0343793.s003.zip › S3/RVcoll.14-N657 .jpeg]

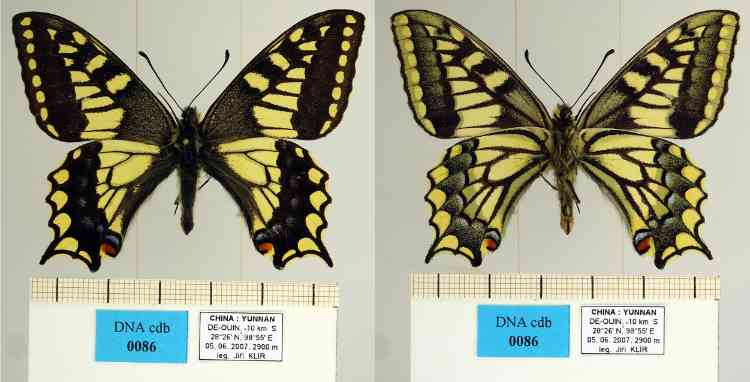

Supplement: S3 Fig — (ZIP) [file pone.0343793.s003.zip › S3/DNAcdb0086 copy.jpg]

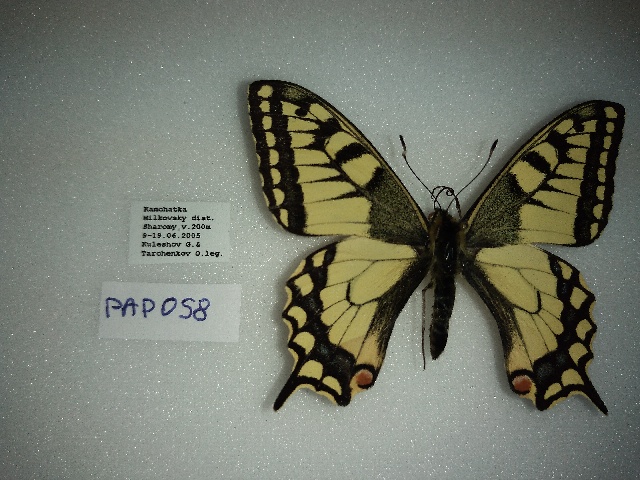

Supplement: S3 Fig — (ZIP) [file pone.0343793.s003.zip › S3/PAP058XR.jpeg]

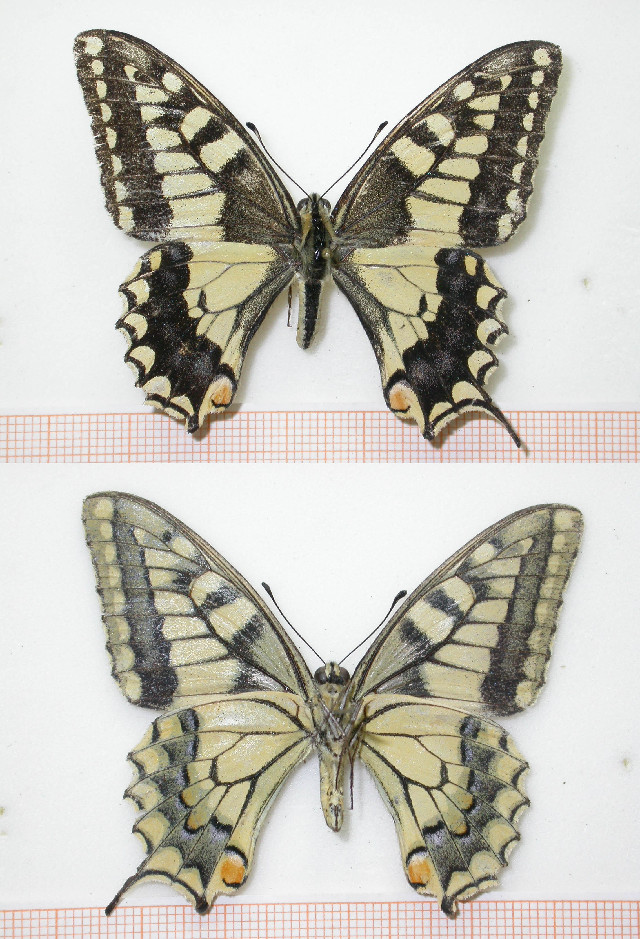

Supplement: S3 Fig — (ZIP) [file pone.0343793.s003.zip › S3/LEP-SS-00141.jpg]

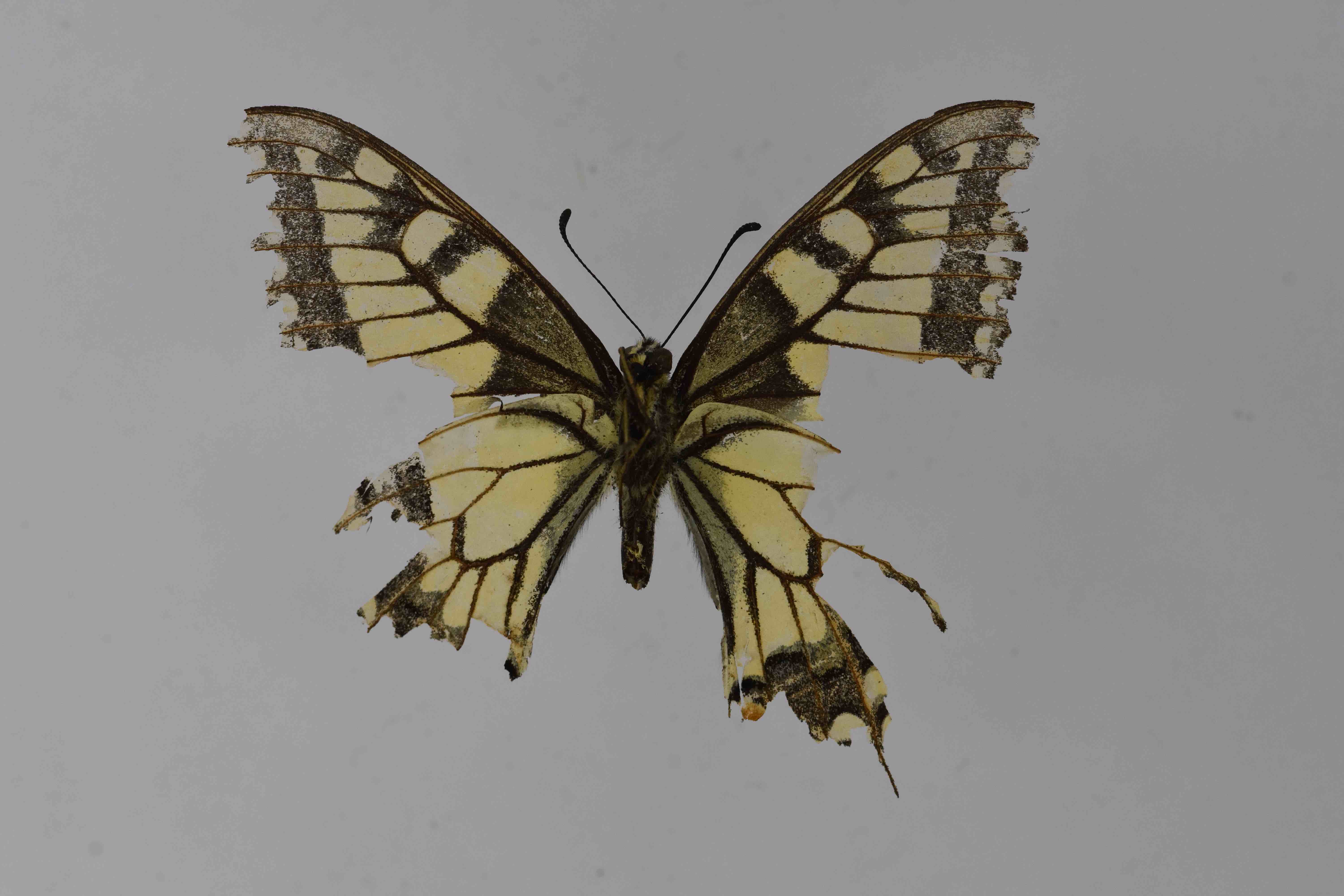

Supplement: S3 Fig — (ZIP) [file pone.0343793.s003.zip › S3/DNAwth027-V copy.jpeg]

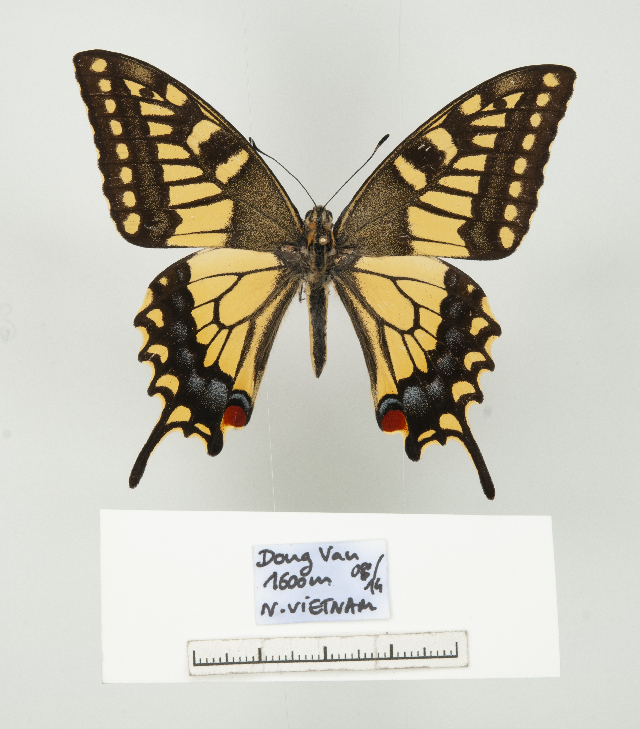

Supplement: S3 Fig — (ZIP) [file pone.0343793.s003.zip › S3/GCB01-D copy.jpeg]

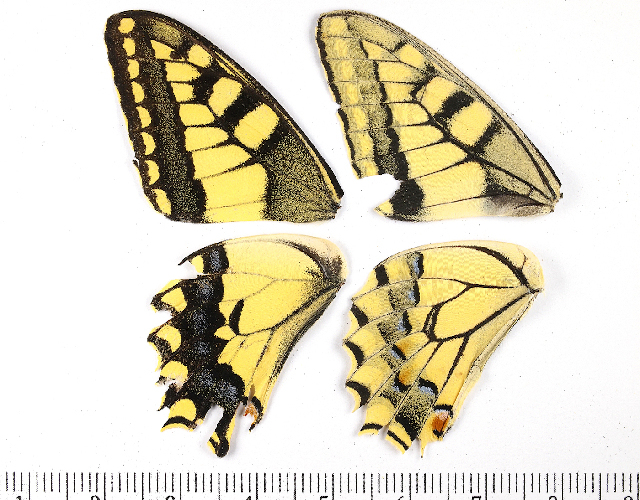

Supplement: S3 Fig — (ZIP) [file pone.0343793.s003.zip › S3/RVcoll15J461 .jpeg]

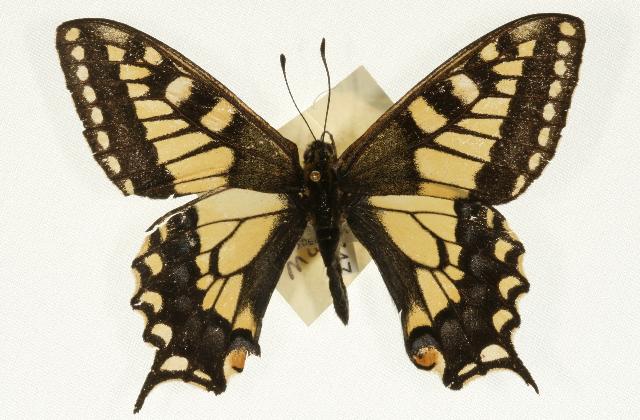

Supplement: S3 Fig — (ZIP) [file pone.0343793.s003.zip › S3/EZ1393CNC.jpeg]

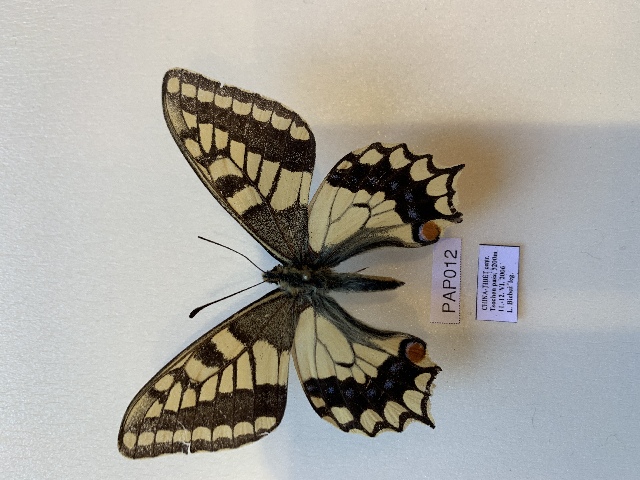

Supplement: S3 Fig — (ZIP) [file pone.0343793.s003.zip › S3/PAP012.jpeg]

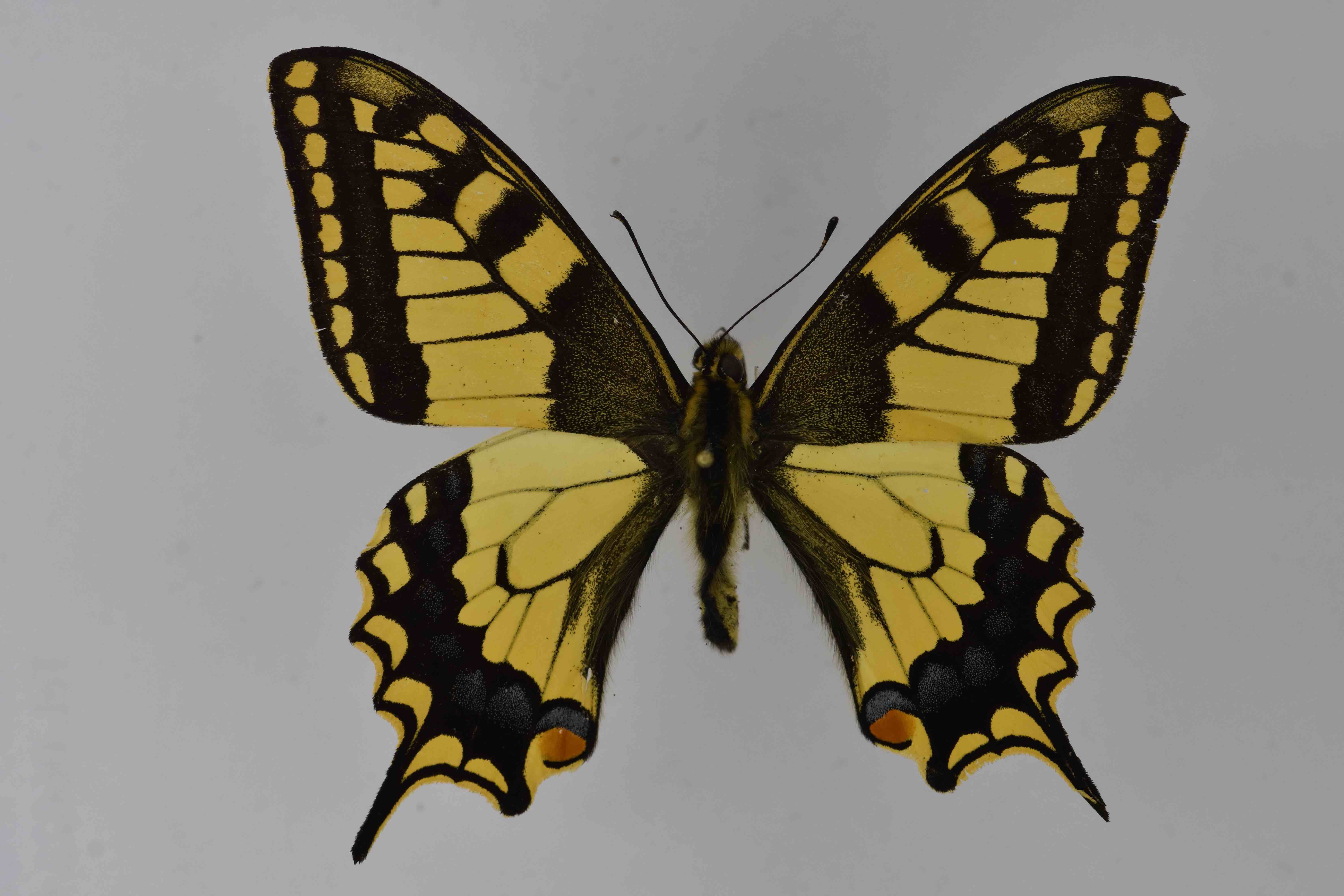

Supplement: S3 Fig — (ZIP) [file pone.0343793.s003.zip › S3/DNAwth003-D copy.jpeg]

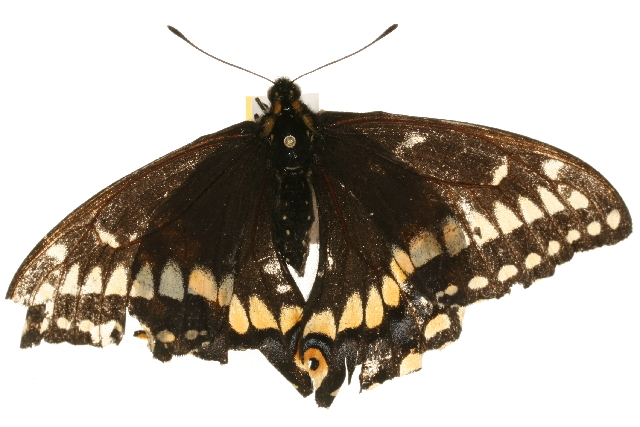

Supplement: S3 Fig — (ZIP) [file pone.0343793.s003.zip › S3/09BBELE-2648.jpeg]

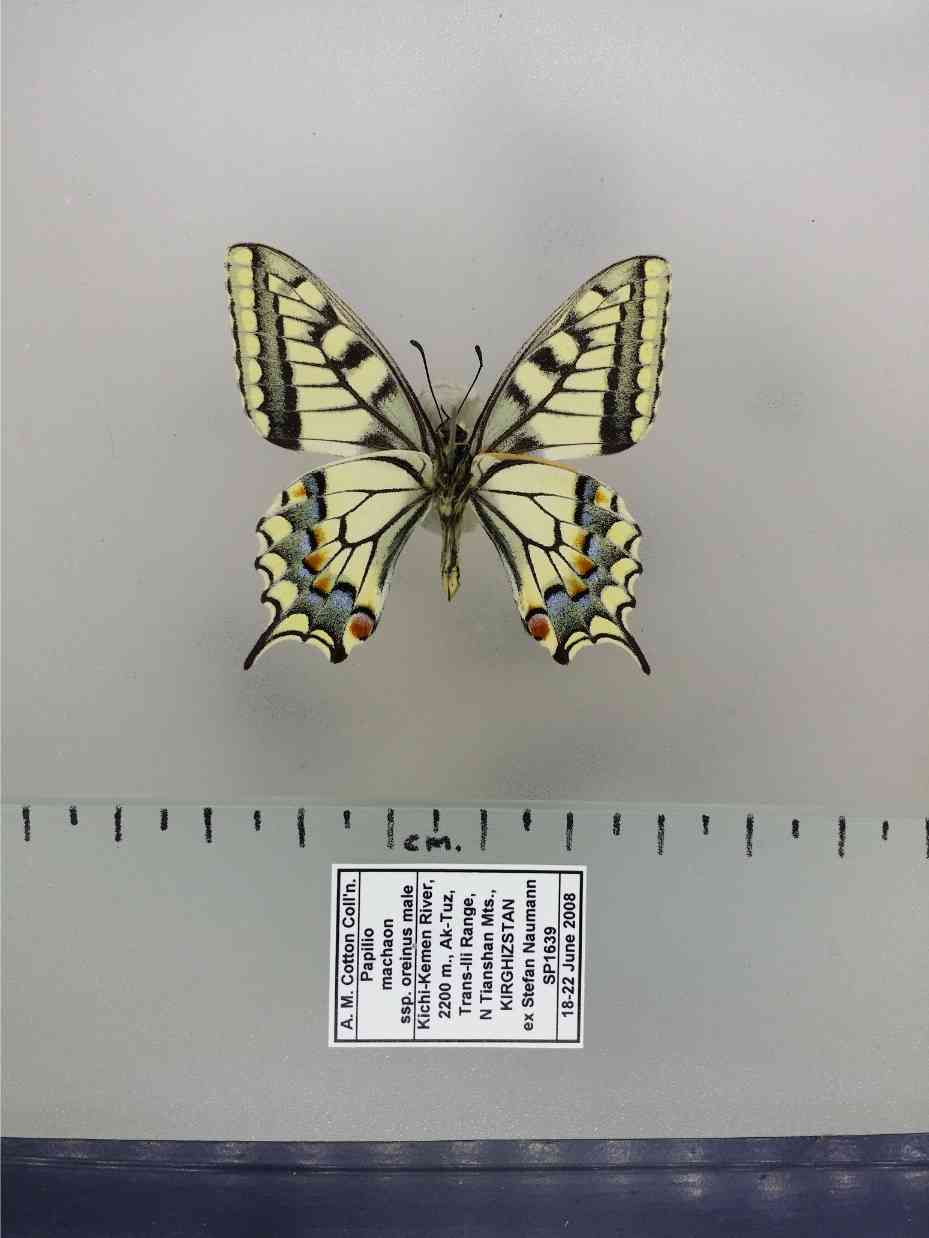

Supplement: S3 Fig — (ZIP) [file pone.0343793.s003.zip › S3/AC-SP1639V copy.jpg]

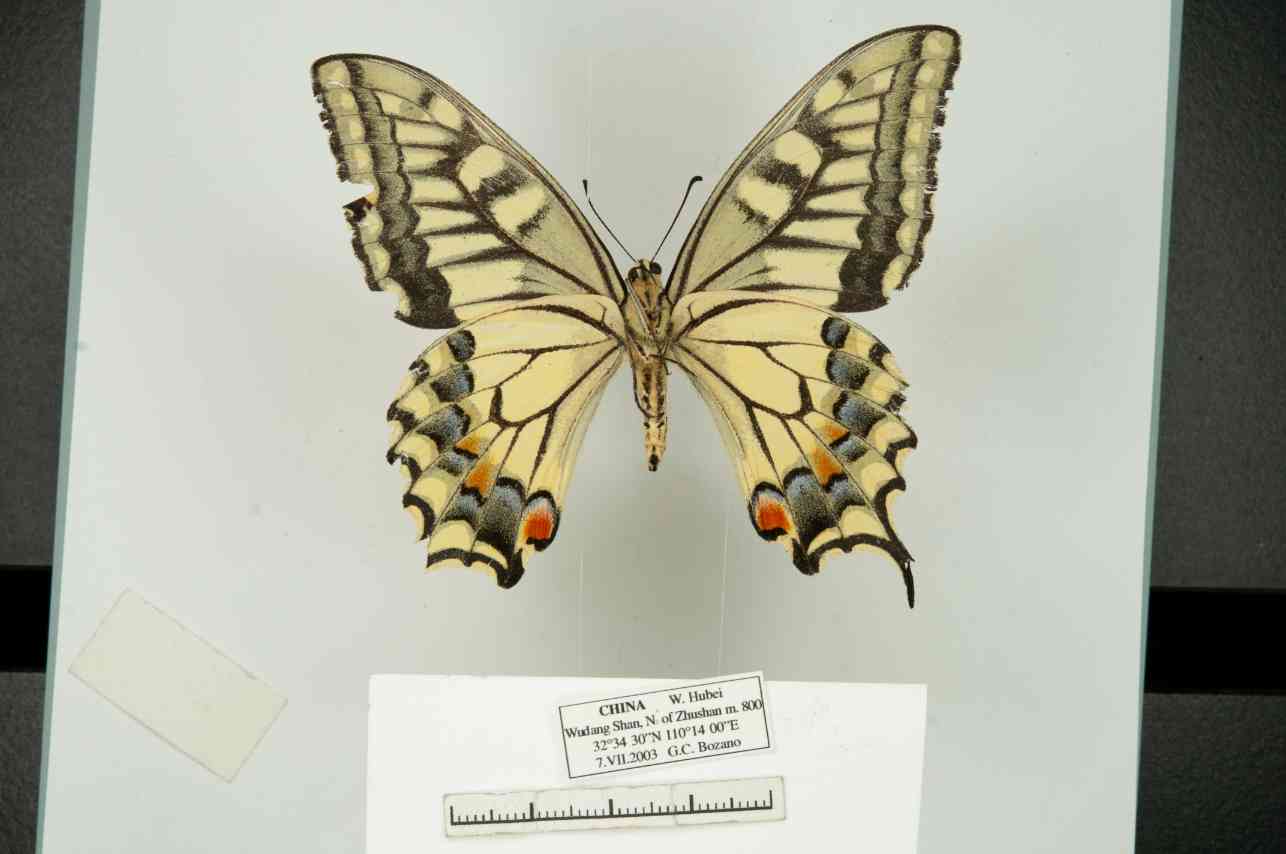

Supplement: S3 Fig — (ZIP) [file pone.0343793.s003.zip › S3/GCB05-V copy.jpg]

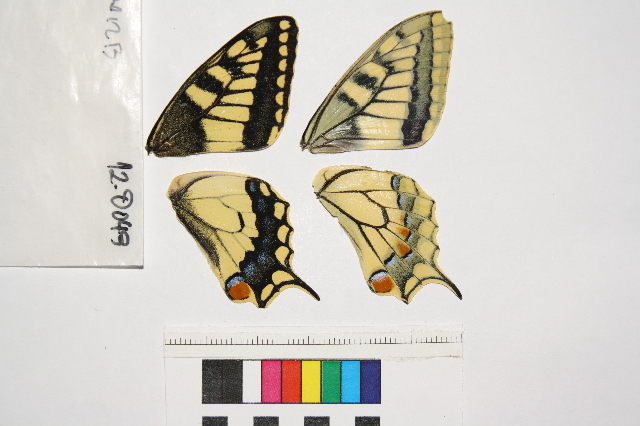

Supplement: S3 Fig — (ZIP) [file pone.0343793.s003.zip › S3/RVcoll.12-O049 .jpeg]

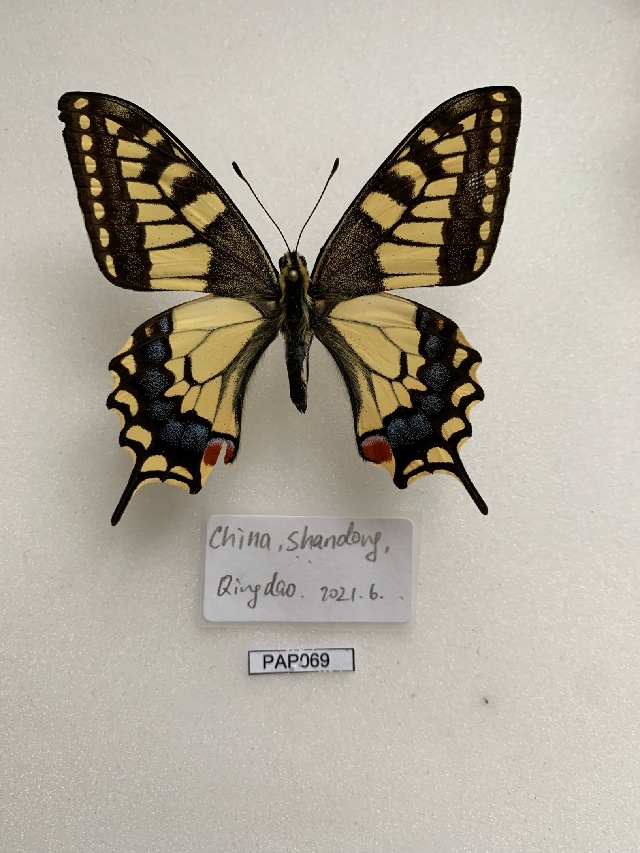

Supplement: S3 Fig — (ZIP) [file pone.0343793.s003.zip › S3/PAP069.jpeg]

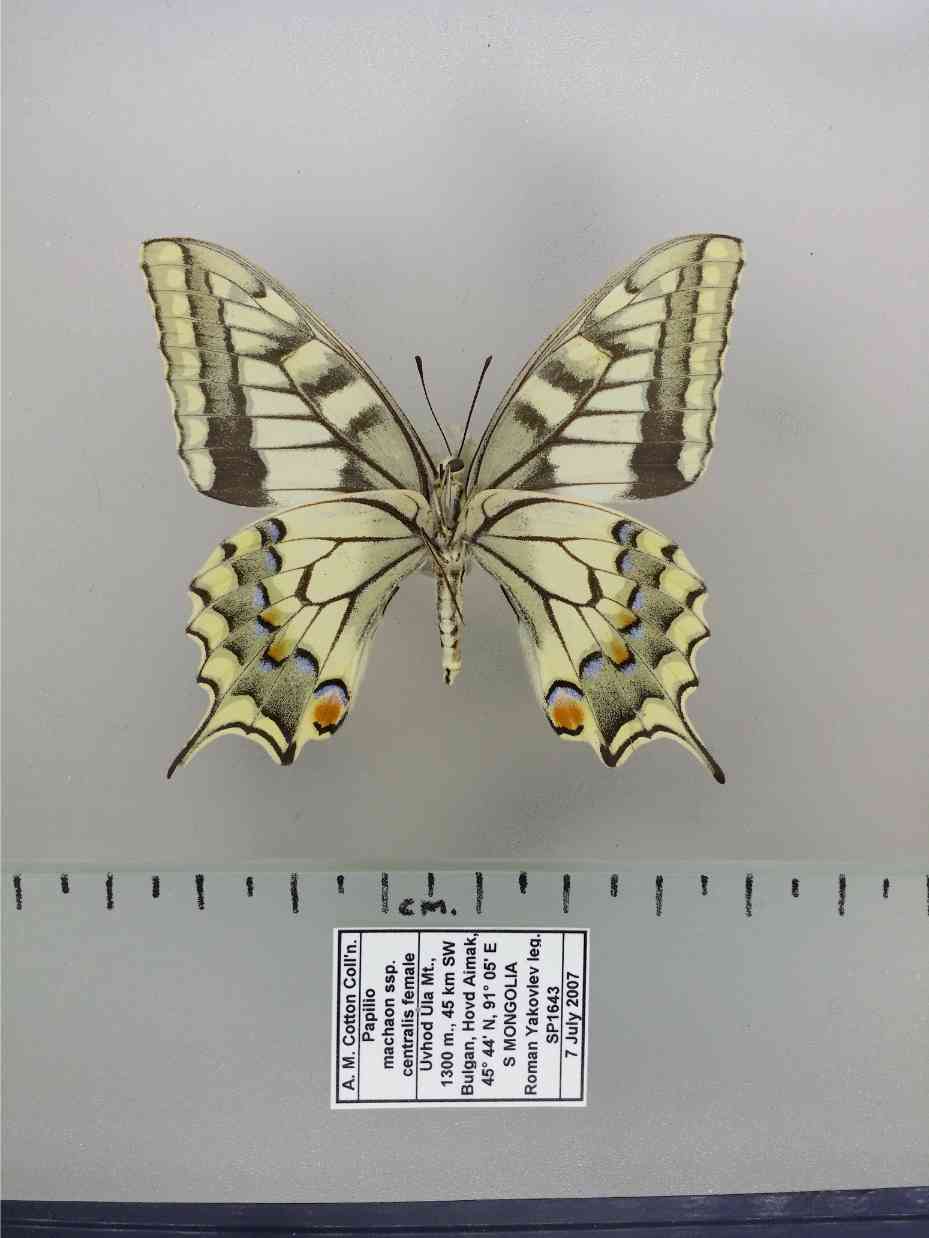

Supplement: S3 Fig — (ZIP) [file pone.0343793.s003.zip › S3/AC-SP1643V copy.jpg]

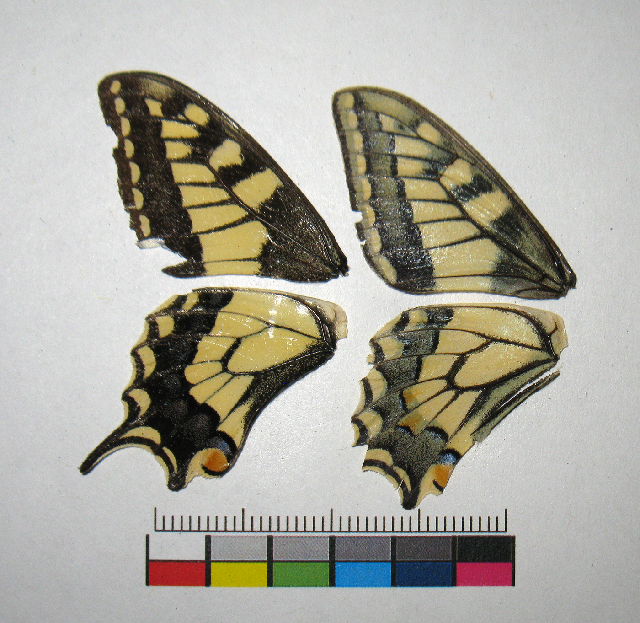

Supplement: S3 Fig — (ZIP) [file pone.0343793.s003.zip › S3/RVcoll.14-H617 .jpeg]

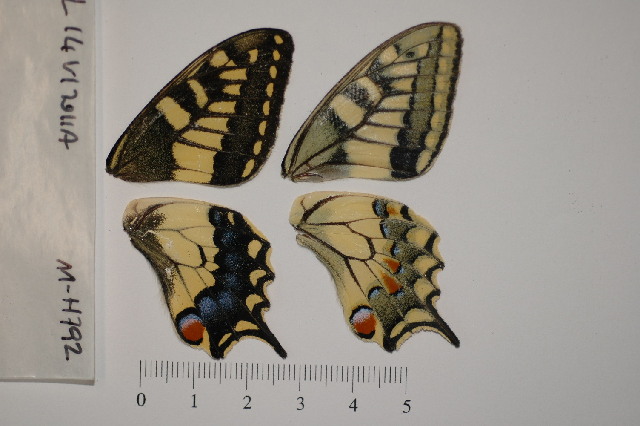

Supplement: S3 Fig — (ZIP) [file pone.0343793.s003.zip › S3/RVcoll.11-H792 .jpeg]

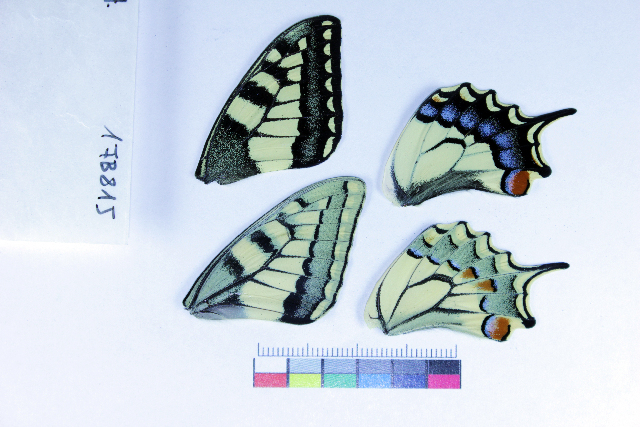

Supplement: S3 Fig — (ZIP) [file pone.0343793.s003.zip › S3/RVcoll17B815.jpeg]

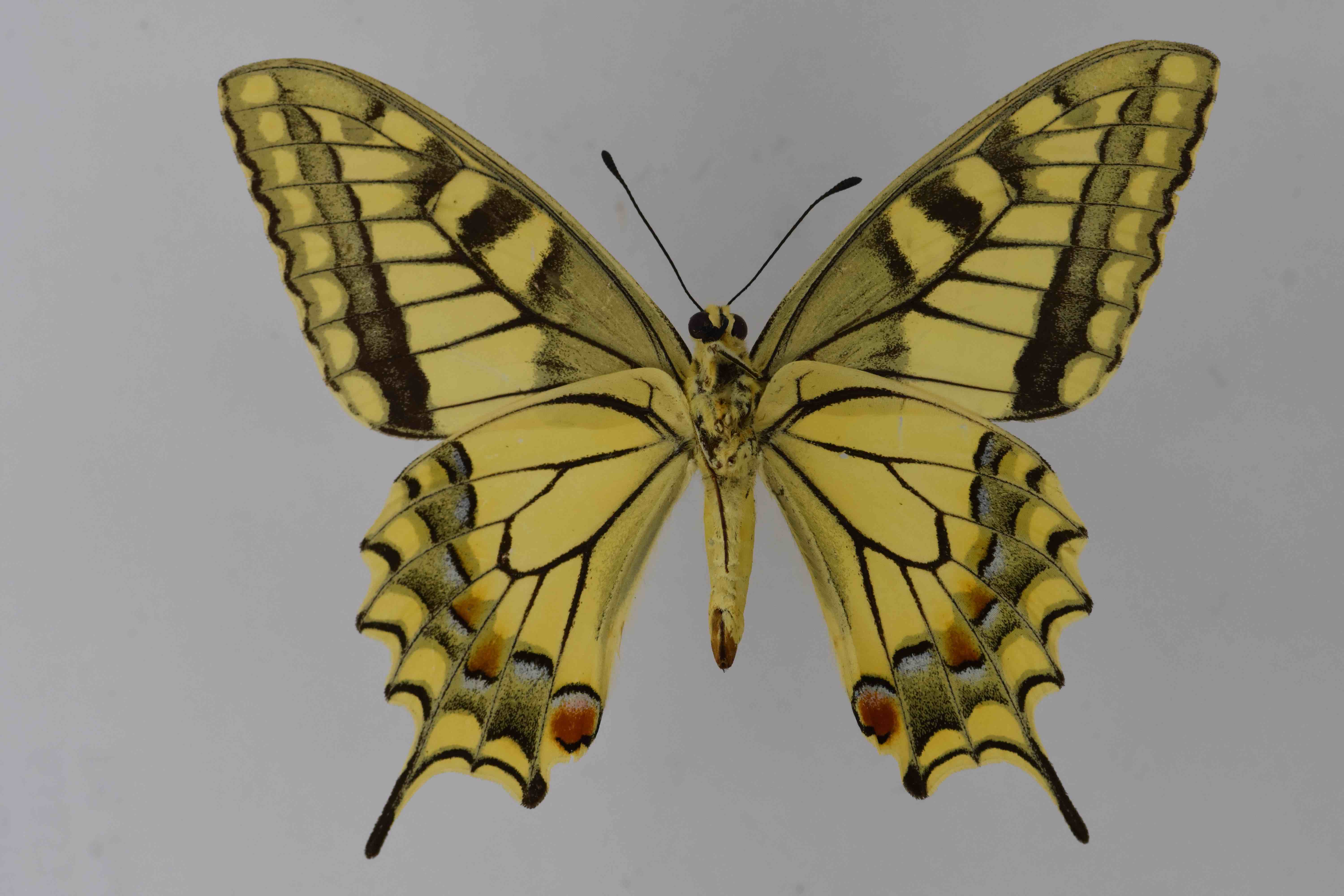

Supplement: S3 Fig — (ZIP) [file pone.0343793.s003.zip › S3/DNAwth015-V copy.jpeg]

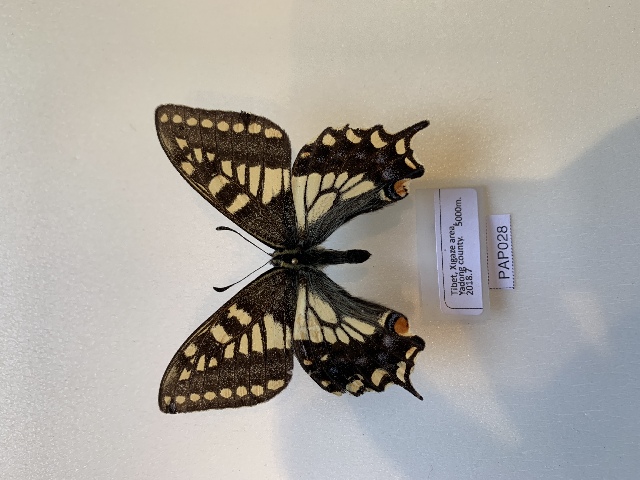

Supplement: S3 Fig — (ZIP) [file pone.0343793.s003.zip › S3/PAP028.jpeg]

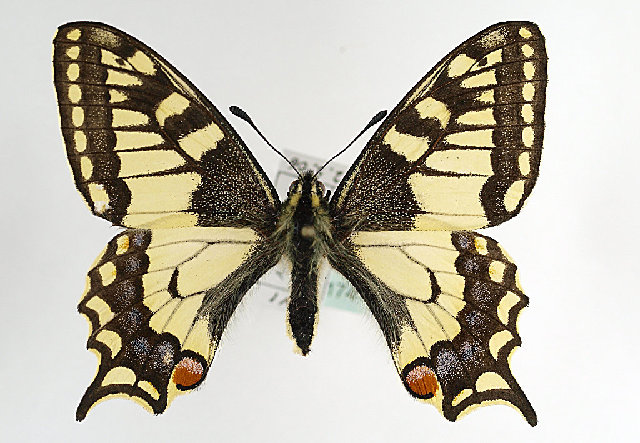

Supplement: S3 Fig — (ZIP) [file pone.0343793.s003.zip › S3/TLMF Lep 14174 .jpg]

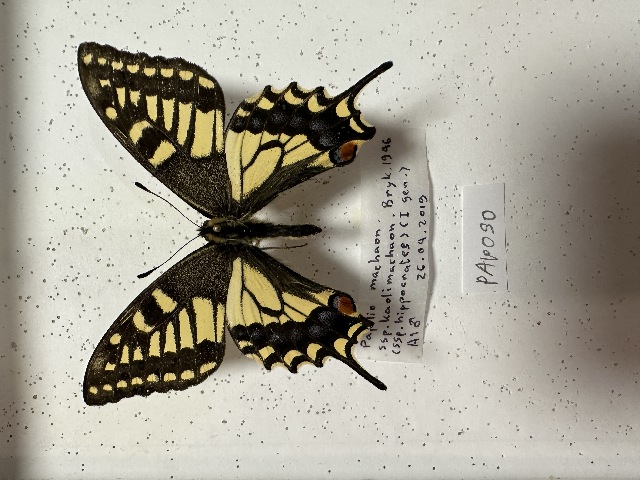

Supplement: S3 Fig — (ZIP) [file pone.0343793.s003.zip › S3/PAP090.jpeg]

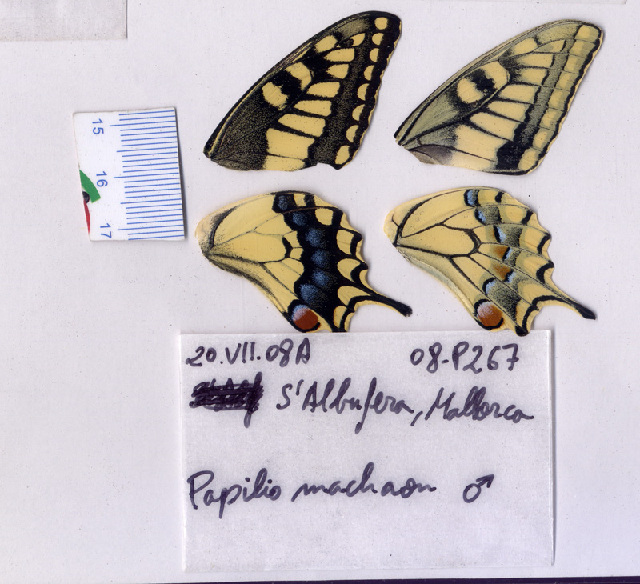

Supplement: S3 Fig — (ZIP) [file pone.0343793.s003.zip › S3/RVcoll.08-P267.jpg]

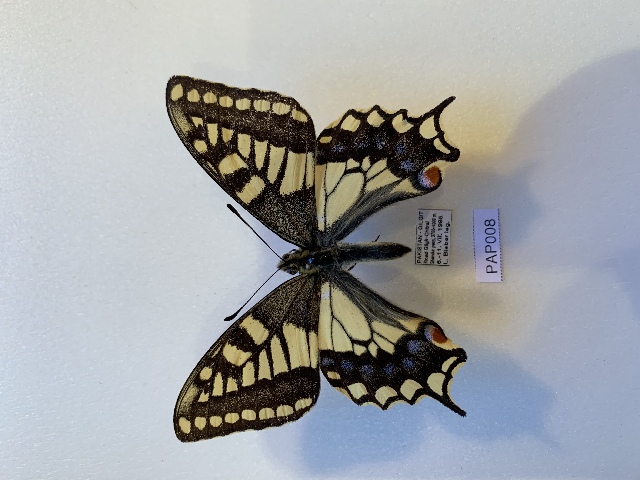

Supplement: S3 Fig — (ZIP) [file pone.0343793.s003.zip › S3/PAP008.jpeg]

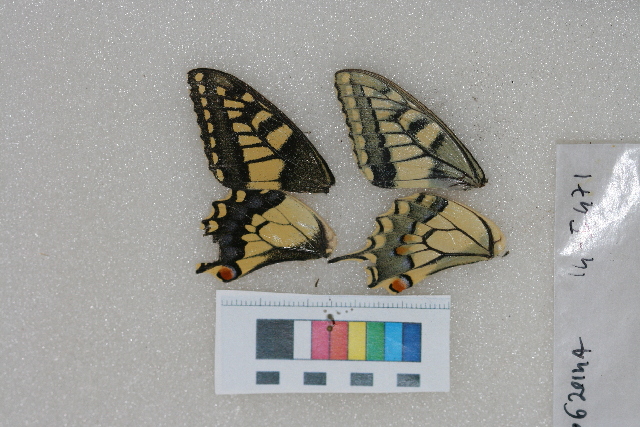

Supplement: S3 Fig — (ZIP) [file pone.0343793.s003.zip › S3/RVcoll.14-I471 .jpeg]

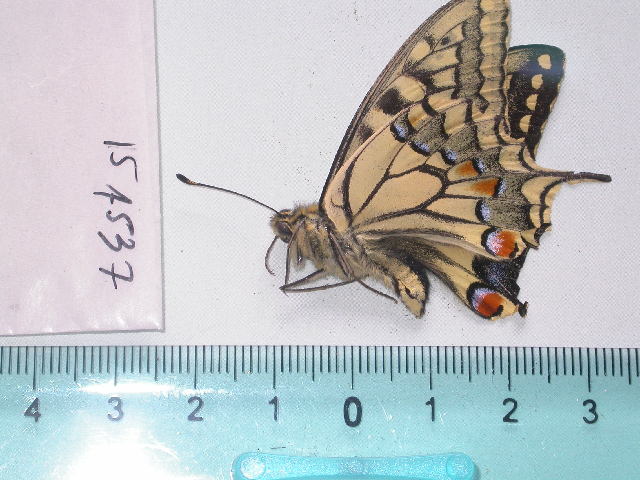

Supplement: S3 Fig — (ZIP) [file pone.0343793.s003.zip › S3/15-A537.jpeg]

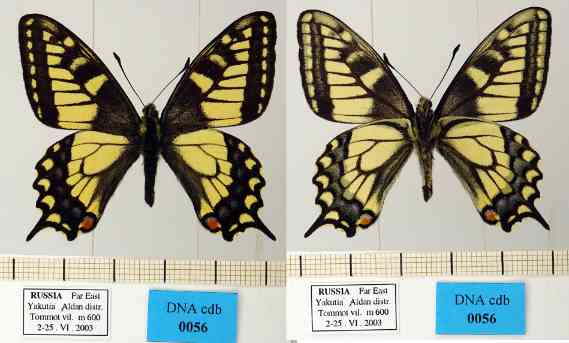

Supplement: S3 Fig — (ZIP) [file pone.0343793.s003.zip › S3/DNAcdb0056 copy.jpg]

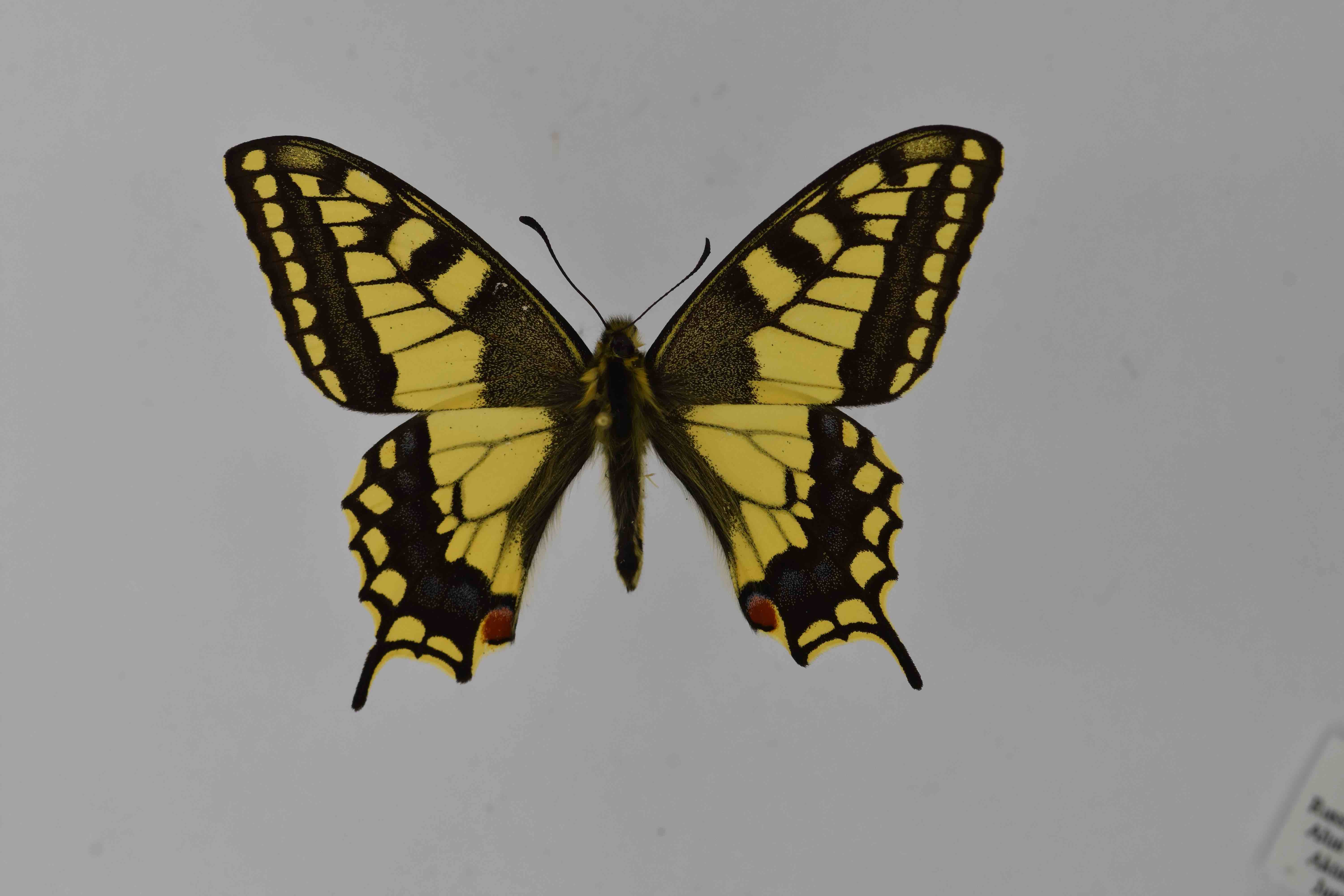

Supplement: S3 Fig — (ZIP) [file pone.0343793.s003.zip › S3/DNAwth016-D copy.jpeg]

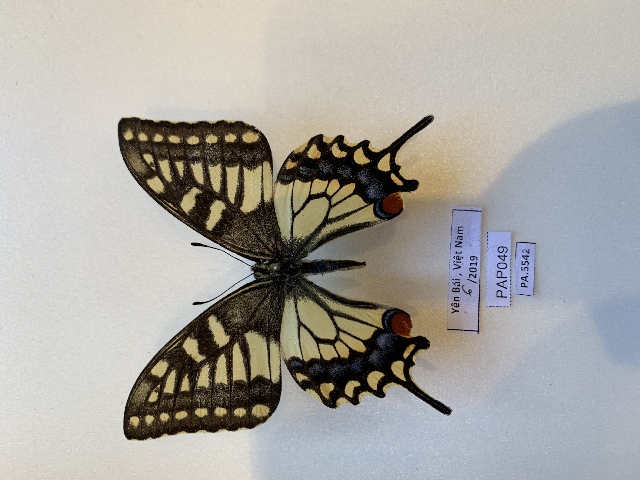

Supplement: S3 Fig — (ZIP) [file pone.0343793.s003.zip › S3/PAP049.jpeg]

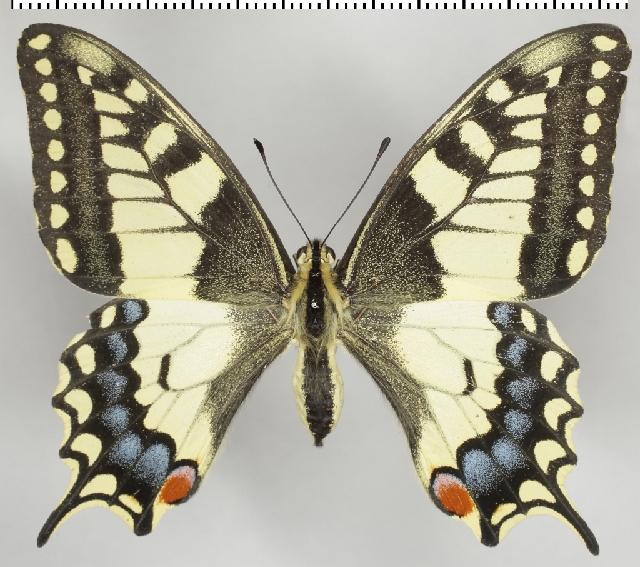

Supplement: S3 Fig — (ZIP) [file pone.0343793.s003.zip › S3/TLMF Lep 21125 .jpg]

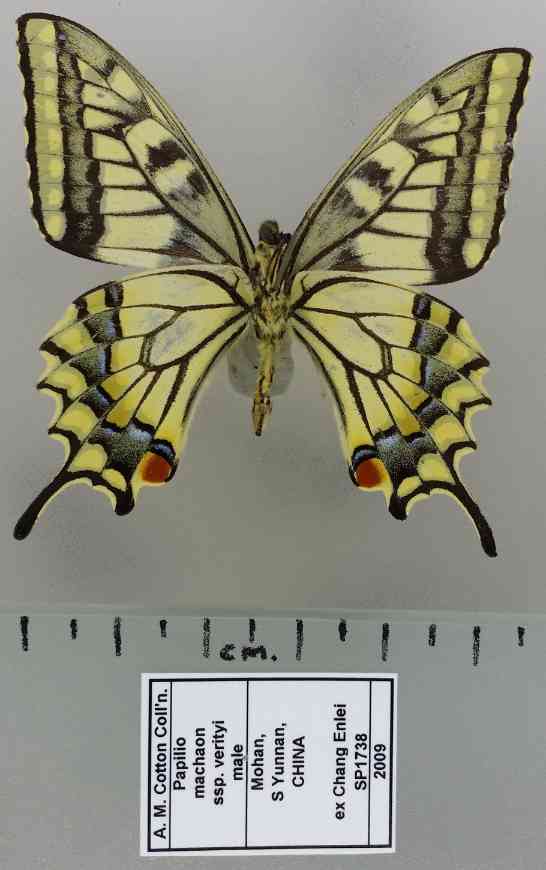

Supplement: S3 Fig — (ZIP) [file pone.0343793.s003.zip › S3/AC-SP1738-V copy.jpg]

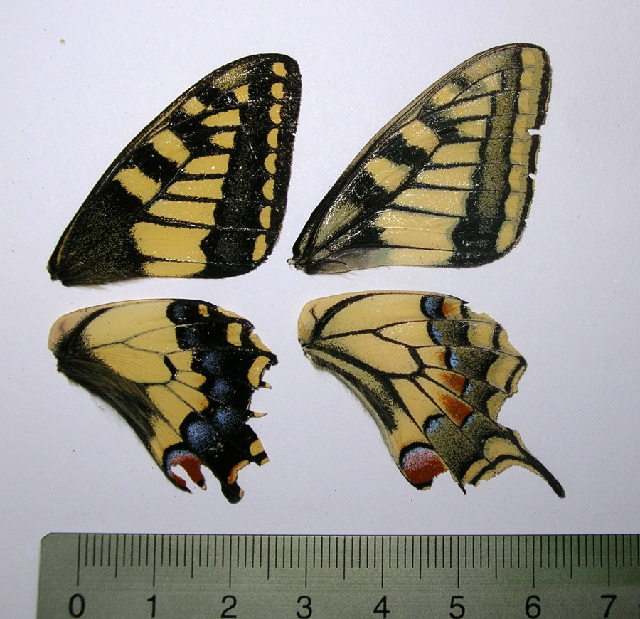

Supplement: S3 Fig — (ZIP) [file pone.0343793.s003.zip › S3/Rvcoll.12-L882 .jpeg]

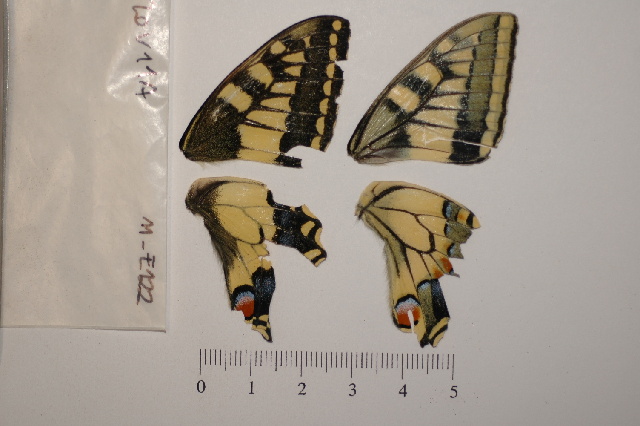

Supplement: S3 Fig — (ZIP) [file pone.0343793.s003.zip › S3/RVcoll.11-E122 .jpeg]

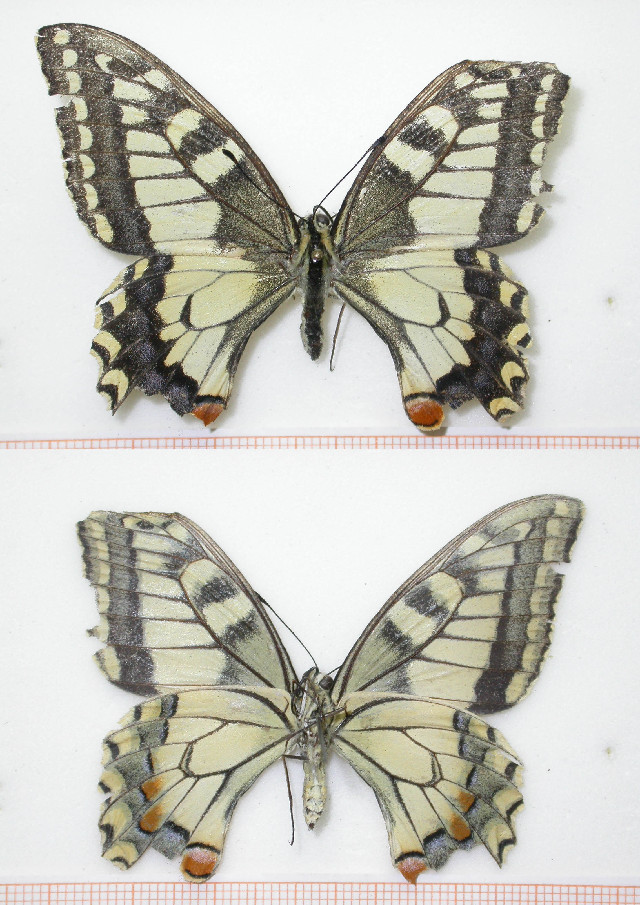

Supplement: S3 Fig — (ZIP) [file pone.0343793.s003.zip › S3/LEP-SS-00142.jpg]

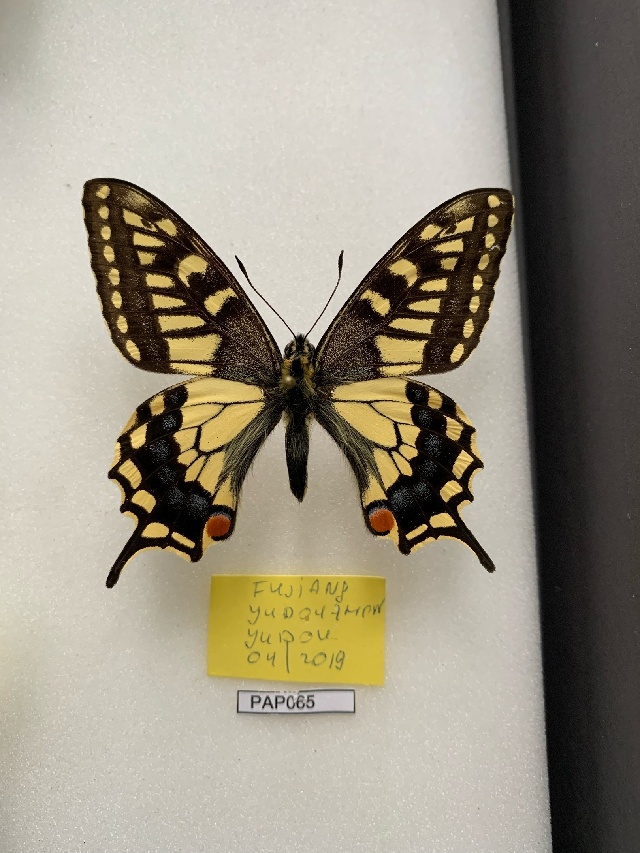

Supplement: S3 Fig — (ZIP) [file pone.0343793.s003.zip › S3/PAP065.jpeg]

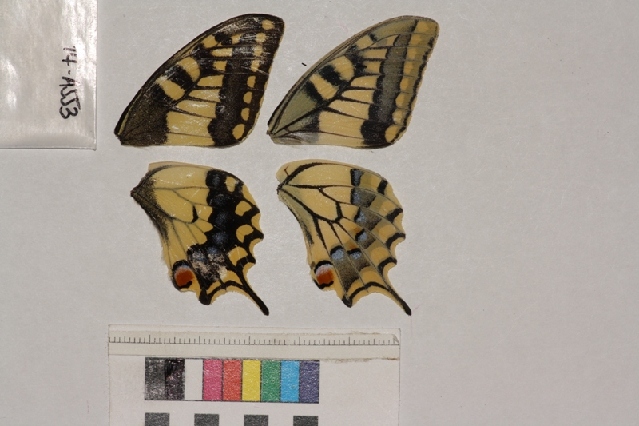

Supplement: S3 Fig — (ZIP) [file pone.0343793.s003.zip › S3/RVcoll.14-A553 .jpg]

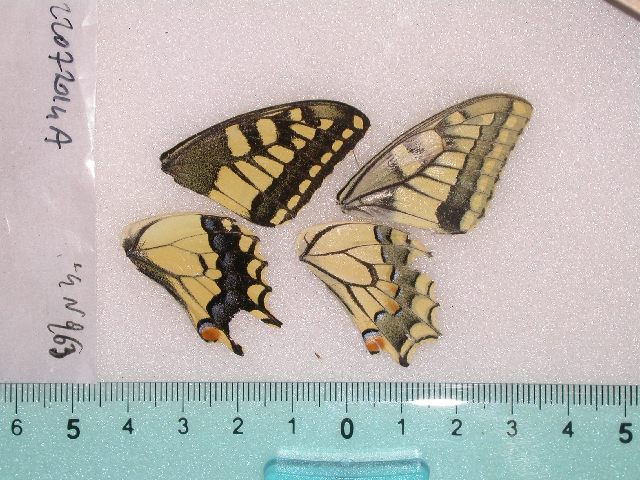

Supplement: S3 Fig — (ZIP) [file pone.0343793.s003.zip › S3/14-N963.jpeg]

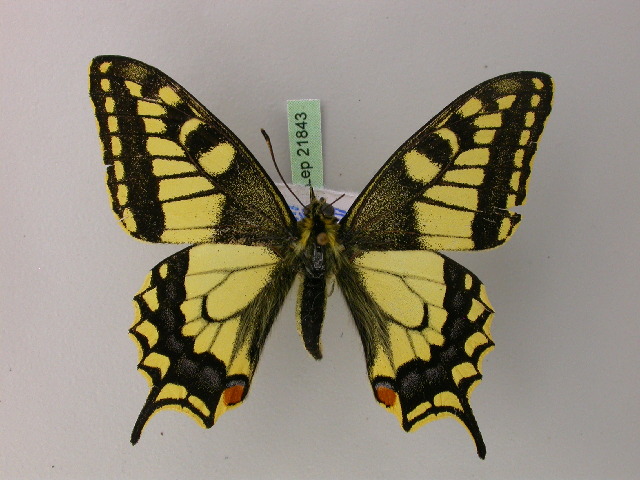

Supplement: S3 Fig — (ZIP) [file pone.0343793.s003.zip › S3/BC ZSM Lep 21843.jpeg]

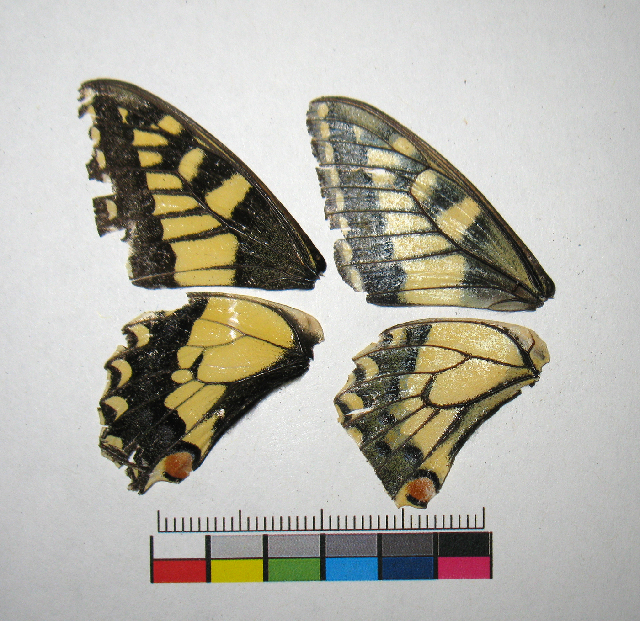

Supplement: S3 Fig — (ZIP) [file pone.0343793.s003.zip › S3/RVcoll.14-F912 .jpeg]

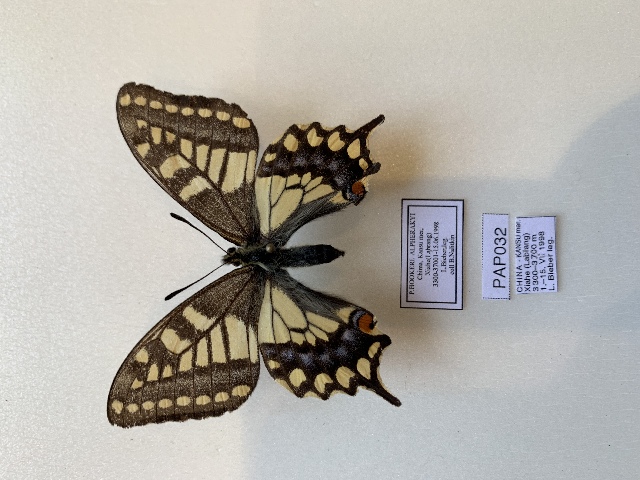

Supplement: S3 Fig — (ZIP) [file pone.0343793.s003.zip › S3/PAP032.jpeg]

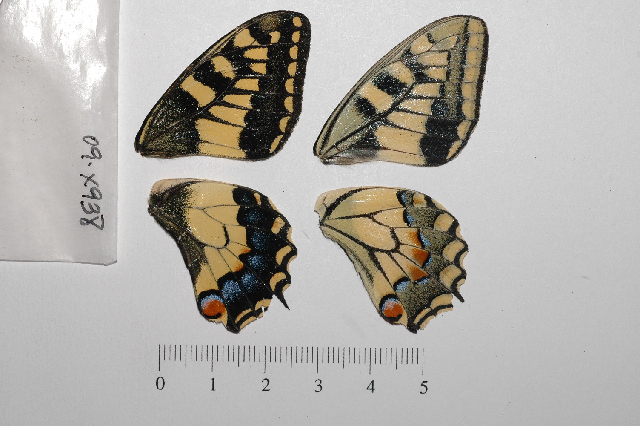

Supplement: S3 Fig — (ZIP) [file pone.0343793.s003.zip › S3/RVcoll.09-X938 .jpg]

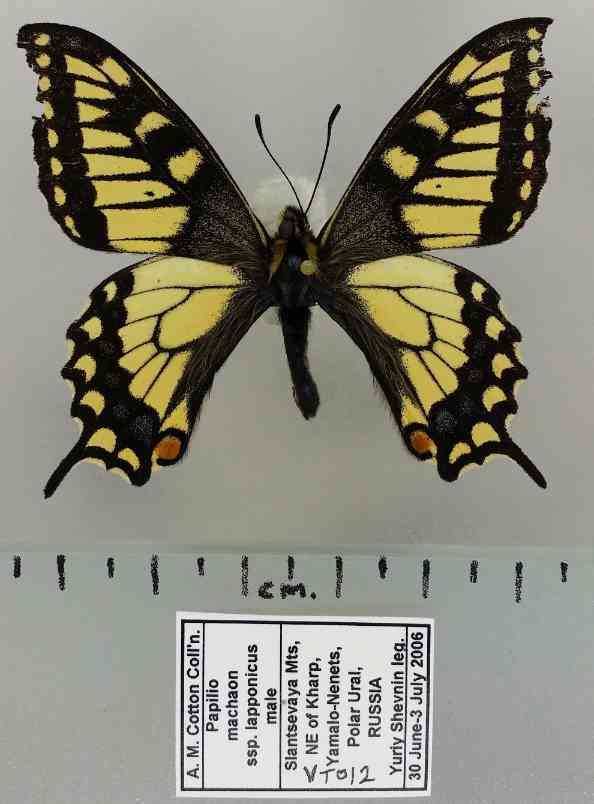

Supplement: S3 Fig — (ZIP) [file pone.0343793.s003.zip › S3/AC-VT012-D copy.jpg]

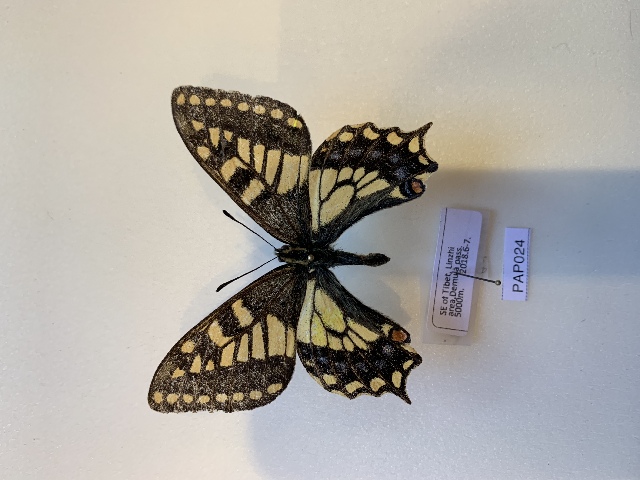

Supplement: S3 Fig — (ZIP) [file pone.0343793.s003.zip › S3/PAP024.jpeg]

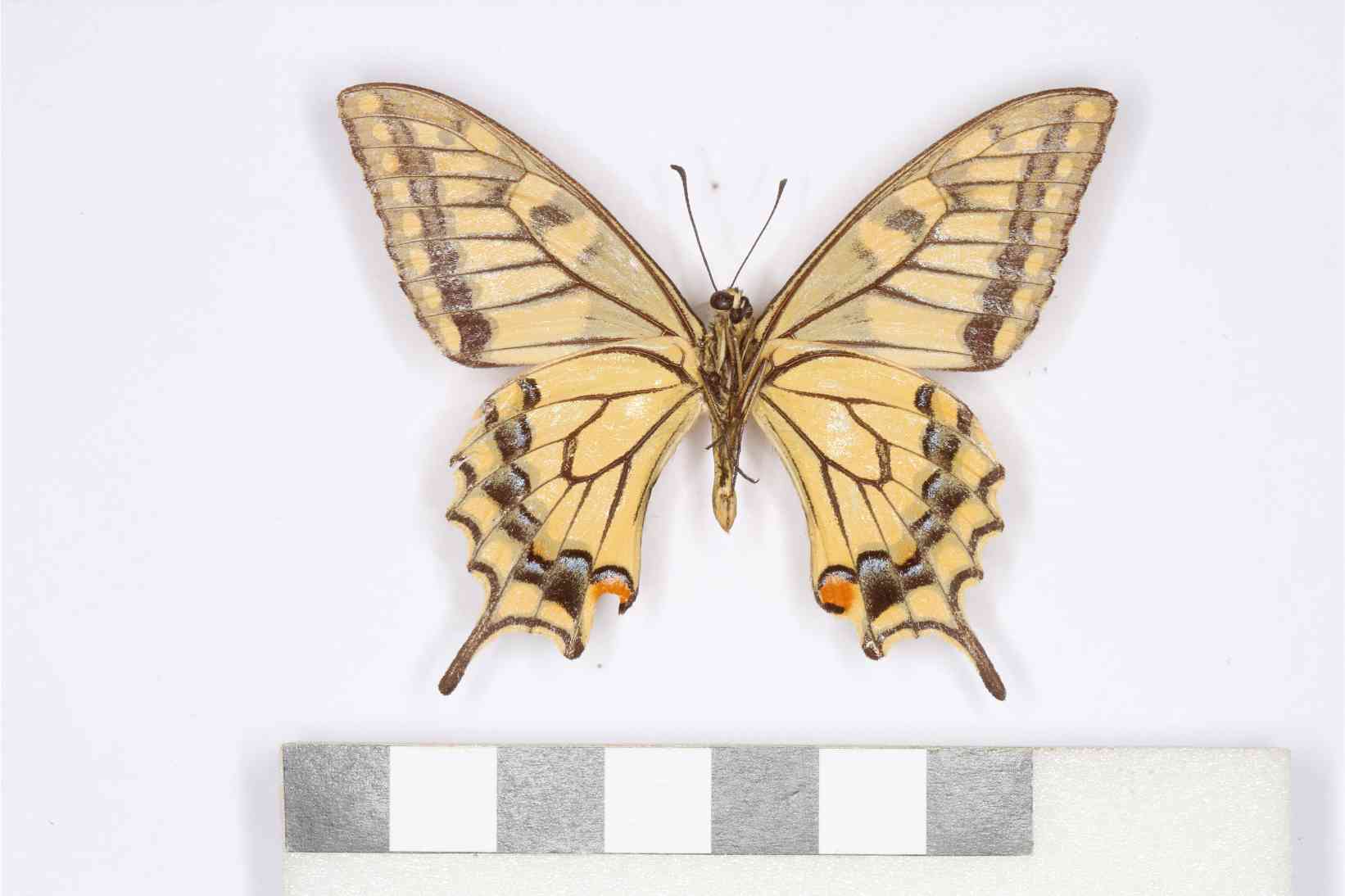

Supplement: S3 Fig — (ZIP) [file pone.0343793.s003.zip › S3/PAP113-V (not the original specimen).jpg]

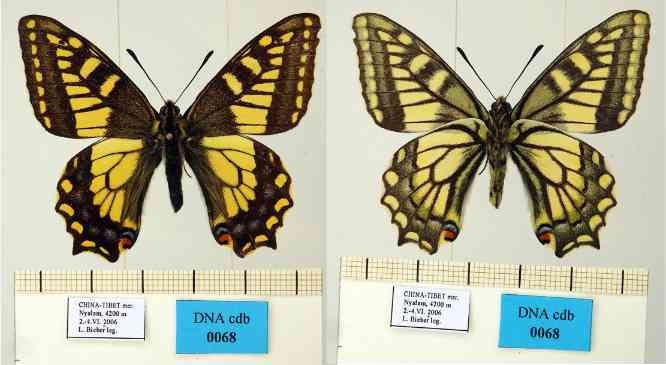

Supplement: S3 Fig — (ZIP) [file pone.0343793.s003.zip › S3/DNAcdb0068 copy.jpg]

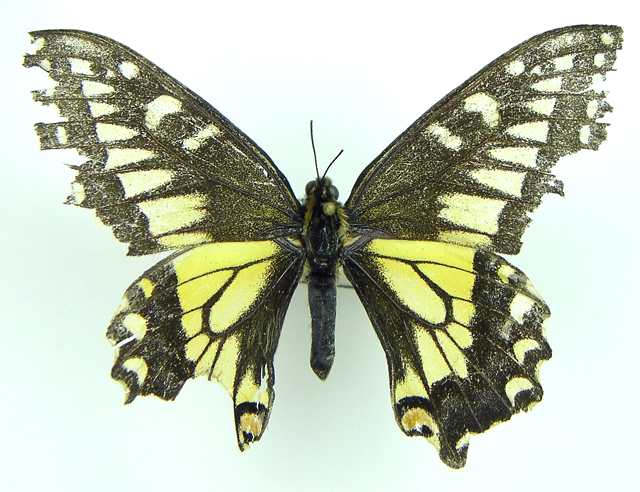

Supplement: S3 Fig — (ZIP) [file pone.0343793.s003.zip › S3/DH000073.jpeg]

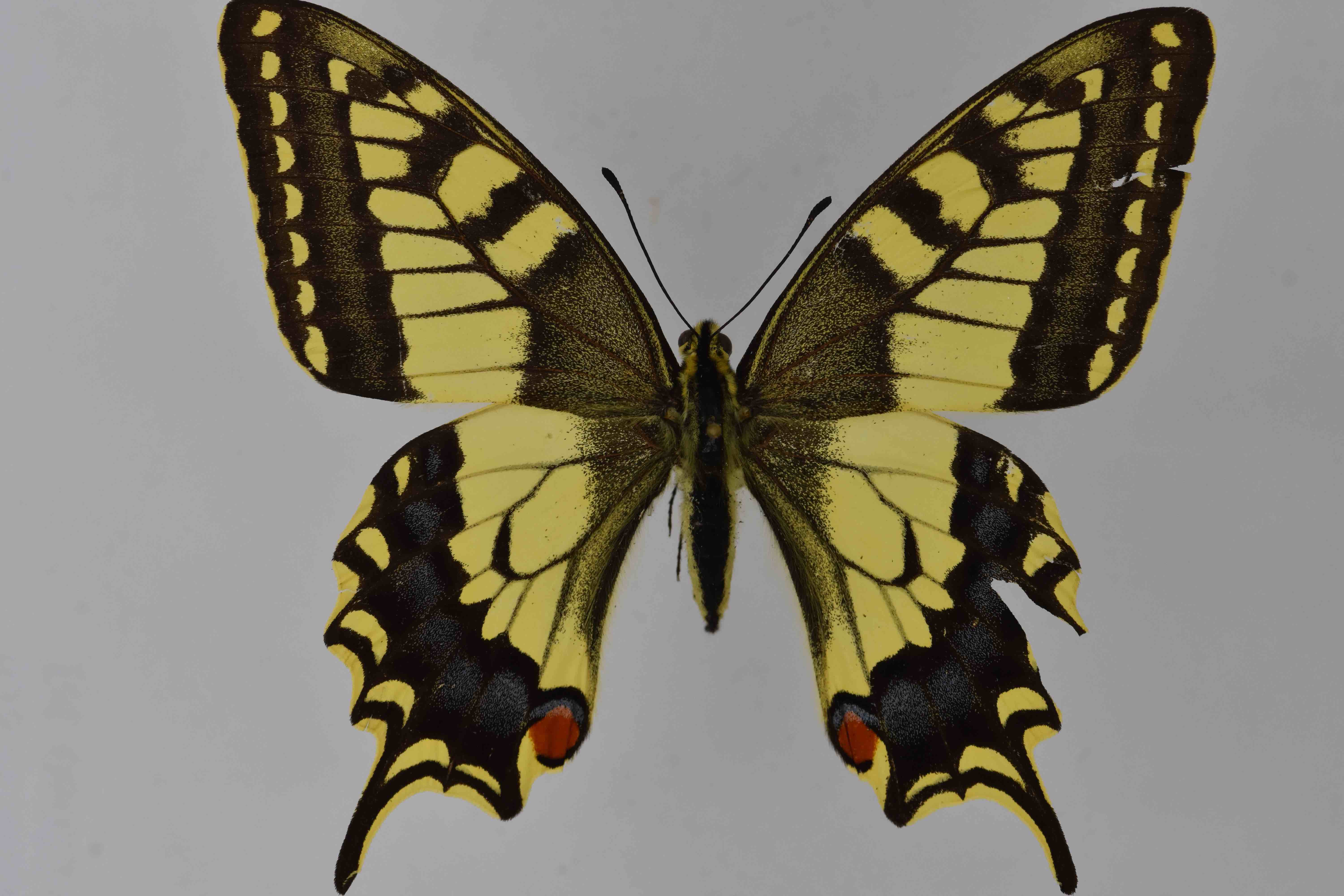

Supplement: S3 Fig — (ZIP) [file pone.0343793.s003.zip › S3/DNAwth024-D copy.jpeg]

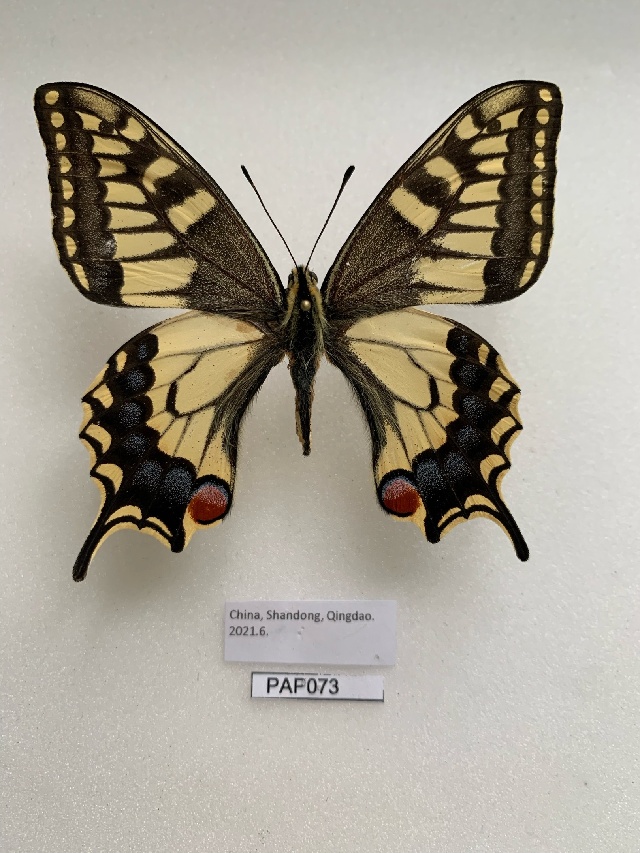

Supplement: S3 Fig — (ZIP) [file pone.0343793.s003.zip › S3/PAP073.jpeg]

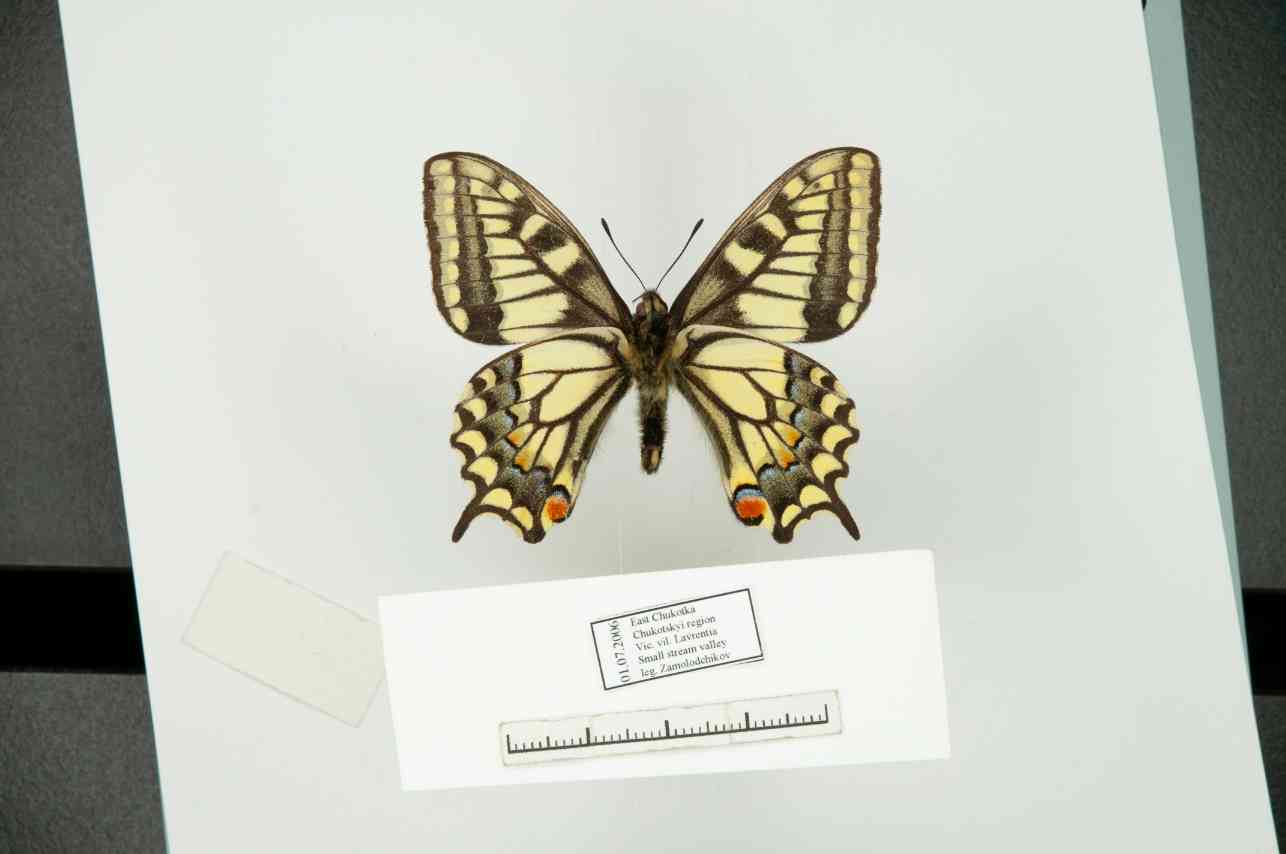

Supplement: S3 Fig — (ZIP) [file pone.0343793.s003.zip › S3/GCB08-V copy.jpg]

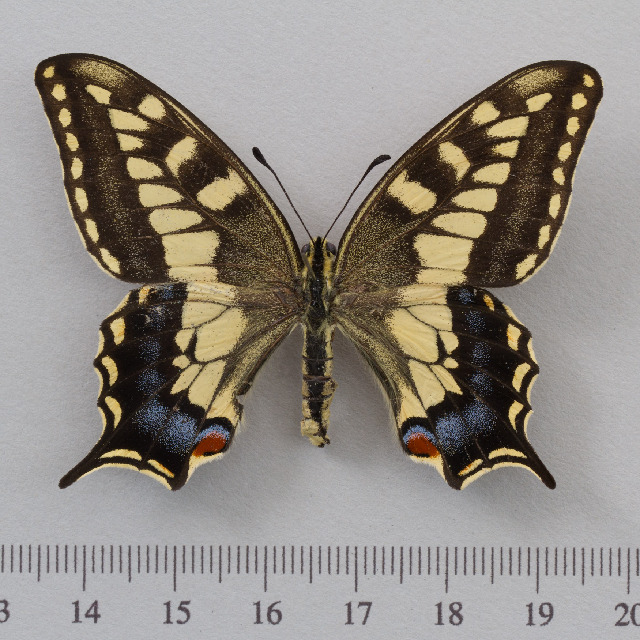

Supplement: S3 Fig — (ZIP) [file pone.0343793.s003.zip › S3/RVcoll.14-O166-D.jpg]

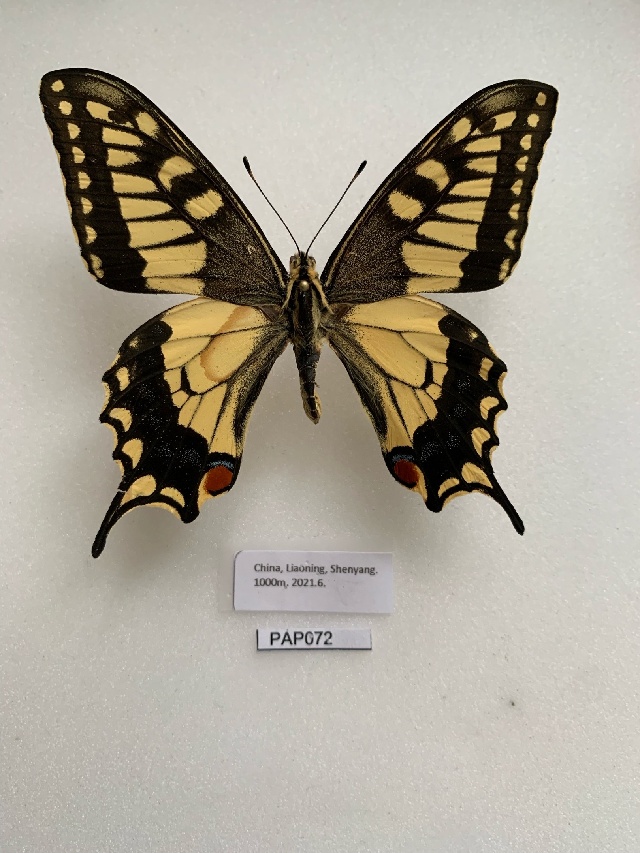

Supplement: S3 Fig — (ZIP) [file pone.0343793.s003.zip › S3/PAP072.jpeg]

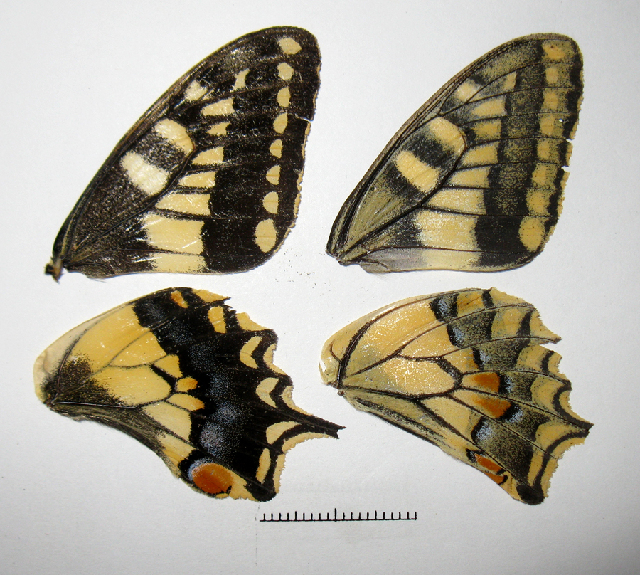

Supplement: S3 Fig — (ZIP) [file pone.0343793.s003.zip › S3/RVcoll.11-H866 .jpeg]

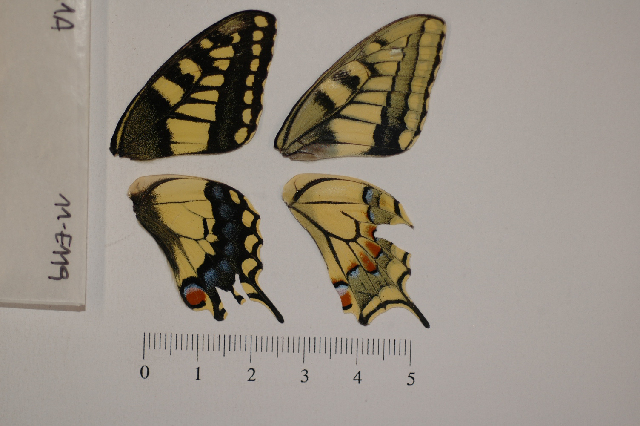

Supplement: S3 Fig — (ZIP) [file pone.0343793.s003.zip › S3/RVcoll.11-E119 .jpg]
