# Supplementary figures and images for: Integrative analyses reveal the evolution of the Old World Swallowtail in the Palearctic (part 1 of 3)
Source: PLoS One. 2026 Jul 8;21(7):e0343793. doi: 10.1371/journal.pone.0343793 (PMC13345299; doi:10.1371/journal.pone.0343793)

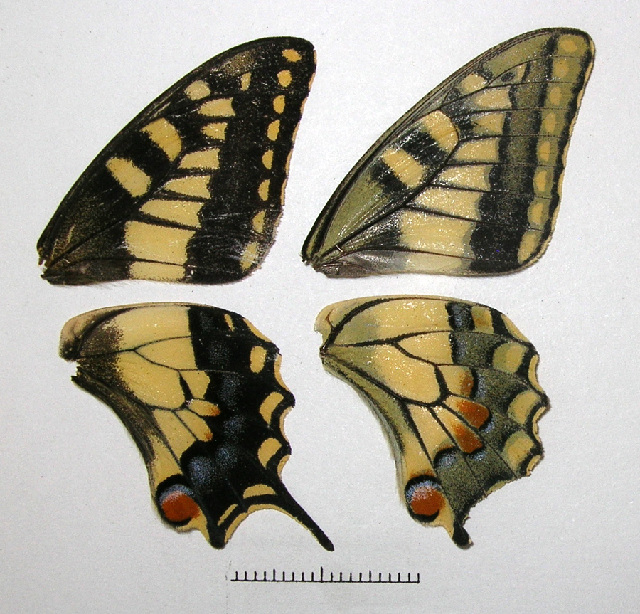

Supplement: S3 Fig — (ZIP) [file pone.0343793.s003.zip › S3/RVcoll.11-H791 .jpeg]

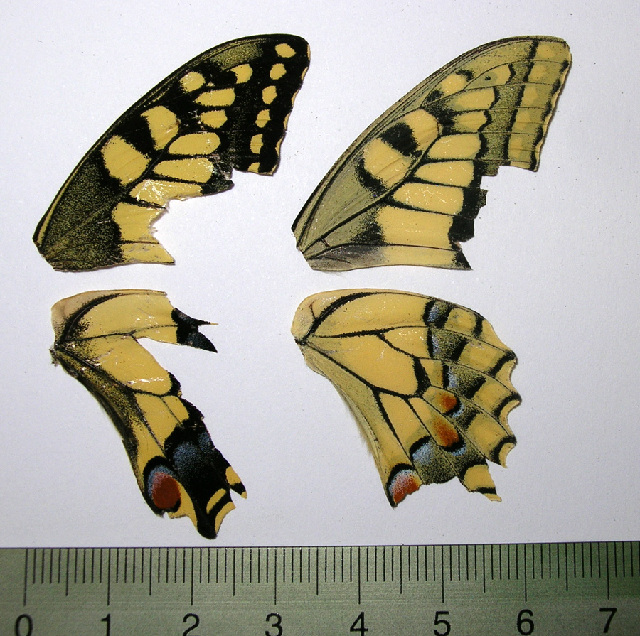

Supplement: S3 Fig — (ZIP) [file pone.0343793.s003.zip › S3/Rvcoll.12-M963 .jpeg]

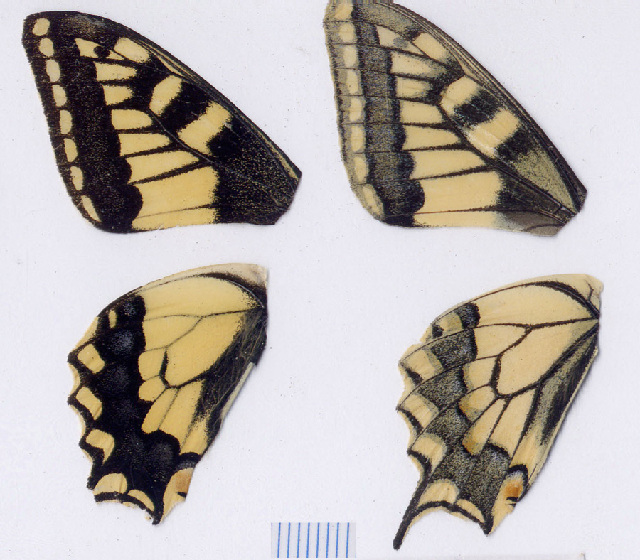

Supplement: S3 Fig — (ZIP) [file pone.0343793.s003.zip › S3/RVcoll.08-J361.jpg]

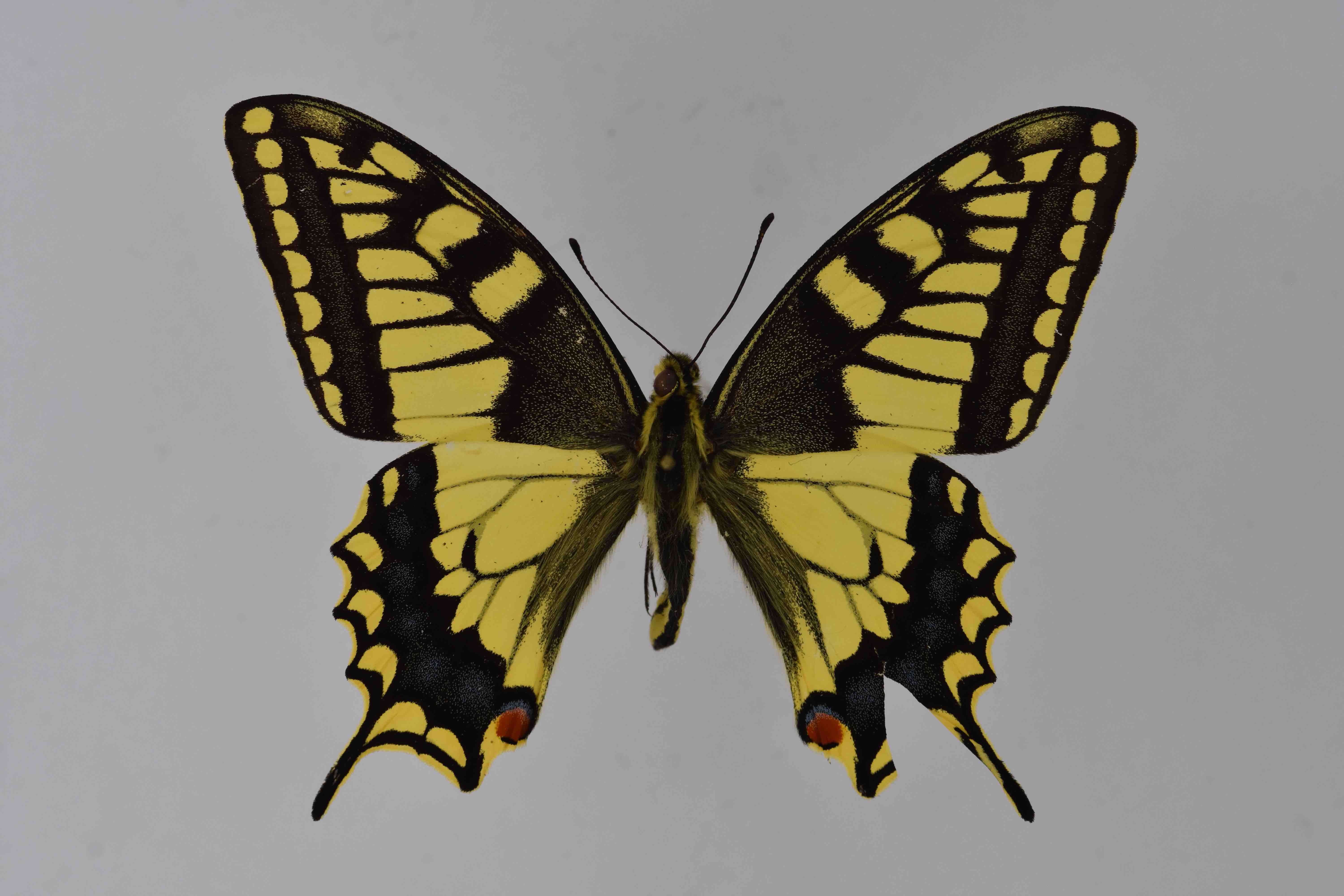

Supplement: S3 Fig — (ZIP) [file pone.0343793.s003.zip › S3/DNAwth005-D copy.jpeg]

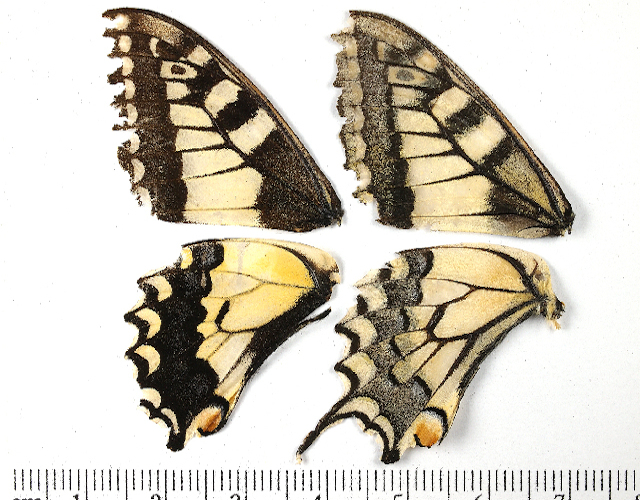

Supplement: S3 Fig — (ZIP) [file pone.0343793.s003.zip › S3/RVcoll15H201 .jpeg]

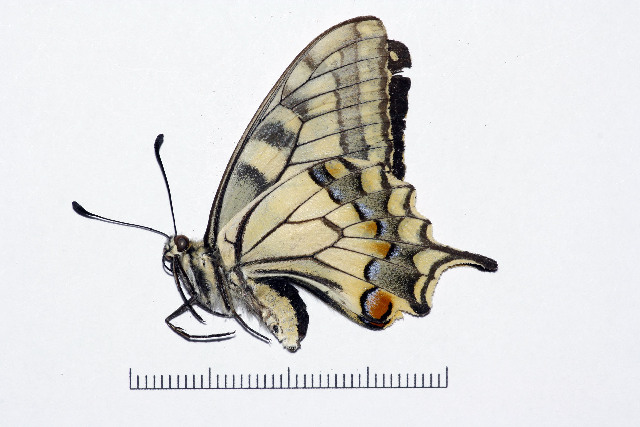

Supplement: S3 Fig — (ZIP) [file pone.0343793.s003.zip › S3/RVcoll16I684 .jpeg]

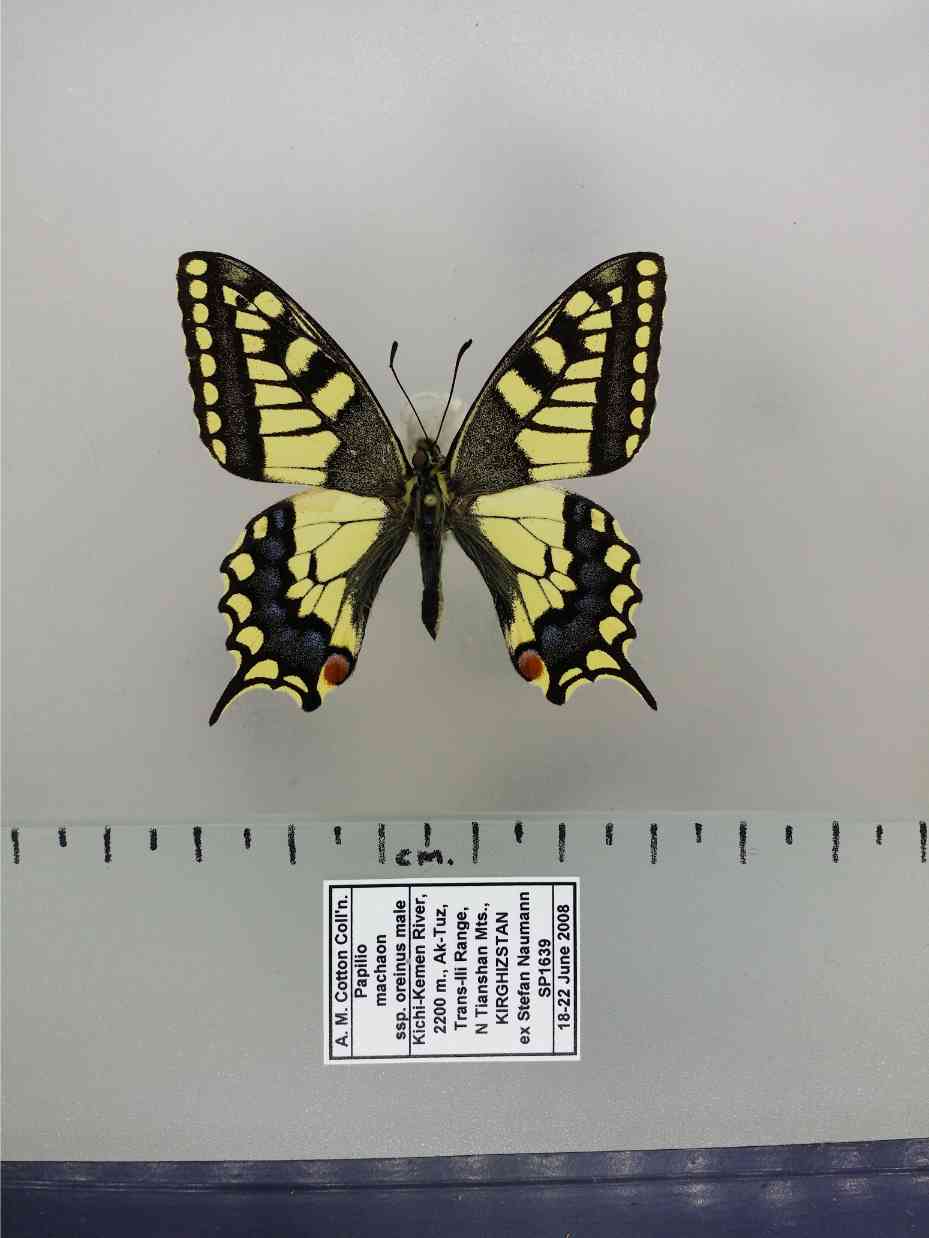

Supplement: S3 Fig — (ZIP) [file pone.0343793.s003.zip › S3/AC-SP1639D copy.jpg]

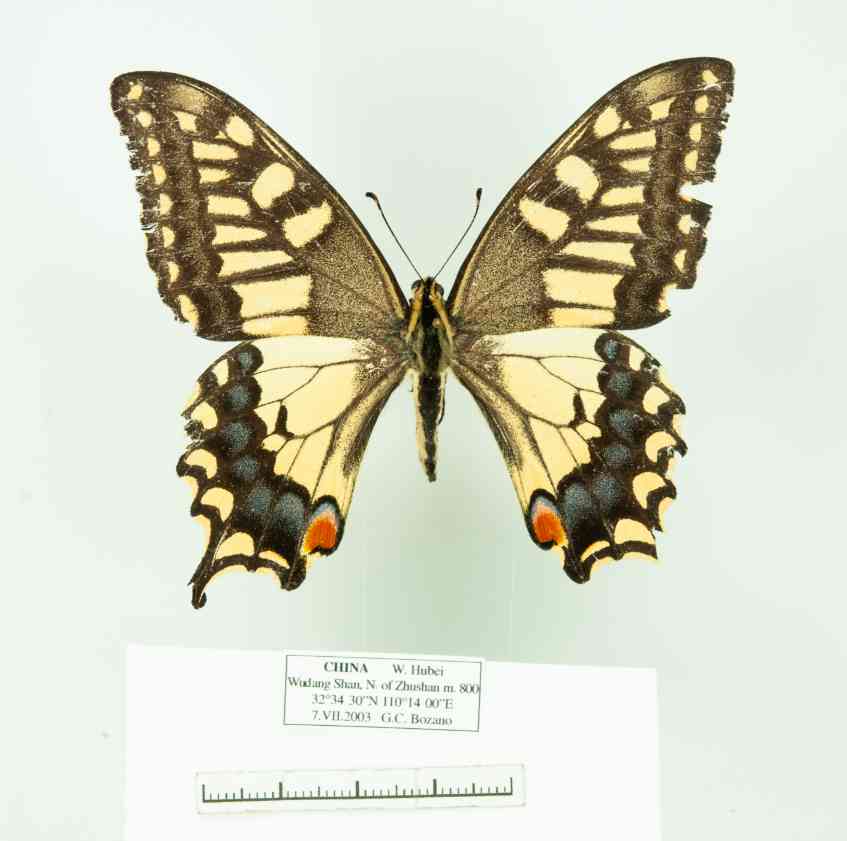

Supplement: S3 Fig — (ZIP) [file pone.0343793.s003.zip › S3/GCB05-D copy.jpg]

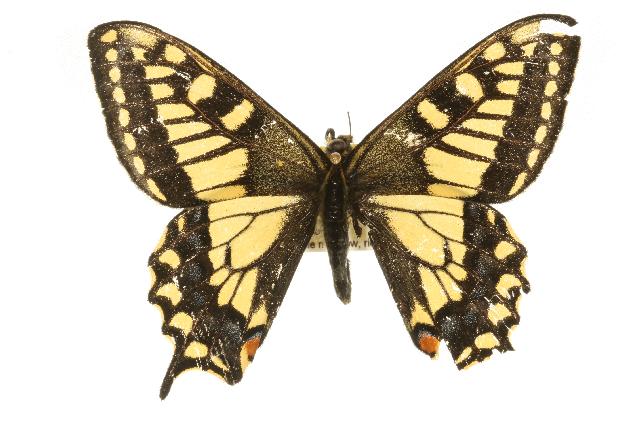

Supplement: S3 Fig — (ZIP) [file pone.0343793.s003.zip › S3/EZ1197CNC.jpeg]

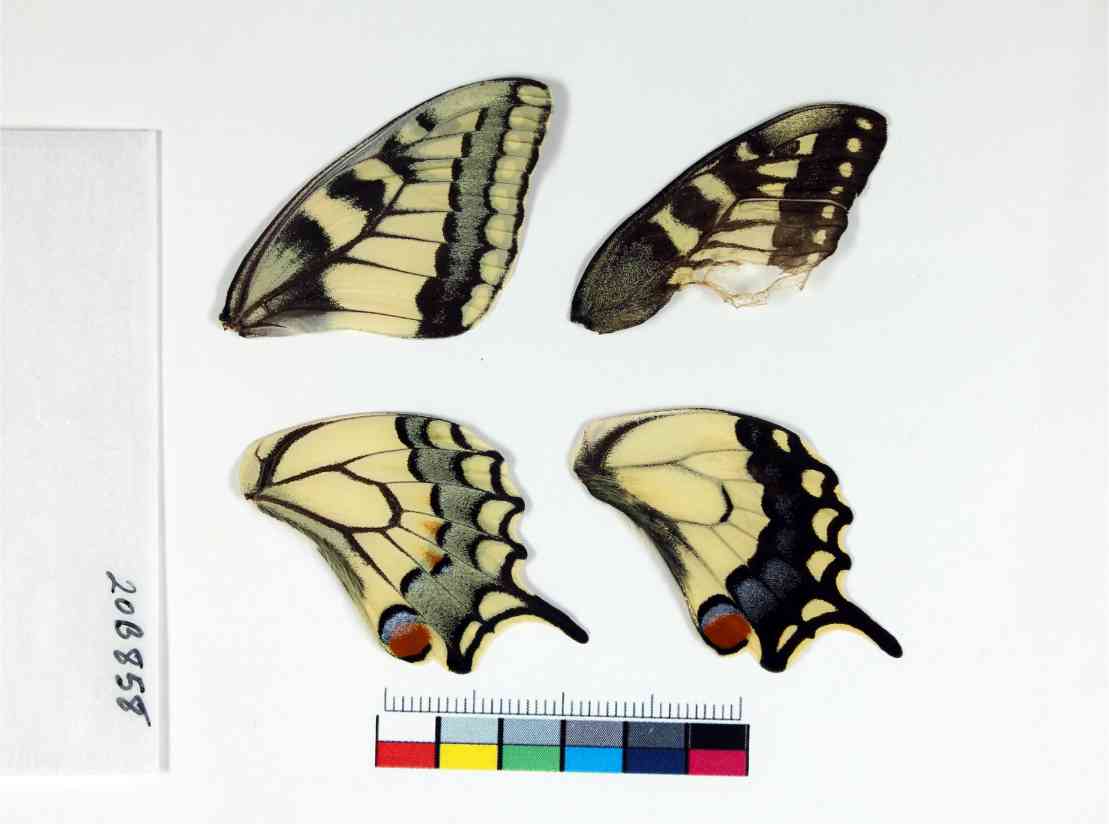

Supplement: S3 Fig — (ZIP) [file pone.0343793.s003.zip › S3/AC-PQ020 copy.jpg]

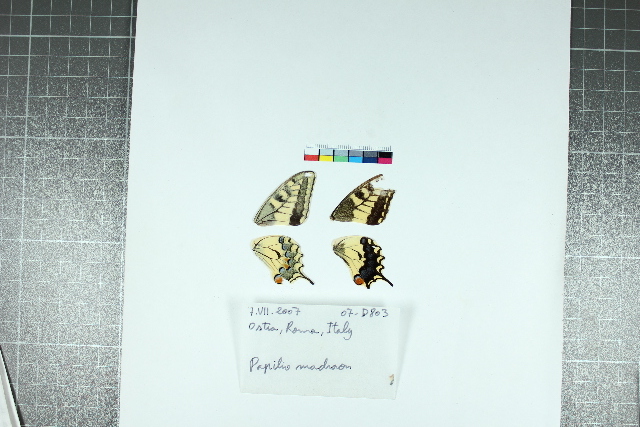

Supplement: S3 Fig — (ZIP) [file pone.0343793.s003.zip › S3/RVcoll07D803 .jpeg]

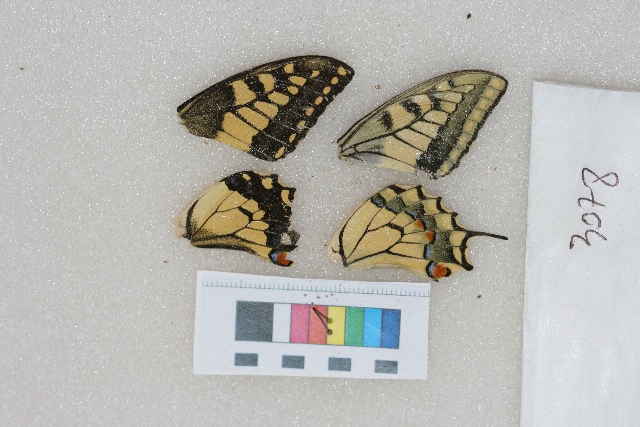

Supplement: S3 Fig — (ZIP) [file pone.0343793.s003.zip › S3/RVcoll.LD-3078 .jpeg]

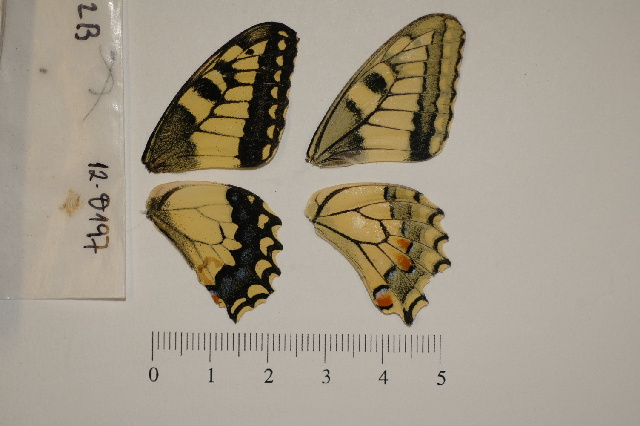

Supplement: S3 Fig — (ZIP) [file pone.0343793.s003.zip › S3/RVcoll.12-O197 .jpeg]

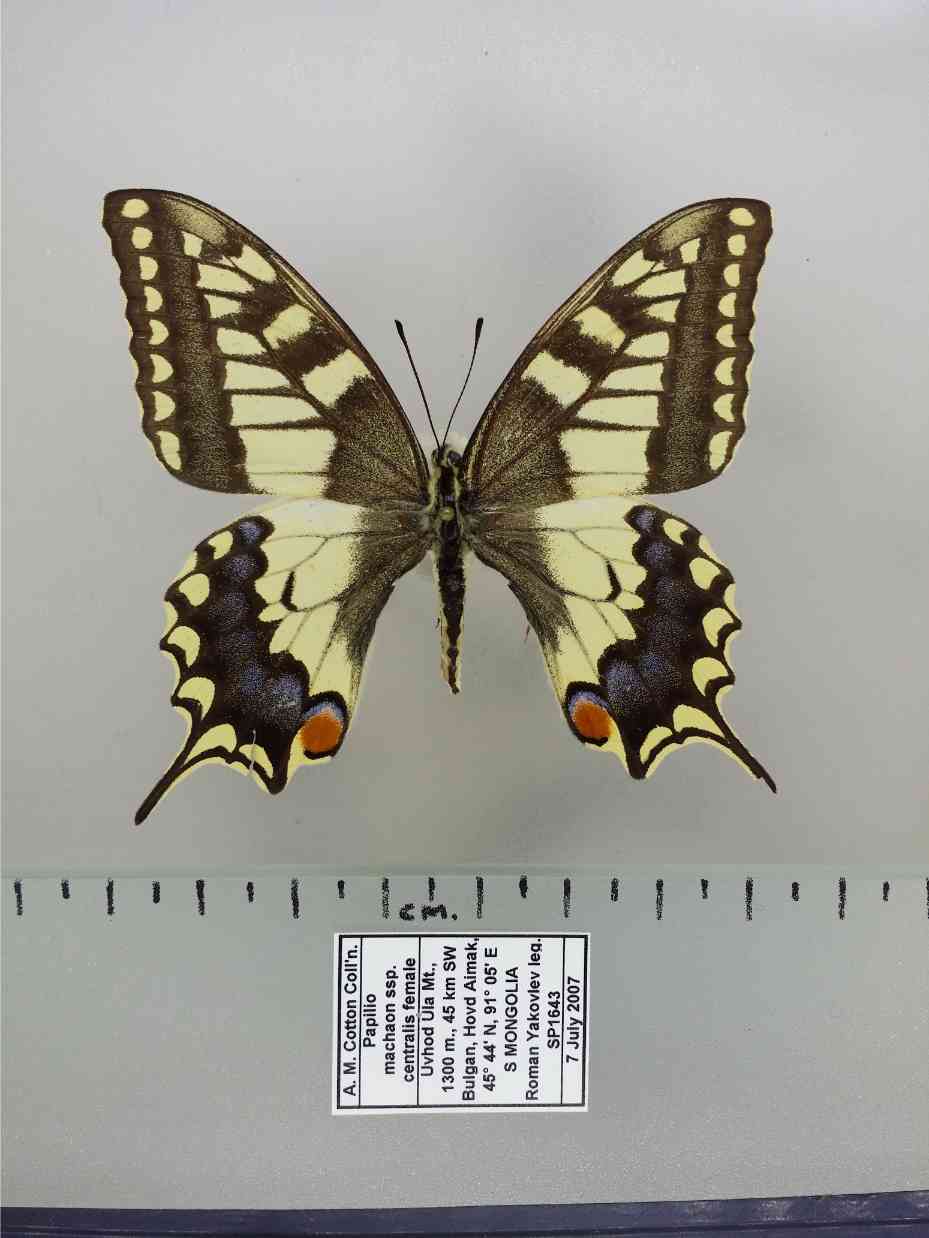

Supplement: S3 Fig — (ZIP) [file pone.0343793.s003.zip › S3/AC-SP1643D copy.jpg]

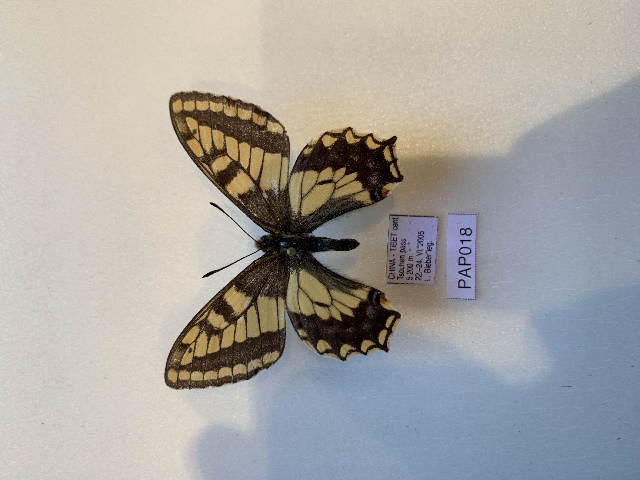

Supplement: S3 Fig — (ZIP) [file pone.0343793.s003.zip › S3/PAP018.jpeg]

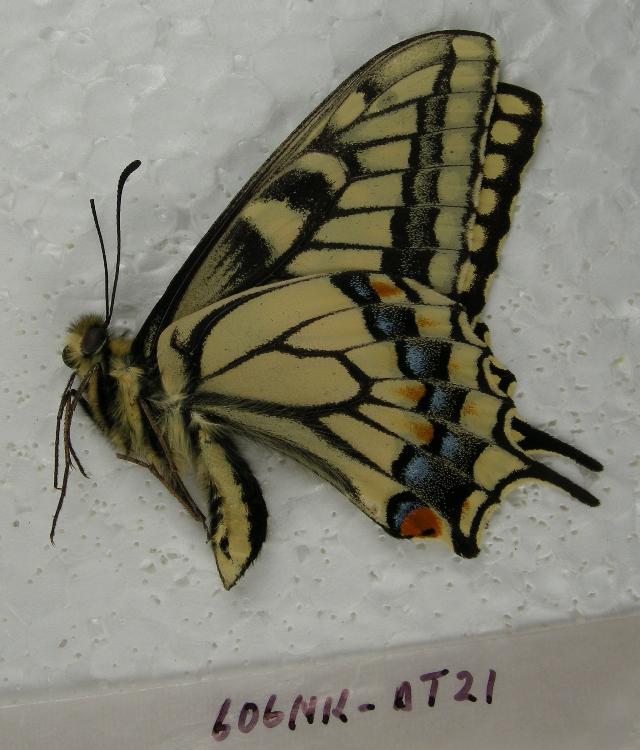

Supplement: S3 Fig — (ZIP) [file pone.0343793.s003.zip › S3/606NK-DT21.jpeg]

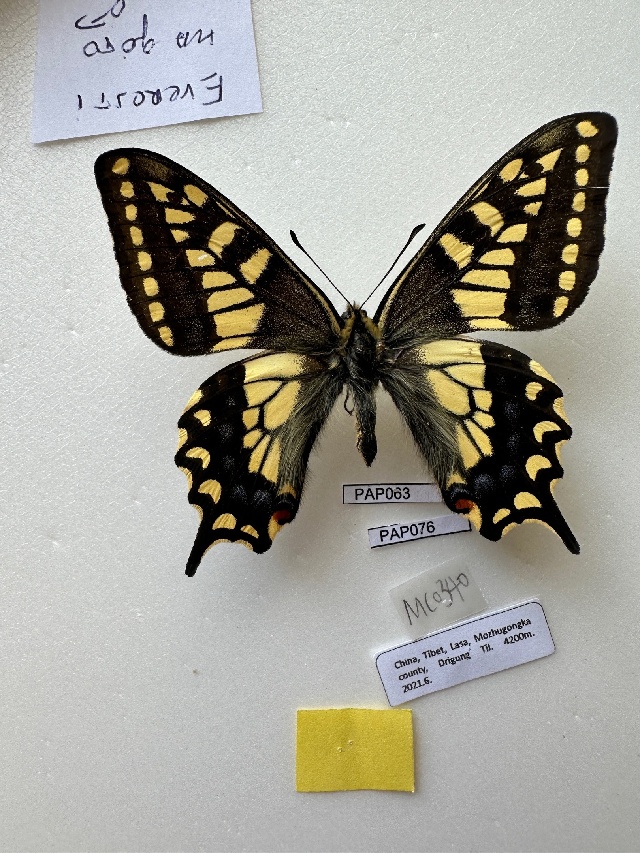

Supplement: S3 Fig — (ZIP) [file pone.0343793.s003.zip › S3/PAP063.jpeg]

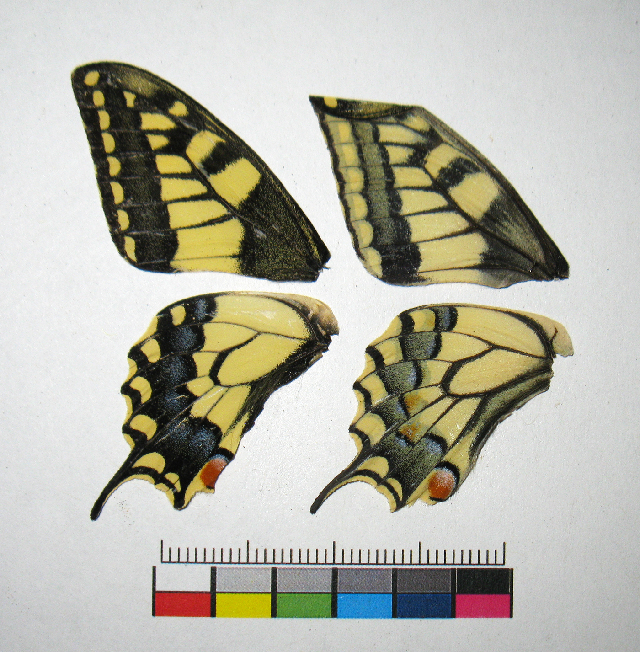

Supplement: S3 Fig — (ZIP) [file pone.0343793.s003.zip › S3/RVcoll.14-H384 .jpeg]

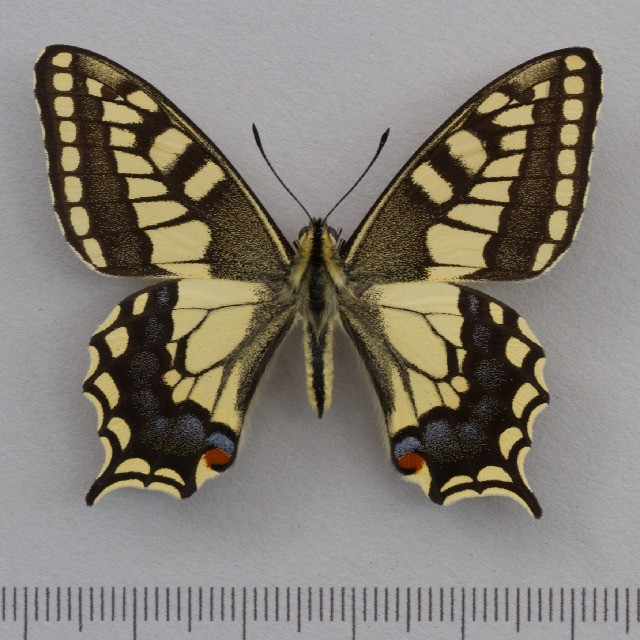

Supplement: S3 Fig — (ZIP) [file pone.0343793.s003.zip › S3/RVcoll.14-O167-D.jpg]

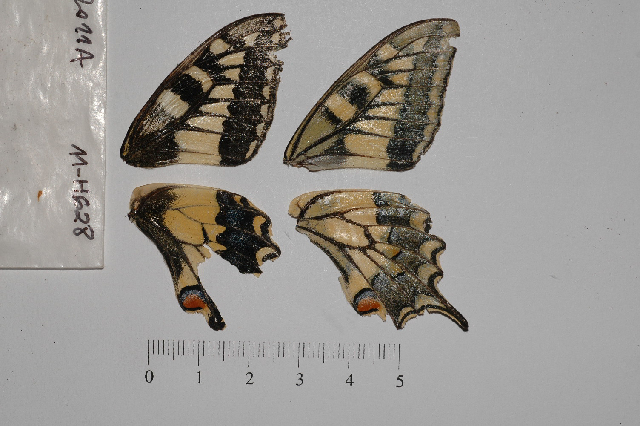

Supplement: S3 Fig — (ZIP) [file pone.0343793.s003.zip › S3/RVcoll.11-H628 .jpg]

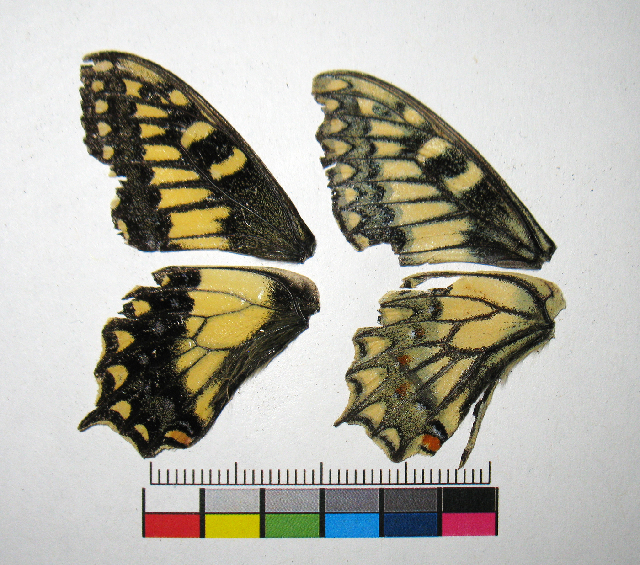

Supplement: S3 Fig — (ZIP) [file pone.0343793.s003.zip › S3/RVcoll.13-U519 .jpeg]

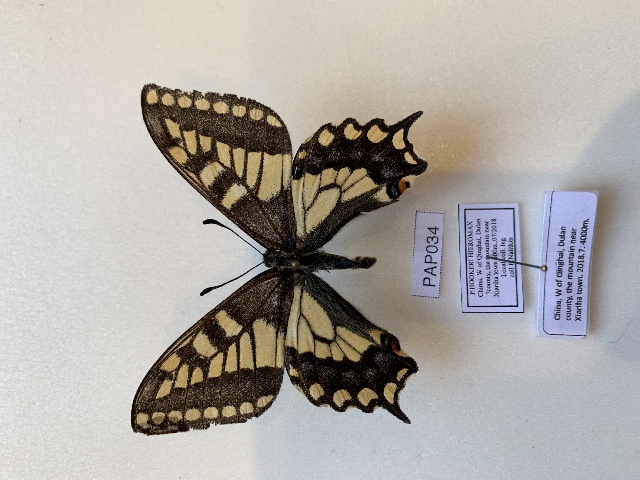

Supplement: S3 Fig — (ZIP) [file pone.0343793.s003.zip › S3/PAP034.jpeg]

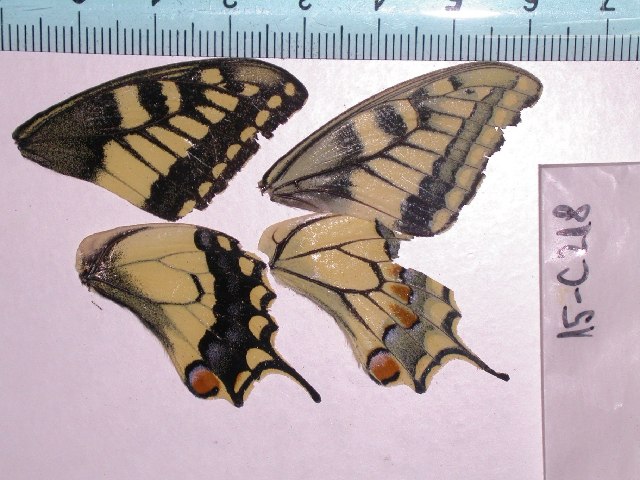

Supplement: S3 Fig — (ZIP) [file pone.0343793.s003.zip › S3/15-C218.jpeg]

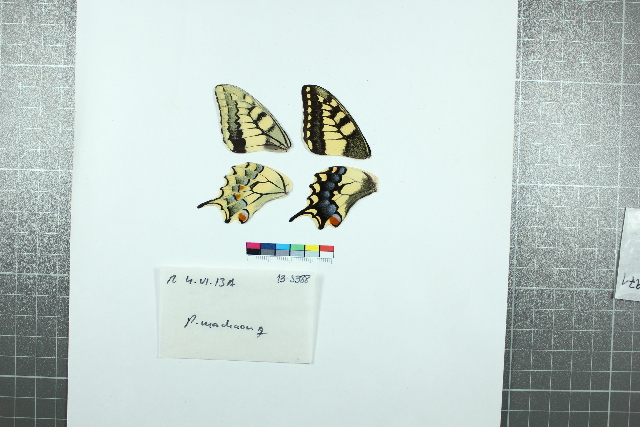

Supplement: S3 Fig — (ZIP) [file pone.0343793.s003.zip › S3/RVcoll13S388 .jpeg]

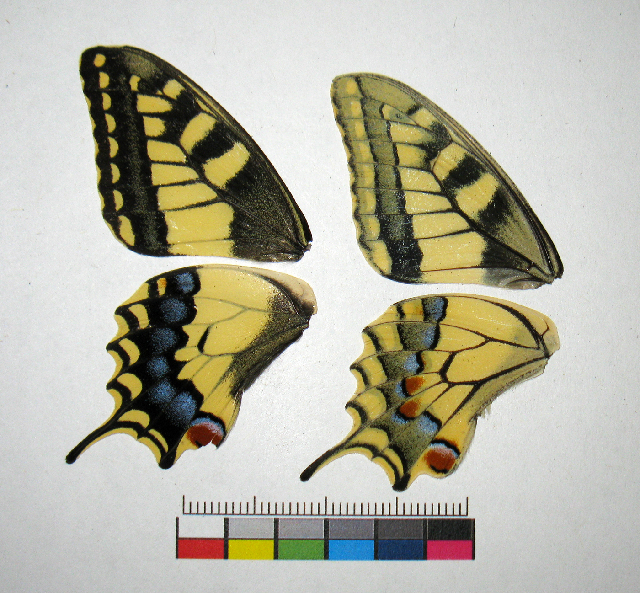

Supplement: S3 Fig — (ZIP) [file pone.0343793.s003.zip › S3/15-M986.jpeg]

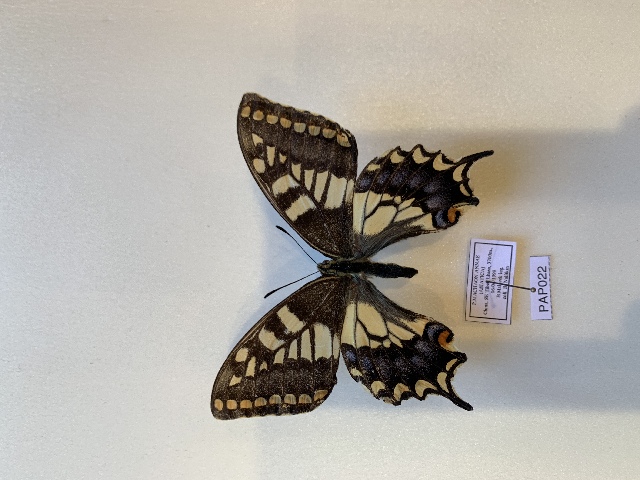

Supplement: S3 Fig — (ZIP) [file pone.0343793.s003.zip › S3/PAP022.jpeg]

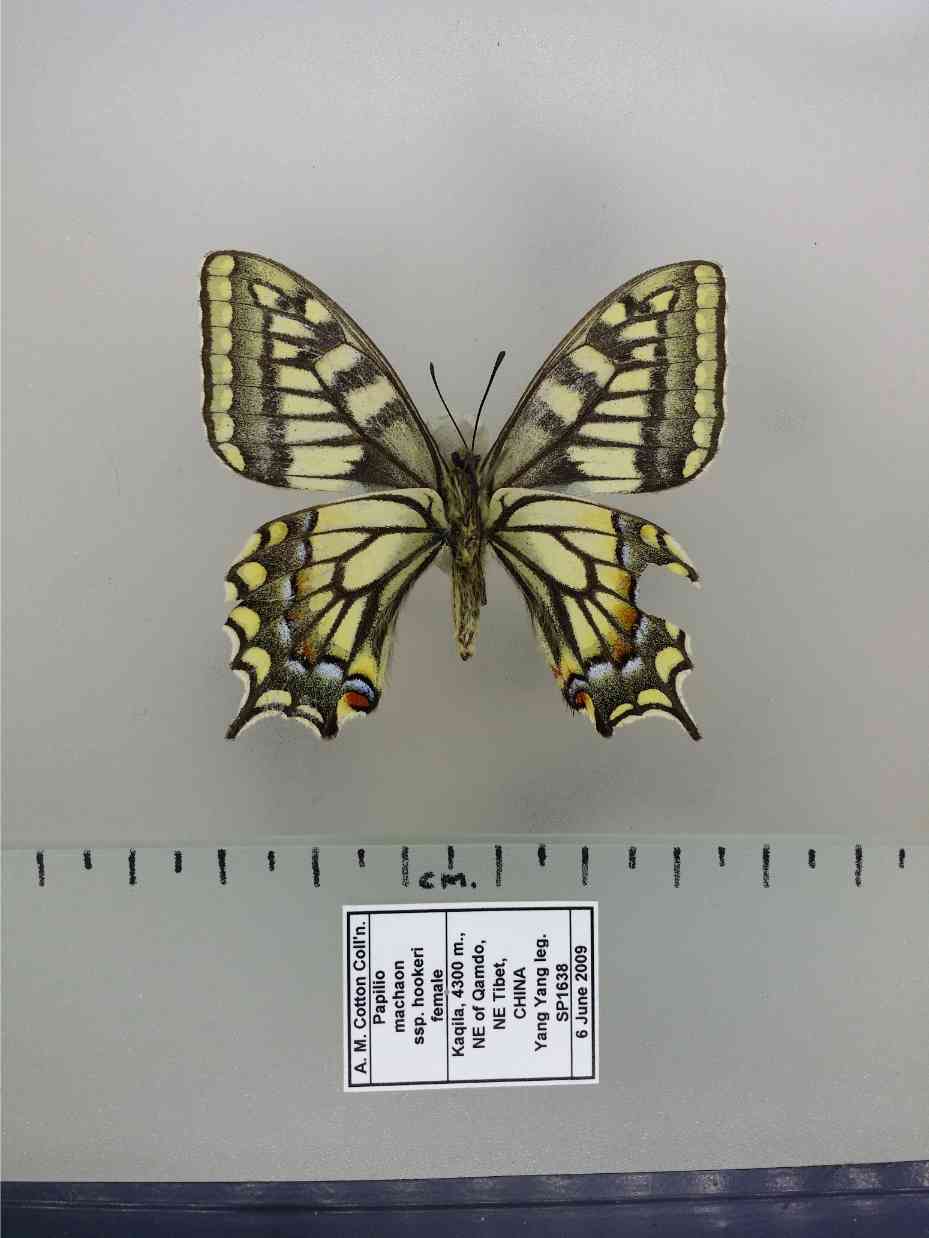

Supplement: S3 Fig — (ZIP) [file pone.0343793.s003.zip › S3/AC-SP1638V copy.jpg]

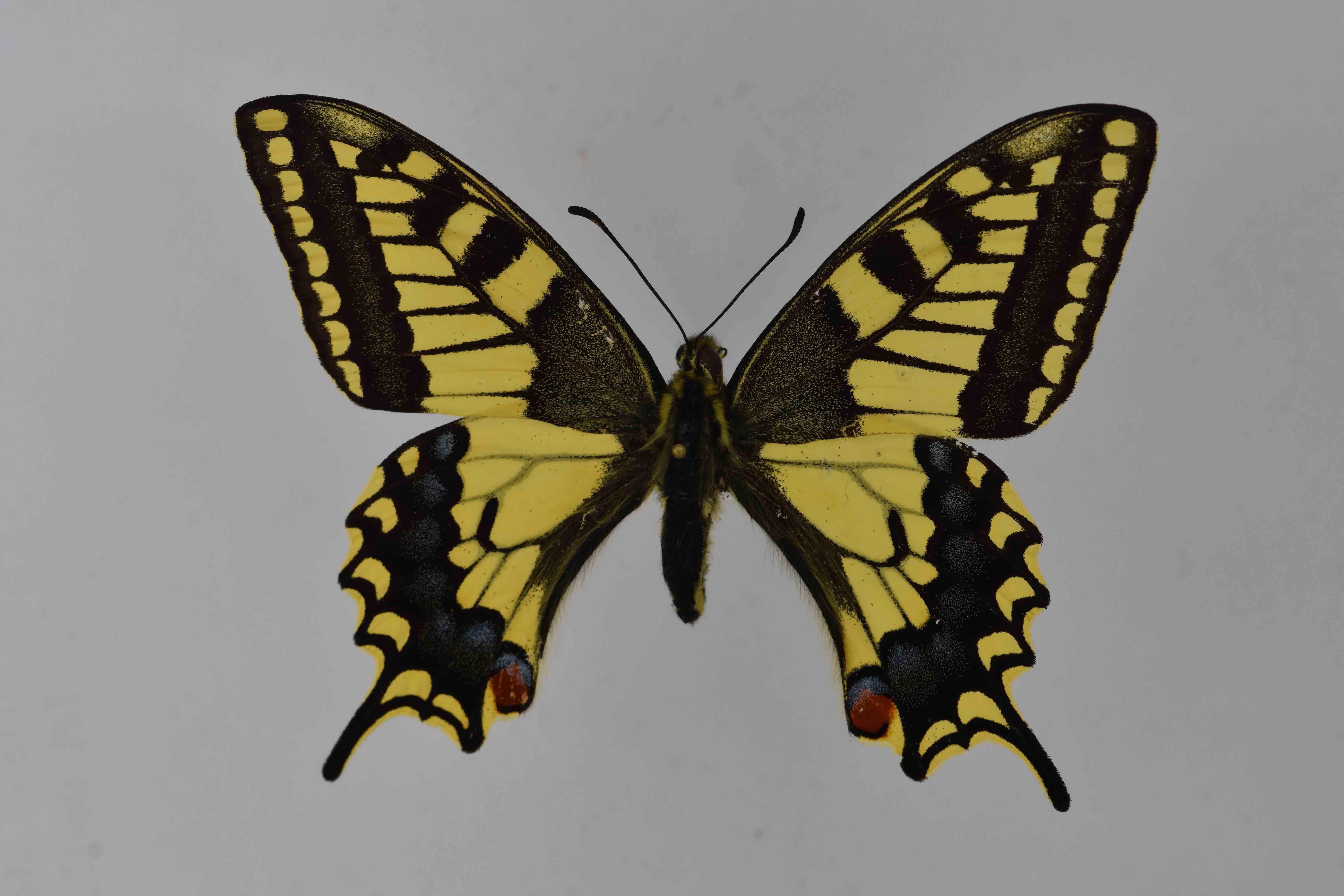

Supplement: S3 Fig — (ZIP) [file pone.0343793.s003.zip › S3/DNAwth019-D copy.jpeg]

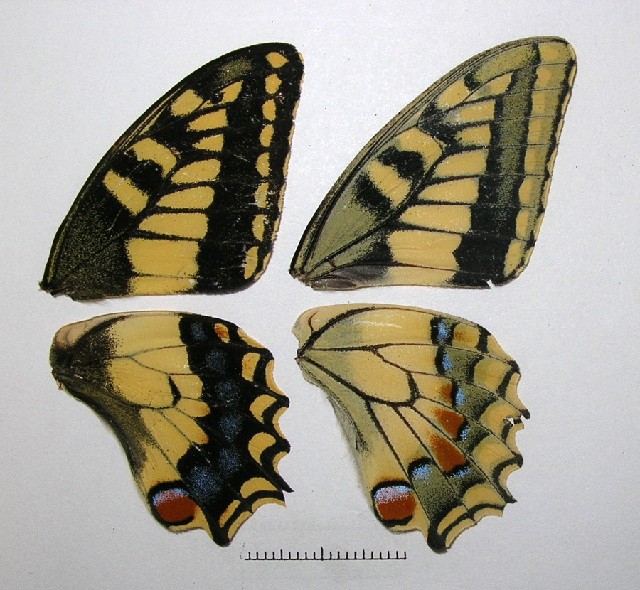

Supplement: S3 Fig — (ZIP) [file pone.0343793.s003.zip › S3/RVcoll.11-H554 .jpeg]

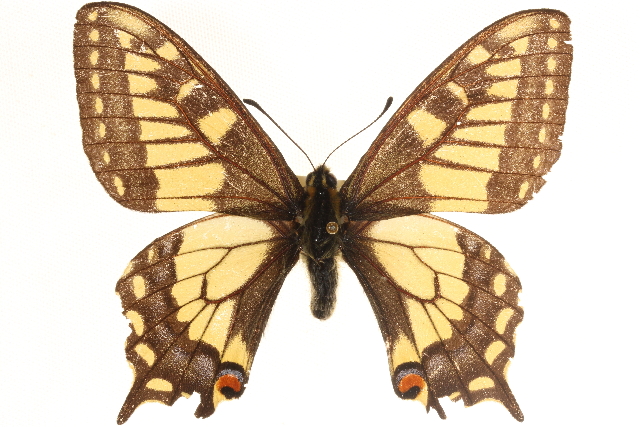

Supplement: S3 Fig — (ZIP) [file pone.0343793.s003.zip › S3/CCDB-24271-G02.jpeg]

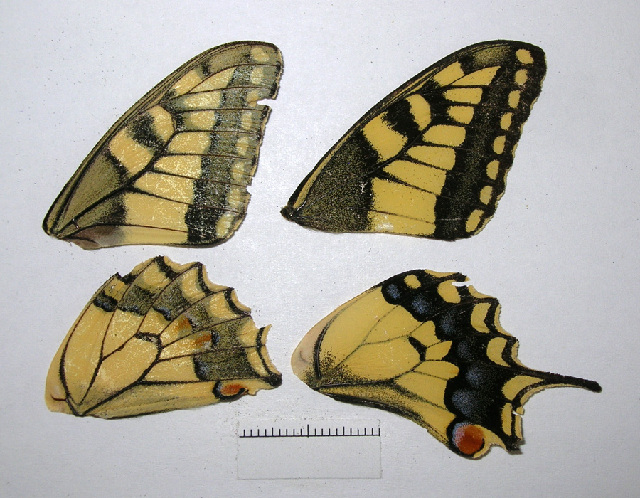

Supplement: S3 Fig — (ZIP) [file pone.0343793.s003.zip › S3/RVcoll.09-V419.jpeg]

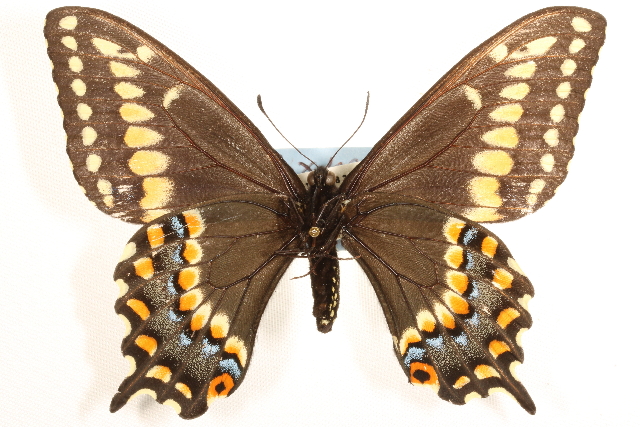

Supplement: S3 Fig — (ZIP) [file pone.0343793.s003.zip › S3/CCDB-24274-C07-V.jpeg]

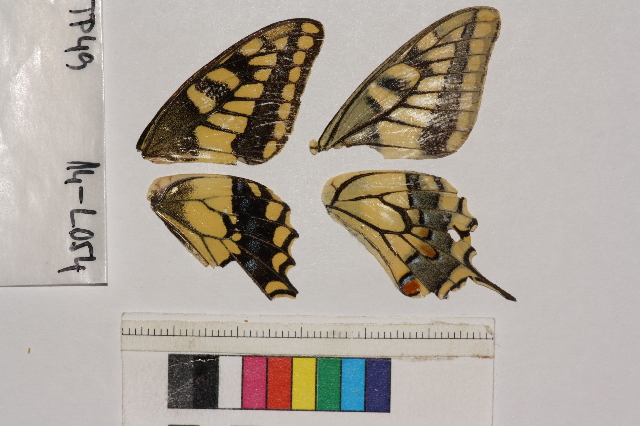

Supplement: S3 Fig — (ZIP) [file pone.0343793.s003.zip › S3/RVcoll.14-L054 .jpeg]

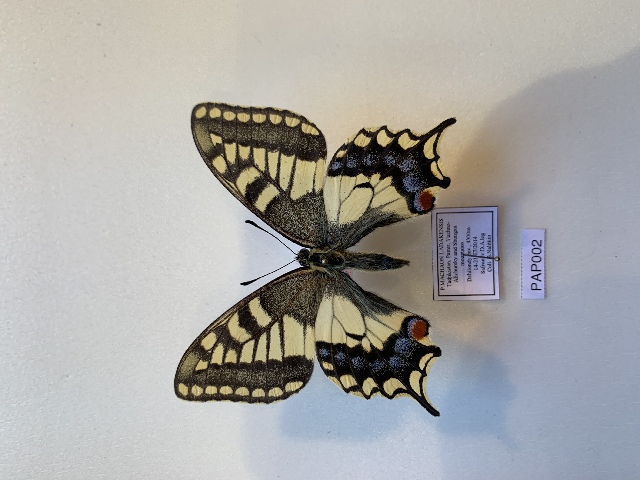

Supplement: S3 Fig — (ZIP) [file pone.0343793.s003.zip › S3/PAP002.jpeg]

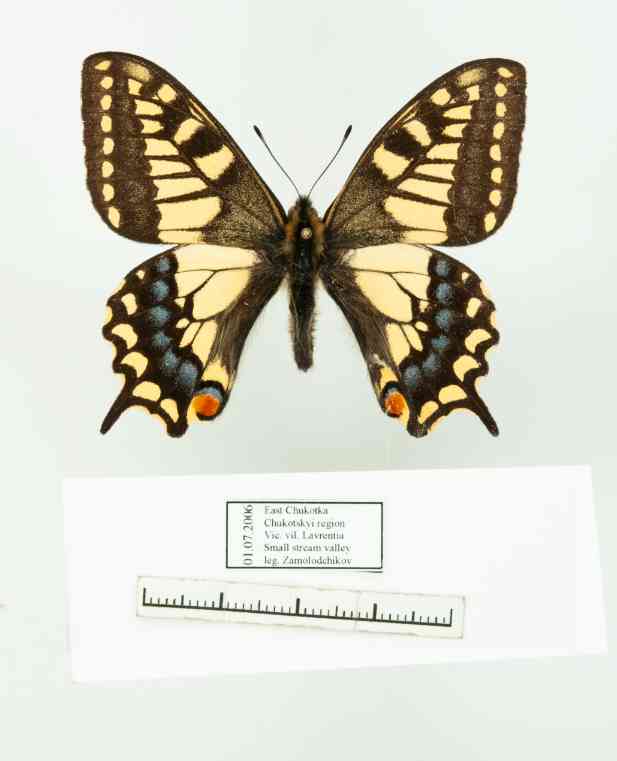

Supplement: S3 Fig — (ZIP) [file pone.0343793.s003.zip › S3/GCB08-D copy.jpg]

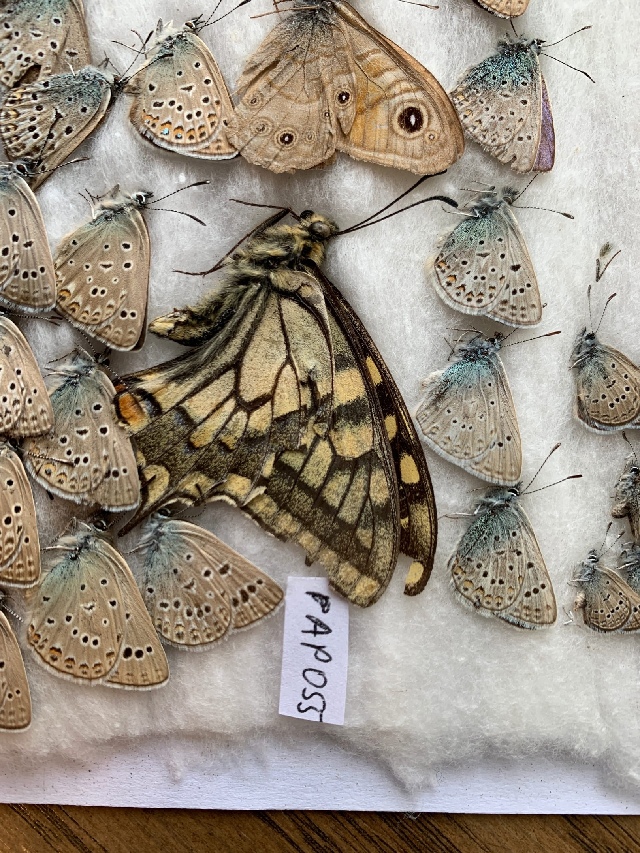

Supplement: S3 Fig — (ZIP) [file pone.0343793.s003.zip › S3/PAP055.jpeg]

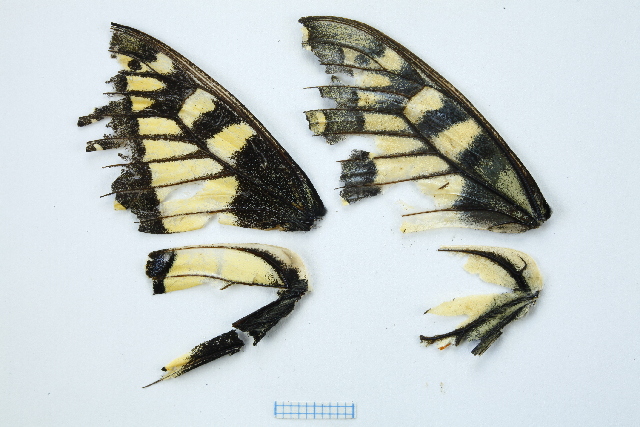

Supplement: S3 Fig — (ZIP) [file pone.0343793.s003.zip › S3/RVcoll.14-K030 .jpeg]

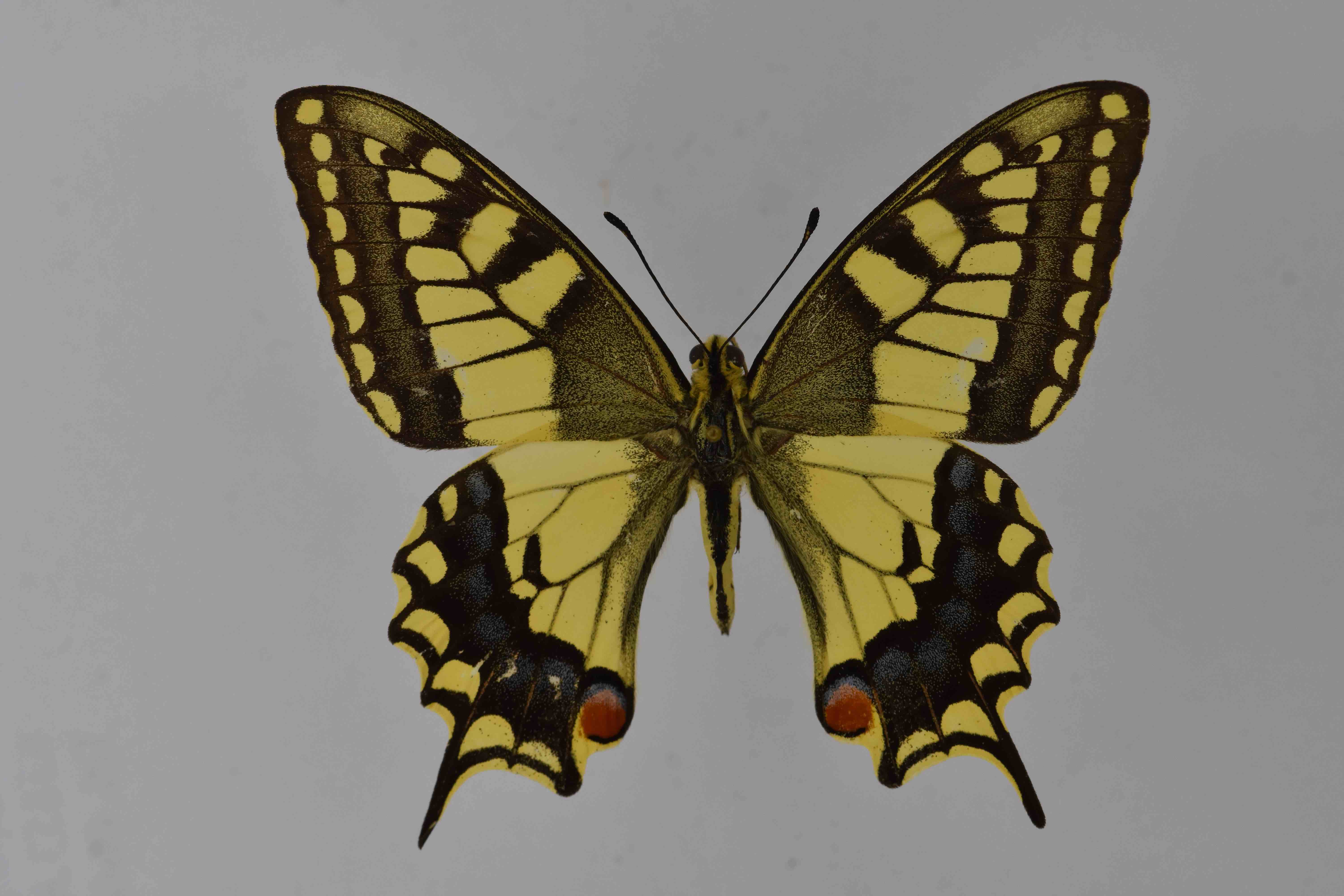

Supplement: S3 Fig — (ZIP) [file pone.0343793.s003.zip › S3/DNAwth022-D copy.jpeg]

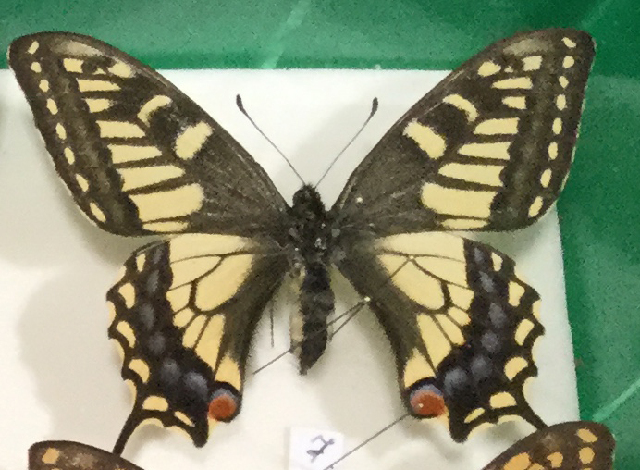

Supplement: S3 Fig — (ZIP) [file pone.0343793.s003.zip › S3/NS_107.jpeg]

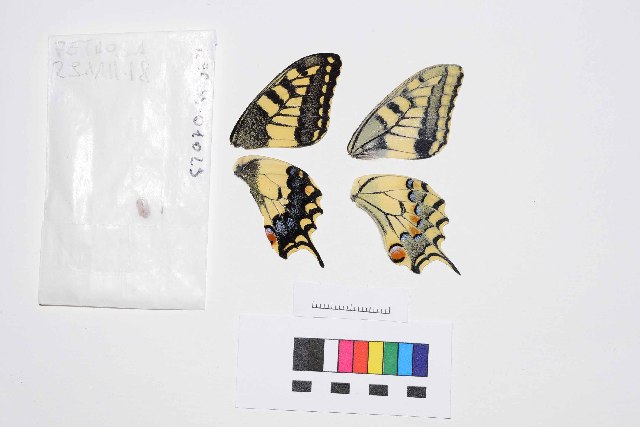

Supplement: S3 Fig — (ZIP) [file pone.0343793.s003.zip › S3/LEP-SS-01029.jpg]

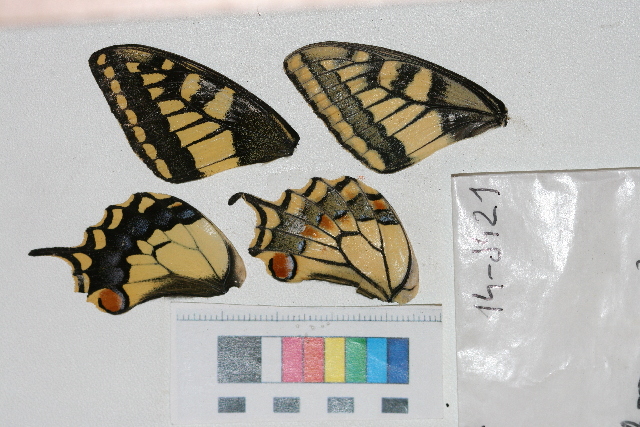

Supplement: S3 Fig — (ZIP) [file pone.0343793.s003.zip › S3/RVcoll.14-J421 .jpeg]

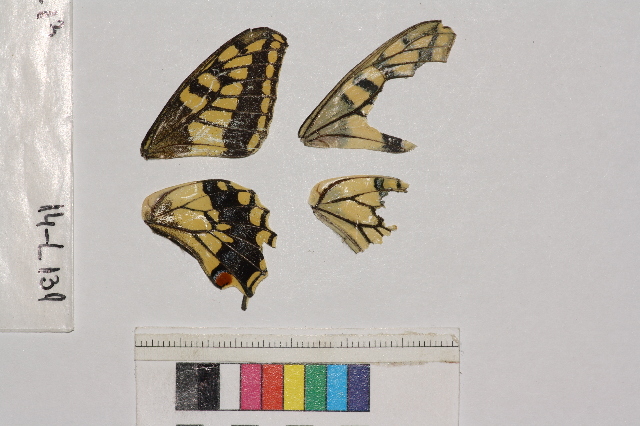

Supplement: S3 Fig — (ZIP) [file pone.0343793.s003.zip › S3/RVcoll.14-L131 .jpeg]

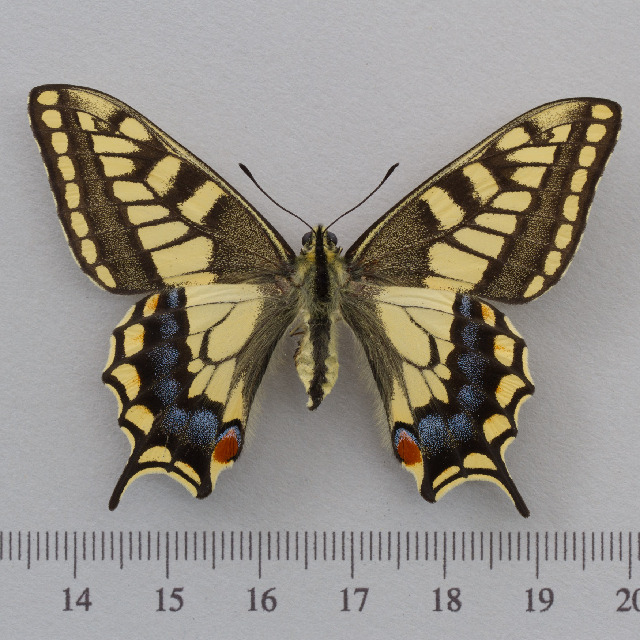

Supplement: S3 Fig — (ZIP) [file pone.0343793.s003.zip › S3/RVcoll.14-O165-D.jpg]

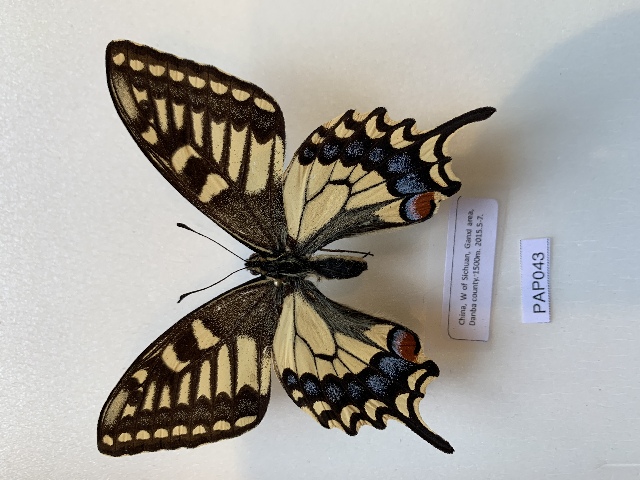

Supplement: S3 Fig — (ZIP) [file pone.0343793.s003.zip › S3/PAP043.jpeg]

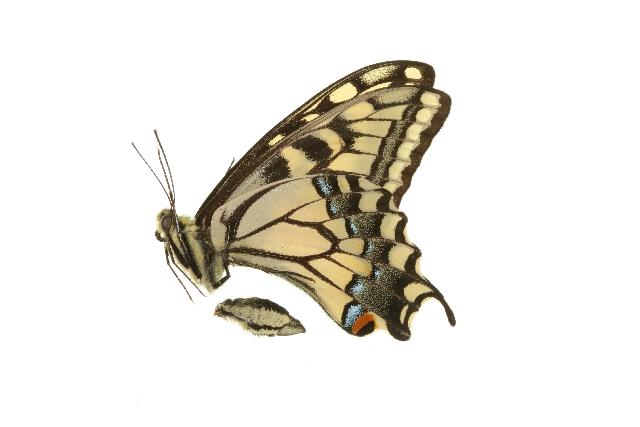

Supplement: S3 Fig — (ZIP) [file pone.0343793.s003.zip › S3/EZ0007CNC .jpeg]

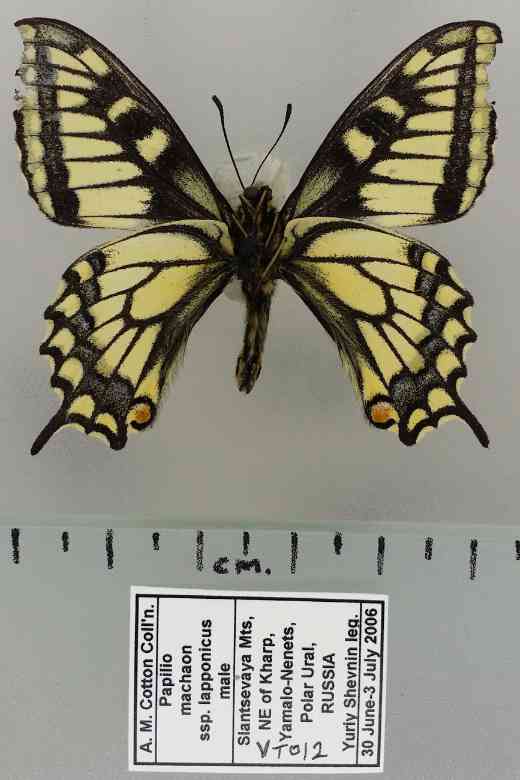

Supplement: S3 Fig — (ZIP) [file pone.0343793.s003.zip › S3/AC-VT012-V copy.jpg]

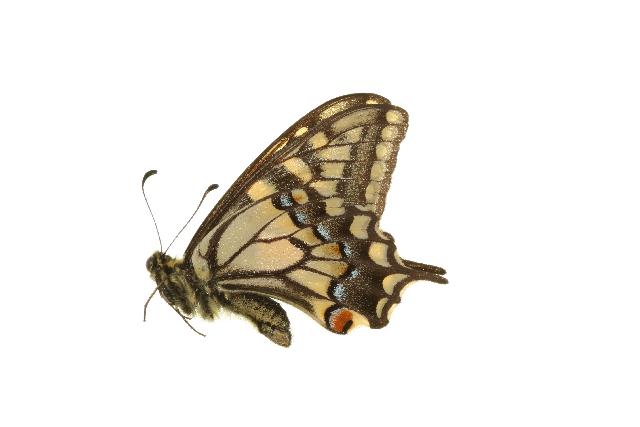

Supplement: S3 Fig — (ZIP) [file pone.0343793.s003.zip › S3/EZ0006CNC .jpeg]

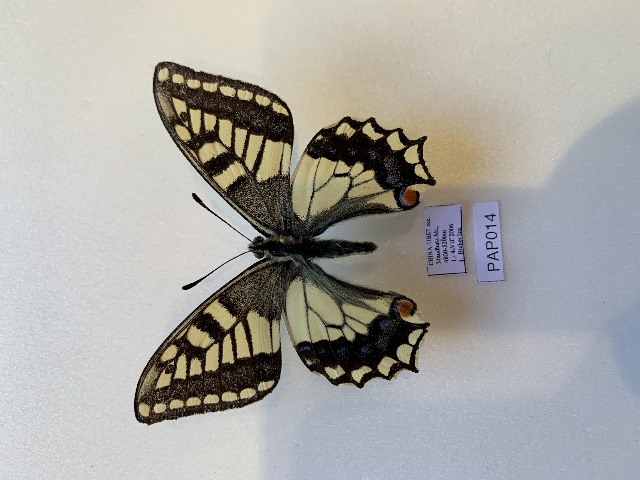

Supplement: S3 Fig — (ZIP) [file pone.0343793.s003.zip › S3/PAP014.jpeg]

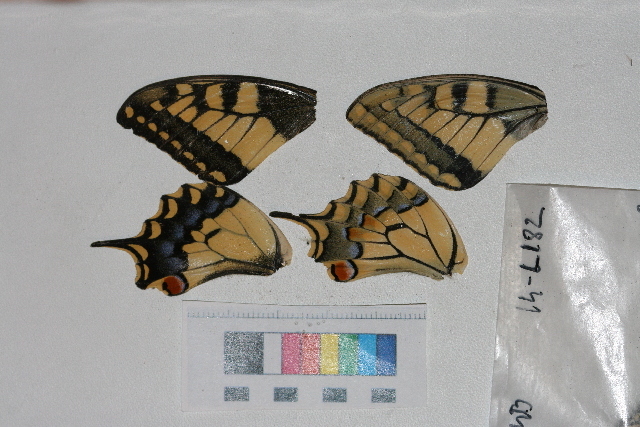

Supplement: S3 Fig — (ZIP) [file pone.0343793.s003.zip › S3/RVcoll. 14-L182 .jpeg]

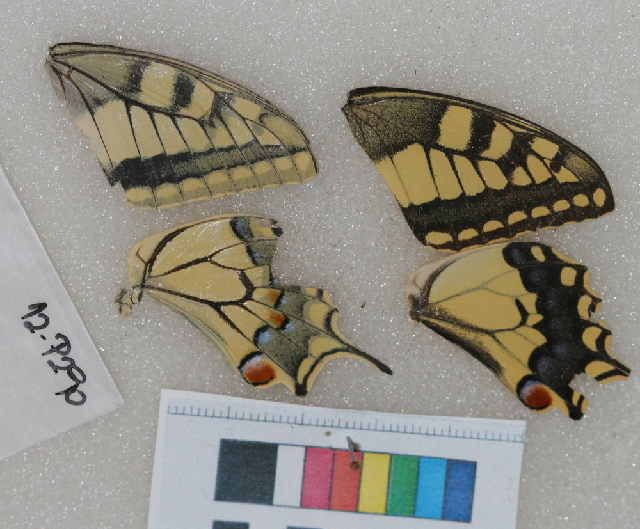

Supplement: S3 Fig — (ZIP) [file pone.0343793.s003.zip › S3/RVcoll.12-P290 .jpeg]

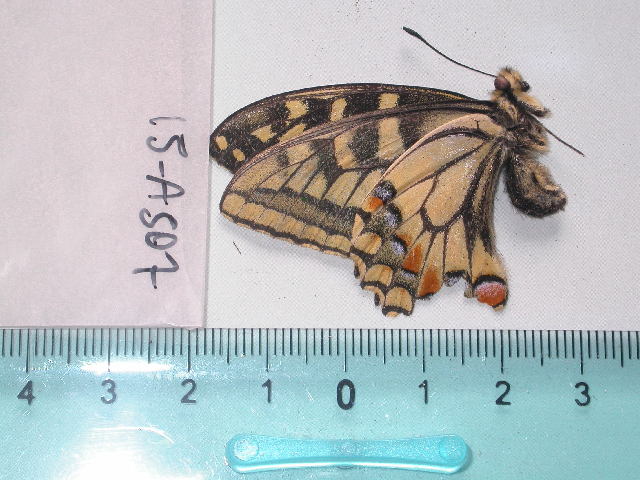

Supplement: S3 Fig — (ZIP) [file pone.0343793.s003.zip › S3/15-A507.jpeg]

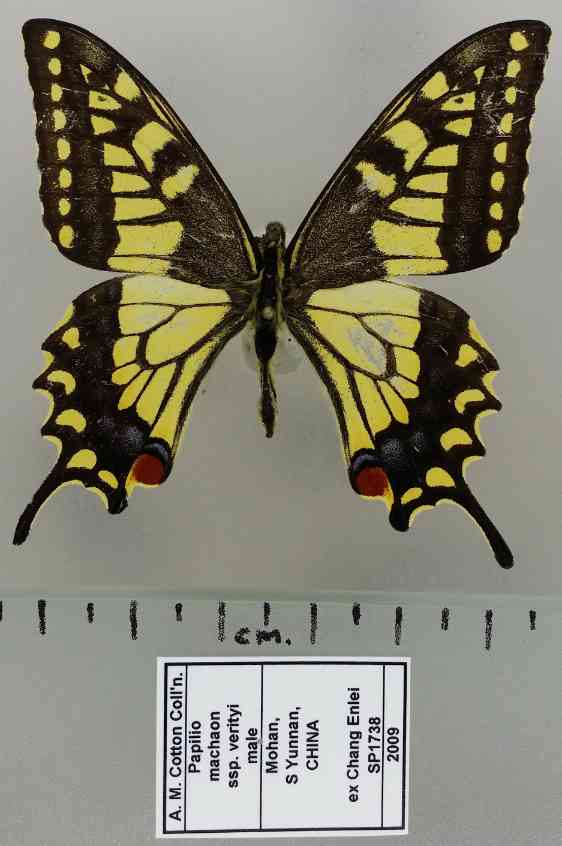

Supplement: S3 Fig — (ZIP) [file pone.0343793.s003.zip › S3/AC-SP1738-D copy.jpg]

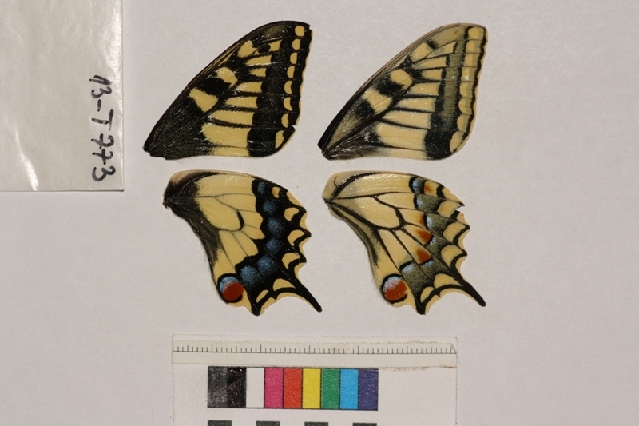

Supplement: S3 Fig — (ZIP) [file pone.0343793.s003.zip › S3/RVcoll.13-T773 .jpg]

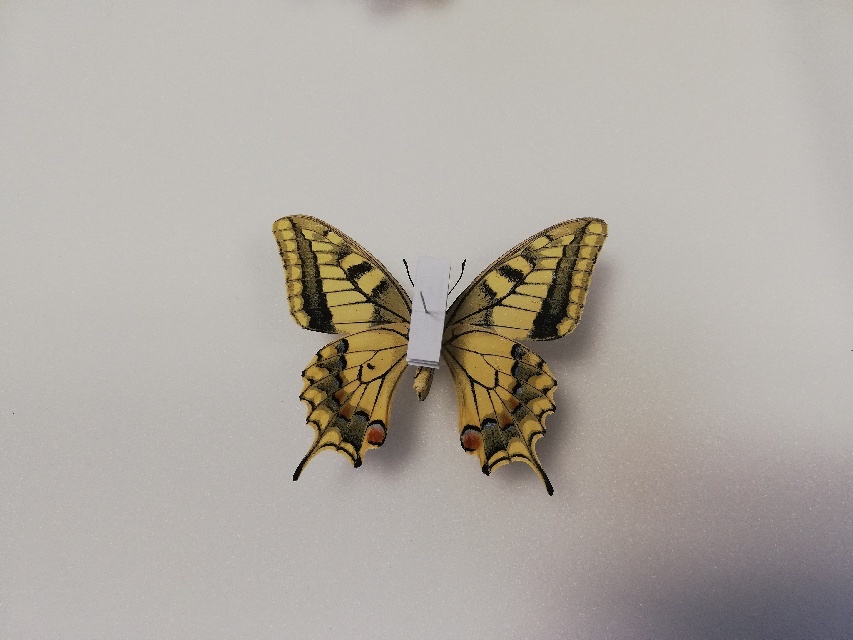

Supplement: S3 Fig — (ZIP) [file pone.0343793.s003.zip › S3/OCIC-PM2-V.jpeg]

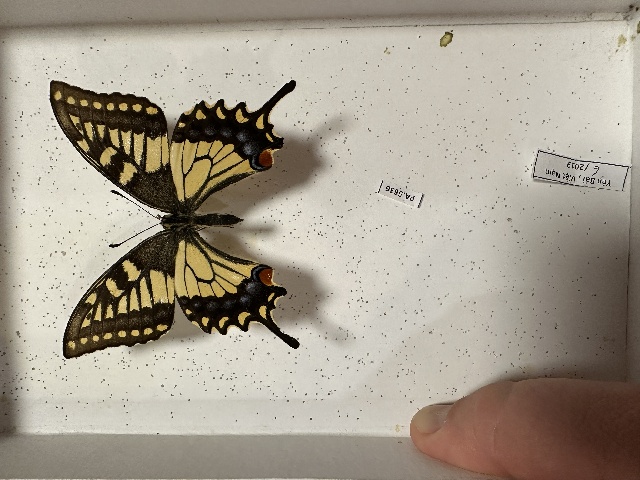

Supplement: S3 Fig — (ZIP) [file pone.0343793.s003.zip › S3/PAP080.jpeg]

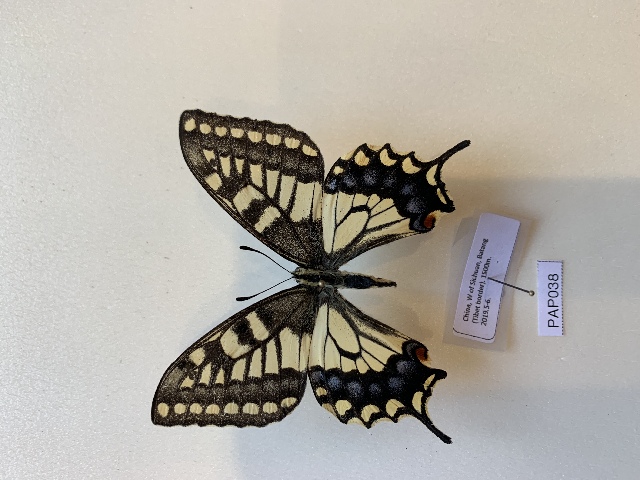

Supplement: S3 Fig — (ZIP) [file pone.0343793.s003.zip › S3/PAP038.jpeg]

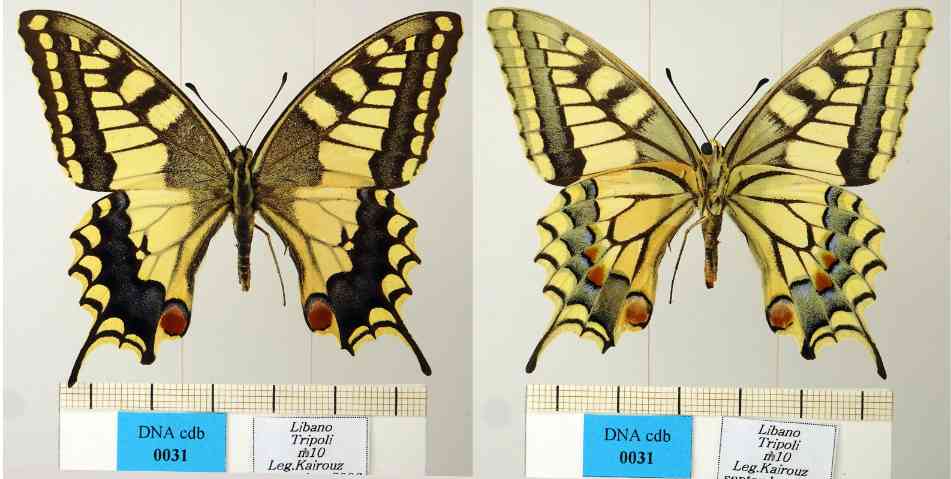

Supplement: S3 Fig — (ZIP) [file pone.0343793.s003.zip › S3/DNAcdb0031 copy.jpg]

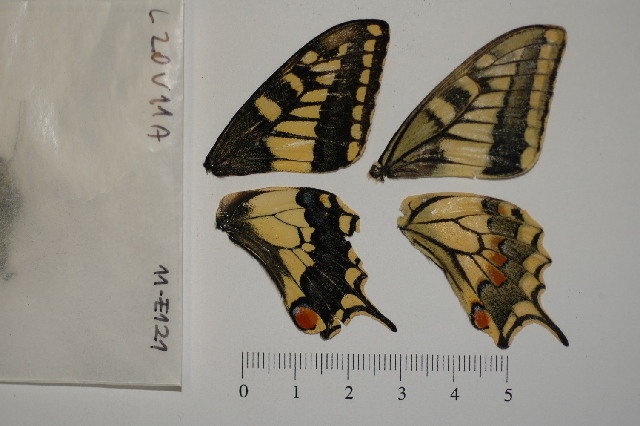

Supplement: S3 Fig — (ZIP) [file pone.0343793.s003.zip › S3/RVcoll.11-E121 .jpeg]

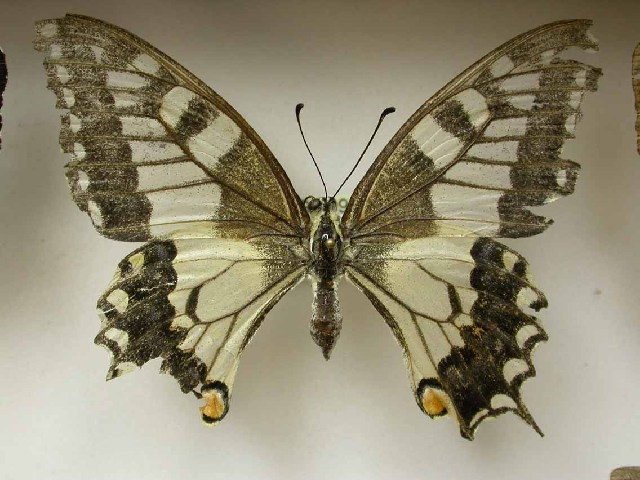

Supplement: S3 Fig — (ZIP) [file pone.0343793.s003.zip › S3/LEP-SS-00929.jpg]

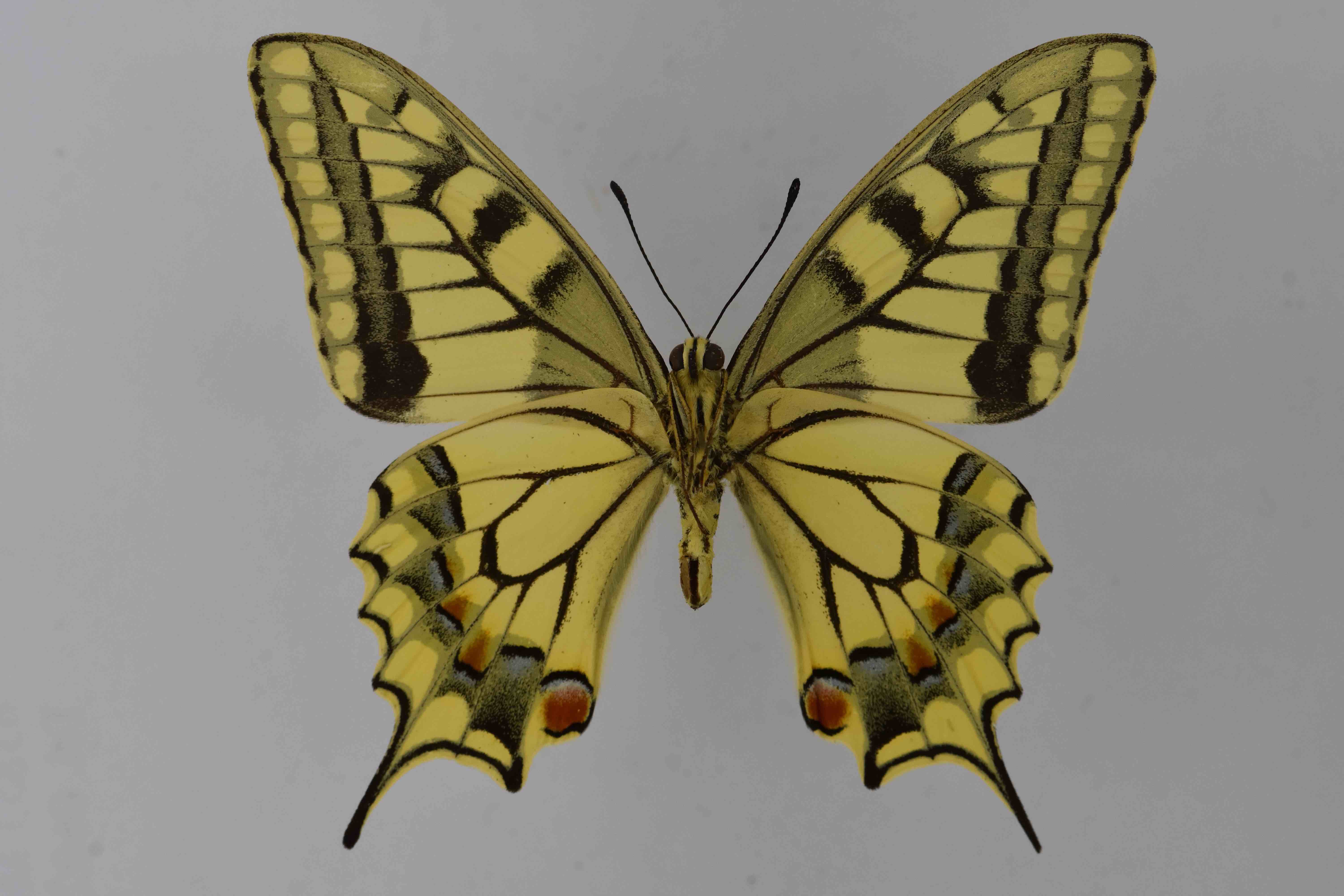

Supplement: S3 Fig — (ZIP) [file pone.0343793.s003.zip › S3/DNAwth028-V copy.jpeg]

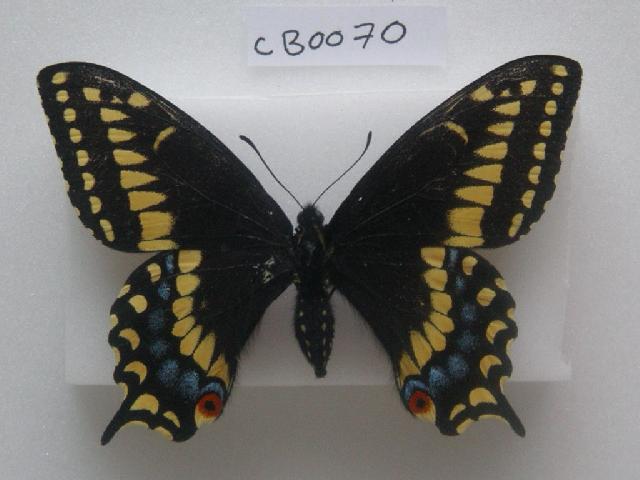

Supplement: S3 Fig — (ZIP) [file pone.0343793.s003.zip › S3/CB0070-D.jpeg]

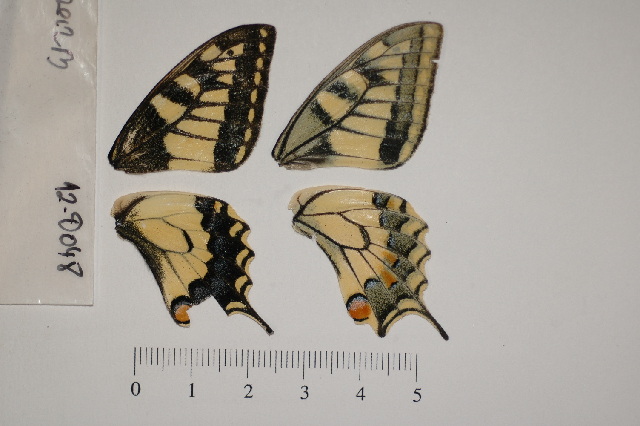

Supplement: S3 Fig — (ZIP) [file pone.0343793.s003.zip › S3/RVcoll.12-O048 .jpeg]

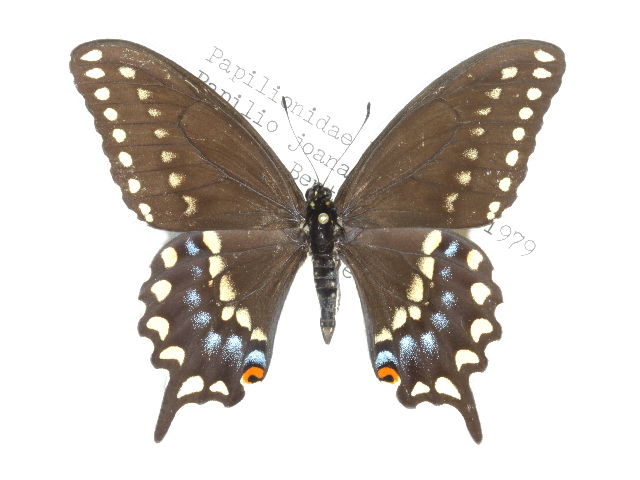

Supplement: S3 Fig — (ZIP) [file pone.0343793.s003.zip › S3/CCDB-30818-B11.jpeg]

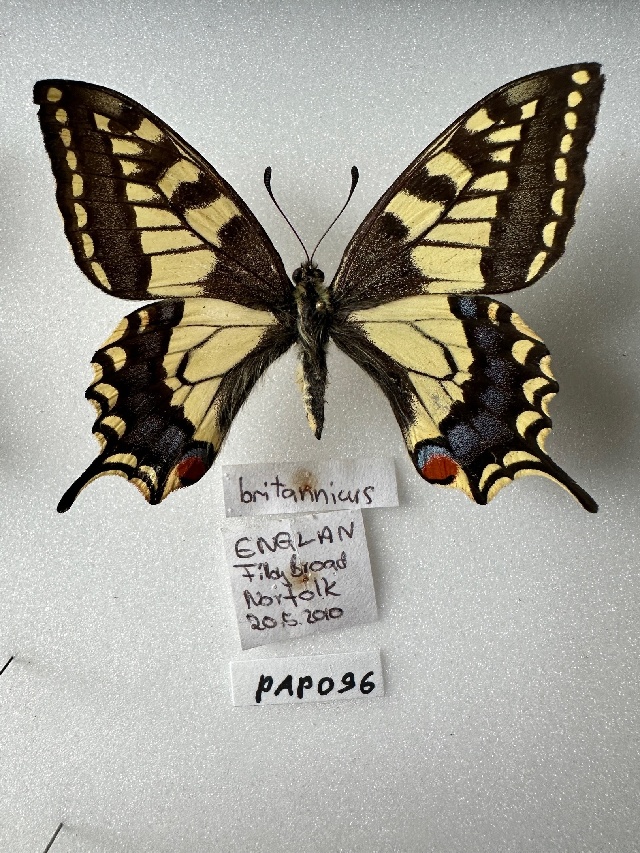

Supplement: S3 Fig — (ZIP) [file pone.0343793.s003.zip › S3/PAP096.jpeg]

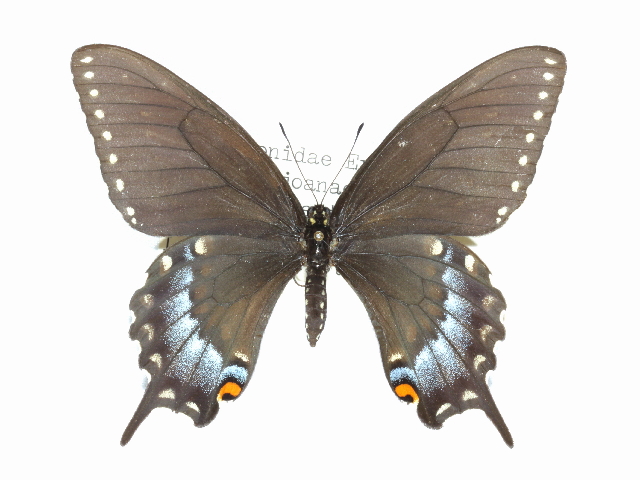

Supplement: S3 Fig — (ZIP) [file pone.0343793.s003.zip › S3/CCDB-30818-B10.jpeg]

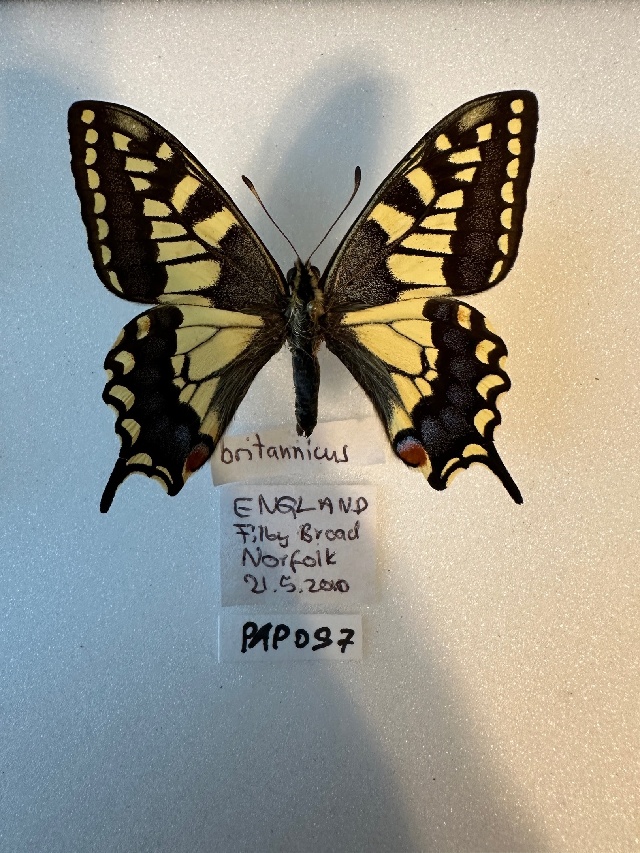

Supplement: S3 Fig — (ZIP) [file pone.0343793.s003.zip › S3/PAP097.jpeg]

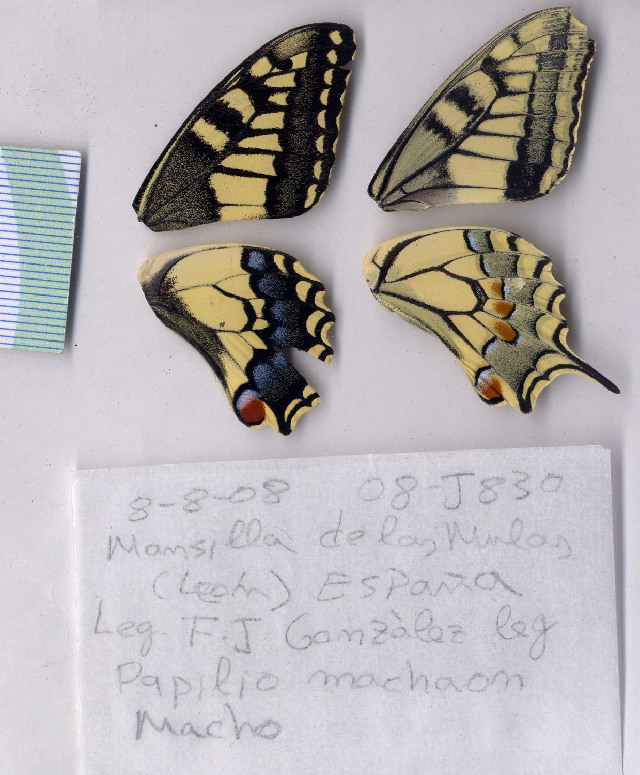

Supplement: S3 Fig — (ZIP) [file pone.0343793.s003.zip › S3/RVcoll.08-J830.jpg]

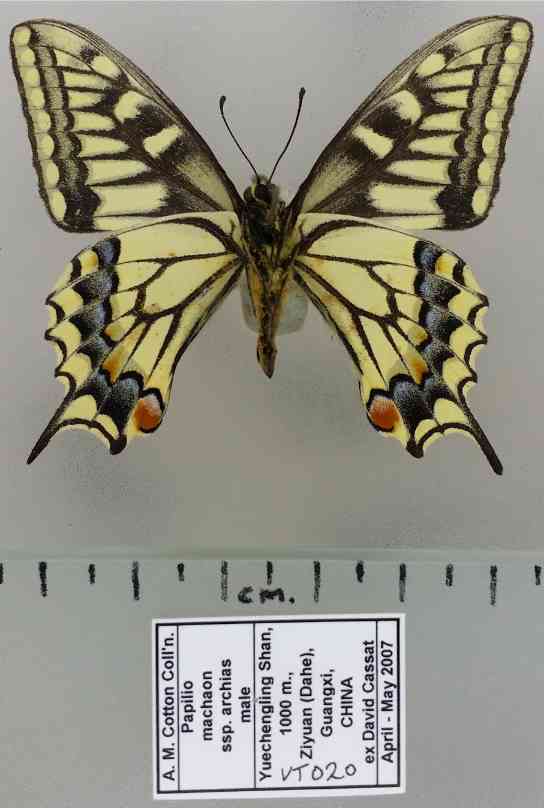

Supplement: S3 Fig — (ZIP) [file pone.0343793.s003.zip › S3/AC-VT020-V copy.jpg]

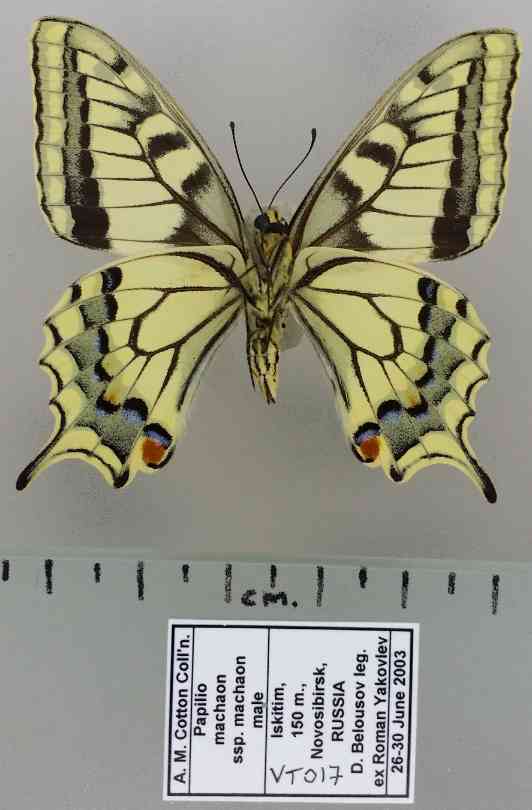

Supplement: S3 Fig — (ZIP) [file pone.0343793.s003.zip › S3/AC-VT017-V copy.jpg]

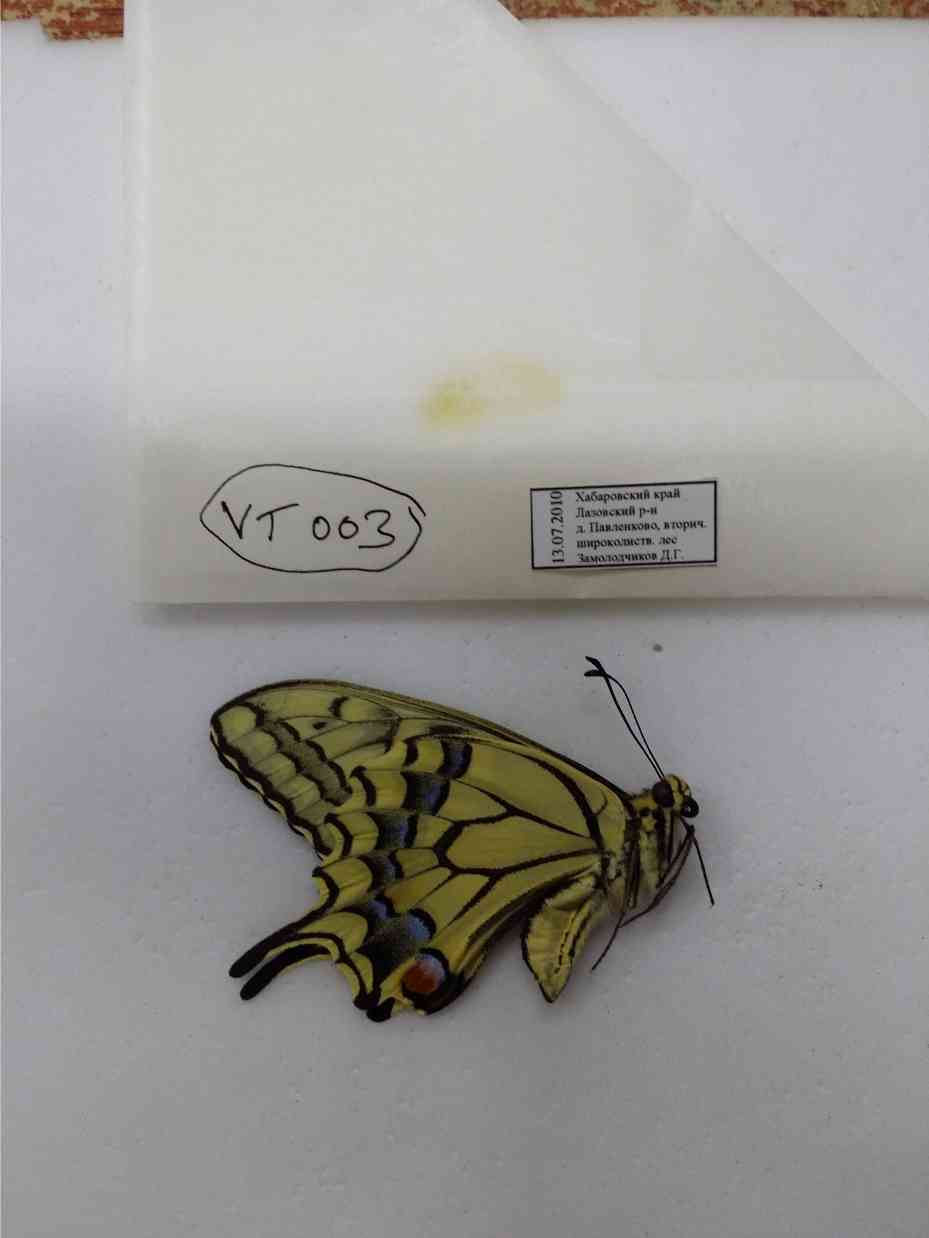

Supplement: S3 Fig — (ZIP) [file pone.0343793.s003.zip › S3/AC-VT003 copy.jpg]

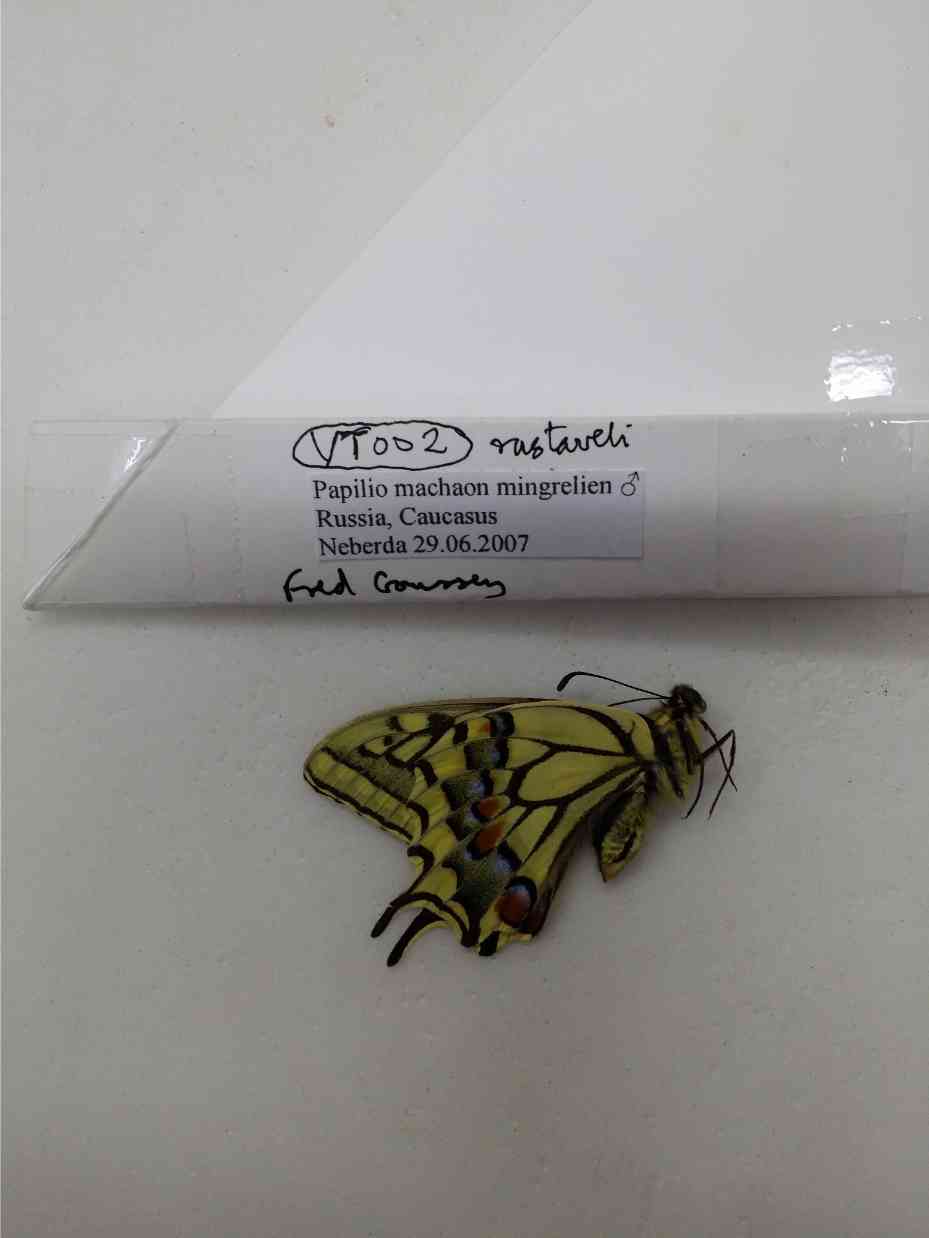

Supplement: S3 Fig — (ZIP) [file pone.0343793.s003.zip › S3/AC-VT002 copy.jpg]

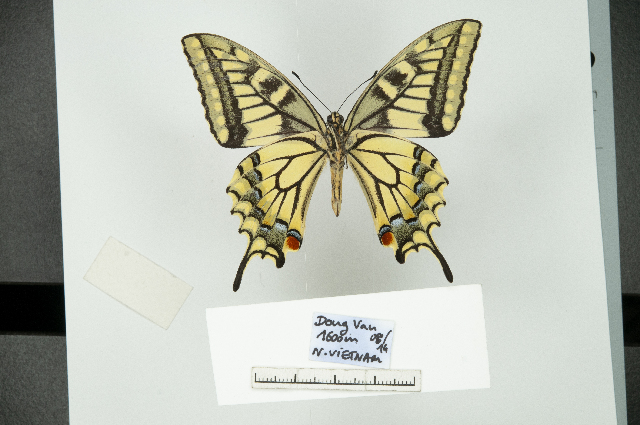

Supplement: S3 Fig — (ZIP) [file pone.0343793.s003.zip › S3/GCB01-V copy.jpeg]

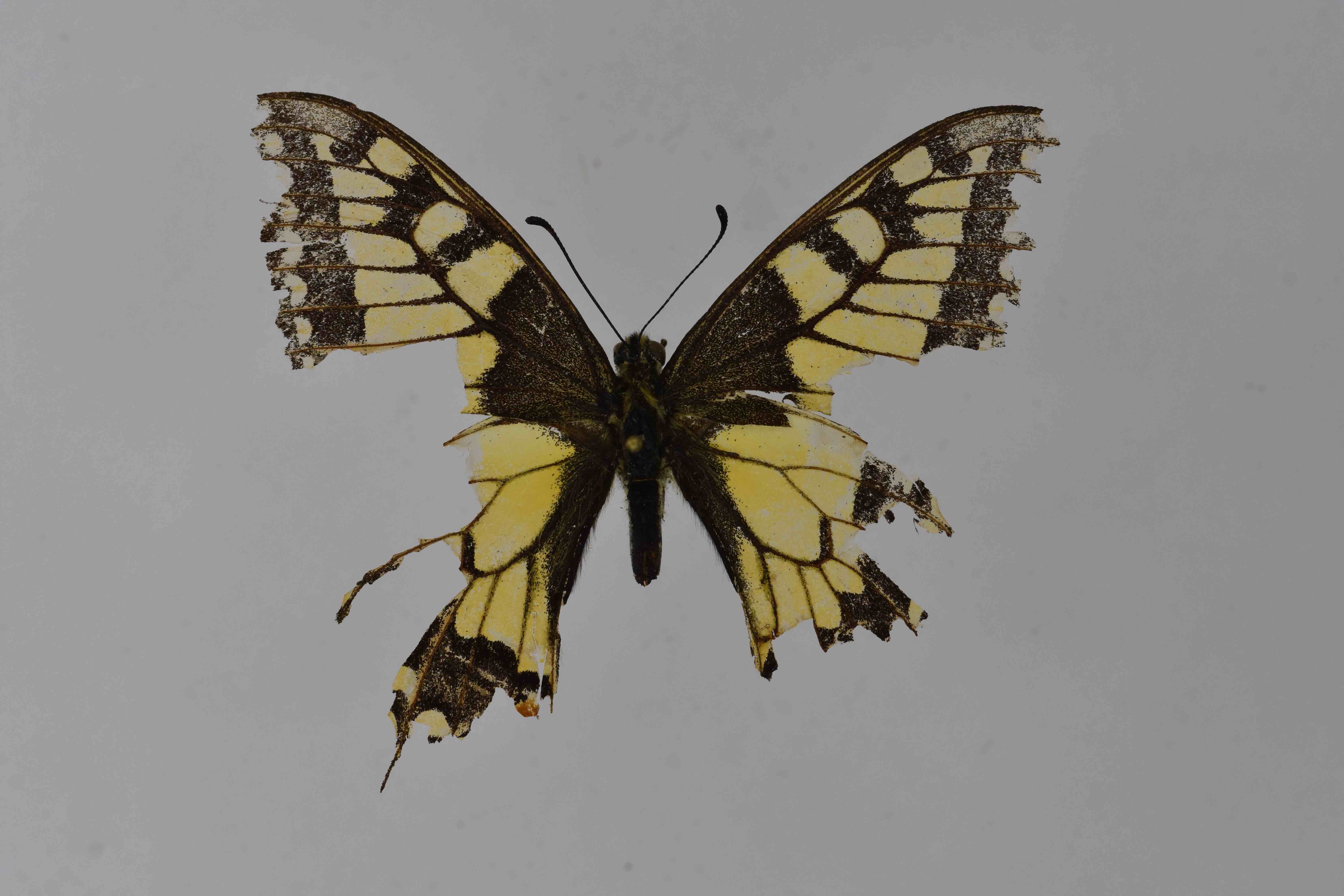

Supplement: S3 Fig — (ZIP) [file pone.0343793.s003.zip › S3/DNAwth027-D copy.jpeg]

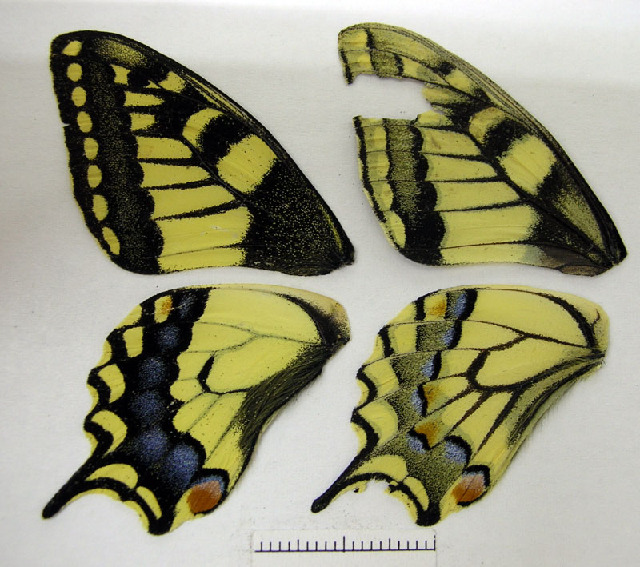

Supplement: S3 Fig — (ZIP) [file pone.0343793.s003.zip › S3/RVcoll.08-M338.jpeg]

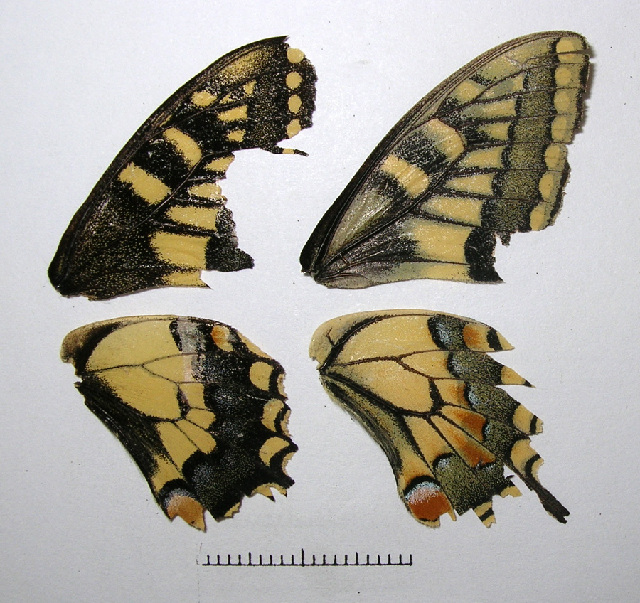

Supplement: S3 Fig — (ZIP) [file pone.0343793.s003.zip › S3/RVcoll.11-D414 .jpeg]

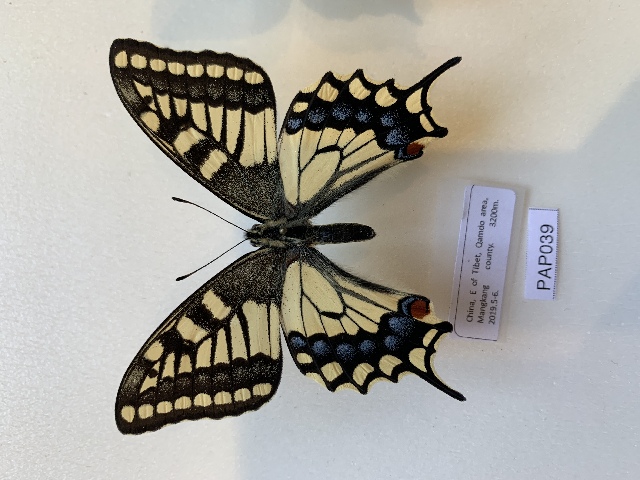

Supplement: S3 Fig — (ZIP) [file pone.0343793.s003.zip › S3/PAP039.jpeg]

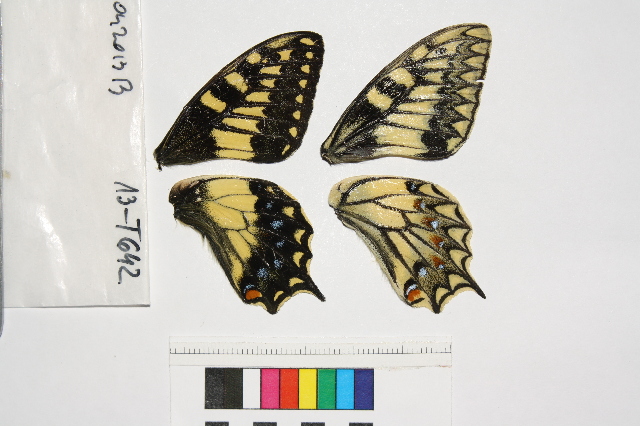

Supplement: S3 Fig — (ZIP) [file pone.0343793.s003.zip › S3/RVcoll.13-T642 .jpeg]

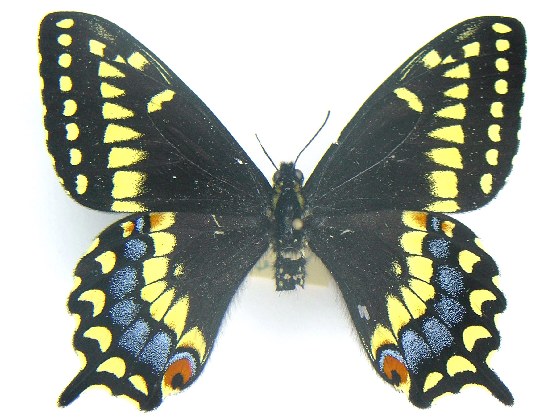

Supplement: S3 Fig — (ZIP) [file pone.0343793.s003.zip › S3/DH010967.jpg]

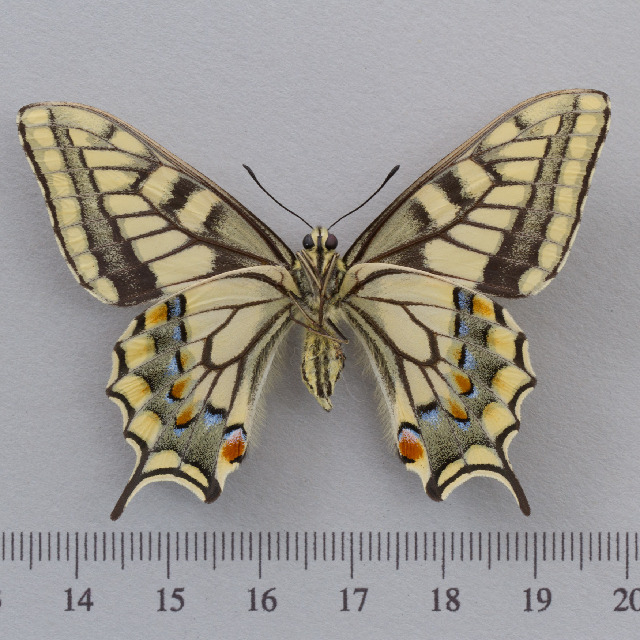

Supplement: S3 Fig — (ZIP) [file pone.0343793.s003.zip › S3/RVcoll.14-O165-V.jpg]

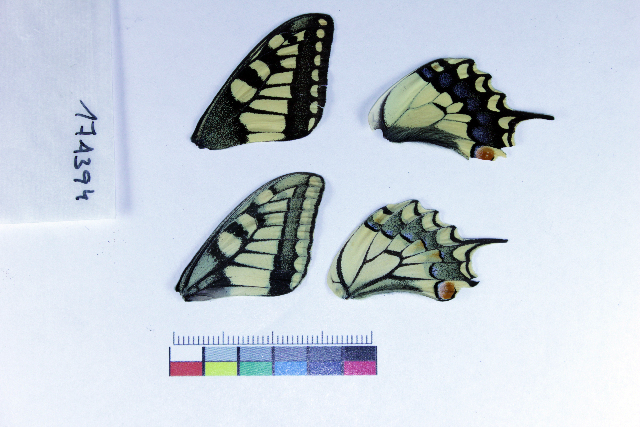

Supplement: S3 Fig — (ZIP) [file pone.0343793.s003.zip › S3/RVcoll17A394.jpeg]

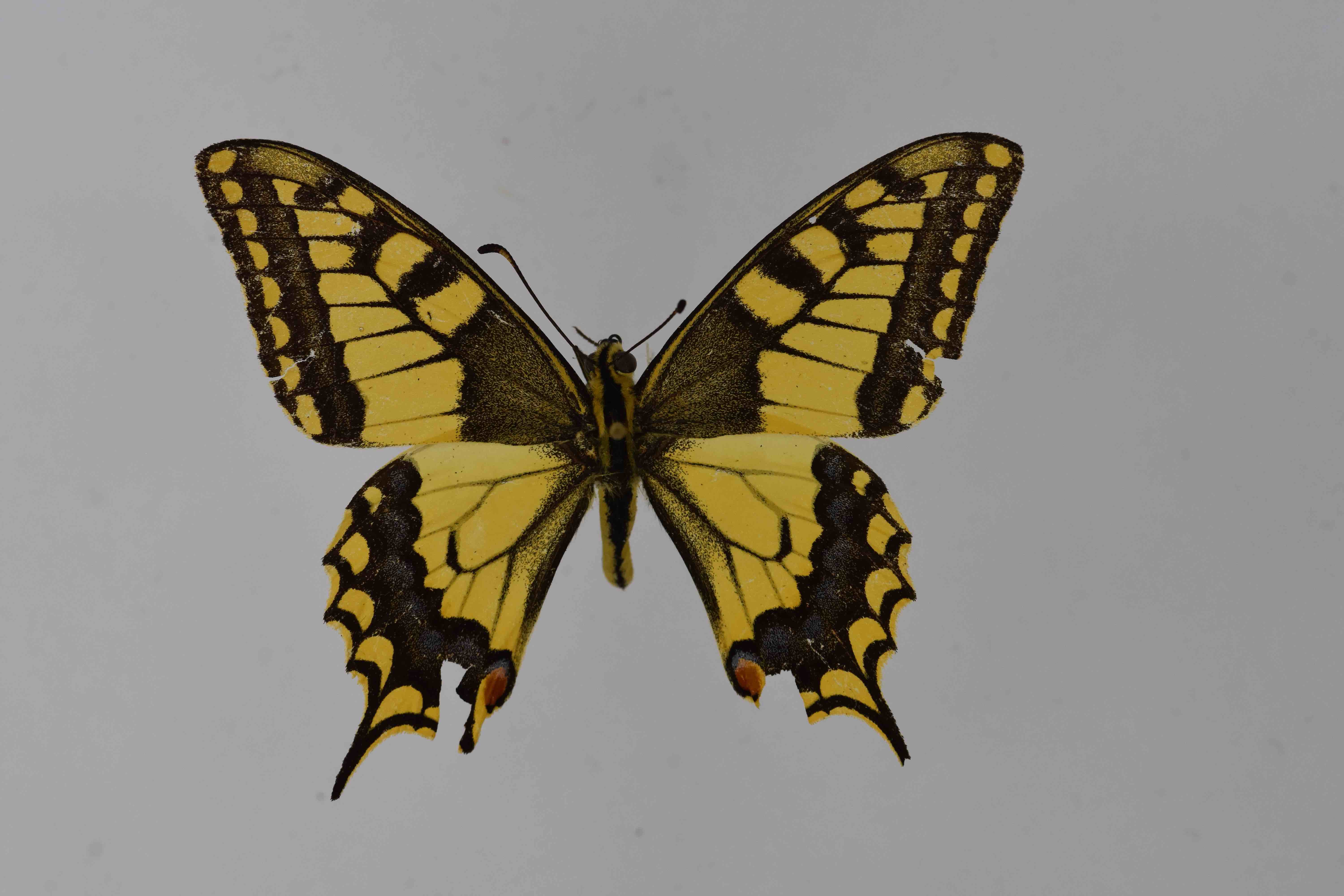

Supplement: S3 Fig — (ZIP) [file pone.0343793.s003.zip › S3/DNAwth009-D copy.jpeg]

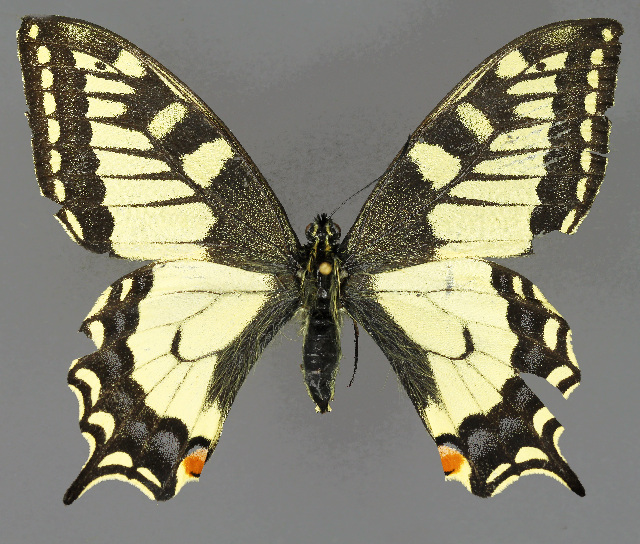

Supplement: S3 Fig — (ZIP) [file pone.0343793.s003.zip › S3/NHMO-DAR-11929.jpg]

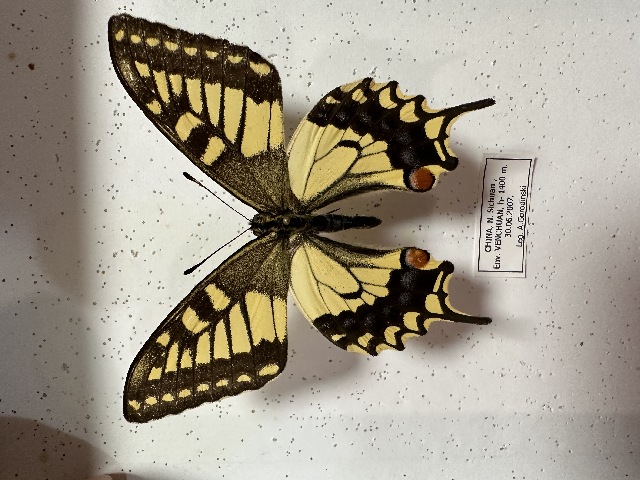

Supplement: S3 Fig — (ZIP) [file pone.0343793.s003.zip › S3/PAP081.jpeg]

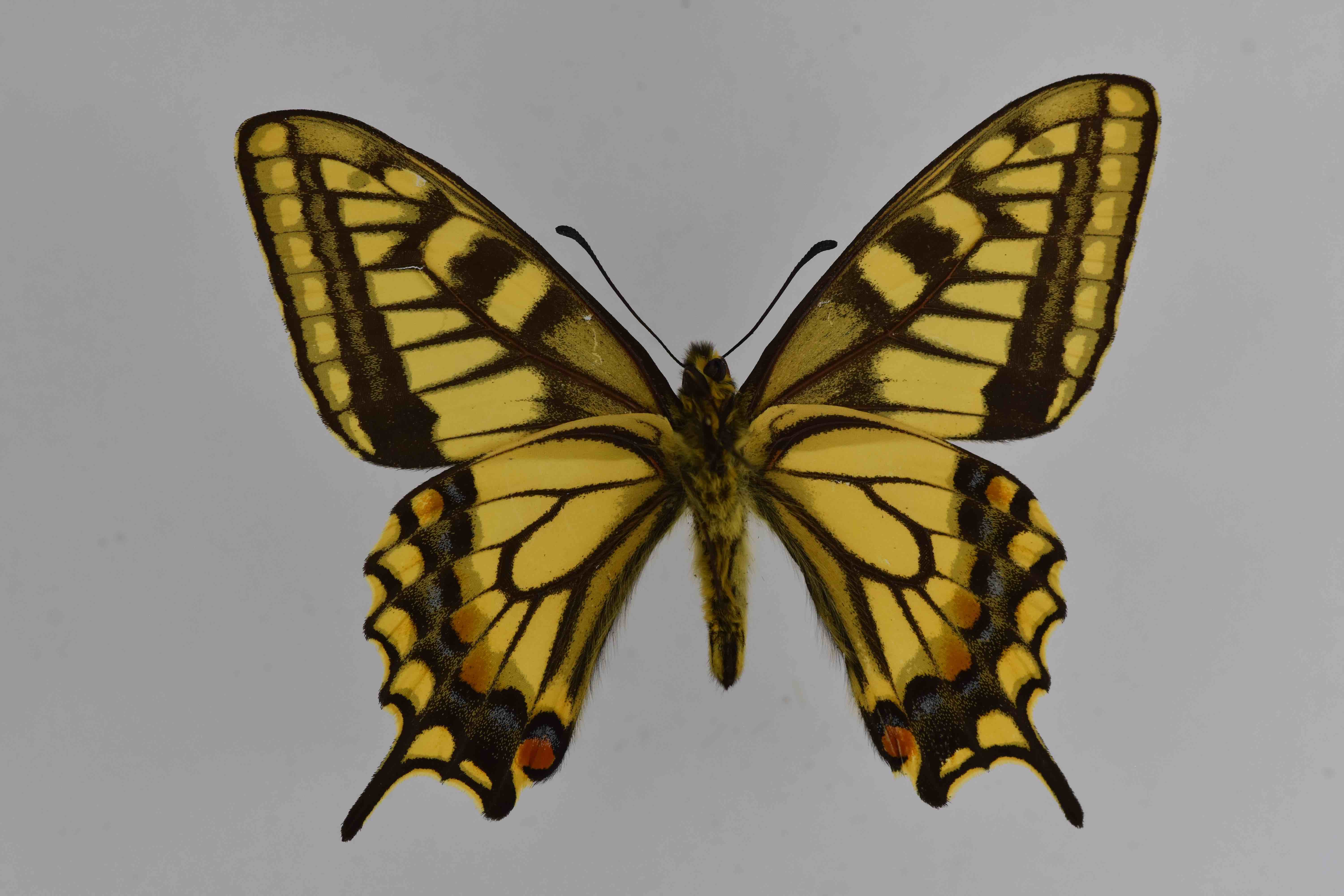

Supplement: S3 Fig — (ZIP) [file pone.0343793.s003.zip › S3/DNAwth031-V copy.jpeg]

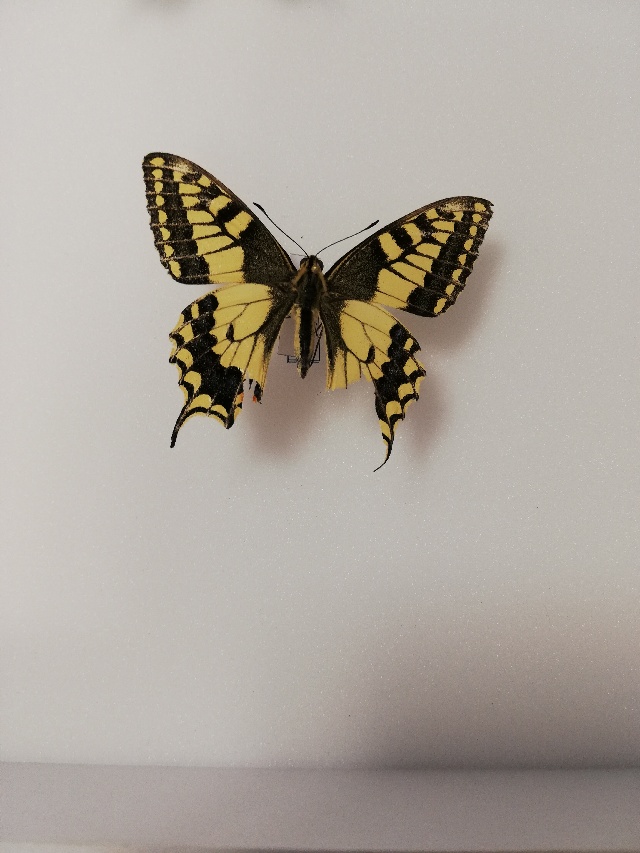

Supplement: S3 Fig — (ZIP) [file pone.0343793.s003.zip › S3/OCIC-PM8-D.jpeg]

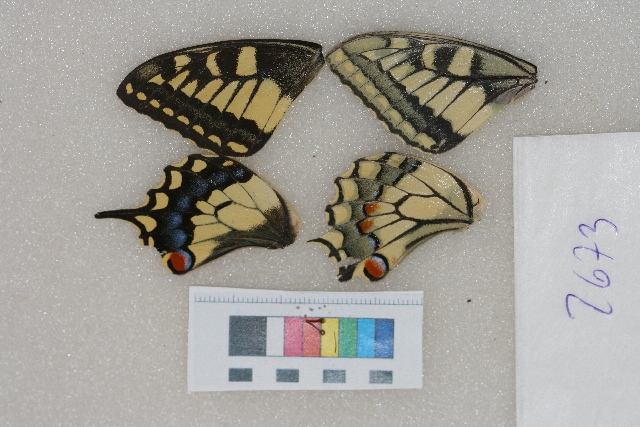

Supplement: S3 Fig — (ZIP) [file pone.0343793.s003.zip › S3/RVcoll.LD-2673 .jpeg]

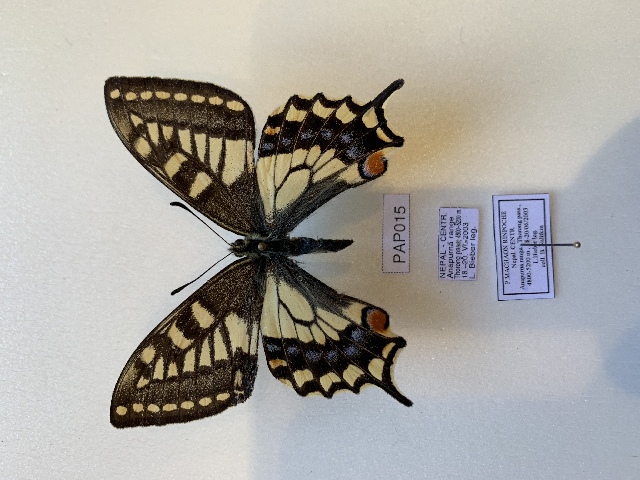

Supplement: S3 Fig — (ZIP) [file pone.0343793.s003.zip › S3/PAP015.jpeg]

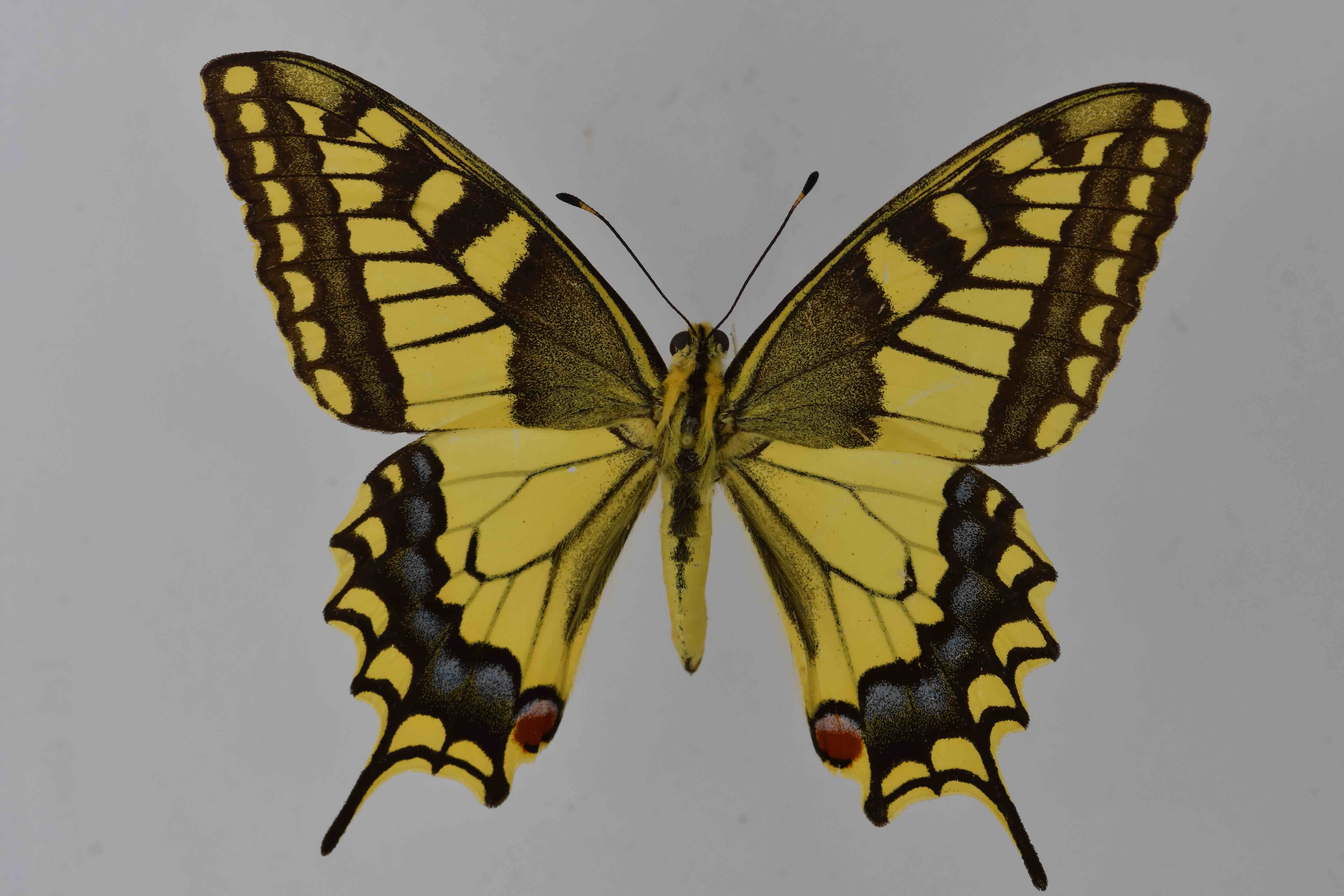

Supplement: S3 Fig — (ZIP) [file pone.0343793.s003.zip › S3/DNAwth015-D copy.jpeg]

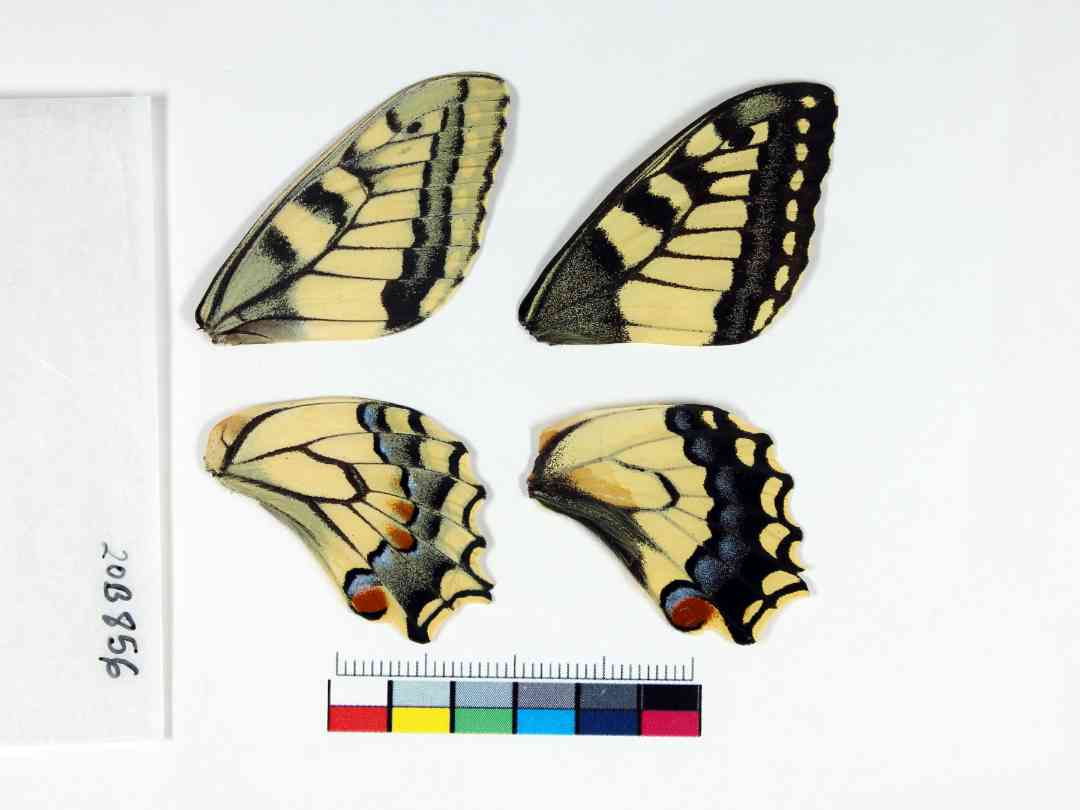

Supplement: S3 Fig — (ZIP) [file pone.0343793.s003.zip › S3/AC-PQ008 copy.jpg]

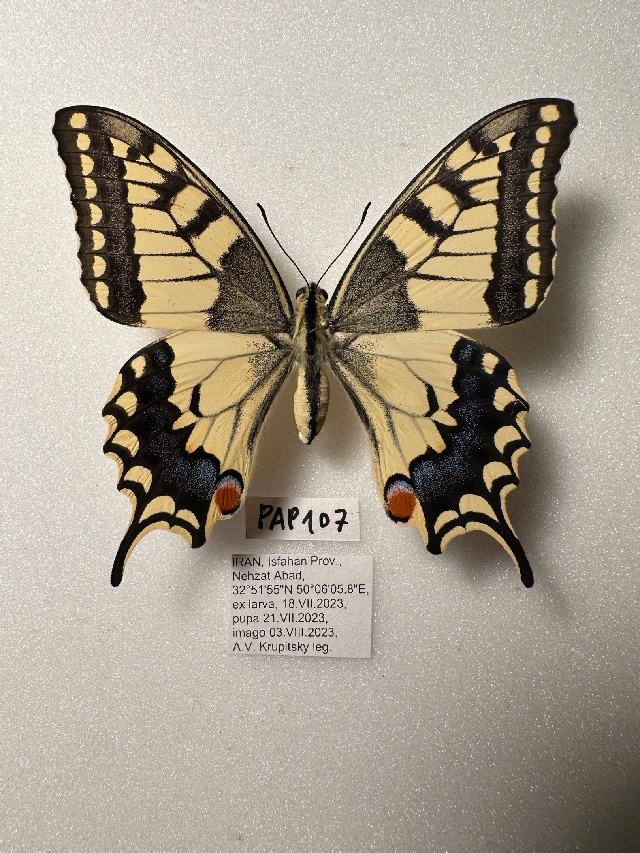

Supplement: S3 Fig — (ZIP) [file pone.0343793.s003.zip › S3/PAP107.jpeg]

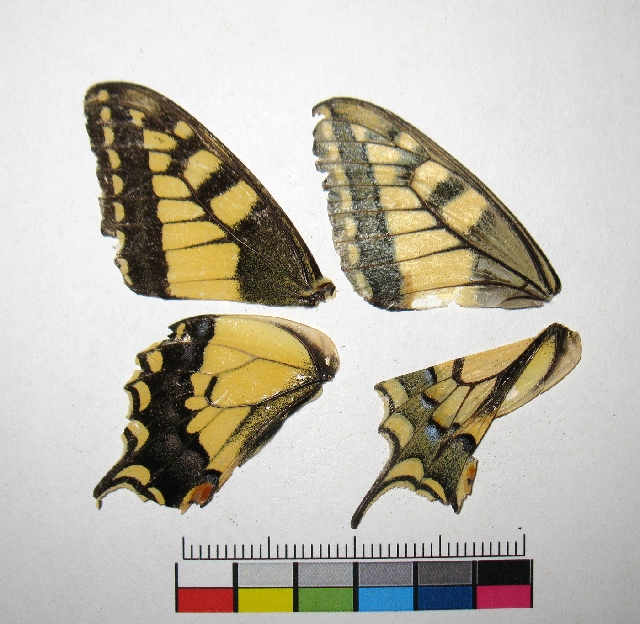

Supplement: S3 Fig — (ZIP) [file pone.0343793.s003.zip › S3/RVcoll.11-J350 .jpeg]

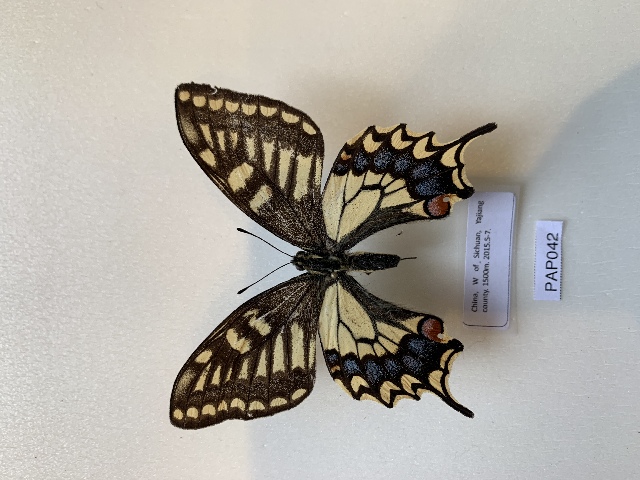

Supplement: S3 Fig — (ZIP) [file pone.0343793.s003.zip › S3/PAP042.jpeg]

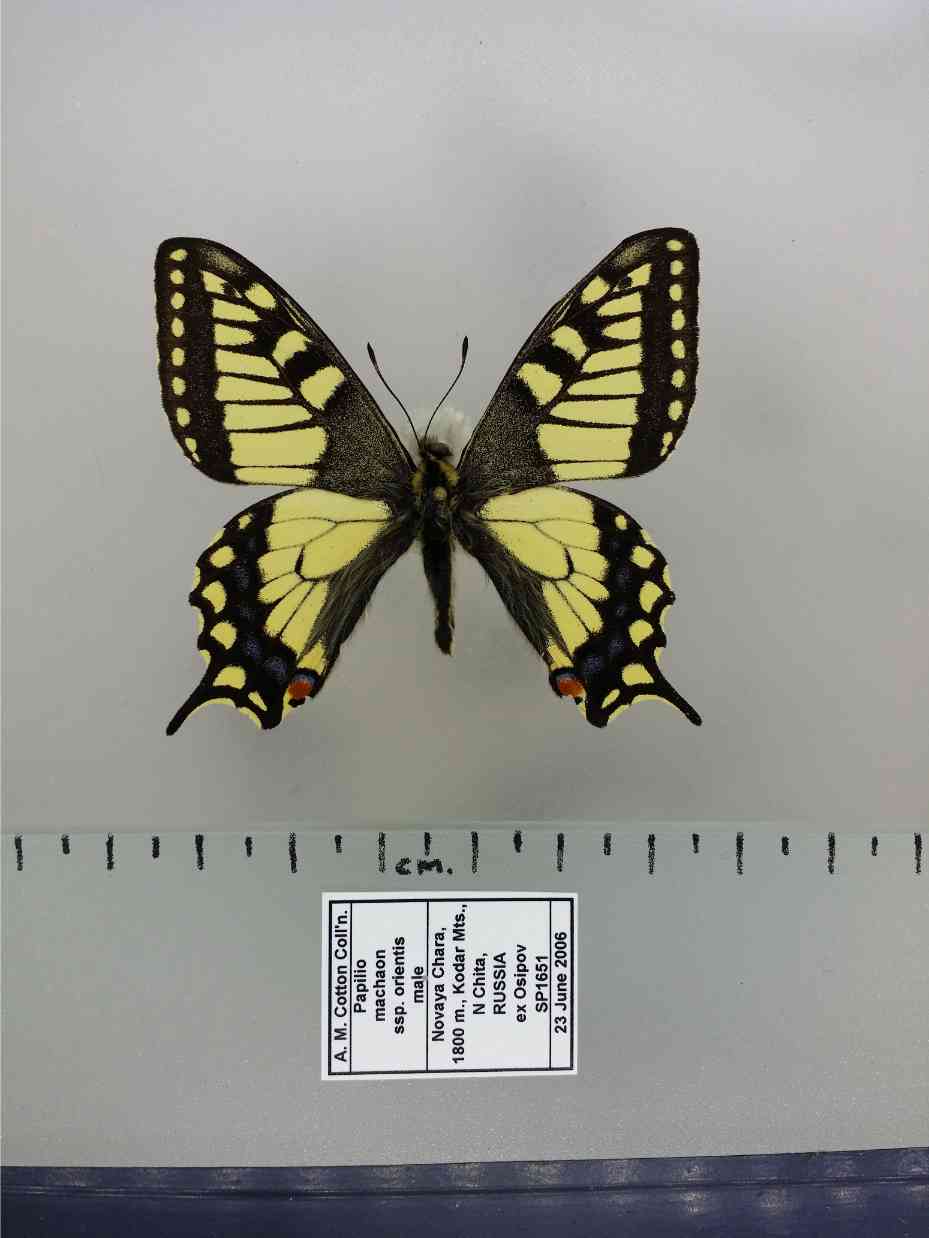

Supplement: S3 Fig — (ZIP) [file pone.0343793.s003.zip › S3/AC-SP1651D copy.jpg]

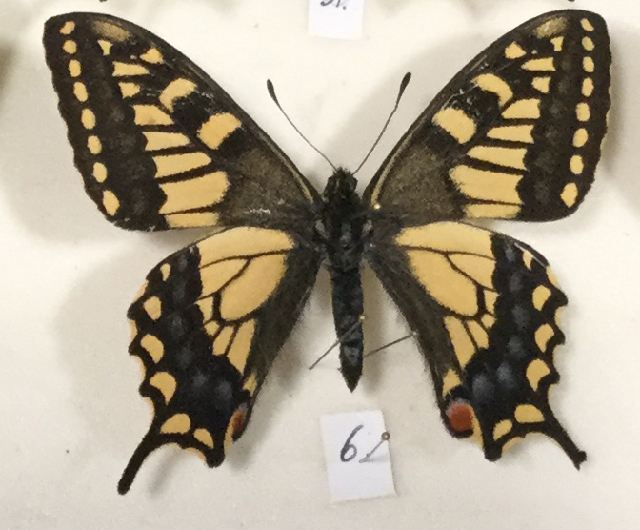

Supplement: S3 Fig — (ZIP) [file pone.0343793.s003.zip › S3/NS_106.jpeg]

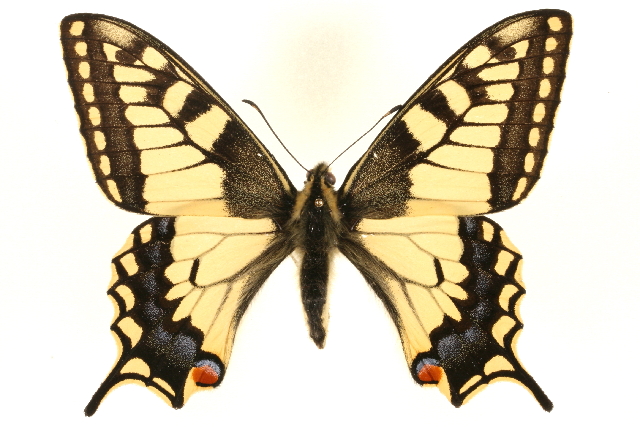

Supplement: S3 Fig — (ZIP) [file pone.0343793.s003.zip › S3/MM05450.jpeg]

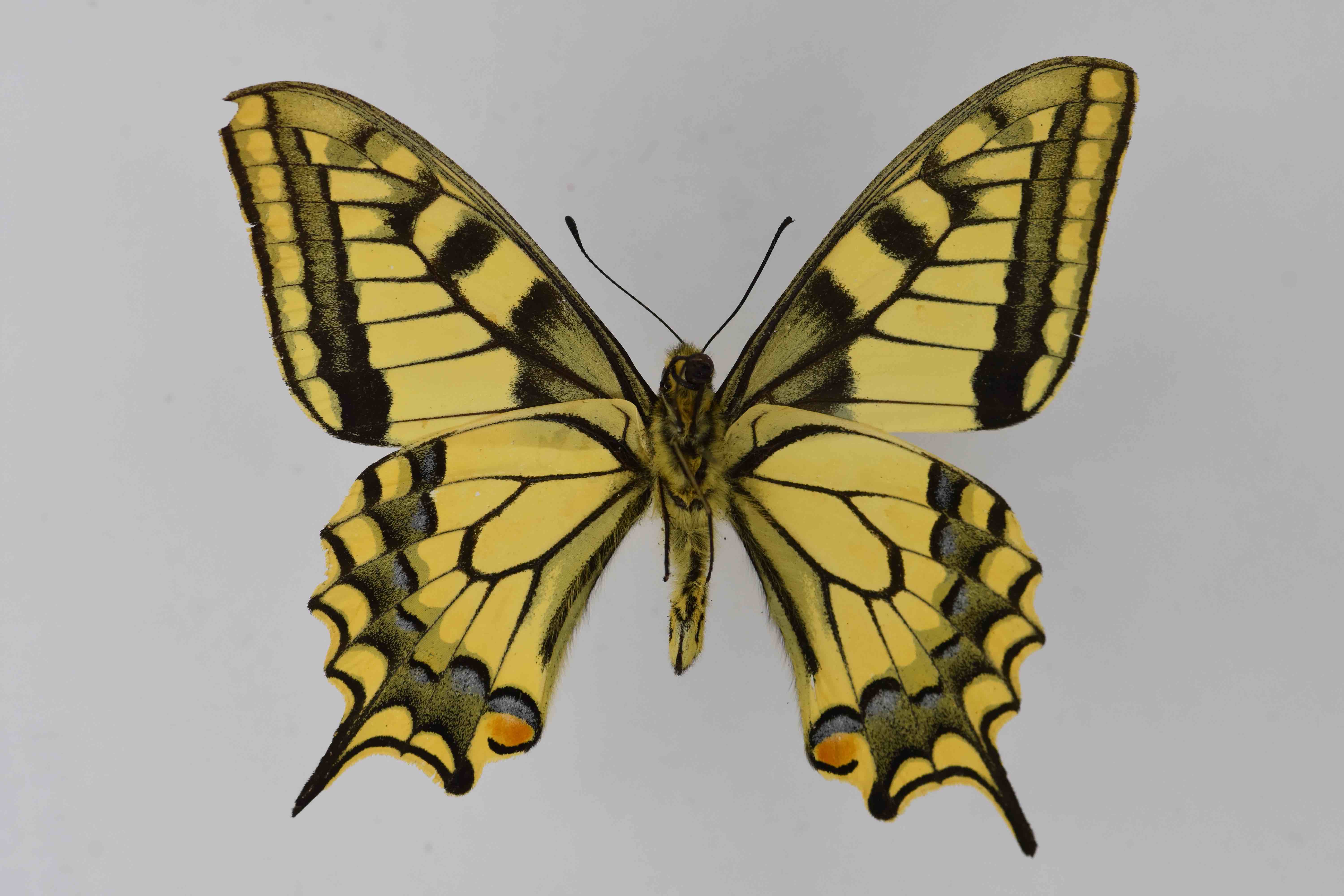

Supplement: S3 Fig — (ZIP) [file pone.0343793.s003.zip › S3/DNAwth003-V copy.jpeg]

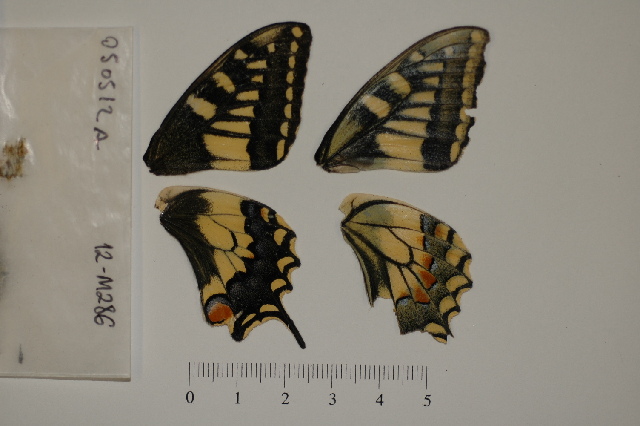

Supplement: S3 Fig — (ZIP) [file pone.0343793.s003.zip › S3/RVcoll.12-M286 .jpeg]

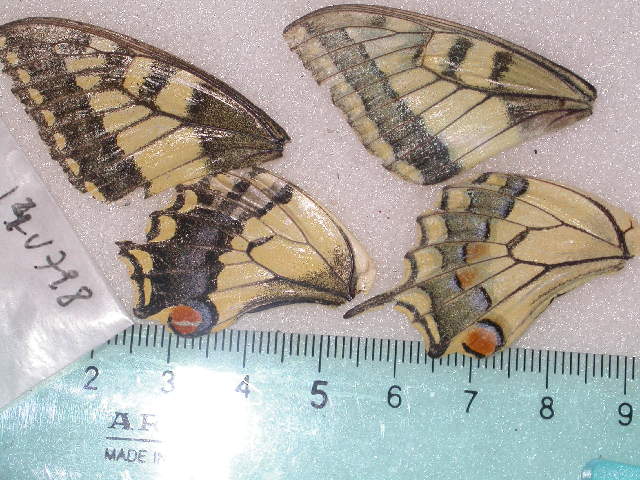

Supplement: S3 Fig — (ZIP) [file pone.0343793.s003.zip › S3/14-U798.jpeg]

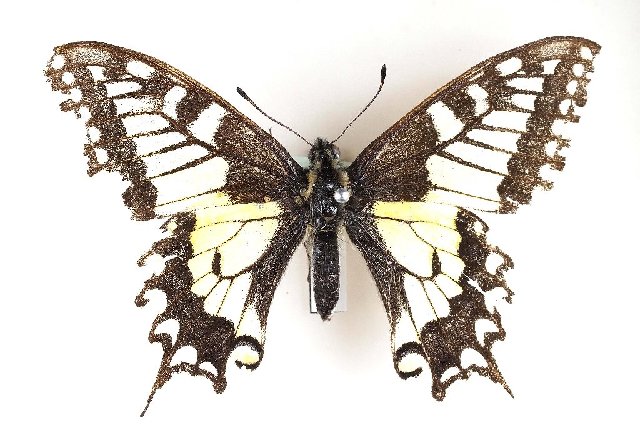

Supplement: S3 Fig — (ZIP) [file pone.0343793.s003.zip › S3/TLMF Lep 19628 .jpg]

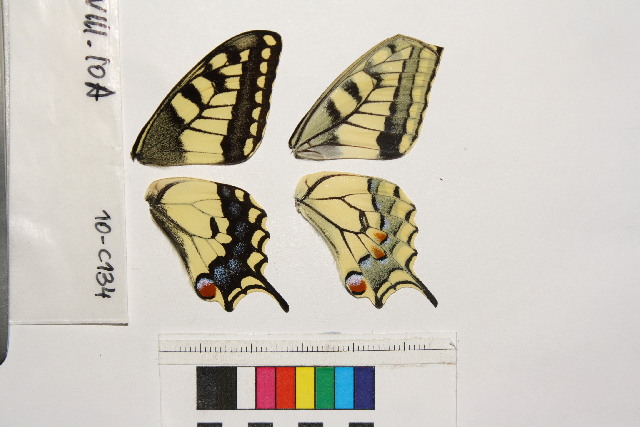

Supplement: S3 Fig — (ZIP) [file pone.0343793.s003.zip › S3/RVcoll.10-C134 .jpeg]
