# Supplementary material for: Integrative analyses reveal the evolution of the Old World Swallowtail in the Palearctic
Source: PLoS One. 2026 Jul 8;21(7):e0343793. doi: 10.1371/journal.pone.0343793 (PMC13345299; doi:10.1371/journal.pone.0343793)
Supplement: S4 Fig — Machine learning trained for 2000 epochs, incorporating an additional triplet balancing step to account for imbalanced sample sizes among label taxa. Final correlation coefficient at 2000 epochs: r = 0.76 (2 d. p.). (PDF) [file pone.0343793.s004.pdf]

S4 Fig. Supplementary machine learning analysis testing correlation between machine learnt embedding and genetic distances (corresponding to the phylogeny in Fig. 2). Machine learning trained for 2000 epochs, incorporating an additional triplet balancing step to account for imbalanced sample sizes among label taxa. Final correlation coefficient at 2000 epochs: $r = 0.76$ (2 d. p.).

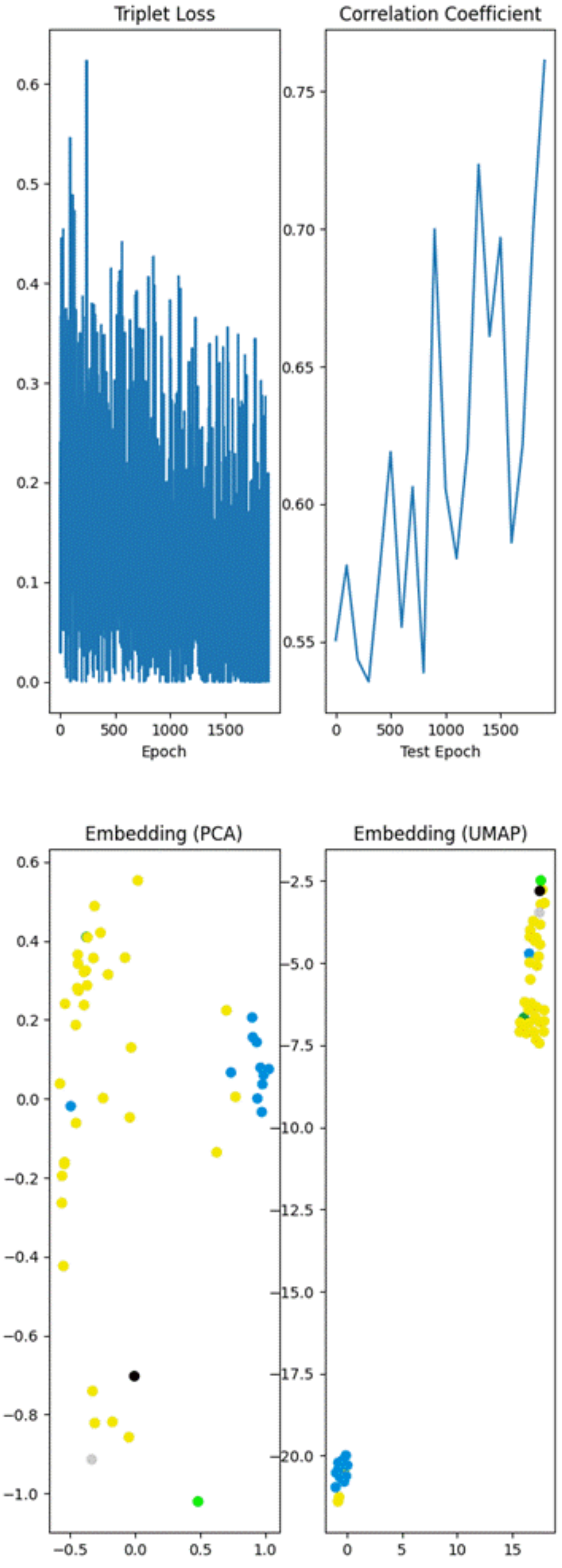

Triplet Loss
Epoch
0.6
0.5
0.4
0.3
0.2
0.1
0.0
0
500
1000
1500
Correlation Coefficient
Test Epoch
0.75
0.70
0.65
0.60
0.55
Embedding (PCA)
0.6
0.4
0.2
0.0
−0.2
−0.4
−0.6
−0.8
−1.0
−0.5
0.0
0.5
1.0
Embedding (UMAP)
−2.5
−5.0
−7.5
−10.0
−12.5
−15.0
−17.5
−20.0
0
5
10
15
