# Supplementary material for: Integrative analyses reveal the evolution of the Old World Swallowtail in the Palearctic
Source: PLoS One. 2026 Jul 8;21(7):e0343793. doi: 10.1371/journal.pone.0343793 (PMC13345299; doi:10.1371/journal.pone.0343793)
Supplement: S5 Fig — (PDF) [file pone.0343793.s005.pdf]

S5 Fig. Visualisation of the 245 swallowtail butterfly photographs used as input for the machine learning analysis (upper) at their locations in a 2D UMAP projection of the embedding (lower) corresponding to S4 Fig.

Photographs

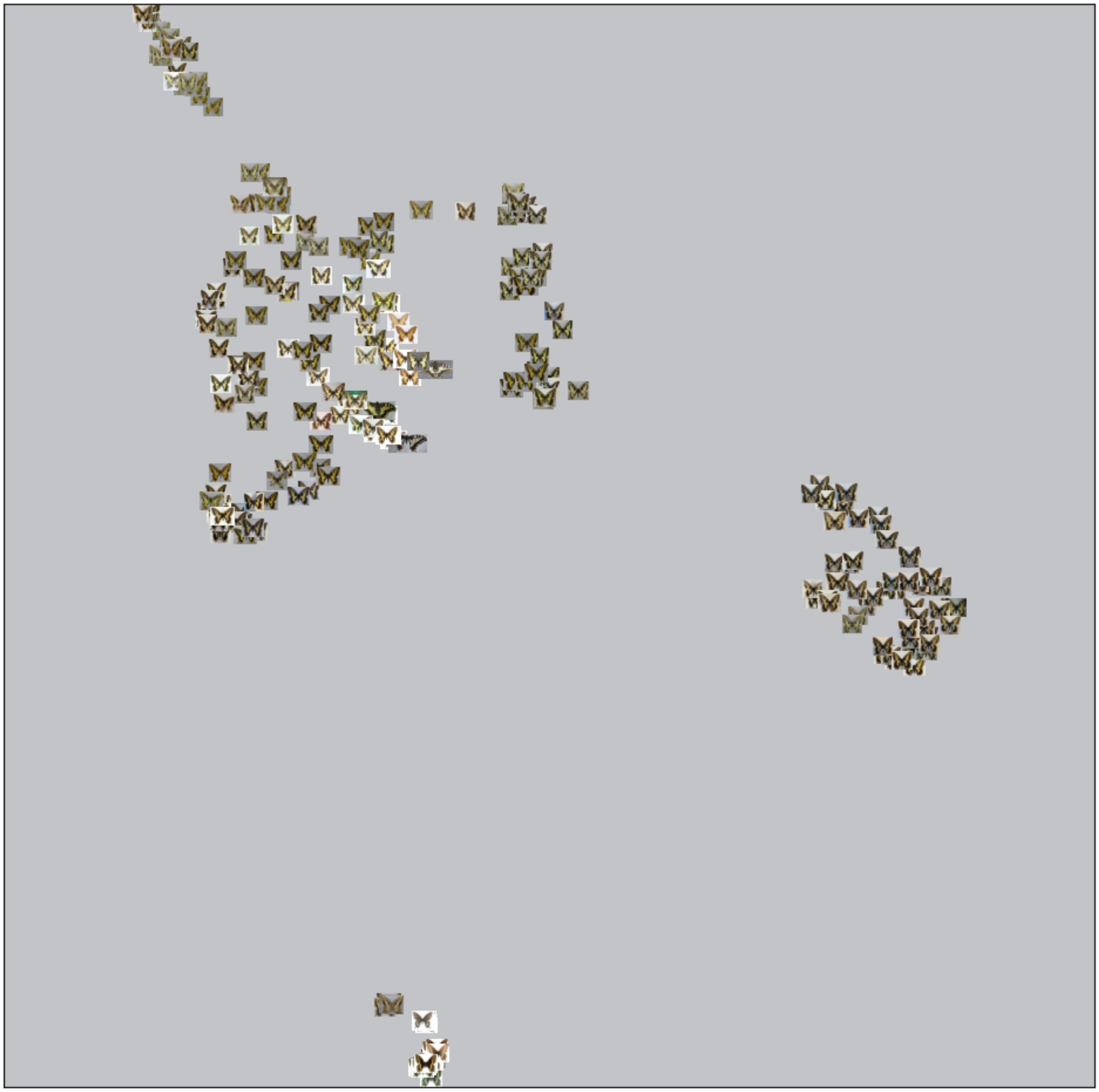

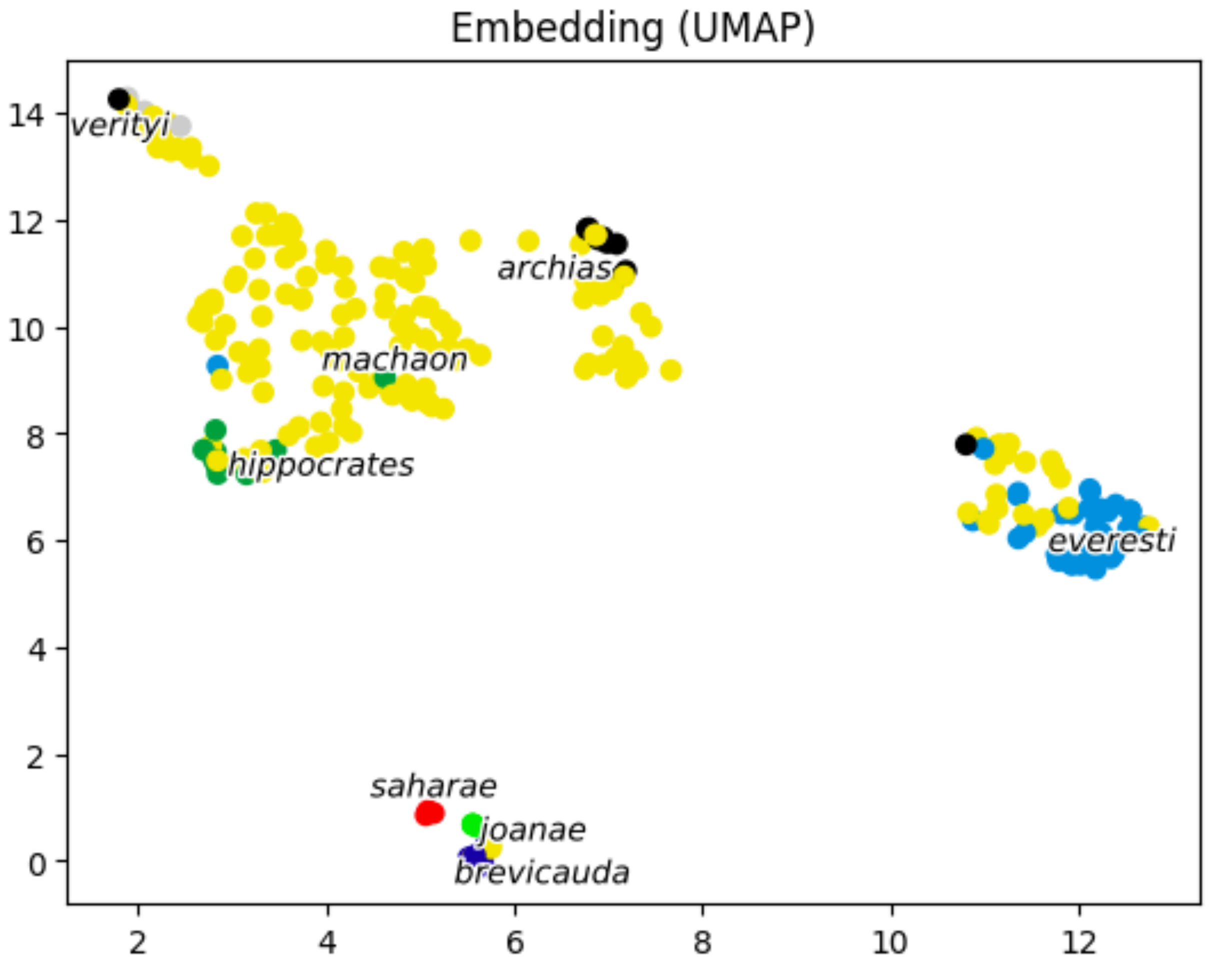

Embedding (UMAP)
verityi
archias
machaon
hippocrates
everesti
saharae
joanae
brevicauda
14
12
10
8
6
4
2
0
2
4
6
8
10
12
