# Supplementary material for: Integrative analyses reveal the evolution of the Old World Swallowtail in the Palearctic
Source: PLoS One. 2026 Jul 8;21(7):e0343793. doi: 10.1371/journal.pone.0343793 (PMC13345299; doi:10.1371/journal.pone.0343793)
Supplement: S6 Fig — (PDF) [file pone.0343793.s006.pdf]

S6 Fig. Phylogenetic trees showing placement of the Wolbachia strain retrieved from two female samples of Papilio archias among closely related 16S, wsp, ftsZ Wolbachia sequences mined from GenBank.

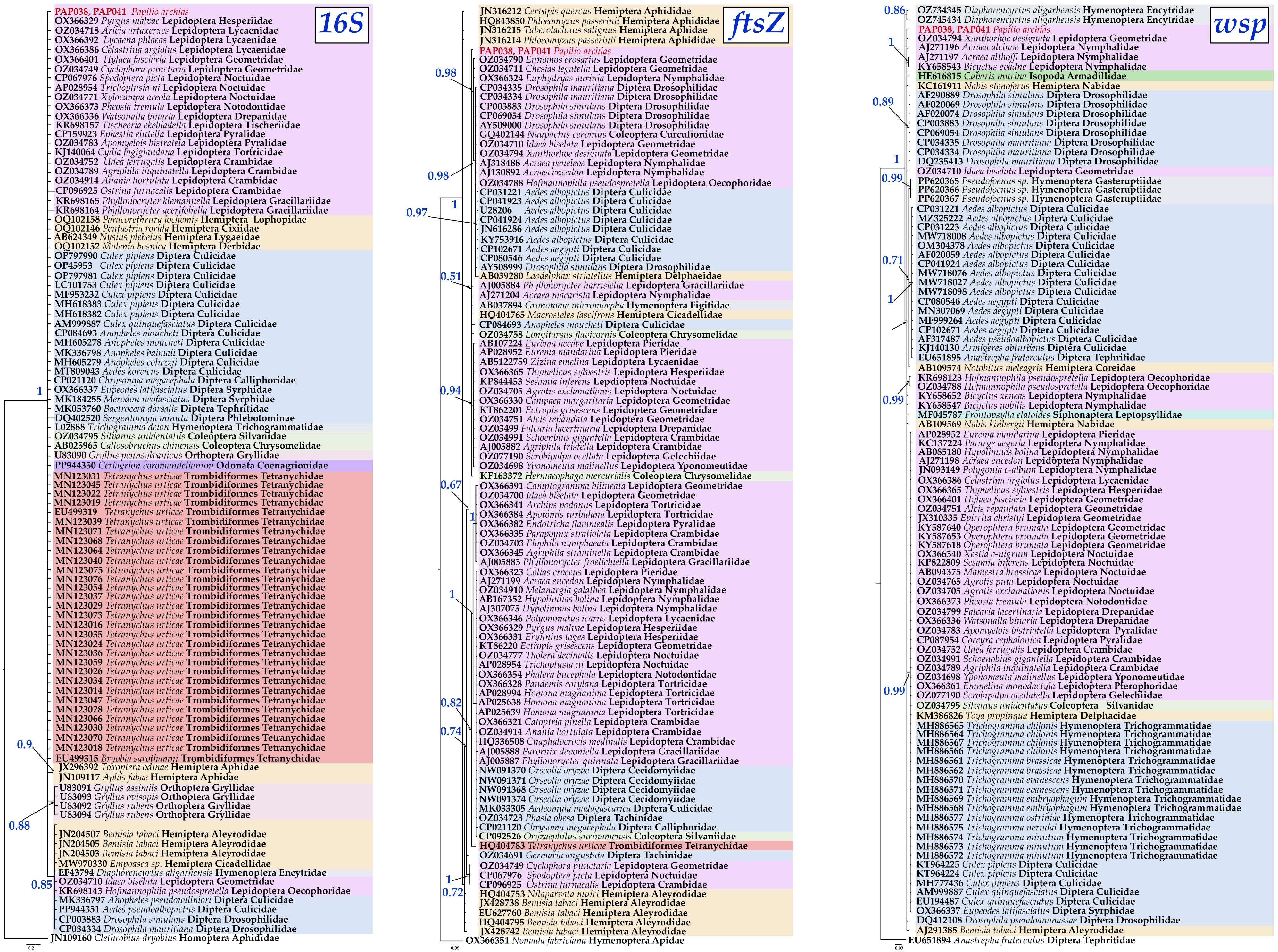
16S
PAP038, PAP041 Papilio archias
OX366329 Pyrgus malvae Lepidoptera Hesperiidae
OZ034718 Aricia artaxerxes Lepidoptera Lycaenidae
OX366392 Lycaena phlaeas Lepidoptera Lycaenidae
OX366386 Celastrina argiolus Lepidoptera Lycaenidae
OX366401 Hylaea fasciaria Lepidoptera Geometridae
OZ034749 Cyclophora punctaria Lepidoptera Geometridae
CP067976 Spodoptera picta Lepidoptera Noctuidae
AP028954 Trichoplusia ni Lepidoptera Noctuidae
OZ034771 Xylocampa areola Lepidoptera Noctuidae
OX366373 Pheosia tremula Lepidoptera Notodontidae
OX366336 Watsonalla binaria Lepidoptera Drepanidae
KR698157 Tischeeria ekebladella Lepidoptera Tischeriidae
CP159923 Ephestia elutella Lepidoptera Pyralidae
OZ034783 Apomyelois bistratela Lepidoptera Pyralidae
KJ140064 Cydia fagiglandana Lepidoptera Tortricidae
OZ034752 Udea ferrugalis Lepidoptera Crambidae
OZ034789 Agriphila inquinatella Lepidoptera Crambidae
OZ034914 Anania hortulata Lepidoptera Crambidae
CP096925 Ostrina furnacalis Lepidoptera Crambidae
KR698165 Phyllonorycter klemannella Lepidoptera Gracillariidae
KR698164 Phyllonorycter acerifoliella Lepidoptera Gracillariidae
OQ102158 Paracorethrura iochemis Hemiptera Lophopidae
OQ102146 Pentastria rorida Hemiptera Cixiidae
AB624349 Nysius plebeius Hemiptera Lygaeidae
OQ102152 Malenia bosnica Hemiptera Derbidae
OP797990 Culex pipiens Diptera Culicidae
OP45953 Culex pipiens Diptera Culicidae
OP797981 Culex pipiens Diptera Culicidae
LC101753 Culex pipiens Diptera Culicidae
MF953232 Culex pipiens Diptera Culicidae
MH618383 Culex pipiens Diptera Culicidae
MH618382 Culex pipiens Diptera Culicidae
AM999887 Culex quinquefasciatus Diptera Culicidae
CP084693 Anopheles moucheti Diptera Culicidae
MH605278 Anopheles moucheti Diptera Culicidae
MK336798 Anopheles baimaii Diptera Culicidae
MH605279 Anopheles coluzzii Diptera Culicidae
MT809043 Aedes koreicus Diptera Culicidae
CP021120 Chrysomya megacephala Diptera Calliphoridae
OX366337 Eupeodes latifasciatus Diptera Syrphidae
MK184255 Merodon neofasciatus Diptera Syrphidae
MK053760 Bactrocera dorsalis Diptera Tephritidae
DQ402520 Sergentomyia minuta Diptera Phlebotominae
L02888 Trichogramma deion Hymenoptera Trichogrammatidae
OZ034795 Silvanus unidentatus Coleoptera Silvanidae
AB025965 Callosobruchus chinensis Coleoptera Chrysomelidae
U83090 Gryllus pennsylvanicus Orthoptera Gryllidae
PP944350 Ceriagrion coromandelianum Odonata Coenagrionidae
MN123031 Tetranychus urticae Trombidiformes Tetranychidae
MN123045 Tetranychus urticae Trombidiformes Tetranychidae
MN123022 Tetranychus urticae Trombidiformes Tetranychidae
MN123019 Tetranychus urticae Trombidiformes Tetranychidae
EU499319 Tetranychus urticae Trombidiformes Tetranychidae
MN123039 Tetranychus urticae Trombidiformes Tetranychidae
MN123071 Tetranychus urticae Trombidiformes Tetranychidae
MN123068 Tetranychus urticae Trombidiformes Tetranychidae
MN123064 Tetranychus urticae Trombidiformes Tetranychidae
MN123040 Tetranychus urticae Trombidiformes Tetranychidae
MN123075 Tetranychus urticae Trombidiformes Tetranychidae
MN123076 Tetranychus urticae Trombidiformes Tetranychidae
MN123054 Tetranychus urticae Trombidiformes Tetranychidae
MN123037 Tetranychus urticae Trombidiformes Tetranychidae
MN123029 Tetranychus urticae Trombidiformes Tetranychidae
MN123073 Tetranychus urticae Trombidiformes Tetranychidae
MN123016 Tetranychus urticae Trombidiformes Tetranychidae
MN123035 Tetranychus urticae Trombidiformes Tetranychidae
MN123024 Tetranychus urticae Trombidiformes Tetranychidae
MN123036 Tetranychus urticae Trombidiformes Tetranychidae
MN123059 Tetranychus urticae Trombidiformes Tetranychidae
MN123026 Tetranychus urticae Trombidiformes Tetranychidae
MN123034 Tetranychus urticae Trombidiformes Tetranychidae
MN123014 Tetranychus urticae Trombidiformes Tetranychidae
MN123047 Tetranychus urticae Trombidiformes Tetranychidae
MN123028 Tetranychus urticae Trombidiformes Tetranychidae
MN123066 Tetranychus urticae Trombidiformes Tetranychidae
MN123030 Tetranychus urticae Trombidiformes Tetranychidae
MN123070 Tetranychus urticae Trombidiformes Tetranychidae
MN123018 Tetranychus urticae Trombidiformes Tetranychidae
EU499315 Bryobia sarothamni Trombidiformes Tetranychidae
JX296392 Toxoptera odinae Hemiptera Aphidae
JN109117 Aphis fabae Hemiptera Aphidae
U83091 Gryllus assimilis Orthoptera Gryllidae
U83093 Gryllus ovisopis Orthoptera Gryllidae
U83092 Gryllus rubens Orthoptera Gryllidae
U83094 Gryllus rubens Orthoptera Gryllidae
JN204507 Bemisia tabaci Hemiptera Aleyrodidae
JN204505 Bemisia tabaci Hemiptera Aleyrodidae
JN204503 Bemisia tabaci Hemiptera Aleyrodidae
MW970330 Empoasca sp. Hemiptera Cicadellidae
EF43794 Diaphorencyrtus aligarhensis Hymenoptera Encytridae
OZ034710 Idaea biselata Lepidoptera Geometridae
KR698143 Hofmannophila pseudospretella Lepidoptera Oecophoridae
MK336797 Anopheles pseudowillmori Diptera Culicidae
PP944351 Aedes pseudoalbopictus Diptera Culicidae
CP003883 Drosophila simulans Diptera Drosophilidae
CP034334 Drosophila mauritiana Diptera Drosophilidae
JN109160 Clethrobius dryobius Homoptera Aphididae
1
0.9
0.88
0.85
0.2
ftsZ
JN316212 Cervapis quercus Hemiptera Aphididae
HQ843850 Phloeomyzus passerinii Hemiptera Aphididae
JN316215 Tuberolachnus salignus Hemiptera Aphidae
JN316214 Phloeomyzus passerinii Hemiptera Aphididae
PAP038, PAP041 Papilio archias
OZ034790 Ennomos erosarius Lepidoptera Geometridae
OZ034711 Chesias legatella Lepidoptera Geometridae
OX366324 Euphydryas aurinia Lepidoptera Nymphalidae
CP034335 Drosophila mauritiana Diptera Drosophilidae
CP034334 Drosophila mauritiana Diptera Drosophilidae
CP003883 Drosophila simulans Diptera Drosophilidae
CP069054 Drosophila simulans Diptera Drosophilidae
AY509000 Drosophila simulans Diptera Drosophilidae
GQ402144 Naupactus cervinus Coleoptera Curculionidae
OZ034710 Idaea biselata Lepidoptera Geometridae
OZ034794 Xanthorhoe designata Lepidoptera Geometridae
AJ318488 Acraea peneleos Lepidoptera Nymphalidae
AJ130892 Acraea encedon Lepidoptera Nymphalidae
OZ034788 Hofmannophila pseudospretella Lepidoptera Oecophoridae
CP031221 Aedes albopictus Diptera Culicidae
CP041923 Aedes albopictus Diptera Culicidae
U28206 Aedes albopictus Diptera Culicidae
CP041924 Aedes albopictus Diptera Culicidae
JN616286 Aedes albopictus Diptera Culicidae
KY753916 Aedes albopictus Diptera Culicidae
CP102671 Aedes aegypti Diptera Culicidae
CP080546 Aedes aegypti Diptera Culicidae
AY508999 Drosophila simulans Diptera Drosophilidae
AB039280 Laodelphax striatellus Hemiptera Delphaeidae
AJ005884 Phyllonorycter harrisiella Lepidoptera Gracillariidae
AJ271204 Acraea macarista Lepidoptera Nymphalidae
AB037894 Gronotoma micromorpha Hymenoptera Figitidae
HQ404765 Macrosteles fascifrons Hemiptera Cicadellidae
CP084693 Anopheles moucheti Diptera Culicidae
OZ034758 Longitarsus flavicornis Coleoptera Chrysomelidae
AB107224 Eurema hecabe Lepidoptera Pieridae
AP028952 Eurema mandarina Lepidoptera Pieridae
AB5122759 Zizina emelina Lepidoptera Lycaenidae
OX366365 Thymelicus sylvestris Lepidoptera Hesperiidae
KP844453 Sesamia inferens Lepdioptera Noctuidae
OZ034705 Agrotis exclamationis Lepidoptera Noctuidae
OX366330 Campaea margaritaria Lepidoptera Geometridae
KT862201 Ectropis grisescens Lepidoptera Geometridae
OZ034751 Alcis repandata Lepidoptera Geometridae
OZ03499 Falcaria lacertinaria Lepidoptera Drepanidae
OZ034991 Schoenbius gigantella Lepidoptera Crambidae
AJ005882 Agriphila tristella Lepidoptera Crambidae
OZ077190 Scrobipalpa ocellata Lepidoptera Gelechiidae
OZ034698 Yponomeuta malinellus Lepidoptera Yponomeutidae
KF163372 Hermaeophaga mercurialis Coleoptera Chrysomelidae
OX366391 Camptogramma bilineata Lepidoptera Geometridae
OZ034700 Idaea biselata Lepidoptera Geometridae
OX366341 Archips podanus Lepidoptera Tortricidae
OX366384 Apotomis turbidana Lepidoptera Tortricidae
OX366382 Endotricha flammealis Lepidoptera Pyralidae
OX366335 Parapoynx stratiolata Lepidoptera Crambidae
OZ034703 Elophila nymphaeata Lepidoptera Crambidae
OX366345 Agriphila straminella Lepidoptera Crambidae
AJ005883 Phyllonorycter froelichiella Lepidoptera Gracillariidae
OX366323 Colias croceus Lepidoptera Pieridae
AJ271199 Acraea encedon Lepidoptera Nymphalidae
OZ034910 Melanargia galathea Lepidoptera Nymphalidae
AB167352 Hypolimnas bolina Lepidoptera Nymphalidae
AJ307075 Hypolimnas bolina Lepidoptera Nymphalidae
OX366346 Polyommatus icarus Lepidoptera Lycaenidae
OX366329 Pyrgus malvae Lepidoptera Hesperiidae
OX366331 Erynnins tages Lepidoptera Hesperiidae
KT86220 Ectropis grisescens Lepidoptera Geometridae
OZ034777 Tholera decimalis Lepidoptera Noctuidae
AP028954 Trichoplusia ni Lepidoptera Noctuidae
OX366354 Phalera bucephala Lepidoptera Notodontidae
OX366328 Pandemis corylana Lepidoptera Tortricidae
AP028994 Homona magnanima Lepidoptera Tortricidae
AP025638 Homona magnanima Lepidoptera Tortricidae
AP025639 Homona magnanima Lepidoptera Tortricidae
OX366321 Catoptria pinella Lepidoptera Crambidae
OZ034914 Anania hortulata Lepidoptera Crambidae
HQ336508 Cnaphalocrocis medinalis Lepidoptera Crambidae
AJ005888 Parornix devoniella Lepidoptera Gracillariidae
AJ005887 Phyllonorycter quinnata Lepidoptera Gracillariidae
NW091370 Orseolia oryzae Diptera Cecidomyiidae
NW091371 Orseolia oryzae Diptera Cecidomyiidae
NW091368 Orseolia oryzae Diptera Cecidomyiidae
NW091374 Orseolia oryzae Diptera Cecidomyiidae
MK033305 Aedeomyia madagascarica Diptera Culicidae
OZ034723 Phasia obesa Diptera Tachinidae
CP021120 Chrysoma megacephala Diptera Calliphoridae
CP092526 Oryzaephilus surinamensis Coleoptera Silvaniidae
HQ404783 Tetranychus urticae Trombidiformes Tetranychidae
OZ034691 Germaria angustata Diptera Tachinidae
OZ034749 Cyclophora punctaria Lepidoptera Geometridae
CP067976 Spodoptera picta Lepidoptera Noctuidae
CP096925 Ostrina furnacalis Lepidoptera Crambidae
HQ404753 Nilaparvata muiri Hemiptera Aleyrodidae
JX428738 Bemisia tabaci Hemiptera Aleyrodidae
EU627760 Bemisia tabaci Hemiptera Aleyrodidae
HQ404795 Bemisia tabaci Hemiptera Aleyrodidae
JX428742 Bemisia tabaci Hemiptera Aleyrodidae
OX366351 Nomada fabriciana Hymenoptera Apidae
0.98
0.98
1
0.97
0.51
0.94
0.67
1
1
0.82
0.74
1
0.72
0.08
wsp
OZ734345 Diaphorencyrtus aligarhensis Hymenoptera Encytridae
OZ745434 Diaphorencyrtus aligarhensis Hymenoptera Encytridae
PAP038, PAP041 Papilio archias
OZ034794 Xanthorhoe designata Lepidoptera Geometridae
AJ271196 Acraea alcinoe Lepidoptera Nymphalidae
AJ271197 Acraea althoffi Lepidoptera Nymphalidae
KY658543 Bicyclus evadne Lepidoptera Nymphalidae
HE616815 Cubaris murina Isopoda Armadillidae
KC161911 Nabis stenoferus Hemiptera Nabidae
AF290889 Drosophila simulans Diptera Drosophilidae
AF020069 Drosophila simulans Diptera Drosophilidae
AF020074 Drosophila simulans Diptera Drosophilidae
CP003883 Drosophila simulans Diptera Drosophilidae
CP069054 Drosophila simulans Diptera Drosophilidae
CP034335 Drosophila mauritiana Diptera Drosophilidae
CP034334 Drosophila mauritiana Diptera Drosophilidae
DQ235413 Drosophila mauritiana Diptera Drosophilidae
OZ034710 Idaea biselata Lepidoptera Geometridae
PP620365 Pseudofoenus sp. Hymenoptera Gasteruptiidae
PP620366 Pseudofoenus sp. Hymenoptera Gasteruptiidae
PP620367 Pseudofoenus sp. Hymenoptera Gasteruptiidae
CP031221 Aedes albopictus Diptera Culicidae
MZ325222 Aedes albopictus Diptera Culicidae
CP031223 Aedes albopictus Diptera Culicidae
MW718008 Aedes albopictus Diptera Culicidae
OM304378 Aedes albopictus Diptera Culicidae
AF020059 Aedes albopictus Diptera Culicidae
CP041924 Aedes albopictus Diptera Culicidae
MW718076 Aedes albopictus Diptera Culicidae
MW718027 Aedes albopictus Diptera Culicidae
MW718098 Aedes albopictus Diptera Culicidae
CP080546 Aedes aegypti Diptera Culicidae
MN307069 Aedes aegypti Diptera Culicidae
MF999264 Aedes aegypti Diptera Culicidae
CP102671 Aedes aegypti Diptera Culicidae
AF317487 Aedes pseudoalbopictus Diptera Culicidae
KJ140130 Armigeres obturbans Diptera Culicidae
EU651895 Anastrepha fraterculus Diptera Tephritidae
AB109574 Notobitus meleagris Hemiptera Coreidae
KR698123 Hofmannophila pseudospretella Lepidoptera Oecophoridae
OZ034788 Hofmannophila pseudospretella Lepidoptera Oecophoridae
KY658652 Bicyclus xeneas Lepidoptera Nymphalidae
KY658547 Bicyclus nobilis Lepidoptera Nymphalidae
MF045787 Frontopsylla elatoides Siphonaptera Leptopsyllidae
AB109569 Nabis kinbergii Hemiptera Nabidae
AP028952 Eurema mandarina Lepidoptera Pieridae
KC137224 Pararge aegeria Lepidoptera Nymphalidae
AB085180 Hypolimnas bolina Lepidoptera Nymphalidae
AJ271198 Acraea encedon Lepidoptera Nymphalidae
JN093149 Polygonia c-album Lepidoptera Nymphalidae
OX366386 Celastrina argiolus Lepidoptera Lycaenidae
OX366365 Thymelicus sylvestris Lepidoptera Hesperiidae
OX366401 Hylaea fasciaria Lepidoptera Geometridae
OZ034751 Alcis repandata Lepidoptera Geometridae
JX310335 Epirrita christyi Lepidoptera Geometridae
KY587640 Operophtera brumata Lepidoptera Geometridae
KY587653 Operophtera brumata Lepidoptera Geometridae
KY587618 Operophtera brumata Lepidoptera Geometridae
OX366340 Xestia c-nigrum Lepidoptera Noctuidae
KP822809 Sesamia inferens Lepidoptera Noctuidae
AB094375 Mamestra brassicae Lepidoptera Noctuidae
OZ034765 Agrotis puta Lepidoptera Noctuidae
OZ034705 Agrotis exclamationis Lepidoptera Noctuidae
OX366373 Pheosia tremula Lepidoptera Notodontidae
OZ034799 Falcaria lacertinaria Lepidoptera Drepanidae
OX366336 Watsonalla binaria Lepidoptera Drepanidae
OZ034783 Apomyelois bistriatella Lepidoptera Pyralidae
CP087954 Corcyra cephalonica Lepidoptera Pyralidae
OZ034752 Udea ferrugalis Lepidoptera Crambidae
OZ034991 Schoenobius gigantella Lepidoptera Crambidae
OZ034789 Agriphila inquinatella Lepidoptera Crambidae
OZ034698 Yponomeuta malinellus Lepidoptera Yponomeutidae
OX366361 Emmelina monodactyla Lepidoptera Pterophoridae
OZ077190 Scrobipalpa ocellatella Lepidoptera Gelechiidae
OZ034795 Silvanus unidentatus Coleoptera Silvanidae
KM386826 Toya propinqua Hemiptera Delphacidae
MH886565 Trichogramma chilonis Hymenoptera Trichogrammatidae
MH886564 Trichogramma chilonis Hymenoptera Trichogrammatidae
MH886567 Trichogramma chilonis Hymenoptera Trichogrammatidae
MH886566 Trichogramma chilonis Hymenoptera Trichogrammatidae
MH886561 Trichogramma brassicae Hymenoptera Trichogrammatidae
MH886562 Trichogramma brassicae Hymenoptera Trichogrammatidae
MH886570 Trichogramma evanescens Hymenoptera Trichogrammatidae
MH886571 Trichogramma evanescens Hymenoptera Trichogrammatidae
MH886569 Trichogramma embryophagum Hymenoptera Trichogrammatidae
MH886568 Trichogramma embryophagum Hymenoptera Trichogrammatidae
MH886577 Trichogramma ostriniae Hymenoptera Trichogrammatidae
MH886575 Trichogramma nerudai Hymenoptera Trichogrammatidae
MH886574 Trichogramma minutum Hymenoptera Trichogrammatidae
MH886573 Trichogramma minutum Hymenoptera Trichogrammatidae
MH886572 Trichogramma minutum Hymenoptera Trichogrammatidae
KT964225 Culex pipiens Diptera Culicidae
KT964224 Culex pipiens Diptera Culicidae
MH777436 Culex pipiens Diptera Culicidae
AM999887 Culex quinquefasciatus Diptera Culicidae
EU194487 Culex quinquefasciatus Diptera Culicidae
OX366337 Eupeodes latifasciatus Diptera Syrphidae
DQ412108 Drosophila pseudoananassae Diptera Drosophilidae
AJ291385 Bemisia tabaci Hemiptera Aleyrodidae
EU651894 Anastrepha fraterculus Diptera Tephritidae
0.86
1
0.89
1
0.99
0.71
1
0.99
0.99
0.03
