# Supplementary material for: Integrative analyses reveal the evolution of the Old World Swallowtail in the Palearctic
Source: PLoS One. 2026 Jul 8;21(7):e0343793. doi: 10.1371/journal.pone.0343793 (PMC13345299; doi:10.1371/journal.pone.0343793)

S7 Fig. Results of the ASAP analysis.

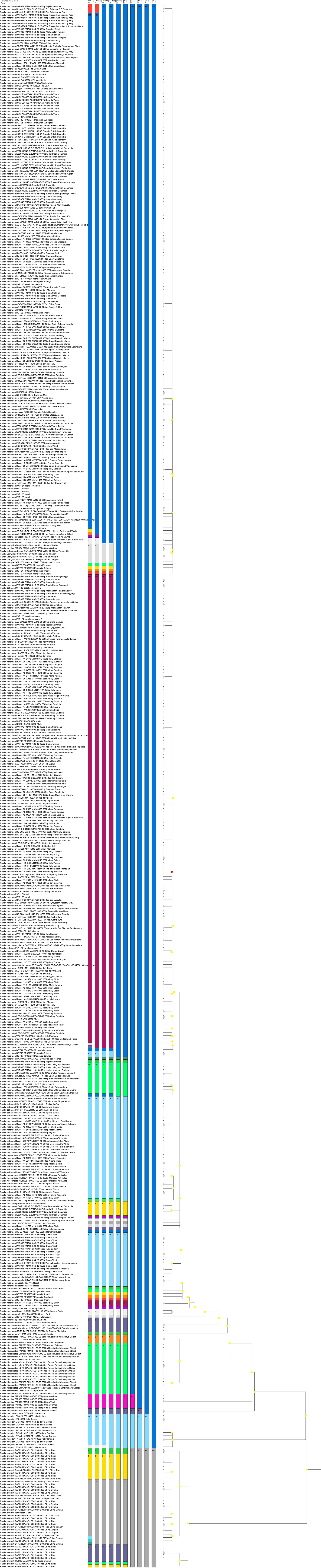

Supplement: S7 Fig — (PDF) [file pone.0343793.s007.pdf]
