# Supplementary figures and images for: Integrative analyses reveal the evolution of the Old World Swallowtail in the Palearctic (part 3 of 3)
Source: PLoS One. 2026 Jul 8;21(7):e0343793. doi: 10.1371/journal.pone.0343793 (PMC13345299; doi:10.1371/journal.pone.0343793)

S8 Fig. IQTree.

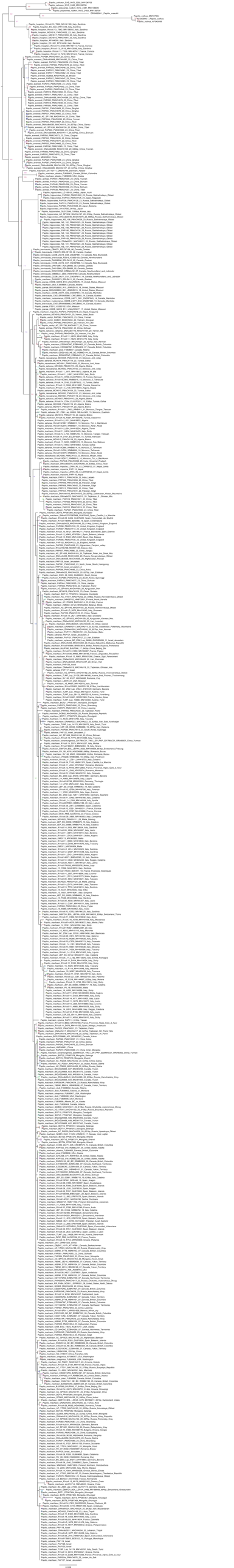

Supplement: S8 Fig — (PDF) [file pone.0343793.s008.pdf]

**S9 Fig.** Mr. Bayes tree.

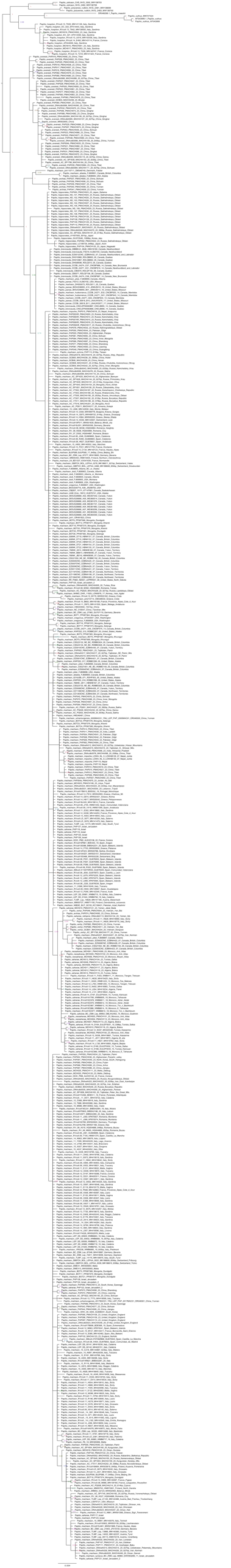

Supplement: S9 Fig — (PDF) [file pone.0343793.s009.pdf]

**S10 Fig.** Tree from BEAST analysis.

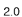

Supplement: S10 Fig — (PDF) [file pone.0343793.s010.pdf]
