# Supplementary material for: Integrative analyses reveal the evolution of the Old World Swallowtail in the Palearctic
Source: PLoS One. 2026 Jul 8;21(7):e0343793. doi: 10.1371/journal.pone.0343793 (PMC13345299; doi:10.1371/journal.pone.0343793)
Supplement: S11 Fig — Latitude and longitude of swallowtail butterfly specimens included in the machine learning image analyses, with points coloured by country. Pairwise comparison of Euclidean distances machine learnt from 245 specimen photographs and the geographic latitude or longitude associated with that specimen. Geographic distance is the geodesic distance calculated using the scipy and geopy packages. Machine learning results correspond to those of SI S7. Pearson correlation p < 0.001, r = 0.344 (3 d. p.). (PDF) [file pone.0343793.s011.pdf]

S11 Fig. Comparisons of latitude, longitude and machine learnt image distances. Latitude and longitude of swallowtail butterfly specimens included in the machine learning image analyses, with points coloured by country. Pairwise comparison of Euclidean distances machine learnt from 245 specimen photographs and the geographic latitude or longitude associated with that specimen. Geographic distance is the geodesic distance calculated using the scipy and geopy packages. Machine learning results correspond to those of SI S7. Pearson correlation $p < 0.001$, $r = 0.344$ (3 d. p.)

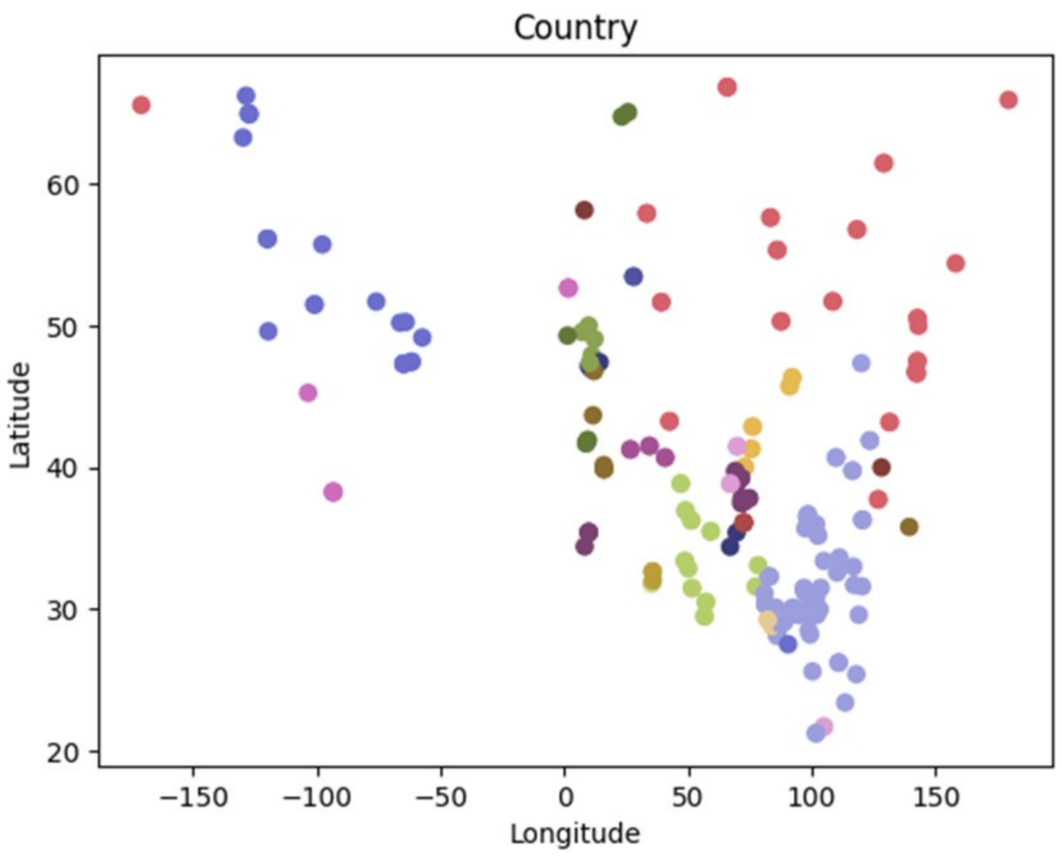
Country
Latitude
Longitude
60
50
40
30
20
−150
−100
−50
0
50
100
150

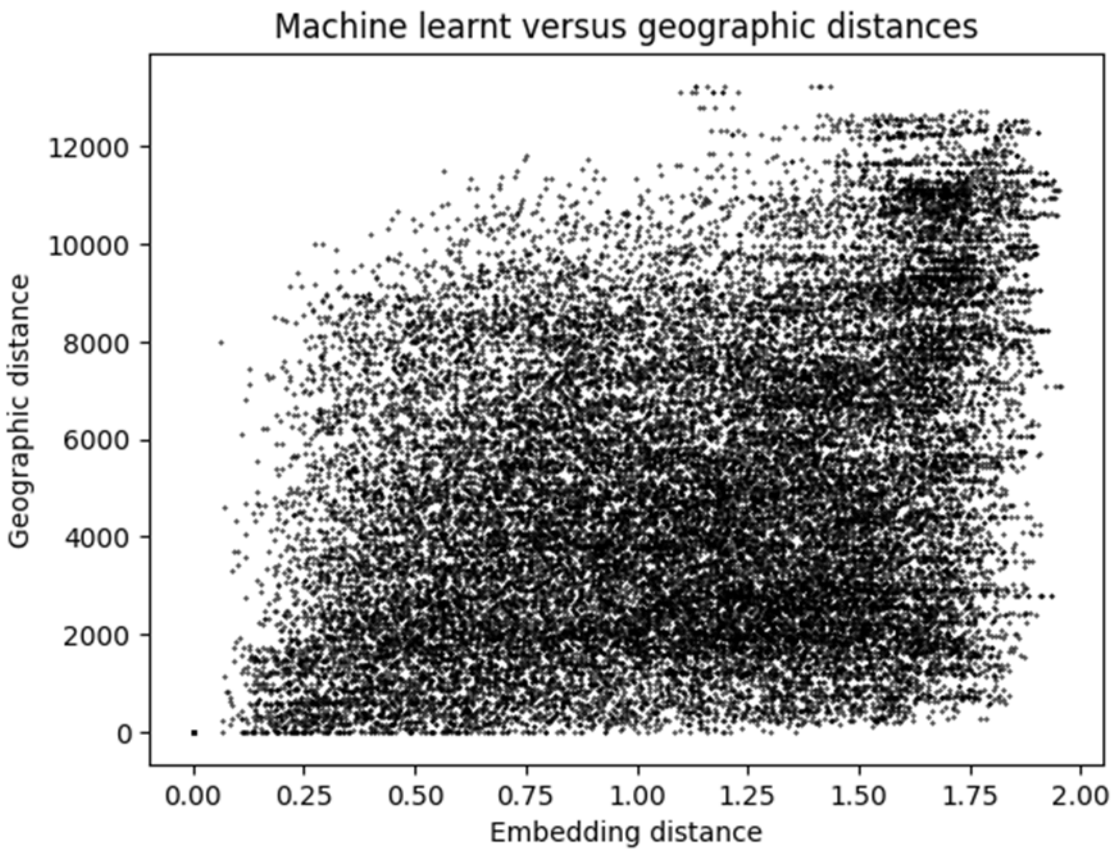
Machine learnt versus geographic distances
Geographic distance
Embedding distance
12000
10000
8000
6000
4000
2000
0
0.00
0.25
0.50
0.75
1.00
1.25
1.50
1.75
2.00
